# A revision of the New Zealand *Kunzea ericoides* (Myrtaceae) complex

**DOI:** 10.3897/phytokeys.40.7973

**Published:** 2014-08-26

**Authors:** Peter J. de Lange

**Affiliations:** 1 Science & Capability Group, Terrestrial Ecosystems, Department of Conservation, Private Bag 68908 Newton, Auckland 1145, New Zealand

**Keywords:** New Zealand Archipelago, Myrtaceae, *Kunzea*, *Kunzea ericoides*, *Kunzea sinclairii*, new combination, *Kunzea linearis* comb. nov., new species, *Kunzea amathicola* sp. nov., *Kunzea triregensis* sp. nov., *Kunzea robusta* sp. nov., *Kunzea salterae* sp. nov., *Kunzea serotina* sp. nov., *Kunzea tenuicaulis* sp. nov., *Kunzea toelkenii* sp. nov., typifications, *Leptospermum ericoides*, Leptospermum ericoides var. lineare, Leptospermum ericoides var. microflorum, Leptospermum ericoides var. pubescens, *Leptospermum sinclairii*, ecology, conservation, ethnobotany

## Abstract

A revision of the New Zealand *Kunzea ericoides* complex is presented. This paper is the final of a series that has explored the systematics of the New Zealand *Kunzea* complex using cytological and molecular variation, as well as experimental hybridisations between postulated segregates. As a result of those studies ten species, all endemic to New Zealand, are recognised; seven of these are new. One species, *Kunzea triregensis* **sp. nov.**, is endemic to the Three Kings Islands and another species *Kunzea sinclairii*, endemic to Aotea (Great Barrier Island). The North Island of New Zealand has seven species, *Kunzea amathicola* **sp. nov.**, *Kunzea salterae* **sp. nov.**, *Kunzea serotina* **sp. nov.**, *Kunzea robusta* **sp. nov.**, *Kunzea tenuicaulis* **sp. nov.**, *Kunzea toelkenii* **sp. nov.**, and *Kunzea linearis* **comb. nov.** Of these, *Kunzea linearis*, *Kunzea salterae*, *Kunzea tenuicaulis* and *Kunzea toelkenii* are endemic to the North Island, and *Kunzea amathicola*, *Kunzea robusta* and *Kunzea serotina* extend to the South Island which also supports one endemic, *Kunzea ericoides*. Typifications are published for *Leptospermum ericoides* A.Rich., Leptospermum ericoides var. linearis Kirk, Leptospermum ericoides var. microflorum G.Simps., Leptospermum ericoides var. pubescens Kirk, and *Leptospermum sinclairii* Kirk, names here all referred to *Kunzea*. The ecology, conservation, extent of natural hybridisation and some aspects of the ethnobotany (vernacular names) of these *Kunzea* are also discussed.

## Introduction

It has long been recognised that New Zealand populations of *Kunzea ericoides* (A.Rich) Joy Thomps. are extremely variable (Hooker 1867; Cheeseman 1906, 1925; Allan 1961; Harris 1996; de Lange and Murray 2004, de Lange et al. 2005). Nevertheless, despite attempts to formally describe this variation (Kirk 1869, 1889, 1899; Simpson 1945), there has been no critical revision of the complex as a whole. The most modern treatments available favour either a single broadly circumscribed Australasian species, *Kunzea ericoides* (Thompson 1983, Wilson 1991), or two species: the Australasian *Kunzea ericoides*, and a narrow-range New Zealand endemic *Kunzea sinclairii* (Kirk) W.Harris (Harris 1987; Harris et al. 1992). Within New Zealand, *Kunzea ericoides* was further subdivided into three varieties by Harris (1987) who made combinations in *Kunzea* at the rank of variety for two New Zealand taxa previously regarded as varieties of Leptospermum ericoides (var. linearis Kirk and var. microflorum G.Simpson), and which had been treated by Thompson (1983) as synonyms of *Kunzea ericoides*. Subsequent research by Harris (1996) into the variation within *Kunzea ericoides* using ‘genecology’ investigated flowering patterns within cultivated ‘populations’ of *Kunzea ericoides* *sens. lat.* and *Kunzea sinclairii*. Harris used 25 New Zealand and two Australian sources for his *Kunzea ericoides* plants and one from Aotea (Great Barrier Island) for *Kunzea sinclairii*. Although Harris (1996) adopted a broad treatment of *Kunzea ericoides* he noted different flowering patterns for his two Australian population samples, and, furthermore, he observed that these samples differed from the New Zealand populations in having larger leaves and capsules. Of the New Zealand populations of *Kunzea ericoides*, he suggested that, aside from Kunzea ericoides var. microflora and var. linearis (samples of which he lacked in his study), there appeared to be two further taxonomic entities present based on leaf form and geographic distribution. One of these, his “southern taxon”, was characterised as having small leaves and was confined to stations south of Latitude 38°S. Within it he suggested that Kunzea ericoides var. microflora could be included, as it differed only by its prostrate habit and restriction to geothermal habitats. The other entity he noted was more northern and that it differed from Kunzea ericoides var. linearis, with which it sometimes grew, by its larger leaves. He further opined that within his southern taxon there may be ‘geographical races’, citing for example, comments made by Allan (1961) of a ‘thicket-forming variant’ characteristic of montane areas in Marlborough. Although Harris did not take these studies further, he recognised that there were distinct, genetically determined and geographically related patterns of variation with *Kunzea ericoides*.

Since 1999 I have used morphological, cytological, and DNA (both rDNA and cpDNA) sequence data in conjunction with hybridisation experiments to investigate the variation within the mainly New Zealand members of the *Kunzea ericoides* complex (de Lange 2006; de Lange 2007; de Lange and Murray 2004; de Lange et al. 2005; de Lange et al. 2010). Collectively these papers conclude that the current broad circumscription of *Kunzea ericoides* as a single species does not adequately address the variation found within that species in New Zealand or indeed Australia. This paper, summarising cytogenetic and molecular evidence published in previous papers (de Lange and Murray 2004; de Lange et al. 2005; de Lange et al. 2010), and morphological and field evidence, addresses this variation and offers a full taxonomic revision of the New Zealand *Kunzea ericoides* complex. The Australian members of the *Kunzea ericoides* complex are being revised independently by H.R. Toelken (pers. comm.). Ten species (seven described here for the first time), all endemic to New Zealand, are recognised. For ease of readability, names for all new taxa and combinations are used throughout the Methods and Characters sections prior to their formal description.

## Materials and methods

At the onset of this investigation in 1999, it became apparent that the then available herbarium collections of New Zealand *Kunzea* were inadequate for a taxonomic revision. This is a frequent world-wide problem for collections of widespread, common, often woody species, which for various reasons are usually under-represented in herbaria (Schmidt et al. 2005; Heenan and de Lange 2007).

Therefore, for this revision fresh herbarium specimens were collected from throughout the Australian and New Zealand range of *Kunzea ericoides* *s. l.* These specimens included samples of trunk and branchlet bark, seedlings, and adult foliage, along with juvenile, epicormic and reversion shoot foliage if present. From a wider sampling of 1000 specimens, preference for this paper was given to flowering and fruiting material, and material with new vegetative growth. For flowering material, flower diameters were recorded fresh, with all measurements taken in the field using Mitutoyo digimatic callipers. Also, for each of the taxa subsequently recognised, a number of sheets showing the progression from seedling to adult were made. Photographs of the growth habit of each gathering were also made, using an SLR Nikon FM 601 Camera and/or a Sony Cybershot 7.2 megapixel, and examples of these lodged with specimens at AK (herbarium accronyms follow Thiers (2014)).

The majority of the 1000 specimens collected for this revision were lodged at AK. Duplicates of these were preferentially lodged at AD because of the ongoing revision of the Australian members of the *Kunzea ericoides* complex being conducted there (H. R. Toelken pers. comm.). A further 280 live accessions representing the full range of New Zealand variation were grown under uniform conditions in the grounds of the Auckland City campus of the University of Auckland, New Zealand. In most cases plants were raised from seedlings sampled from wild populations deemed to be ‘pure’ (i.e. free from hybridisation). On occasion plants were also raised from seed or cuttings. For cuttings, semi-hardwood material sampled from the field was first struck by a commercial nursery and then grown on at the university. Because cuttings proved fickle and hard to strike, they were used only as a last resort for samples that were considered vital to this study, and for which seed and/or seedlings had already failed.

With the exception of flower measurements, the species descriptions and distributions are based on dry, wild-collected, herbarium vouchered material, with measurements made at comparable stages of growth. Specimens and type material were examined from the following herbaria: AD, AK, BM, CANB, CANU, CHR, F, FI, HO, K, MEL, MPN, NZFRI, NSW, OTA, P, UNITEC, WAIK, and WELT and the distribution of all specimens plotted. All specimens handled were annotated with identification labels, and in some situations accompanying notes and illustrations were attached to specimens to explain the decisions that were made. A selected list of ‘Representative Specimens’ is cited under each species entry, the specimens listed being a subset of the full range of specimens examined (for a full listing of these see Appendix 1 of de Lange 2007). Distributional records and maps are based on specimens seen and annotated by the author.

Scanning Electron Microscopy (SEM) was employed to examine the branchlet indumentum and seeds of selected *Kunzea* samples. Selected young branchlets from herbarium specimens and fresh material were removed by scalpel, and trimmed down to 5 mm lengths. These were placed separately in porous pots in a steel trough, flash frozen in liquid nitrogen, and the trough then sealed in a pressure chamber for critical point drying with liquid CO_2_ at 1200 psi and 34 °C. The samples were then mounted on adhesive discs on 25 mm diameter aluminium stubs and gold sputter-coated in a Polaron E5000 SEM coating unit. Seeds were sampled from herbarium specimens and, being already dry, were mounted directly on to adhesive discs on 25 mm diameter aluminium stubs, and gold sputter coated. Samples were then viewed in a Philips XL 30S FEG (Field Emission Gun) SEM at the School of Engineering, University of Auckland.

Images of the indumentum of all taxa, and seeds for all taxa except *Kunzea toelkenii* (for which seed was not available) were obtained. In most cases 20 or more seed samples were examined from a suite of specimens spanning the range of the species. No cultivated seed material was used. However, for two species, *Kunzea ericoides* and *Kunzea salterae*, only a few seeds could be obtained from herbarium material. Measurements for seeds were obtained from seed mounted on slides and then examined using a Zeiss Axioplan 2 fitted with a graticule, and/or images captured from the Zeiss Axioplan 2 on a Zeiss AxioCam HRc digital camera using Zeiss Axio Vision 3.0 software (Carl Zeiss Pty Ltd, Göttingen, Germany). Digital images were measured using Micro-Measure Version 3.3 (Reeves 2000). As a guide to seed terminology and descriptions, critical attention was paid to the *Kunzea* treatment in Webb and Simpson (2001).

Branchlet hairs were measured from scanning electron micrographs, or images were mounted on slides and examined using a Zeiss Axioplan 2 mounted with a graticule. All branchlet indumentum descriptions were supplemented by observations obtained using a binocular Leica Wild M3C light microscope at AK and calibrated by staff there.

Pollen measurements were obtained from a minimum of 20 fresh flowers randomly selected from 20 individuals for all species except *Kunzea triregensis* and *Kunzea salterae*, which are narrow-range endemics that had only limited material available. Pollen was careful tapped off the anthers on to slides. To ensure that the medium in which pollen was mounted did not affect the pollen sizes recorded, sufficient pollen was gathered to treat half of each sampling with cotton blue and the other with FLP orcein (Jackson 1973). Pollen slides were left to take up the stain for up two hours, then examined using a Zeiss Axioplan and images captured using Zeiss Axio Vision 3.0 software (Carl Zeiss Pty Ltd., Göttingen, Germany). Digital images were measured using Micro-Measure Version 3.3 (Reeves 2000).

Flowering and fruiting times were determined exclusively from herbarium specimens. Preference was given to herbarium data because many observations of flowering times have been based on cultivated plant behaviour (e.g., Harris 1996), or were based on unverifiable observations influenced by the timing of fieldwork (Given 1980a). Further, observations of the behaviour of cultivated accessions of the *Kunzea ericoides* complex grown for this study in uniform conditions at the University of Auckland for nine years, suggested that flowering times in some taxa can vary from year to year by up to three months. Admittedly herbarium specimens may have their own bias in that people tend to collect out of season flowering but at least the source is verifiable and the flowering times generated are less open to dispute. Full ranges are given for each species, adopting the format (Aug–)Sep–Oct(–Mar), to mean that flowers can be observed any time between August and March but that the peak flowering time is between September and October. Fruiting times are based on the presence of fruit whether dehisced or not, because my intention is to show the length of time that fruit may be found on the various *Kunzea* species, not the period between fruit development and seed dehiscence. Popular mythology is that New Zealand “*Kunzea ericoides*” can be distinguished from superifically similar, persistent fruited *Leptospermum scoparium* J.Forst. et G.Forst. (Myrtaceae) by its deciduous fruits which are said to be all shed by about March each year. While in part this reflects the usual timing of fruit maturation and seed release (see Burrows 1973), my own field collections and observations, supplemented by studies of cultivated plants used in revision, suggest that undehisced fruits may be retained for up to eight months in some species and that viable seeds may be released for up to ten months from fruit maturation.

## Characters

The *Kunzea* species descriptions mostly follow the terminology used by Briggs and Johnson (1979) for the Myrtaceae with modifications as suggested by H.R. Toleken (pers. comm.) who is actively revising the Australian species. Particular attention was paid to a range of characters as detailed below and noted in Table 1.

**Table 1.** Distinguishing features of New Zealand *Kunzea*.

	*Kunzea amathicola*	*Kunzea ericoides*	*Kunzea linearis*	*Kunzea triregensis*
**Habitat**	Coastal to lowland (sea level – 320 m a.s.l.). Primarily a species of mobile or stabilised sand country and associated coastal headlands. Also found around estuaries and extending up river valleys. Occasionally on offshore islands (Hauraki Gulf)	Coastal to low alpine (sea level – 1600 m a.s.l.). A primary coloniser of formerly forested habitats on a range of substrates including sand, clay, loams, alluvium, sedimentary, igneous, plutonic and ultramafic rock	Coastal to lowland (sea level – 310 m a.s.l.). Favouring stable sand, sand and clay podzols and the margins of peat bogs. Rarely extending into tall forest. Occasionally found in hill country as a component of successional vegetation. Also on offshore islands	Coastal (sea level – 296 m a.s.l.). In open ground, shrublands and as the dominant of tall forest
**Growth form**	Heterophyllous. Either rounded shrubs (up to 2 × 3 m) or erect to spreading trees (up to 18 × 8 m)	Homophyllous. Erect to pendulous trees up to 18 × 6 m	Homophyllous. Erect small trees up to 12 × 3 m	Homophyllous. Erect tall trees up to 18 × 3 m
**Trunk**	1(–2) usually branching from or near to base. Up to 0.85 m d.b.h. Erect, soon arching outwards. Juveniles much branched from base. Adults usually devoid of branches in lower half of trunk	1(–4). Usually devoid of branches in lower half of trunk. Up to 0.85 m d.b.h. Erect	1(–4 or more). Usually devoid of branches in lower half of trunk. Up to 0.85 m d.b.h. Mostly erect	1(–6). Devoid of branches in lower half of trunk. Up to 0.85 m d.b.h. Mostly erect
**Old bark**	Corky-coriaceous, tessellated, peeling upwards along trunk as broad, tabular strips with ± entire margins or weakly irregular. Secondary peeling not evident. Bark sparsely vegetated by liverwort and lichen growth	Corky-coriaceous, coarsely tessellated or broken in long elongate sections, peeling inwards along transverse and longitudinal cracks, remaining centrally attached. Flakes mostly tabular, peeling in chartaceous layers, with ± entire to sinuous margins. Secondary peeling common. Bark often bare but may be densely covered by moss, liverwort and lichen growth	Corky-coriaceous, coarsely tessellated, peeling inwards along transverse and longitudinal cracks, remaining centrally attached. Flakes mostly detaching in layers as chartaceous, lunate (in profile) flakes, margins often irregular with frayed apices. Secondary peeling not evident. Bark sparingly vegetated by liverwort and lichen growth	Corky-coriaceous, ± tessellated, peeling upwards along trunk as broad tabular strips, margins ± entire, surface often deeply corrugated and cracked. Secondary peeling not evident. Bark usually sparingly vegetated by moss, liverwort and lichen growth
**Epicormic growth**	Occasional	Not present	Not present	Not present
**Reversion shoots**	Common on damaged trunk and branch bases	Not present	Not present	Not present
**Suckers**	Absent	Absent	Absent	Absent
**Branches**	Juvenile branches erect to suberect not spreading. Adult branches initially suberect, soon arching and spreading, weakly flexuose. Reversion shoots common	Slender, initially ascending, soon spreading, apices usually pendulous. Reversion shoots absent	Ascending to upright, very rarely spreading, distinctly plumose. Reversion shoots absent	Upright to ± spreading. Reversion shoots absent
**Branchlet hairs**	Copious, persistent, antrorse-appressed, 225–500 μm long	Initially copious, soon glabrescent, divergent, 20–50 μm long	Usually copious (rarely glabrous), persistent, antrorse-appressed, 400–700 μm long	Copious, persistent, antrorse-appressed, 220–520 μm long
**Leaves**	Adult and juvenile leaves adaxially dark glossy green, abaxially paler. Juvenile leaves (2.4–)3.4(–5.3) × (1.2–)1.9(–2.3) mm, ovate, broadly ovate, rhomboid to obovate. Adult leaves (6.0–)8.2(–12.5) × (1.8–)2.6(–3.8) mm, oblong, oblong-obovate, broadly oblanceolate to broadly lanceolate	Bright green, yellow green, rarely dark green, (4.0–)13.5(–25.0) × (0.5–)1.1(–1.8) mm, linear, linear-lanceolate to narrowly lanceolate	Initially silvery-grey, maturing dark green to glaucous green, (9.3–)12.7(–19.5) × (0.3–)0.7(–1.2) mm, linear	Adaxially dark glossy green, abaxially paler, (6.0–)10.0(–13.5) × (1.1–)1.8(–2.3) mm, lanceolate to narrowly lanceolate
**Leaf margins and midrib**	Leaf margins and abaxial midrib densely covered in a thick (up to 0.6 mm wide), plumose band of white sericeous, antrorse-appressed hairs, converging at leaf apex in a distinct tuft of hairs. Surfaces glabrous to sparsely hairy	Leaf margins sparsely covered with antrorse-appressed hairs, tending to glabrate; abaxial midrib glabrate to glabrous. Hairs failing, short of leaf apex. Surfaces glabrous	Leaf margins and abaxial midrib densely covered in a thick (up to 0.4 mm wide), plumose band of antrorse-appressed hairs, usually converging just short of leaf apex. Surfaces sparsely hairy to glabrate, rarely glabrous	Leaf margins and abaxial midrib densely covered in a thick (up to 0.6 mm wide), plumose band of white sericeous, antrorse-appressed hairs, converging at leaf apex in a distinct tuft of hairs. Surfaces glabrous to sparsely hairy
**Flowering**	(Jul–)Nov–Jan(–Jun)	(Nov–)Dec–Jan(–Mar)	(Jul–)Nov–Jan(–May)	(Oct–)Dec(–May)
**Inflorescence**	Elongate, (5–)12(–20)-flowered botryum up to 200 mm long. Male flowers absent	Mostly a compact, corymbiform to shortly elongate, (3–)8(–15)-flowered botryum up to 60 mm long. Male flowers absent	Mostly a compact, spiciform (3–)8(–12)-flowered botryum up to 80 mm long. Male flowers absent	Elongate, (3–)10(–2)-flowered botryum up to 200 mm long, often interrupted by lengths of vegetative growth, sometimes bearing additional lateral elongate botrya. Male flowers absent
**Pherophylls**	Persistent, foliose, spreading, strongly recurved; pherophylls of juvenile plants (2.0–)3.4(–5.3) × (1.2–)1.9(–2.3) mm; adult pherophylls (4.1–)5.4(–6.0) × (1.6–)2.3(–3.1) mm, oblong, oblong-obovate, broadly obovate to elliptic	± Persistent, foliose, spreading, (3.0–)6.7(–7.8) × (0.9–)1.1(–1.4) mm, narrowly elliptic, lanceolate to narrowly lanceolate	Persistent, foliose, ascending to suberect, rarely spreading, (6.0–)9.8(–12.8) × (0.9–)1.8(–2.2) mm, linear to linear-falcate	Persistent, foliose, spreading, strongly recurved, (6.0–)9.8(–12.8) × (0.9–)1.8(–2.2) mm, broadly lanceolate to lanceolate
**Hypanthium**	Broadly obconic, turbinate to hemispherical, (1.9–)2.8(–4.0) × (3.0–)4.0(–5.6) mm. Free portion 0.7–1.3 mm long	Sharply obconic, (1.4–)2.1(–3.2) × (1.9–)2.9(–4.1) mm. Free portion 0.4–1.0 mm long	Barrel-shaped, cupular or narrowly campanulate, (2.0–)2.8(–4.0) × (2.5–)3.4(–4.1) mm. Free portion 0.6–0.9 mm long	Hemispherical to broadly obconic, sometimes campanulate or cupular. Free portion 0.6–0.8 mm long
**Flower diameter**	(6.8–)11.6(–12.5) mm	(4.1–)6.3(–8.3) mm	(1.9–)3.9(5.7) mm	(6.3–)10.2(–12.3) mm
**Petals**	5(–8). White (often drying yellow). Orbicular to broadly ovate, spreading, (1.8–)2.6(3.7) × (0.6–)1.0(–1.8) mm. Oil glands colourless	5. White (often drying yellow). Orbicular, suborbicular to narrowly ovate, spreading, (1.4–)2.2(–2.6) × (1.5–)2.2(–2.9) mm. Oil glands ± colourless	5(–6). Cream, pale pink or cream basally flushed pink (drying white). Narrowly ovate to suborbicular, suberect, distal 30% often weakly recurved, (0.9–)1.4(–2.0) × (0.7–)1.4(–1.9) mm. Oil glands colourless	5(–6). White (drying white). Orbicular to broadly ovate, spreading, (1.3–)2.8(–4.3) × (1.9–)2.8(–4.8) mm. Oil glands colourless
**Anthers**	Ellipsoid, ovoid-ellipsoid to ovoid-scutiform, 0.40–0.60 × 0.20–0.35 mm. Anther connective gland present or absent. Deep golden-yellow to orange when fresh, drying orange to pink	Broadly ellipsoid, 0.35–0.48 × 0.16–0.24 mm. Anther connective gland prominent, pink or pinkish-orange when fresh, drying red-orange	Testiculate, 0.04–0.06 × 0.02–0.04 mm. Anther connective gland prominent, pale pink or golden yellow when fresh, drying yellow to pale orange	Testicular-ellipsoid, 0.05–0.10 × 0.06–0.08 mm. Anther connective gland pink or golden yellow when fresh, drying yellow to pale orange
**Pollen**	(9.9–)14.8(–18.9) μm	(14.1–)14.6(–17.3) μm	(13.2–)16.2(–21.0) μm	(12.0–)13.8(–16.0) μm
**Ovary**	5(–6) locular	(4–)5 locular	(3–)4(–5) locular	4(–5) locular
**Style and stigma**	Style 2.0–3.2 mm long at anthesis, white or pinkish-white. Stigma broadly capitate at least 50% wider than style or even wider, surface flat	Style 1.5–2.2 mm long at anthesis, white, flushing pink at anthesis. Stigma capitate, c.25% wider than style, surface flat	Style 0.8–2.0 mm long at anthesis, cream or pale pink. Stigma narrowly capitate as wide as or slightly wider than style, surface ± flat	Style 1.9–3.1 mm long at anthesis, white or pinkish white. Stigma broadly capitate much wider than style, surface ± flat
**Fruit**	Broadly obconic, turbinate to hemispherical, (2.4–)3.9(–4.8) × (3.6–)4.8(–6.0) mm. Long persistent	Cupular, barrel-shaped, shortly cylindrical to hemispherical, (1.9–)2.7(–3.4) × (1.8–)2.8(–3.9) mm. Rarely persistent	Barrel-shaped to narrowly obconic, (1.6–)2.3(–2.9) × (2.3–)3.0(–4.1) mm. Long persistent	Hemispherical, broadly obconic, campanulate to cupular, (1.9–)3.2(–5.2) × (2.0–)3.1(–4.9) mm. Long persistent
**Seed**	Orange-brown to dark brown, oblong, oblong-obovate, narrowly ellipsoid to cylindrical, 1.2–1.5(1.7) × 0.3–0.4(–0.6) mm. Surface coarsely reticulate	Orange-brown to dark brown, obovoid, oblong, oblong-ellipsoid, or cylindrical and ± curved, 0.8(-1.0) × 0.32(–0.50) mm. Surface coarsely reticulate	Orange-brown to dark brown, obovoid, oblong, oblong-ellipsoid, or cylindrical and ± curved, 0.5–1.0(–1.1) × 0.48–0.63(–0.70) mm. Surface coarsely reticulate	Orange-brown to dark brown, oblong, oblong-obovate, 0.50–1.00(–1.10) × 0.50–0.60(–0.80) mm. Surface coarsely reticulate
**Chromosome karyotype**	10 chromosomes pairs, 2–2.5 μm long, one pair 1.5 μm long	10 chromosome pairs, 1.8–2 μm long, one pair 0.6 μm long	Eight chromosome pairs 1.2–1.5 μm long, three pairs 0.8–0.9 μm long	10 chromosomes pairs, 2–2.5 μm long, one pair 1.5 μm long

	*Kunzea robusta*	*Kunzea salterae*	*Kunzea serotina*	*Kunzea sinclairii*
**Habitat**	Coastal to montane (rarely subalpine) (sea level – 1000 m a.s.l.). An important component of successional shrubland and forest. Also found in mature forest on slip scars, around tree falls and rarely as a canopy constituent. Colonising a wide variety of substrates but preferring well drained clays, loams and alluvium or hard rock. Usually avoiding mobile sand systems	Coastal (sea level – 220 m a.s.l.). On mobile sand dunes, active and quiescent geothermal fields, associated clay, and hard rock as well as stable sand soils. Dominant on sand dunes and dominant to co-dominant of successional forest	Inland in low-lying areas to alpine situations (30 – 2000 m a.s.l.). In lowland areas favouring seasonally frost-prone situations. Inland locally common in intermontane basins, on steep mountain slopes, in frost-flats, tussock grasslands and in subalpine shrublands. Common on a range of skeletal soils, in flood prone soils, on fresh alluvium, and hard rock	Lowland to montane (20 – 510 m a.s.l.). Mostly confined to sparsely vegetated rhyolite rock tors and associated talus. Extending down stream and river gorges on rhyolite, and into open ground and scrub. Sometimes along roadsides in tall forest
**Growth Habit**	Heterophyllous. Erect, spreading trees up to 30 × 8 m	Homophyllous. Shrubs (0.1 × 2 m) or small trees (up to 10 × 6 m)	Heterophyllous. Shrubs (up to 2 × 2 m) or trees (up to 20 × 4 m)	Heterophyllous. Shrubs (up to 3 × 1 m). Rarely small trees (up to 6 × 4 m)
**Trunk**	1(–6). Mostly solitary. Up to 1 m d.b.h. Erect. Adults usually devoid of branches for at least the lower 1–3 m	Usually multi-trunked from base. In exposed conditions branched from base, otherwise mostly devoid of branches in lower half. Up to 0.3 m d.b.h. Widely spreading to suberect, flexuose	1(–3) arising from ground, basally buttressed. Except in tall shrublands branched from base. Up to 0.86 m a.b.h. Erect	1(–4) or more. Shortly erect, mostly branching at 0.2–1 m from base, sometimes indistinguishable due to branches arising from ground level
**Bark**	Corky-coriaceous, stringy to coarsely tessellated, peeling upwards in broad, tabular strips, margins ± entire to weakly irregular. Secondary peeling uncommon. Bark mostly bare, sometimes supporting sparse moss, liverwort and lichen growth	Corky-chartaceous, coarsely tessellated, peeling inwards along transverse and longitudinal cracks, remaining centrally attached. Flakes mostly narrowly and shortly tabular, often lunate (in profile). Secondary peeling uncommon. Bark devoid of moss, liverwort and lichen growth	Chartaceous to corky-chartaceous, somewhat stringy, readily peeling inwards along transverse and longitudinal creaks, often inrolled. Flakes hanging in loose inrolled masses, ± tabular, with deeply sinuous, to highly irregular margins, often deeply cracked, frayed, and crumpled. Secondary peeling common. Bark usually supporting dense moss, liverwort and lichen growth	Corky-coriaceous to somewhat chartaceous, coarsely stringy to tessellated, firmly attached, peeling inwards along transverse and longitudinal cracks, remaining centrally attached. Flakes ± tabular with entire margins and coarsely frayed apices. Secondary peeling common. Bark mostly bare, sometimes supporting sparse moss, liverwort and lichen growth
**Epicormic growth**	Not present	Not present	Occasional	Not present
**Reversion shoots**	Not present	Not present	Occasional	Not present
**Suckers**	Absent	Absent	Absent	Absent
**Branches**	Initially erect, soon arching outwards and spreading, distal ends mostly erect, rarely pendulous	Suberect to widely spreading, rarely ascending, mostly pendulous	Obliquely ascending, fastigiate	Prostrate and widely spreading, new growth subscandent
**Branchlet hairs**	Copious, persistent, mostly long (150–380 μm) to short (50–150 μm) antrorse-appressed; from East Cape to near Mahia Peninsula in mixtures of sparse long (100–200 μm), antrorse-appressed and abundant short (25–80 μm), divergent hairs	Initially copious, rarely glabrate to glabrous; hairs initially mixed, at first dominated by long (up to 550 μm) antrorse-appressed hairs, these deciduous, leaving behind persistent, mostly divergent, short (40–100 μm) hairs with ± curled apices	Copious, persistent, divergent, 50–80 μm long, apices weakly curled	Copious, persistent, antrorse-appressed, 280–600 μm long
**Leaves**	Adaxially light to dark green, abaxially paler. Juvenile leaves of mainly northern New Zealand and coastal locations, (14.6–)19.0(–28.4) × (1.6–)2.2(–2.5) mm; from the Rangitikei, central and northern Wairarapa and Mt Egmont, (3.2–)4.6(–6.3) × (0.7–)1.2(–1.5) mm. Adult leaves of northern New Zealand and coastal locations, (4.9–)14.2(–20.1) × (0.9–)1.7(–3.0) mm; from inland areas especially the Rangitikei, Wairarapa and Central Otago, (5.8–)9.3(–12.3) × (1.2–)1.8(–2.2) mm. Adult leaves oblanceolate, broadly oblanceolate, lanceolate to linear-lanceolate, rarely elliptic to obovate. Surfaces glabrous	Bright glossy green, yellow-green, bronze-green to dark green, (4–)10(–18) × (0.6–)1.2(–2.0) mm, linear-lanceolate to narrowly oblanceolate. Surfaces glabrous	Juvenile, sub-adult and reversion shoot leaves red-green, pale green suffused with red, or bright green, (0.8–)5.2(–7.8) × (0.6–)0.8(–1.2) mm, linear-lanceolate to lanceolate. Surfaces glabrous. Adult leaves dark glossy green or bronze-green, margins and base often flushed red, (2.0–)3.7(–6.3) × (0.8–)1.1(1.8) mm, linear-oblanceolate, oblanceolate to obovate. Surfaces glabrous	Juvenile leaves dark green or glaucous, up to 25.0 × 3.5 mm, oblanceolate to lanceolate, glabrous. Adult leaves silvery-white, silvery-grey to reddish-grey, (5.6–)14.5(–20.6) × (2.0–)3.2(–4.5) mm, broadly lanceolate, elliptic, obovate to oblong-obovate. Surfaces densely hairy
**Leaf margins and midrib**	Leaf margins initially finely covered with a thin often interrupted band of flexuose, spreading to antrorse-appressed hairs not or rarely meeting at apex, glabrescent; adaxial and abaxial midribs glabrate, basally clad with, deciduous, fine, antrorse-appressed hairs	Leaf margins sparsely to densely covered with antrorse-appressed hairs; abaxial midrib usually glabrous, sometimes with a dense weft of antrorse-appressed hairs near base. Hairs failing short of leaf apex	Leaf margins sparsely hairy, hairs antrorse to subantrorse, aligned in 1 or 2 often interrupted rows failing well short of leaf apex. Adaxial and abaxial midribs glabrescent, sometimes hairy near bases	Leaf margins and midribs of adult leaves distinctly hairy (though much less so than rest of lamina), hairs converging at leaf apex
**Flowering**	(Aug–)Nov–Jan–Feb(–Jun)	Aug–Apr	(Nov–)Jan–Feb(–May)	(Sep–)Nov–Jan(–Mar)
**Inflorescence**	Initially corymbiform often becoming shortly elongate, (1–)12(–30)-flowered, up to 60 mm long, sometimes with late season elongate botrya up to 80 mm long. Male flowers absent	Corymbiform, (2–)4(–8)-flowered, up to 45 mm long. Male flowers absent	Compact, corymbiform, (1–3–)8(–12)-flowered up to 25 mm long. Inflorescences on ultimate branchlet terminus often elongate with active, terminal vegetative growth. Male flowers absent	Mostly compact, corymbiform (4–)9(–20)-flowered, up to 20 mm long, usually terminated by active vegetative growth; sometimes extending as late season elongate botrya. Male flowers absent
**Pherophylls**	Deciduous or persistent, squamiform or foliose; squamiform clasping pedicels, foliose spreading. Squamiform pherophylls 0.4–1.2 × 0.3–0.6 mm, broadly to narrowly deltoid or lanceolate; foliose 6.0–)9.0(–17.9) × (1.1–)1.2(–1.8) mm, elliptic, oblanceolate, broadly lanceolate to lanceolate, flat or weakly recurved	Deciduous, mostly squamiform (rarely foliose), spreading, 0.6–1.8 mm long, broadly to narrowly linear lanceolate	Deciduous, mostly foliose (rarely squamiform), clasping pedicels, 0.9–2.5 mm long, spathulate, spathulate-orbicular, rarely pandurate or lanceolate	Deciduous, foliose or squamiform; foliose tightly clasping pedicel, (1.0–)1.2 × (0.2–)0.4 mm, oblong to oblong-lanceolate, very rarely broadly spathulate. Squamiform pherophylls tightly clasping pedicels, 0.3–1.0 × 0.4–0.8 mm, broadly to narrowly ovate or lanceolate
**Hypanthium**	Broadly obconic to turbinate, rarely cupular, (2.1–)3.1(–4.1) × (3.0–)3.9(–5.2) mm. Free portion 0.4–0.9 mm long	Narrowly obconic to funnelform, (2.1–)2.2(–3.8) × (1.8–)2.2(–3.2) mm. Free portion 1.0–1.6 mm long	Urceolate to campanulate, (1.6–)2.0(–3.4) × (1.5–)1.9(–3.8) mm. Free portion 0.4–0.8 mm long	Narrowly obconic to obconic or cupular, (1.9–)2.6(–3.6) × (2.1–)3.1(–4.2) mm. Free portion 0.4–0.7 mm long
**Flower diameter**	(4.3–)7.7(–12.0) mm	(9–)10(–12) mm	(2.8–)5.2(8.8) mm	(5.7–)8.1(–10.2) mm
**Petals**	5(–6). White, rarely pink (sometimes drying yellow or cream), orbicular, suborbicular to ovate, spreading, (1.5–)2.6(–3.8) × (1.3–)2.6(–3.6) mm. Oil glands colourless, drying opaque or grey	5. White, rarely basally flushed pink, orbicular to suborbicular, spreading, 1.4–1.6 × 1.4–1.6 mm. Oil glands not evident when fresh, drying colourless or rose-pink	5(–6). White, sometimes basally flushed pink, narrowly orbicular to broadly ovate or cuneate, 1.4–1.6(–2.0) × 1.2–1.6(–2.0). Oil glands yellow, drying pale yellow to ± colourless	5(–6). White, rarely basally flushed pink, broadly ovate, suborbicular to orbicular, rarely ± cuneate-truncate, spreading upper 30% ften weakly recurved, (2.0–)2.9(–3.6) × (2.1–)2.7(–3.3) mm. Oil glands not evident in fresh or dried material
**Anthers**	Ellipsoid to ovoid-ellipsoid or deltoid, 0.38–0.63 mm. Anther connective gland prominent, light pink, salmon pink, yellow to orange when fresh, drying dark orange, orange-brown or dark brown	Scutiform to ovoid, 0.11–0.16 × 0.10–0.14 mm, each anther deeply and longitudinally furrowed, with one anther lobe in each pair fused at right angles along inner margin with adjoining anther lobe to form a prominent “pinched” longitudinal ridge. Anther connective gland, pale orange to pink when fresh, drying orange-brown	Testiculate to ellipsoid, 0.04–0.06 × 0.02–0.04 mm. Anther connective gland, orange flushed with rose when fresh, drying dark orange-brown or purple	Broadly ellipsoid to scutiform, 0.06–0.1 × 0.06–0.09 mm. Anther connective gland, pale pink when fresh, drying pale orange
**Pollen**	(9.1–)14.7(–15.1) μm	(10.2–)14.7(–16.6) μm	(11.1–)12.4(–13.7) μm	(11.9–)15.4(–19.9) μm
**Ovary**	5(–6) locular	(3–)4 locular	3–4(–5) locular	(3–)4(–5) locular
**Style and stigma**	Style 2.0–3.5 mm long at anthesis, white or pinkish white; stigma broadly capitate, at least 1.5× style diameter of even wider, flat	Style 2.1–3.2 mm long at anthesis, white basally flushed with pink; stigma capitate, at least 1× style diameter, flat, abruptly broadened	Style 0.6–1.2 mm long at anthesis, white; stigma capitate, scarcely wider than style, usually flat or weakly domed along margins and centrally depressed	Style 1.8–3.0 mm long at anthesis, white basally flushed pink or pale pink; stigma narrowly capitate, as wide or scarcely wider than style, ± flat
**Fruit**	Obconic, broadly obconic to ± turbinate, rarely cupular, (2.2–)3.8(–4.6) × (3.2–)4.0(–5.3) mm. Rarely persistent	Cupular to suburceolate (2.0–)2.2(–2.7) × (2.0–)2.9(–4.0) mm. Rarely persistent	Urceolate to shortly campanulate, rarely cupular, (1.2–)2.1(–3.0) × (1.2–)2.1(–3.4) mm. Rarely persistent	Narrowly obconic to obconic, rarely cupular, (2.2–)3.0(–3.6) × (2.7–)3.2(–3.9) mm. Long persistent
**Seed**	Orange-brown to dark brown, oblong, oblong-obovate, oblong-elliptic, 0.9–1.0(–1.1) × 0.35–0.40(–0.48) mm. Surface coarsely reticulate	Orange-brown, narrowly oblong, oblong, oblong-obovate to falcate-oblong or elliptic, 0.80–1.00 × 0.45–0.48 mm. Surface coarsely reticulate, ridges prominent, central portion of each cell bearing a short, deciduous, tubular-spiny, protuberance	Orange-brown to dark brown, narrowly oblong, oblong, oblong-obovate, 0.60–0.90(–1.00) × 0.48–0.50(–0.60) mm. Surface coarsely reticulate	Orange-brown to dark brown, obovoid, oblong, or oblong-ellipsoid, 0.52–1.04(–1.09) × 0.38–0.58(–0.72) mm. Surface coarsely reticulate
**Chromosome karyotype**	Four chromosome pairs 2–2.5 μm long, six intermediate pairs 1.5–1.8 μm long, and one small pair 0.6 μm long	11 chromosome pairs, 0.9–1 μm long	11 chromosome pairs, 0.9–1 μm long	11 chromosome pairs, 0.9–1 μm long

	*Kunzea tenuicaulis*	*Kunzea toelkenii*
**Habitat**	Lowland to montane (40 – 580 m a.s.l.). Confined to sites of geothermal activity where it is often the dominant woody species	Coastal (< 20 m a.s.l.). Confined to mobile and semi-stable sand dunes
**Growth Habit**	Heterophyllous. Shrubs (up to 3 × 1 m) or small trees (up to 6 × 4 m)	Homophyllous. Shrubs (up to 4 × 6 m)
**Trunk**	(1–)4–6, in arborescent forms multi-trunked from base. Up to 0.6 m d.b.h. At first erect, soon widely spreading and curving to somewhat sinuous invariably soon branched; in decumbent plants trunk virtually indistinguishable, 0.01–0.10 m d.b.h., trailing to semi-erect, curved and somewhat sinuous, obscured by numerous branches	(1–)6(–10), up to 0.4 m d.b.h. Mostly arising from the top of a broad, serpentine rootstock, also appearing from exposed sections of root flange. Ascending to suberect, serpentine, highly contorted, twisted, bent, and spiralled. Lower half of trunk usually devoid of branches
**Bark**	Chartaceous to ± corky, tessellated, peeling upwards in small, thin, narrow mostly elongated flakes, these easily detached, margins mostly tabular to slightly sinuous or irregular. Secondary peeling not evident. Bark mostly bare. Rarely supporting sparse moss, liverwort and lichen growth	Corky-coriaceous, stringy, deeply furrowed, initially peeling inwards along transverse and longitudinal cracks and then upwards in long, thick, highly irregular, deeply sinuate, cracked and frayed flakes, often remaining central attached, and then lunate in profile. Flakes easily detached. Secondary peeling common peels lunate in profile. Bark usually supporting dense lichen growth
**Epicormic growth**	Occasional. Arising from basal portion of trunk only when damaged	Common. Arising from basal portion of damaged or undamaged trunk
**Suckers**	Absent	Commonly present
**Reversion shoots**	Occasional	Not present
**Branches**	Slender, often weakly flexuose; in prostrate plants trailing, otherwise initially ascending, soon suberect to widely spreading, arching, often pendulous	Widely spreading, ± serpentine, flexuose, often pendulous, usually interwoven with adjoining branches
**Branchlet hairs**	Copious, persistent, divergent, weakly flexuose, 25–78 μm long, apices ± straight	Copious, persistent, of two types; antrorse-appressed, up to 260 μm long, weakly flexuose, and divergent, 40–180 μm long, with apices twisted or spiralled
**Leaves**	Mostly dark glossy green, red-green to bronze-green, sometimes bright green, spreading to recurved. Juvenile leaves 0.9–3.0(–4.5) × 0.2–0.4(–0.6) mm, linear-lanceolate, persistent in stressed habitats, or as reversion shoots. Adult leaves (1.1–)4.0(–10.0) × (0.8–)1.3(–2.8) mm, narrowly oblanceolate, oblanceolate, obovate to obovate-rostrate. Surfaces glabrous, rarely abaxial surface with fine hair covering toward leaf base	Dark glossy green or bright-green, spreading to weakly to strongly recurved, (2.6–)5.7(–8.5) × (0.6–)1.6(–2.5) mm, obovate, clavate, to broadly oblanceolate. Surfaces glabrous
**Leaf margins and midrib**	Margins sparsely to densely covered with deciduous, antrorse-appressed to subantrorse, weakly spreading hairs failing just short of cuspidate leaf apex. Adaxial and abaxial midribs sparsely covered in deciduous, antrorse-appressed hairs, these increasing in density toward base, not reaching to leaf apex	Margins sparse to densely covered with ± persistent, antrorse, subantrorse to spreading hairs meeting just short of leaf apex. Lower half of adaxial midrib finely covered in deciduous, antrorse-appressed hairs, abaxial glabrous
**Flowering**	(Aug)Sep–Oct(–Mar)	(Sep–)Oct–Nov
**Inflorescence**	Mostly compact, corymbiform (1–)6(–10)-flowered botrya up to 25 mm long, rarely with inflorescence at the ultimate branchlet tips elongated; these elongate botrya always surmounted by active terminal vegetative growth. Male flowers not seen	Mostly compact, corymbiform (1–3–)7(–10)-flowered botrya, up to 40 mm long. Inflorescences at the ultimate branchlet terminus uncommon (except in trailing epicormic growth), if present then up to 80 mm long, bearing active terminal growth. Flowers of late season elongate botrya often functionally male
**Pherophylls**	Deciduous, initial few foliose rest squamiform, tightly clasping pedicels to ± spreading, 0.5–1 mm long, foliose pherophylls pale green, oblong, oblong-obovate to oblanceolate; squamiform pherophylls brown or pink, deltoid to oblong-ovate	Deciduous, initial few foliose rest squamiform, tightly clasping pedicel or spreading, 0.4–1.6 mm long; foliose pherophylls green to bronze-green, shortly lanceolate to obovate; squamiform pherophylls amber-brown to brown, deltoid to ovate
**Hypanthium**	Narrowly cupular to campanulate, (1.8–)2.5(–3.3) × (1.7–)2.4(–3.1) mm. Free portion 0.3–1.0 mm long	Obconic to funnelform, (1.7–)2.4(–3.2) × (2.8–)3.6(–4.3) mm, with free portion 0.6–0.9 mm long
**Flower diameter**	(3.3–)5.5(–9.0) mm	(3.6–)6.8(–9.0) mm
**Petals**	5(–6). White, pinkish white, usually basally flushed pink, sometimes completely pink, orbicular, sometimes cuneate, 1.4–1.6(–2.0) × 1.4–1.6(–2.0) mm. Oil glands not evident when fresh, drying colourless	5(–6). White, orbicular to very broadly ovate, 1.5–1.9(–2.8) × 1.5–1.9(–2.6) mm. Oil glands colourless in fresh and dried material
**Anthers**	Testiculate, 0.04–0.08 × 0.02–0.04 mm. Anther connective gland orange when fresh, drying pale brown	Testicular-oval to testicular-ellipsoid, 0.06–0.09 × 0.05–0.08 mm. Anther connective gland pale lemon to pink when fresh, drying yellow to pale orange
**Pollen**	(12.8–)14.7(–16.6) μm	(12.2–)13.6(–17.8) μm
**Ovary**	(3–)4(–5) locular	3–4(–5) locular
**Style and stigma**	Style 2.0–3.6 mm long at anthesis, white basally flushed with pink; stigma capitate, scarcely wider than style. Domed along margins with a basal central depression	Style 1.0–1.8 mm long at anthesis, white; stigma capitate, scarcely wider than style, flat
**Fruit**	Usually barrel-shaped, rarely cupular, (1.0–)2.3(–3.3) × (1.6–)2.2(–3.2) mm. ± Persistent	Broadly obconic to cupular, (2.1–)2.6(–3.0) × (2.5–)3.0(–3.7) mm. Rarely persistent
**Seed**	Orange-brown to dark brown, narrowly oblong, oblong, oblong-obovate to falcate-oblong, 0.80–1.00 × 0.45–0.50 mm. Surface coarsely reticulate	Amber, orange-brown to brown, oblong, oblong-obovate 0.50–1.02 × 0.52–0.68 mm. Surface coarsely reticulate
**Chromosome karyotype**	11 chromosome pairs, 0.9–1 μm long	11 chromosome pairs, 0.9–1 μm long

### Growth habit

Past treatments of New Zealand *Kunzea* have not paid much attention to the growth habit of the taxa then recognised. For the purposes of this revision the growth-habit terminology adopted by Hickey and King (2000) has been used.

The majority of the New Zealand species are normally arborescent, and the form of the adult tree in these species is often diagnostic (see Table 1). Accordingly, *Kunzea sinclairii* is easily distinguished from all of the arborescent species by its normally decumbent, widely spreading, semi-scandent growth habit. Three species, *Kunzea salterae*, *Kunzea tenuicaulis* and *Kunzea toelkenii*, inhabiting active geothermal, sand dune and insular coastal habitats, may be shrubs or small trees. Two of the three species that may grow on ultramafic substrates, *Kunzea ericoides* and *Kunzea linearis*, often adopt a completely decumbent and long trailing growth habit on this geology. However, in cultivation all transplants, cutting-grown specimens and seedlings germinated from decumbent, ultramafic ‘races’ of *Kunzea ericoides* and *Kunzea linearis* and grown in a potting mix comprising equal portions of pine bark, peat, and sand soon developed the erect growth habit usual for these species elsewhere. This also proved the case with seed germinated from wild-collected decumbent forms of the Three Kings Island group endemic, *Kunzea triregensis*. One species, *Kunzea tenuicaulis* exhibits a greater diversity of growth habit than any other New Zealand species with plants ranging from decumbent shrubs through to widely spreading, multi-trunked mostly pendulous branched trees. Unlike the majority of the other shrub forms seen in New Zealand *Kunzea*, some of the variants of *Kunzea tenuicaulis* appear to have a genetic basis. Therefore, while the majority of cultivated decumbent plants sampled from near active geothermal vents soon developed the multi-trunked, widely spreading tree habit seen in specimens growing away from these more physiologically stressed habitats; a minority retained the decumbent growth habit. Notably, such plants tended to retain their juvenile foliage despite flowering.

In all species the nature of the trunk and the attitude of the branches provide additional distinctions. One species, *Kunzea serotina*, has a mostly pyramidal growth habit, with distinctly obliquely ascending branches and fastigiate branchlets. This growth habit is only occasionally lost in very old trees, where only the crown branches are left. *Kunzea linearis* can be recognised by its erect, plumose branchlets, caused in part by the subappressed leaves, which are densely crowded toward the branchlet tips. While several species tend to have single trunks, *Kunzea salterae*, *Kunzea sinclairii*, and *Kunzea tenuicaulis* typically have multiple trunks, which is also the usual condition in *Kunzea toelkenii*.

One peculiarity of New Zealand *Kunzea* is the tendency to see reversals in growth habit. Thus, while *Kunzea sinclairii* is usually a decumbent, widely spreading shrub, very occasionally it can form a tree up to 6 m tall. In such examples, however, the subscandent and widely spreading branchlets typical of this species are retained. Similarly *Kunzea robusta*, the tallest species in the genus, while mostly arborescent with suberect, widely spreading branches, may occasionally develop a pendulous growth habit, with branches that can touch the ground and trail for some distance from the tree. Much less frequently *Kunzea robusta* can develop a low, compact shrub habit. In both these cases there is sometimes a genetic basis for these forms, with at least some plants with a pendulous and/or decumbent habit sterile aneuploids (2*n* = 23; de Lange and Murray 2004).

Heterophylly is also common in the New Zealand species, with only *Kunzea ericoides*, *Kunzea linearis* and *Kunzea salterae* lacking a distinct juvenile form. In some species, such as the sand country inhabiting *Kunzea amathicola*, the juvenile form is often persistent, particularly so in stressed habitats where it often flowers and fruits. The same condition is also seen in *Kunzea tenuicaulis* plants growing near active fumaroles. Several species exhibit epicormic growth when damaged but in one species, *Kunzea toelkenii*, epicormic growth is produced irrespective of whether the trunk is damaged, and so is especially diagnostic of this species. In this species the trunk bases of mature shrubs are usually surrounded at ground level by a dense, encircling mass of completely decumbent, long trailing, flowering and fruiting epicormic growth. Reversion shoots of juvenile foliage are unusual in the New Zealand species. They are known only from *Kunzea amathicola*, *Kunzea tenuicaulis*, and, more rarely, *Kunzea serotina*. Reversion shoots are most commonly seen near the branch bases.

### Bark

The bark of New Zealand *Kunzea* offers some useful characters for field identification and examples of these are illustrated under each species treatment below. Bark from the trunk is preferred for this revision. This is because the ‘early bark’ produced by branchlets scarcely differs between most species. I refer to the large flakes or strips of decorticated bark frequently found festooning the trunks of mature trees as the ‘primary bark’ and the smaller flakes and shards which may peel from the upper surface of the primary bark, I refer to as the ‘secondary bark’. The bark types generally follow the terminology used to describe *Eucalyptus* L’Hér. bark by Brooker and Kleinig (1983) and in their subsequent publications. Under their system and with some modification *Kunzea* bark can be divided into four main types:

1. Primary bark corky-coriaceous, tessellated to stringy, peeling up the trunk in long (0.8–8.0 m), ± tabular strips, with little or no secondary peeling, the strips mostly not breaking easily in half, and usually leaving a ‘clean’ ± regular margin when snapped. Bark of this type does not crumble easily in the hand, and is typical of *Kunzea amathicola*, *Kunzea triregensis*, and *Kunzea robusta*.
2. Primary bark mostly corky-coriaceous, sometimes chartaceous, initially tessellated, ± stringy, either remaining firmly attached at the middle and peeling from the ends le, in small, ± regular to highly irregular flakes (up to 0.1 m long), leaving the flakes centrally attached such that the flakes present as ‘lunate peels’ when viewed from the side. This bark type is usually readily broken, and snaps with either a ‘clean’ ± regular margin or one that is highly irregular. The bark flakes are also distinctive in that they often crumble readily in the hand. This bark type is typical of *Kunzea linearis* and *Kunzea toelkenii*.
3. Primary bark mostly corky-coriaceous or chartaceous, initially tessellated or broken in long elongate sections; peeling from the margins inwards and remaining centrally attached or peeling from the base upwards, in either case forming small to large (up to 0.6 m long) ± tabular strips or smaller flakes with a ‘clean’ ± regular or slightly irregular to sinuous margin. The primary bark is usually moderately free of extensive secondary peeling. If secondary peels are absent, the primary bark is often deeply cracked and furrowed. This bark type is readily broken, and snaps with either a ± regular margin or one that is highly irregular. The bark flakes are also distinctive in that they often crumble readily in the hand. This bark type is typical of *Kunzea ericoides*, *Kunzea salterae*, *Kunzea sinclairii*, and *Kunzea tenuicaulis*.
4. Primary bark mostly chartaceous to corky-chartaceous, stringy, readily peeling inwards along margins, usually inrolled (like wood shavings), often left hanging semi-attached by the middle or apex of the bark, in loose masses of unevenly ± tabular or not, deeply cracked, frayed and crumpled masses. Bark margins usually highly irregular, and mostly deeply sinuous. Secondary peeling is common. Bark of this type is often covered by dense bryophyte and lichen growth. The flakes often form dense piles of ‘wood shavings’ at the trunk base. This bark type readily detaches from the trunk, and crumbles freely in the hand. If snapped when dry it usually shatters into a mass of variable sized pieces. If wet it snaps less readily, characteristically with a highly irregular frayed margin. This bark type is unique to *Kunzea serotina*.

These bark types are potentially highly diagnostic and are exhibited in cultivated plants grown in uniform conditions. However, they can vary within species and seem to be partially influenced by growing conditions in the wild. Thus specimens of *Kunzea robusta* growing in shaded or damp situations can have less coriaceous more chartaceous bark, which may at times peel in a lunate fashion to resemble *Kunzea linearis*. *Kunzea ericoides*, particularly at higher altitudes, may have bark approaching that seen in *Kunzea serotina*, and in very dry habitats its bark can resemble *Kunzea robusta* in that it lacks secondary peeling. For this reason, although bark is described carefully for each species, it is not used to key them out, and should not be used as the sole means for identifying species. Further, it is important to note the type of bark in some detail before pressing, as characters can be lost on drying. Ideally bark should be photographed *in situ* before collecting, or stored unpressed in paper bags, to be mounted later with the rest of the herbarium specimen once it has dried.

### Branchlet hairs

The utility of branchlet hairs as an aid toward species delimitation was recognised by Thompson (1989) in her revision of the allied Myrtaceous genus *Leptospermum* J.R.Forst et G.Forst. Branchlet hairs were found to be invaluable in segregating taxa and also in determining putative wild hybrids throughout this study. For hairs, the terminology of Hewson (1988) was adopted because her treatment is exhaustive, and supplemented by excellent, unambiguous line drawings of the different hair types described. Two main hair types were distinguished: 1. divergent and 2. antrorse-appressed (short or long). It is essential that young emergent growth is used to view these, because only then can the branchlet hairs be easily seen, unfettered by the usually longer, flexuose, spreading or antrorse-appressed hairs that typically emanate from the decurrent leaf bases of all the species except *Kunzea ericoides*. In that species, uniquely, antrorse-appressed hairs are completely absent. Hairs should ideally be viewed from the third leaf back from the branchlet tip because some species, e.g., *Kunzea salterae*, may have occasional, usually deciduous antrorse-appressed hairs close to the emergent branchlet tip. The type of hair present can be easily determined using a standard 10× field lens in most cases. However, a higher magnification, such as 20× is useful for *Kunzea ericoides* because the hairs of that species are the smallest of all the New Zealand *Kunzea* and so they may not be seen with lower magnification. Although branchlet hairs are a critical part of this revision, it is important to recognise that in zones of hybridism, hair types may lose their utility, especially where two species with antrorse-appressed hairs meet. However, in situations where taxa with divergent or antrorse-appressed hairs meet, hairs can be a useful first step toward hybrid recognition. Nevertheless at least three species recognised here, *Kunzea robusta* (eastern North Island populations), *Kunzea salterae* and *Kunzea toelkenii* usually have mixtures of predominantly divergent and some antrorse-appressed hairs. For those species, recourse to other characters will be needed to ensure accurate identification.

### Vegetative buds

**Figure 1.**
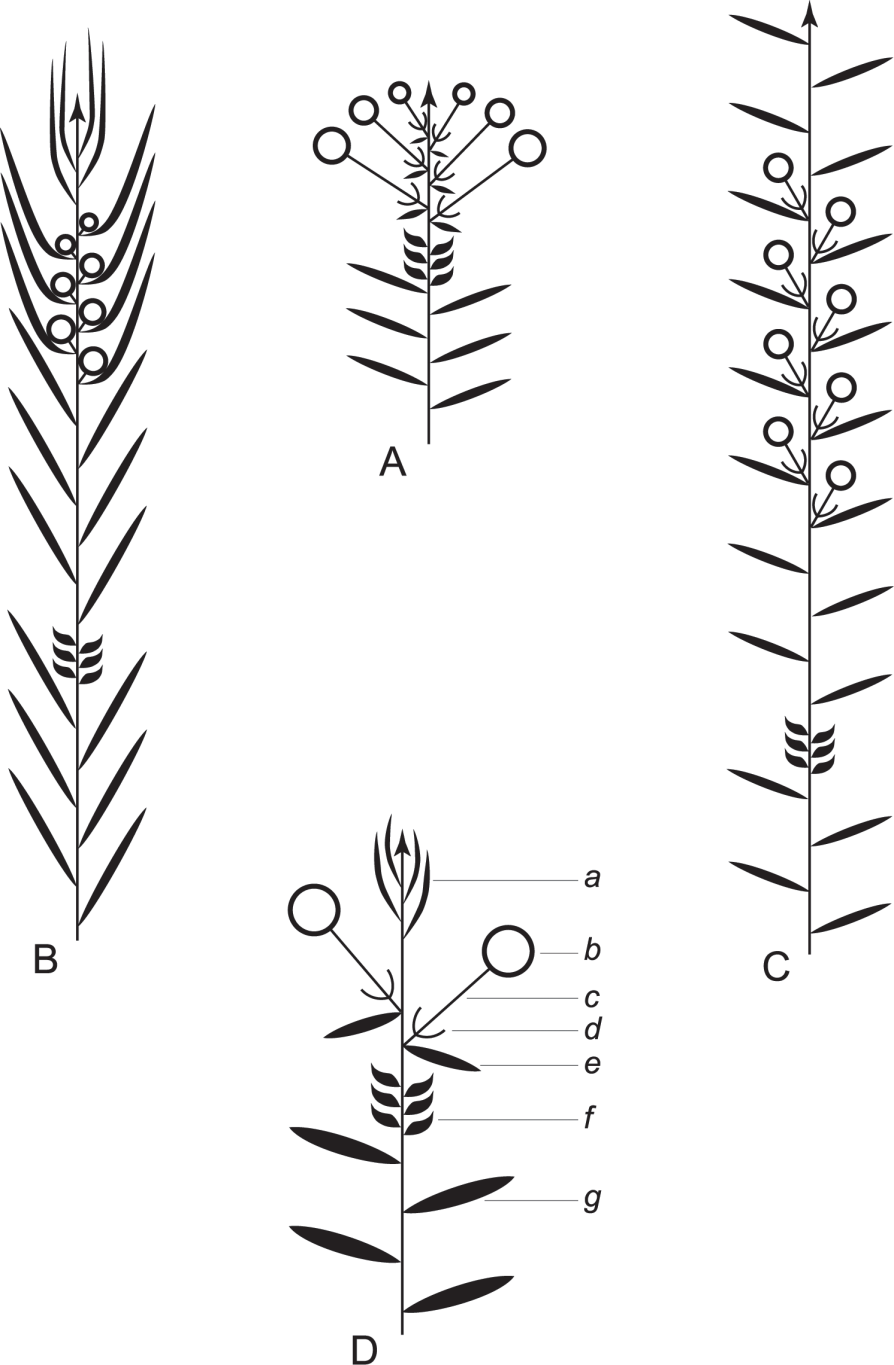
Schematic diagram of the inflorescences (i.e. conflorescences) of New Zealand members of the *Kunzea ericoides* complex. **A** ‘corymbiform’ (*Kunzea ericoides*, *Kunzea robusta*, *Kunzea salterae*, *Kunzea serotina*, *Kunzea sinclairii* (aggregated corymbiform), *Kunzea tenuicaulis*, *Kunzea toelkenii*) **B** ‘spiciform’ (*Kunzea linearis* only – to aid figure interpretation prophylls are not shown) **C** ‘elongate racemiform’ (*Kunzea amathicola*, *Kunzea triregensis* only) **D** inflorescence terminology: (**a**) active vegetative bud, (**b**) flower bud, (**c**) pedicel, (**d**) prophyll, (**e**) pherophyll, (**f**) perules, (**g**) leaves.

The vegetative buds are covered with scales that form a protective covering over the vegetative primordia (see perule). Although strictly speaking these scales should be called perules (see Briggs and Johnson 1979) I follow the suggestion of Toelken (1996; and *in litt.*) that perules should be reserved for the scales that cover the floral primordia (see Fig. 1). Vegetative bud scales offer little toward enabling species recognition in the New Zealand *Kunzea ericoides* complex, mainly because for most of the species treated here they show considerable overlap in size, shape, indumentum and other more cryptic characters. It should be noted that in some species, such as *Kunzea ericoides*, *Kunzea sinclairii*, *Kunzea triregensis* and *Kunzea linearis*, vegetative bud scales may be inconspicuous, in part because they are obscured by the surrounding leaves, but also because in these species more than any other the scales grade into foliose forms, such that they closely resemble and can be mistaken for diminutive leaves.

### Leaves

With few exceptions the lamina-shape of leaves of *Kunzea* are so variable that they afford few consistent characters to assist with accurate species recognition (Table 1). Nevertheless adult leaf size divides New Zealand *Kunzea* into three groups: 1. the ‘small-leaved’ species (*Kunzea serotina*, *Kunzea tenuicaulis*, and *Kunzea toelkenii*), 2. those with mostly linear leaves (*Kunzea ericoides*, *Kunzea linearis*, *Kunzea triregensis*, and *Kunzea salterae*), and 3. those with ‘large broad leaves’ (*Kunzea amathicola* (adults and most juveniles), *Kunzea robusta*, and *Kunzea sinclairii*). These groups seem to correlate with the relative sizes of the species’ chromosome complements (de Lange and Murray 2004), and also minor but consistent base changes in their rDNA External Transcribed Spacer (ETS) sequence (de Lange 2007; de Lange et al. 2005). When examining leaves it is important that the distinction between true leaves and pherophylls is recognised, because many of the New Zealand species have mixtures of foliose and squamiform pherophylls, or exclusively foliose pherophylls. Foliose pherophylls often so closely resemble leaves that it is only their proximity to the flowers that clarifies their identity. Leaf indumentum can be highly diagnostic for some species. *Kunzea sinclairii* for example, is the only species with adult leaves that are consistently hairy throughout. Also the presence, absence, persistence and thickness of hairs on the leaf margins, adaxial and abaxial midrib, and whether the hairs converge or fail at the leaf apex, are diagnostic of *Kunzea amathicola*, and *Kunzea triregensis*. Further, the presence of oil glands on the adaxial surface can be used to aid the separation of *Kunzea amathicola* and *Kunzea ericoides* from the other species. In some species, such as *Kunzea amathicola* and *Kunzea triregensis*, the adaxial leaf surface is distinctly glossy, while the other species mostly have dull surfaces—though occasional glossy forms may occur, especially in *Kunzea serotina* and *Kunzea tenuicaulis*. As with growth habit, occasional reversals in leaf indumentum and colour variants may occur. For example, the normally hairy leaved *Kunzea linearis* can occasionally be glabrescent, which is especially the case in the north-eastern area of Te Paki. Further, dark green and bright green foliage variants of species such as *Kunzea robusta* may be found growing together. This appears to be a normal part of the variation exhibited by the New Zealand species, and this is genetically based in some cases. Heterophylly, as already observed, can also aid species separation, especially when it is as marked as it is in *Kunzea amathicola*. In that species, the juvenile foliage is smaller (up to 5.3 × 2.3 mm) than the adult (up to 12.5 × 3.8 mm), long persistent and, coupled with the widely spreading, typically erect to suberect often interwoven branches of juveniles, and their tendency to flower in stressed habitats, readily distinguishes *Kunzea amathicola* from all other New Zealand species.

### Inflorescences

Inflorescence terminology mostly follows Briggs and Johnson (1979) with some modifications. Under their system New Zealand *Kunzea*, in common with their Australian counterparts in Kunzea subg. Niviferae de Lange et Toelken, have blastotelic rather than anthotelic inflorescences (i.e. the inflorescence apex is terminated by a dormant or active vegetative bud) arranged as conflorescences, which are characteristically auxotelic (i.e., the terminal vegetative bud is actively growing). In *Kunzea* the conflorescence is distinctly in the form of a reduced botryum (H.R. Toelken pers. comm.). Briggs and Johnson (1979) further distinguish a range of conflorescence types, which in New Zealand *Kunzea* are either racemiform or spiciform (Fig. 1B). However, for this treatment I refer to plants with the racemiform condition as ‘corymbiform’ (Fig. 1A), because in those species with this inflorescence type, the inflorescence presents as a distinctly corymbose structure at the onset of flowering, a condition which may or may not be progressively lost towards the end of flowering if the apical vegetative bud commences growth (Fig. 1A). Only then is the racemiform type clearly seen. Two species, *Kunzea amathicola* and *Kunzea triregensis*, have distinctively elongated, racemiform inflorescences (Fig. 1C) in which the flowers appear monadic due to their distinctly widely spaced, pedicellate flowers, which are subtended by foliose pherophylls (Fig. 1C, D). This inflorescence type is termed ‘elongate’ (*sensu* Toelken 1996) in this treatment. Only one species, *Kunzea linearis*, has a spiciform inflorescence (Fig. 1B), recognisable by the flowers which are sessile to subsessile, usually with little spacing between them. In this species, and indeed all the other species, but most particularly *Kunzea robusta*, late season inflorescences may develop the elongate condition seen in *Kunzea amathicola* and *Kunzea triregensis*.

### Perules

Perules are the protective, scale-like modified phyllomes (see Briggs and Johnson 1979) that cover a resting bud. In this treatment I follow Toelken (1996) who used them solely for the scales that cover floral primordia (Fig. 1D). The perules of the New Zealand *Kunzea* are rarely diagnostic. In all species the perules are mostly deciduous though occasional specimens with persistent perules were seen.

### Pherophylls

The pherophyll is defined as a foliar organ subtending the flower pedicel (Fig. 1D), or in sessile flowers subtending the flower itself (Briggs and Johnson 1979). Although Briggs and Johnson (1979) used the terms ‘frondose’ and ‘bracteose’ to describe their morphology, I favour ‘foliose’ and ‘squamose’ to distinguish those ‘leaf-like’ pherophylls from those with a ‘scale-like’ morphology. Pherophylls provide a range of diagnostic characters important for species recognition in New Zealand *Kunzea* species. In *Kunzea amathicola*, *Kunzea ericoides*, *Kunzea linearis*, and *Kunzea triregensis*, the pherophyll is greatly enlarged, consistently foliose and usually persistent. In *Kunzea robusta*, *Kunzea salterae*, *Kunzea serotina*, *Kunzea sinclairii*, *Kunzea tenuicaulis* and *Kunzea toelkenii* foliose and squamiform pherophyll types are present and they are typically deciduous. Pherophyll size and shape is often diagnostic of a species. For example, *Kunzea serotina* can be identified by the unique presence of mostly spathulate to spathulate-orbicular foliose pherophylls. Similarly, *Kunzea linearis* can be distinguished by the obliquely ascending and linear to linear-falcate pherophylls. Although pherophylls may be shed early in inflorescence maturation in some species, they are nearly always present on at least some flowers and so form an integral guide toward species recognition.

### Flower buds

The flower buds of the New Zealand *Kunzea* species offer a number of useful characters aiding species recognition. In the species descriptions, shapes and measurements are offered only for what are termed ‘mature’ flower buds, meaning those at the peak of maturation just prior to bud burst. For shapes I have adopted the terminology used for *Eucalyptus* by Brooker and Kleinig (1983). The position of the calyx lobes in mature flower buds and whether they touch each other is important. In most species the apex of the mature bud when viewed from the side is flat or slightly raised and convex (termed ‘domed’ in this revision). In these species the calyx lobes are appressed to and cover the bud surface, with the calyx lobes rarely meeting. However, in *Kunzea linearis* the calyx lobes are touching and consistently pinched inwards towards their apices. *Kunzea triregensis* is occasionally also similar, in which the usually flat or curved, and separated calyx lobes are suberect and touching though not, as in *Kunzea linearis* apically pinched inwards. The calyx lobes of *Kunzea tenuicaulis* are also distinctive in that they are basally thickened, and in most examples a distinct junction between the calyx lobe and the hypanthium apex is evident. This is usually seen as a slight to prominent groove, and is most evident in mature flower buds and young emergent flowers.

### Hypanthia

Hypanthium terminology follows that adopted for *Eucalyptus* by Brooker and Kleinig (1983). While it is important that shapes are determined from mature hypanthia only, the hypanthia of New Zealand *Kunzea* species are highly variable and no single type consistently and uniquely defines any of the species recognised here. Nevertheless, despite the range offered for each species, certain shapes are more commonly associated with particular species and this, taken together with other characters such as indumentum, is usually diagnostic (Table 1). For example, *Kunzea linearis* can be distinguished from the other New Zealand species by its subsessile, mostly barrel-shaped hypanthia, which as a rule have the external faces copiously covered in fine hairs.

### Petals

Petals are rarely diagnostic in most species. Size can be highly variable, and shapes can vary between orbicular and ovate. However, the petal oil glands of *Kunzea serotina* are uniquely yellow pigmented and so diagnostic. In *Kunzea linearis*, the petals of mature flowers are crowded and usually held suberect, often with the upper third weakly recurved. In all other species the petals are spreading. While the usual number of petals is five, a few species, particularly *Kunzea amathicola* and especially *Kunzea triregensis* may have 6 to 8 petals.

### Stamens

At the onset of this study much time was devoted to determining absolute stamen numbers, as well as the ranges of antipetalous to antisepalous stamens for each species. It was soon found that this character was highly labile and, while certain stamen numbers and their positions acted as an approximate field character for some species, there was too much overlap between species. Further, it was observed that stamen numbers of transplanted wild plants were often double or triple that of stamens seen in wild situations. It is now known that stamen number and size in the Myrtaceae is not predetermined. Rather, it is highly variable and the ultimate number is controlled by a diverse range of external and internal factors that can maximise or limit their production (Belsham and Orlovich 2002, 2003; Carrucan and Drinnan 2000; Orlovich et al. 1996, 1998; Ronse Decraene and Smets 1991). All species may produce petaloid stamens and in some species, especially *Kunzea robusta*, these can be quite common. The anthers of New Zealand *Kunzea* are more consistent with respect to their size and shape, and in some species they are especially diagnostic. The anthers of the ten species fall into three main types, mostly testiculate (*Kunzea linearis*, *Kunzea triregensis*, *Kunzea serotina* and *Kunzea toelkenii*), mostly ellipsoid (*Kunzea amathicola*, *Kunzea ericoides*, *Kunzea robusta*, and *Kunzea sinclairii*) or mostly scutiform (shield-shaped) which is the usual condition for *Kunzea salterae*. Between the anthers is a spheroidal connective gland. In most species this is prominent, though in one, *Kunzea amathicola*, it may be absent. The surface texture and colour of the gland when fresh or dry is also diagnostic for a few species (see Table 1).

### Pollen

The pollen of *Kunzea* has been described in detail by Moar (1993) who found no morphological differences between the taxa then recognised and regarded the pollen of the New Zealand representatives difficult if not impossible to distinguish from *Leptospermum scoparium*. This is interesting as Thornhill (2010) was able to distinguish the pollen of Australian *Kunzea* from *Leptospermum*. Irrespective, this study found that average pollen sizes separated *Kunzea linearis*, *Kunzea serotina* and *Kunzea sinclairii* from the other species, and that *Kunzea amathicola*, *Kunzea ericoides*, *Kunzea robusta*, *Kunzea salterae* and *Kunzea tenuicaulis* had pollen ranges that grouped them together, while *Kunzea triregensis* and *Kunzea toelkenii* formed another group (Table 1). There is no obvious correlation between pollen size, and any of the other groupings that are apparent based on leaf size, chromosome complement, or rDNA ETS sequence data (de Lange and Murray 2004; de Lange et al. 2005; de Lange et al. 2010).

### Ovary

The number of locules and ovules per species is rarely diagnostic, though for *Kunzea serotina* and *Kunzea toelkenii* 3–4-locular ovaries are the usual condition, while in *Kunzea amathicola* and *Kunzea robusta* 5–6 locules is usual (Table 1). The number of ovules per species was also not diagnostic. *Kunzea salterae* had 8–10 ovules per locule, which was the lowest range for any of the species and so potentially diagnostic for that species, but so few samples of *Kunzea salterae* were available that it was not clear if the range observed is truly consistent. A possible correlation between ovule number, chromosome complement and leaf size (de Lange and Murray 2004) also requires further evaluation. Taxa with ‘large’ leaves and chromosomes had the highest numbers of ovules per locule (*Kunzea amathicola* up to 42, *Kunzea triregensis* up to 38, *Kunzea robusta* up to 36, *Kunzea sinclairii* up to 34 and *Kunzea linearis* up to 30), and those with smaller chromosome complements and leaves mostly had the lower ovule numbers per locule (*Kunzea salterae* up to 10, *Kunzea serotina* up to 23, *Kunzea tenuicaulis* up to 22 and *Kunzea toelkenii* up to 24). The linear-leaved *Kunzea ericoides* with chromosomes of intermediate size had up to 24 ovules per locule.

### Style and stigma

The style length and stigma shape and diameter at anthesis are potentially useful for species recognition. Although style length can be variable, it can distinguish most species from each other when used in conjunction with branchlet indumentum and leaf size and shape. However as a field character, style length is difficult to use and measurements show considerable variation depending on when they were taken because the styles in all species continue to elongate after petal drop. For this revision I have made measurements only from freshly opened and fully expanded flowers prior to pollination. Pollination was easily determined for most species by the change in colour of the receptacle from green or light red or pink to dark crimson, a colour change universally recognised in Australia (H. R. Toelken pers. comm.) to coincide with pollination. Accepting the limitations of undertaking style length measurements, one species, *Kunzea serotina* consistently emerged as having the shortest style length.

While all species recognised here have a capitate stigma, the relative size and shape, as seen when viewed from above and on the side proved useful. Two main groups are evident, those with prominent ‘broad’ stigma that were one-half or more as wide as the style diameter, and those with narrowly capitate stigma as wide as or only slightly wider than the style. Species with broad stigma are *Kunzea amathicola*, *Kunzea triregensis* and *Kunzea robusta*, with *Kunzea ericoides* and *Kunzea sinclairii* occupying somewhat intermediate states. The remaining species had narrowly capitate stigmas. An approximate correlation between stigma diameter and the size of their chromosome complements (*sensu* de Lange and Murray 2004) is suggested, with most of the species that have ‘large’ chromosomes also having broad stigmas (the exception being *Kunzea sinclairii*) and the rest of the species with ‘intermediate’ or ‘small’ chromosome complements, narrowly capitate ones. Two species with narrowly capitate stigma, *Kunzea serotina* and *Kunzea tenuicaulis*, were further distinguished by their stigma having a distinctly domed margin and a depressed concave centre when viewed from the side. All the other species, irrespective of size, had flat stigma surfaces.

### Fruit

Fruit shape follows the terminology used for *Eucalyptus* by Brooker and Kleinig (1983) and in their subsequent publications. New Zealand *Kunzea* have a wide range of fruit shapes of which the most frequently encountered is obconic (including narrow or broad variations). However, campanulate, cupular, barrel-shaped and hemispherical fruits are also common, with urceolate fruits confined to *Kunzea serotina* (though the fruits of *Kunzea salterae* can occasionally be suburceolate). Although most species show a range of fruit shapes, some (including the aforementioned *Kunzea serotina*) have particular fruit shapes diagnostic for that species. Thus *Kunzea linearis* and *Kunzea tenuicaulis* usually have barrel-shaped fruits, *Kunzea ericoides* and *Kunzea salterae* cupular, *Kunzea triregensis* hemispherical, and *Kunzea amathicola*, *Kunzea robusta*, *Kunzea sinclairii* and *Kunzea toelkenii* obconic. Size ranges show much overlap between species, though *Kunzea amathicola* and *Kunzea robusta* have consistently the largest fruits sizes and *Kunzea serotina* the smallest. Fruit persistence is also a useful guide. Several species are characterised by long persistent fruits, meaning that the fruits are retained well after the seed is shed, usually with some retained almost permanently on established trees. Species with such persistent fruits include *Kunzea amathicola*, *Kunzea linearis*, *Kunzea triregensis*, *Kunzea sinclairii* and *Kunzea tenuicaulis*. *Kunzea ericoides*, *Kunzea robusta*, *Kunzea salterae*, *Kunzea toelkenii* and *Kunzea serotina* usually have deciduous fruits, meaning that they rarely persist longer than three or so months after seed dehiscence. As fruit size, shape and persistence are often diagnostic of particular species, fruits are an important tool enabling species recognition.

### Seed

Webb and Simpson (2001) observed that seed descriptions were sorely lacking for many indigenous New Zealand vascular plants. In their treatment they provided the first detailed descriptions and illustrations of New Zealand *Kunzea* seed. They concluded that there was little morphological or size difference between the taxa they recognised. This is still true because, despite the recognition of seven new species in this revision, all species overlap in seed colour, size and shape. It should be noted that in all species the ‘reticulum’ of the seed (as described by Webb and Simpson 2001) results from the collapse of the cells of the outer layer leaving protruding anticlinal walls, rather than any true ornamentation of the cell surfaces. Although this ‘reticulum’ presented little variation between species, in some of the seed of one species *Kunzea salterae*, apparently unique, spiny protuberances arising from the centre of the collapsed periclinal wall of some cells of the ‘reticulum’ were present (Fig. 23K, L). This observation requires further assessment as only one seed sample of this species was available for study.

### Chromosome and molecular evidence

This revision uses data obtained from cytological analysis of the New Zealand members of the *Kunzea ericoides* complex and published in de Lange and Murray (2004) and de Lange et al. (2005). Molecular data (Table 2) obtained from the rDNA Internal Transcribed Spacer (ITS) and ETS regions (de Lange 2007; de Lange et al. 2010) is also used here to help define species and show possible relationships.

**Table 2.** Sites of character variability within Australian and New Zealand taxa and informal entities of the *Kunzea ericoides* complex (from de Lange 2007).

Taxon/Informal Entity	ITS-1											ITS-2				ETS																				
Alignment Position	542	548	553	581	594	639	646	671	724	742	756	994	1020	1028	1077	18	41	62	68	75	123	153	201	202	210	213	221	232	252	259	269	274	275	276	286	404
**Australian *Kunzea ericoides* complex**																																				
*Kunzea leptospermoides* F.Muell ex Miq.	a	*	c	a	a	g	c	c	g	t	t	g	c	*	g	c	a	a	g	c	a	c	g	a	a	c	c	c	c/a	g	*	a	a	t	*	c/t
*Kunzea peduncularis* F.Muell.	a	*	c	*	*	g	c	c	g	t	t	g	c	c	g	c	a	a	g	c	a	c	g	a	a	c	c	c	c	g	*	a	a	t	*	c
*Kunzea phylicioides* (A.Cunn. ex Schauer) Druce	a/t	a	c	*	*	g	c	c	g	t	t	g	c	*	g	c	a	a	g	c	a	c	g/a	a	a	c	c	c	c	g	*	a	a	t	*	c
Kunzea aff. peduncularis	t	a	c/t	*	*	g	c/t	c	g	t	t	g	c	*	g	c	a	a	g	c	a	c	g	a	a	c/t	c	c	c	g	*	a	a	t	*	c
Kunzea aff. ericoides (g)	a	*	c	*	*	g	c	c	g	t	t	g	c	*	g	c	a	a	g	t	a	c	g/a	a	a	c	c	c	c	g	*	a	a	t	*	c
Kunzea aff. ericoides (h)	a	*	c	*	*	g	c	c	g	t	t	g	c	c	g	c	a	a	g	c	a	c	g	a	a	c	c	c	c	g	*	a	a	t	*	c
Kunzea aff. ericoides (i)	a	a	c	*	*	g	c	c	c	t	t	g	c	*	a	c	a	a	g	c	g	c	a	a	a	c	t	c	c	g	*	a	a	t	*	c
**New Zealand *Kunzea ericoides* complex**																																				
*Kunzea amathicola*	a	c	c	*	*	g	c	t	g	g/t	c	g	c	*	g	c	a	a	g	c	g	c	g	a	g	c	c	c	c	g	g	*	*	*	*	c
*Kunzea ericoides*	a	c	c	*	*	g	c	c	g	t	c	g	c	*	g	c	a	a	a	c	g	c/t	g	a	g	c	c	g/c	c	g	c	*	*	*	*	c
*Kunzea linearis*	a	c	c	*	*	g	c	c	g	t	c	g	c	*	g	c	g	a	a	c	g	c	g	a	g	c	c	g/c	c	g/a	c	*	*	*	*	c
*Kunzea robusta*	a	c	c	*	*	g	c	c	g	t	c	g	c	*	g	c	a	a	a	c	g	c	g	a	g	c	c	c	c	g	g	*	*	*	*	c
*Kunzea robusta* (Mt Egmont only)	a	c	c	*	*	g	c	c	g	t	c	g	c	*	g	c	a	a	a	c	g	c	g	a	g	c	c	g/c	c	g	g	*	*	*	*	c
*Kunzea tenuicaulis*	a	c	c	*	*	a	c	c	g	g/t	c	a	c	*	g	t	a	a	a	c	g	c	g	c	g	c	c	c	c	g	a	*	*	*	*	c
*Kunzea toelkenii*	a	c	c	*	*	g	c	c	g	t	c	g	c	*	g	c	a	a	a	c	g	c	g	a	g	c	c	g/c	c	g	a	*	*	*	*	c
*Kunzea triregensis*	a	c	c	*	*	g	c	c	g	t	c	g	c	*	g	c	a	a	a	c	g	c	g	a	g	c	c	c	c	g	*	*	*	*	*	c
*Kunzea salterae*	a	c	c	*	*	g	c	c	g	g/t	c	g	c/t	*	g	c	a	a	a	c	g	c	g	a	g	c	c	g/c	c	g	a	*	*	*	*	c
*Kunzea serotina*	a	c	c	*	*	g	c	c	g	t	c	g	c	*	g	c	a	a	a	c	g	c	g	a	g	c	c	g/c	c	g	a	*	*	*	*	c
*Kunzea sinclairii*	a	c	c	*	*	g	c	c	g	t	c	g	c	*	g	c	a	a	a	c	g	c	g	a	g	c	c	c	c	g	g	*	*	*	*	c
Kunzea aff. ericoides " Lottin Point"	a	c	c	*	*	g	c	c	g	t	c	g	c	*	g	c	a	t	g	c	g	c	g	a	g	c	c	c	c	g	*	*	*	*	g	c

Key:		- unique character										- shared character										- shared Australian character														
		- shared character										- shared character																								

### Species concept

The *Kunzea* species recognised here are, in common with other Australasian Myrtaceous genera, recognised by combinations of morphological characters including cryptic lines of evidence based on chromosome complements (de Lange 2007; de Lange and Murray 2004; de Lange et al. 2005; de Lange et al. 2010). Molecular data obtained from rDNA ITS and ETS sequences (de Lange 2007; de Lange et al. 2010) in particular the sympatry and indeed syntopy of ribotypes correlated here to morphological species, provides another line of evidence to justify taxonomic segregation of *Kunzea ericoides* (see de Lange 2007; de Lange et al 2010). Extensive use has also been made of species distribution, their degree of sympatry and their ecology, and extent of hybridism (see in particular de Lange et al. 2005). All species, except *Kunzea triregensis*, are sympatric with at least one, sometimes three other species, with which they usually show ecological partitioning. Syntopy is also frequent, particularly in such variable landscapes as those caused by geothermal systems in which active (heated), and recently quiescent (cold), substrates, can occur within metres of each other. Thus, in these habitats, it is not uncommon to find the geothermal endemic *Kunzea tenuicaulis* flourishing on heated ground and active fumaroles, and within a distance of several metres on adjacent ‘cold’ ground ‘inliers’ *Kunzea robusta*, and, less frequently *Kunzea serotina*. In these situations, all three species may not only grow together but, because of the growth habit of *Kunzea tenuicaulis*, the plants may be completely intertwined.

As in the Australian species (see Toelken 1996; Tierney and Wardle 2004; de Lange et al. 2005), hybridism is a feature of New Zealand *Kunzea* and nearly all experimental crosses investigated by de Lange et al. (2005) were found to be fully fertile. However, hybrids are usually scarce in natural systems in the wild and, following a now well established world-wide pattern (Anderson 1949; Brockie 1959; Stebbins 1959; Hair 1966; Raven and Raven 1976), are abundant only in those sites that have been and continue to be extensively modified by humans. Nevertheless, the ability to hybridise and in particular for hybrids to form fully fertile introgressive swarms has been viewed by some as sufficient reason to rule out taxonomic recognition (see the review by Stace 1989). However, as Stace (1989) then argues, that view is being increasingly regarded as unnecessarily conservative and naïve. For New Zealand in particular, it is now well established that hybridism is not only a feature of the indigenous flora but that it is a critical speciation pathway adopted there by many genera and species (Cockayne 1929; Cockayne and Allan 1934; Hair 1966; Connor 1985; Molloy et al. 1999). Therefore there is no logic in excluding morphologically and ecologically distinct taxa simply because they form hybrids. Indeed, to adopt such conservative criteria would effectively eliminate a wide range of morphologically diverse and distinct, universally accepted New Zealand species in such genera as *Asplenium*, *Celmisia*, *Chionochloa*, *Coprosma*, *Corokia*, *Elaeocarpus*, *Epilobium*, *Gaultheria*, *Hebe*, *Lepidium*, *Leucogenes*, *Leptinella*, *Lobelia*, *Melicytus*, *Metrosideros*, *Muehlenbeckia*, *Myrsine*, *Ranunculus* and *Sophora* (Oliver 1935; Allan 1961; Franklin 1962, 1964; Rattenbury 1962; Fisher 1965; Lloyd 1972; Raven and Raven 1976; Brownsey 1977a; Given 1980b, 1984; Connor 1991; Molloy 1995; Molloy and Clarkson 1996; Heenan et al. 2001; Heenan and de Lange 2004; Murray et al. 2004; de Lange et al. 2013a). All of the species in these genera routinely hybridise, and some can form extensive introgressive hybrid swarms. They also include species known to have resulted from past hybridisation events. Therefore, the presence of hybrids is not seen as any strong justification to avoid taxonomic recognition of what are otherwise distinctive entities in the New Zealand *Kunzea ericoides* complex. Instead it is suggested that hybridism within the genus is an important speciation mechanism. Indeed, past hybridism is postulated for the origin of at least three species (*Kunzea triregensis*, *Kunzea salterae* and *Kunzea toelkenii*) recognised in this treatment. Similar evolutionary pathways involving hybridism have been proposed for a range of New Zealand species both as means toward further speciation, and as a survival mechanism during times of adversity (see Rattenbury 1962). Many species with suspected hybrid origins that had been postulated on morphological and sometimes experimental evidence have now been confirmed by molecular evidence, while yet others newly recognised to science have been shown to have evolved from hybrids (see Lloyd 1972; Brownsey 1977a, 1977b; Molloy 1995; Blanchon et al. 2002; Wichman et al. 2002; Gardner et al. 2004; Perrie and Brownsey 2005). Hybridism needs to be viewed in the context of the genus under revision (Stace 1989) and no simple rule applies. As it is normal for *Kunzea* species to hybridise throughout their range, often forming introgressive hybrid swarms (Toelken 1996; Tierney and Wardle 2004; de Lange et al. 2005), the ability to hybridise is not regarded as a valid reason to reject taxonomic segregation. Therefore, formal taxonomic recognition is accorded here to any *Kunzea* entity which demonstrates consistent morphological, cytological, molecular and ecological partitioning irrespective of whether it can or does hybridise, so according with the unified species concept advocated by de Queiroz (2007). No subspecies are recognised in this treatment, all species are sympatric with at least one other species, except *Kunzea triregensis* which is the sole species present on the Three Kings Islands. *Kunzea triregensis* has been accorded species rank because it has the same (or even greater) levels of morphological distinction than several other sympatric species accepted here, e.g., *Kunzea sinclairii*.

### Ethnobotany

In this paper I have adopted the heading ‘vernacular names’ wherein I have recorded the names given to *Kunzea* by the indigenous Maori people of New Zealand. I have done this because in my studies it became clear that Maori have long recognised the distinctive nature of at least four of the species treated here, furnishing these with names in *te reo Maori* (their language). Further these names were often attributed to some past use or wood property of the species concerned, though in some cases the meanings have already become lost. Nevertheless these names serve as an important record of the connection between these people and the indigenous flora of Aotearoa (New Zealand).

## Systematics

### Kunzea

Taxon classificationPlantaeMyrtalesMyrtaceae

Rchb. nom. cons.

Kunzea Rchb. *Consp. Regn. Veg.*: 175. (Dec 1828) nom. cons.
Stenospermum Sweet ex Heynh., *Hort. Brit. (Sweet), ed. 2*: 209 (1830) *nom. inval.* (*fide*Toelken 1981a)
Tillospermum Salisb., *Monthly Rev. 75*: 74 (1814) *nom. rej.*
Kunzia Sprengel *nom. superf.* (*fide*Toelken 1981a, 1981b)
Pentagonaster Klotsch in Otto et Dietrich, *Allgemeine Gartenzeitung IV*: 112 (1836)
Salisia Lindl. *Sketch. Veg. Swan R.* 10 (1839)

#### Lectotype species.

*Kunzea capitata* (Sm.) Heynh. (*fide* Toelken 1981b)

#### Description.

Creeping shrubs, shrubs, small or tall trees with or without lignotubers and rhizomes. Leaves mostly alternate, opposite in a few species. Inflorescences reduced conflorescences (botrya) usually pseudoterminal, globose to spiciform or cylindrical bearing sessile to subsessile flowers, otherwise corymbiform to elongate, with pedicellate flowers rarely reduced to solitary. Flowers 5-merous, red, pink, purple, yellow or white, free part of hypanthium usually exceeding the ovary summit. Calyx persistent in fruit. Petals free often much reduced. Stamens mostly numerous, in one or more series, exceeding petals or included; filaments finely striated, anthers versatile. Ovary mostly 2–3-locular sometimes up to 5–6-locular; placentation axillary and ovules spreading, numerous, to apical with few larger pendent ovules. Fruit a capsule, usually loculicidal, mostly dry, rarely indehiscent or fleshy, not persisting. Chromosome number: 2*n* = 22 based on *x* = 11 (Dawson 1987; de Lange and Murray 2004).

#### Distribution.

Australia: c.54 spp. (all endemic) New Zealand: 10 spp. (all endemic).

#### Key to New Zealand *Kunzea*

This key requires material with active new growth, buds, flowers, and ideally seedlings. In some species, such as *Kunzea amathicola*, *Kunzea linearis* and *Kunzea triregensis*, the inflorescence condition can be easily determined in the absence of flowers from fruiting specimens, as fruits in these species are especially persistent. Use young growth only to determine branchlet indumentum, and examine the hairs produced 10–20 mm back from the branchlet tip–this is important as some species produce sparse, deciduous, antrorse-appressed hairs at the base of the actively growing branchlet apices. This key will not resolve hybrids, but these may be recognised by the hybridism notes given for each species. Geographic and ecological information is included in this key as a further aid to identification. For example, on Moutohora (Whale Island), Bay of Plenty, North Island, New Zealand, *Kunzea salterae* can very occasionally (only one specimen with this condition seen) have glabrescent to almost fully glabrous branchlets, and so would key out to *Kunzea ericoides*. In these very rare instances, in the absence of flowers and fruits, such specimens could only reliably be identified by their location.

**Table:** 

1a	Branchlet hairs on new season growth mostly divergent; divergent hairs up to 0.1 mm long	2
1b	Branchlet hairs on new season growth mostly antrorse-appressed; hairs up to 0.7 mm long	6
2a	Mature branchlets glabrescent; branchlet hairs strictly divergent, 0.02–0.05 mm long; pherophylls foliose, ± persistent, narrowly elliptic, lanceolate to narrowly lanceolate; hypanthia sharply obconic, glabrous (very rarely sparsely hairy); endemic to the northern South Island, New Zealand	1 *Kunzea ericoides*
2b	Mature branchlets hairy; branchlet hairs mostly divergent 0.03–0.12 mm long; pherophylls foliose or squamiform, deciduous, spathulate, spathulate-orbicular, rarely pandurate, oblong, oblong-obovate to oblanceolate, shortly lanceolate or broadly to narrowly linear-lanceolate; hypanthia urceolate, campanulate, narrowly cupular, funnelform to obconic, puberulent (very rarely glabrescent); not endemic to the South Island, New Zealand	3
3a	Branches obliquely ascending, fastigiate; pherophylls foliose not squamiform, mostly spathulate (sometimes pandurate); petals with yellow oil glands when fresh	2 *Kunzea serotina*
3b	Branches spreading to widely spreading, suberect to erect and ascending but not obliquely ascending or fastigiate; pherophylls foliose, squamiform or usually both, never spathulate; petals with rose-pink or colourless oil glands when fresh, or oil glands not evident fresh or dry	4
4a	Plants heterophyllous; branchlet hairs copious, divergent, weakly flexuose, 0.03–0.08 mm long, apices ± straight; leaves of juveniles and reversion shoots linear-lanceolate, 0.9–3.0(–4.5) × 0.2–0.4(–0.6) mm long, sometimes long persistent; calyx-lobes distinctly thickened toward the base, and with an obvious external junction with the hypanthium; species confined to active geothermal habitats of the mainland Taupo Volcanic Zone, North Island, New Zealand	3 *Kunzea tenuicaulis*
4b	Plants not heterophyllous, branchlets glabrescent or hairy; if hairy then hairs of two types, antrorse-appressed (often deciduous) straight to weakly flexuose, up to 0.55 mm long, or divergent, up to 0.12 mm long, with curled apices; adult leaves variable, if linear-lanceolate then 4–10(–18) × 0.6–1.2(–2.0) mm long; calyx lobes of hypanthia not thickened toward base; species of mostly non geothermal habitats of Moutohora (Whale Island) and the coastal Bay of Plenty, North Island, New Zealand	5
5a	Epicormic growth and suckers absent; branchlets hairy (rarely glabrescent); hairs in mixtures of longer (up to 0.55 mm long), deciduous, antrorse-appressed hairs and shorter (up to 0.10 mm long), persistent, divergent hairs with ± curled apices; antrorse-appressed hairs confined to active branchlet tips; adult leaves linear-lanceolate to narrowly oblanceolate; species endemic to Moutohora (Whale Island), Bay of Plenty, New Zealand, where widespread, and sometimes found in active geothermal habitats	4 *Kunzea salterae*
5b	Epicormic growth and suckers frequent, prostrate and widely trailing from trunk base; branchlet hairs copious, persistent, in mixtures of divergent and antrorse-appressed hairs; antrorse-appressed hairs straight up to 0.03 mm long, not confined to active branchlet tips; divergent hairs up to 0.14 mm long, apices strongly curled and spiralled; leaves mostly obovate to clavate, sometimes broadly oblanceolate; species endemic to mobile sand systems of the eastern Bay of Plenty, Bay of Plenty, North Island, New Zealand not known from geothermal habitats	5 *Kunzea toelkenii*
6a	Inflorescences spiciform; leaves consistently linear with distinctly hairy margins and abaxial midrib (rarely glabrescent); pherophylls obliquely ascending, linear to linear-falcate; flowers sessile to subsessile; calyx lobes sharply erect and apically pinched inwards in mature flower buds	6 *Kunzea linearis*
6b	Inflorescences elongate or corymbiform, never spiciform; leaves variable but rarely linear (if linear then glabrescent, and with inflorescences consistently corymbiform); pherophylls spreading or recurved, oblong, oblong-obovate, broadly oblong to elliptic, narrowly deltoid, narrowly lanceolate, lanceolate, oblanceolate or rarely broadly spathulate; flowers pedicellate; calyx lobes flat or slightly domed in mature flower buds, rarely suberect, if so then not apically pinched inwards	7
7a	Inflorescences elongate, never corymbiform; pherophylls foliose, persistent	8
7b	Inflorescences initially corymbiform, sometimes elongate toward end of flowering season; pherophylls foliose and squamiform, mostly deciduous, rarely persistent	9
8a	Plants heterophyllous; shrubs or trees of mainly coastal mobile sand systems; reversion shoots and epicormic growth occasional; juvenile long persistent, often flowering; juvenile leaves ovate, broadly ovate, rhomboid to obovate; adult leaves oblong, oblong-obovate, broadly oblanceolate to lanceolate; pherophylls oblong, oblong-obovate, broadly oblong to elliptic; species endemic to North and South Islands of New Zealand, not known from the Three Kings Islands group	7 *Kunzea amathicola*
8b	Plants not heterophyllous; trees of coastal shrubland and forest; reversion shoots and epicormic growth absent; leaves lanceolate to narrowly lanceolate; pherophylls broadly lanceolate to lanceolate; species endemic to the Three Kings Islands group	8 *Kunzea triregensis*
9a	Prostrate, widely spreading shrubs (very rarely small trees up to 6 m tall) of mainly exposed, sparsely vegetated rhyolitic rock and talus; new growth subscandent; adult leaf surfaces densely covered in persistent, long (0.45–1.23 mm long) antrorse-appressed hairs; lamina silvery white, silvery-grey to reddish-grey; species endemic to Aotea (Great Barrier Island), New Zealand	9 *Kunzea sinclairii*
9b	Erect trees up to 30 m tall of coastal to montane successional forested habitats; new growth initially erect, soon widely spreading, rarely pendulous, never subscandent; adult leaf surfaces glabrous except for margins and midrib, these ± finely covered with a thin, often interrupted band of deciduous hairs tending toward glabrate; lamina light to dark green; widespread throughout the main islands of New Zealand	10 *Kunzea robusta*

### 1. Kunzea ericoides

Taxon classificationPlantaeMyrtalesMyrtaceae

(A.Rich.) Joy Thomps.

Leptospermum ericoides A.Rich in *Essai. Fl. N.Z.*, (1832), 338

#### Lectotype

**Figure 2.**
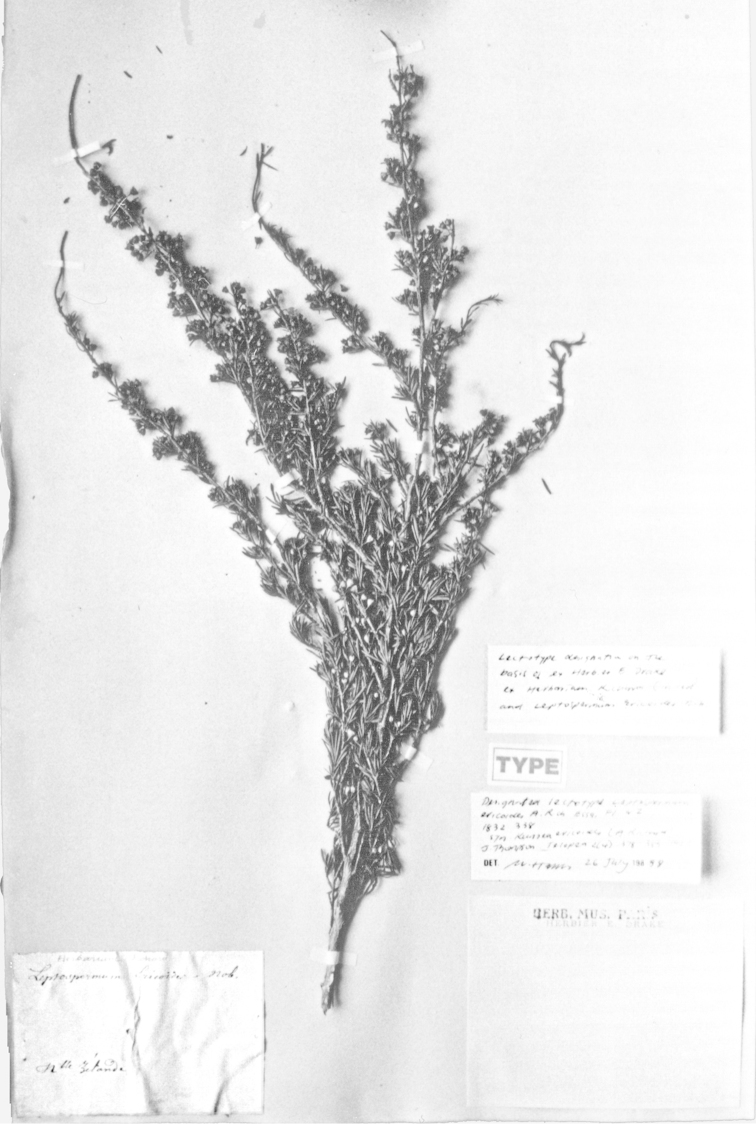
Lectotype of *Leptospermum ericoides* A.Rich. (held at l’Herbier du Laboratoire de Phanérogamie du Muséum National d’Histoire Naturelle (P)).

**(here designated)** **(Fig. 2).** ‘*Leptospermum ericoides* nob. N^lle^ Zélande’ Herbarium Richard, Ex. Herbier E. Drake, P! Specimen labelled ‘TYPE’ in bold red lettering and bearing two handwritten labels by W. Harris dated 26 July 1989.

#### Paralectotype

**Figure 3.**
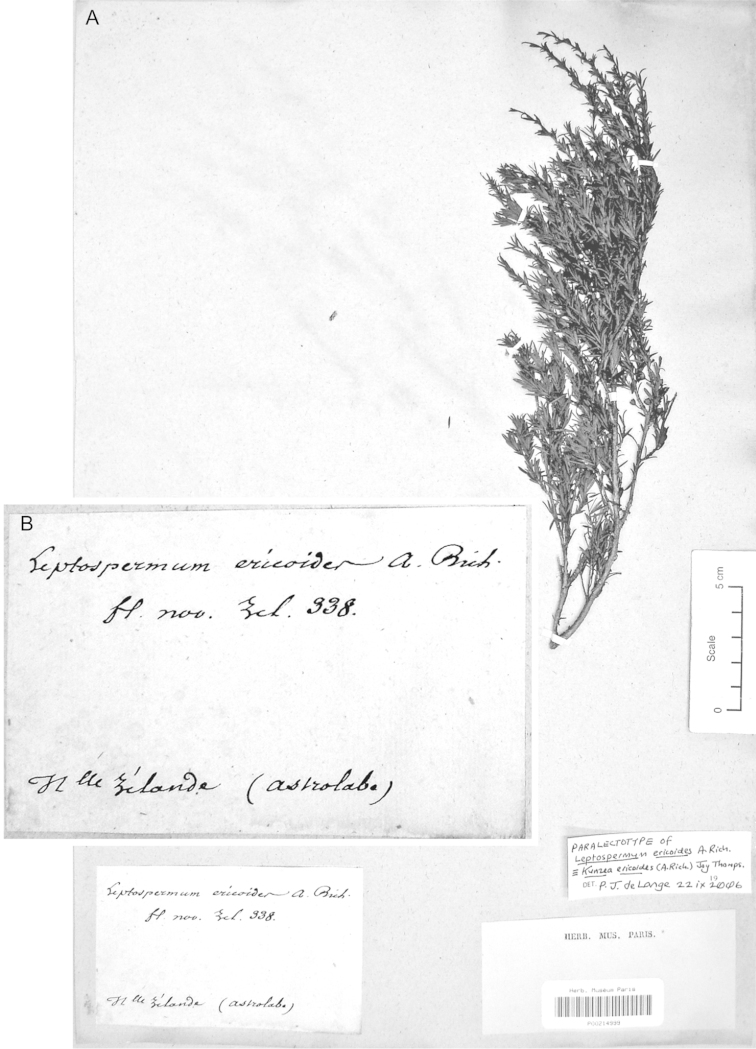
Paralectotype of **A** *Leptospermum ericoides* (held at l’Herbier du Laboratoire de Phanérogamie du Muséum National d’Histoire Naturelle (P)). **B** Enlargement of paralectotype label showing Achille Richard’s distinctive handwriting.

**(here designated)** **(Fig. 3).** ‘*Leptospermum ericoides* A.Rich. fl N^lle^ Zél 338 N^lle^ Zélande (Astrolabe)’ P214999!

#### Notes.

Allan (1961; p. 322), Thompson (1983) and Harris and Cadic (1998; p. 36) published partial lectotypifications of *Leptospermum ericoides* A.Rich (see Article 9, of the International Code of Nomenclature (McNeill et al. 2012). The first two authors did this through their citation of elements of the protologue and by their indication of where type material was lodged, while Harris and Cadic (1998) also published statements of their intent to typify, noting also the location of type material and stating that they had seen ‘Richard’s type’. However, because there are at least two syntypes of *Leptospermum ericoides* A.Rich. in l’Herbier du Laboratoire de Phanérogamie du Muséum National d’Histoire Naturelle (P) (Figs 2, 3) that bear the distinctive handwriting of Achille Richard (Fig. 2, 3; see also Burdet 1978) and match the protologue with respect to their morphological condition and collection details, and because none of these author’s publications specified which one of these was the lectotype, a further lectotypification is necessary. Of the two sheets that I have seen, both are without date or collector. One (Fig. 2) from the ‘Herbarium Richard’ has two labels. The first of these is in Richard’s hand and reads ‘Leptospermum ericoides nob. N^lle^ Zélande’. The second label is printed ‘HERB. MUS. PARIS’ and ‘HERBIER E. DRAKE’. This sheet has also been stamped by the herbarium staff (P. Morat pers. comm.) ‘TYPE’ and bears two handwritten labels by W. Harris. The upper label states ‘Lectotype designation on the basis of ex Herbier E. Drake ex Herbarium Richard (in red) and *Leptospermum ericoides* Rich.’ and the next reads ‘Designated Lectotype *Leptospermum ericoides* A.Rich. Essai. Fl. N.Z. 1832, 338, syn. *Kunzea ericoides* (A. Richard) Joy Thompson Telopea 2(4) 378, 1983 W. Harris 26 July 1988’. The second herbarium sheet (Fig. 3) also bears two labels (Fig. 2A, B). The first of these (see Fig. 3B) is written in Richard’s hand, and reads ‘*Leptospermum ericoides* A.Rich. fl. Nov. Zél. N^lle^ Zélande (Astrolabe)’, the second (Fig. 3A) is printed ‘HERB. MUS. PARIS’. I designate as lectotype, the sheet labelled by Richard ‘*Leptospermum ericoides* nob.’ because this matches his protologue (p. 338) with respect to the usage of the possessive Latin abbreviation ‘nob.’ i.e. *nobis*, meaning ‘to us, of us’ in the sense of ‘this is my [choice of] name’ (Stearn 1992; R. O. Gardner pers. comm.). Further, it is the only sheet clearly identified as part of Richard’s herbarium. This was also the sheet designated lectotype by Harris. However, because Harris did not publish this, his lectotypification, although accurate cannot be upheld.

I conservatively designate as paralectotype the second sheet at P. This is because it is without date or collection notes so it is impossible to tell if it was part of the same gathering as the lectotype.

#### Etymology.

The specific epithet *ericoides* alludes to the similarity in the growth form of *Kunzea ericoides* to *Erica arborea* L. i.e., ‘*Ericam arboream* habitu referens’ (Richard 1832; p. 338).

#### Description

**Figure 4.**
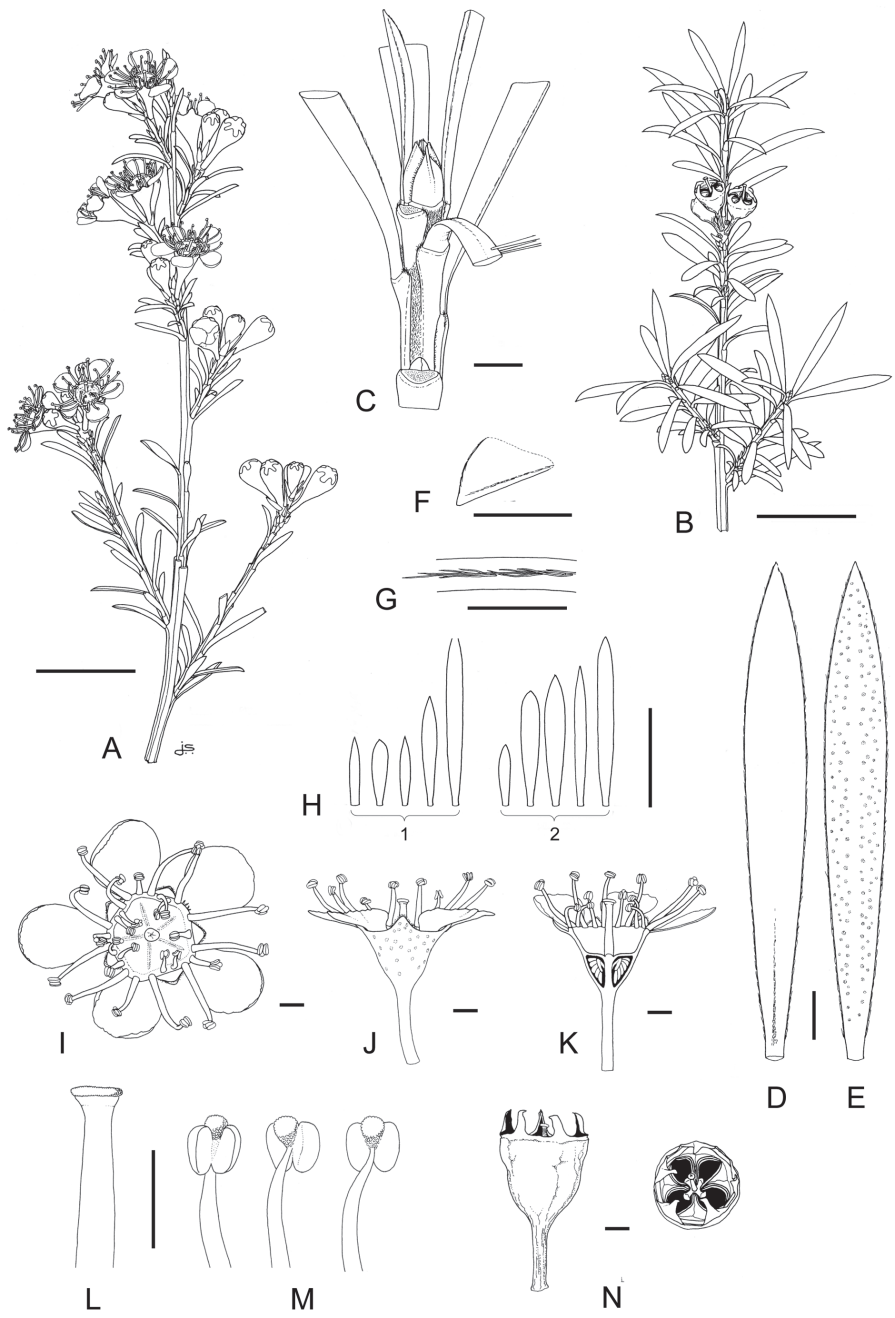
Distinguishing features of *Kunzea ericoides*. **A** Flowering branchlet (ex cult. AK 289138) **B** Fruiting branchlet (ex cult. AK 289138) **C** Vegetative bud and branchlet indumentum (ex cult. AK 289138) **D** Adaxial leaf surface (ex cult. AK 289138) **E** Abaxial leaf surface (ex cult. AK 289138) **F** Adaxial leaf apex (ex cult. AK 289138) **G** Leaf margin indumentum (ex cult. AK 289138) **H** Leaf variation: (**H1**) Abel Tasman National Park, Astrolabe Roadstead, (AK 253380), (**H2**) Knuckle Hill (AK 289160) **I** Flower (top view) (AK 289138) **J** Flower and hypanthium (side view) (AK 289138) **K** Flower cross section showing anther, style and ovules (AK 289138) **L** Style and stigma (AK 289138) **M** Stamen (AK 289138) **N** Dehisced fruit (AK 253380). Scale bars: (**A, B, H**) 10 mm; (**C–F, I–N**) 1 mm; (**G**) 0.5 mm.

**Figure 5.**
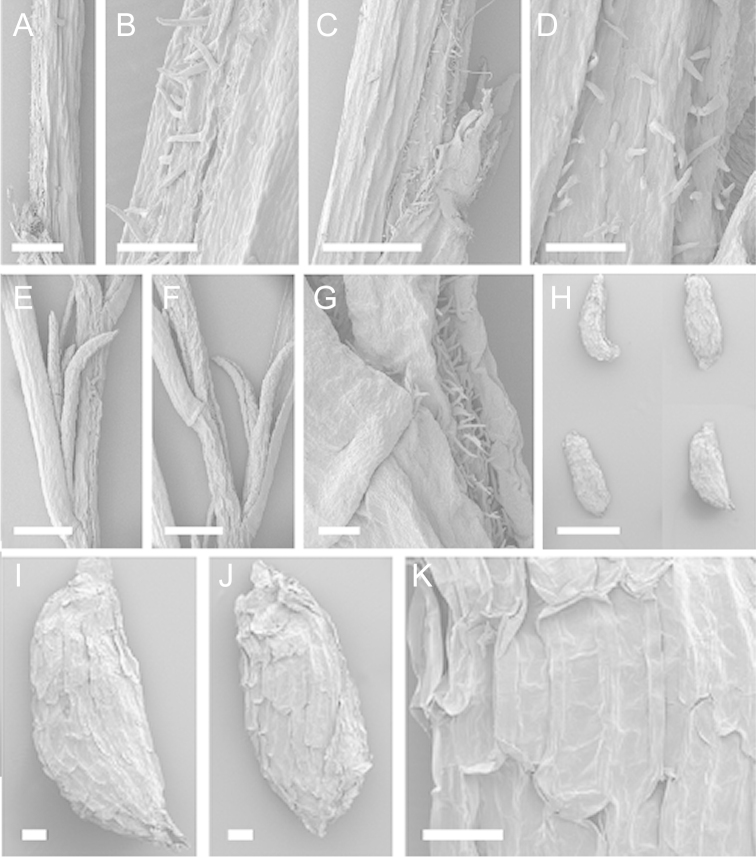
Scanning Electron Micrographs of *Kunzea ericoides*. **A–G** Branchlet indumentum (AK 253380; AK 289161); Seeds **H–K** (HR 3766 **K** Testa surface showing reticulum (CHR 3766). Scale bars: (**A, C**) 500 μm; (**B, D, G, H, J–K**) 100 μm; (**E, F, I**) 1 mm.

**Figure 6.**
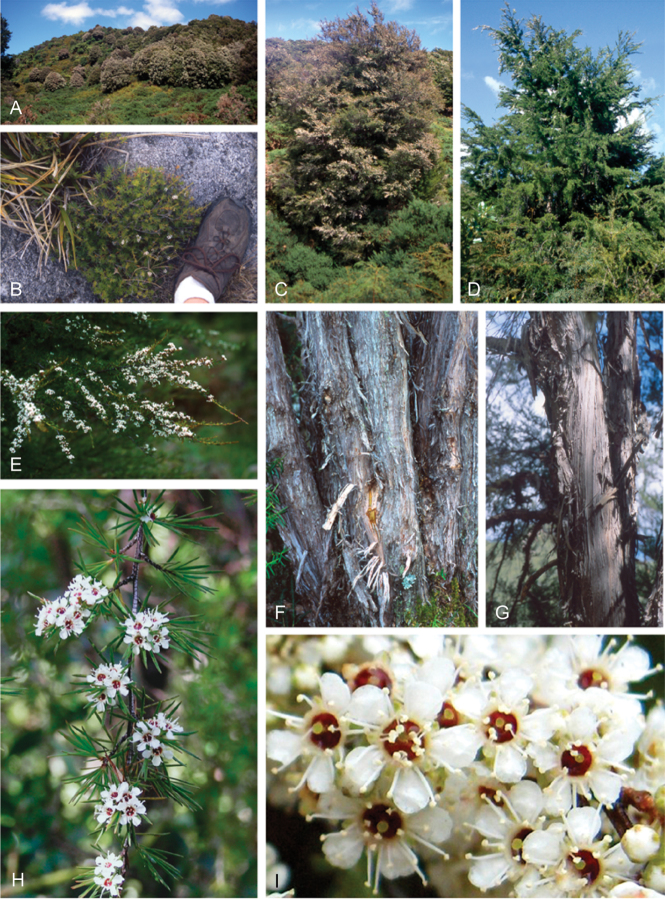
*Kunzea ericoides*. **A** *Kunzea ericoides* trees colonising old burn, South Island, north-west Nelson, Abel Tasman National Park, Awapoto River (photo: *P. J. de Lange*) **B** Flowering decumbent plant on windswept ridge line, South Island, north-west Nelson, Wakamarama Range, Knuckle Hill (photo: *P. J. de Lange*) **C** Young *Kunzea ericoides* tree, South Island, north-west Nelson, Marahau (photo: *P. J. de Lange*) **D** Mature *Kunzea ericoides* tree, South Island, north-west Nelson, Canaan Downs (photo: *G. M. Crowcroft*) **E** Flowering branchlets, South Island, eastern Nelson, Richmond Range, Hackett Creek (photo: *G. M. Crowcroft*) **F** Bark, South Island, eastern Nelson, Richmond Range, Hackett Creek (photo: *G. M. Crowcroft*) **G** Bark, South Island, north-west Nelson, Canaan Downs (photo: *G. M. Crowcroft*) **H** Flowering branchlet showing brachyblasts, leaves and few-flowered corymbiform botrya, South Island, north-west Nelson, Golden Bay, Pupu Springs (photo: *M. D. Wilcox*) **I** Close up of flowers, South Island, north-west Nelson, Golden Bay, Bishops Saddle (photo: *G. M. Crowcroft*).

(Figs 4, 5, 6). *Growth habit* mostly trees up to 18 m, sometimes (such as on ultramafic rocks and soils) decumbent and trailing forming shrubs up to 2 × 1 m. Plants with tree-habit usually rather slender and gracile with a somewhat spreading canopy; those in exposed conditions branching at or close to the trunk base, while those growing in dense stands or sheltered sites usually with the lower half devoid of branches. Plants with a decumbent habit usually heavily branched, not rooting on contact with soil. *Trunk* 1(–4) arising from the ground, 0.10–0.60(–0.85) m d.b.h., mostly erect, slender, weakly flexuose; often basally buttressed, mature trees usually devoid of branches for the first 1–2(–4) m; decumbent plants with scarcely discernible trunk due to branches arising from or close to the base; basal portion of trunks covered with layers of somewhat firm to semi-detached, weakly tessellated, short to long, ± irregularly tabular lengths of subcoriaceous brown-grey to greyish-white bark. *Bark* early bark chartaceous to subcoriaceous, brown to grey-brown, ± elongate, usually bearing a few transverse cracks (especially on branch flanges and decurrent leaf bases) otherwise remaining firmly attached, margins elongate sinuous, ± entire with scarcely any flaking; old bark similar though more distinctly corky subcoriaceous, often coarsely tessellated or broken in long elongate sections, otherwise remaining firmly attached, if detaching then usually doing so along transverse cracks, and peeling inwards and upwards to leave distinct layers of elongate to coarsely tabular, chartaceous, flakes that are centrally attached, with sinuous margins; upper bark surface usually with much secondary peeling, these flakes similar to primary flakes but more distinctly chartaceous, smaller, narrowly elongate with widely sinuous margins; bark usually crumbling readily in hand, and breaking readily if pulled hard into numerous, small, ± tabular to distinctly irregular flakes. *Branches* depending on growth habit and situation, numerous, initially arising from close to or at trunk base but as plants mature basally thinning such that branches are retained only in the upper half of the tree; usually rather slender, initially ascending but soon spreading, with apices often distinctly pendulous, branch bases mostly clean, sometimes congested by partially decorticated bark; branchlets numerous, usually rather slender, gracile, initially ascending, soon spreading, terminal growth erect or pendulous; initially bright green or bronze green, sometimes red, ± quadrangular to subterete, glabrescent; new growth sericeous, indumentum initially copious, soon sparse, deciduous, hairs divergent 0.02–0.05 mm long, hyaline to translucent (appearing silvery-white when young maturing silver-grey), apices straight not curled or curved; leaves of branchlets densely crowded along stems and brachyblasts; brachyblasts usually closely spaced, though in vigorous new growth they are sometimes quite widely spaced. *Vegetative buds* inconspicuous, usually obscured from view by surrounding leaves; at resting stage 0.5–0.8 mm diam. narrowly to broadly ovoid; scales often persistent; (0.4–)1.1 mm long, dark red-brown, broadly ovate, ovate-lanceolate grading through to lanceolate, rostrate to cuspidate; midrib strongly keeled, with one row of 4–8 oil glands on either side of midrib; scales glabrous except for the margins and apex; these densely invested in white, silky hairs. *Leaves* homophyllous; sessile, lamina surfaces glabrous, margins and the basal, adaxial portion of the midrib hairy (especially on young leaves); densely crowded (particularly toward apices) along branchlets and brachyblasts; initially obliquely ascending, mostly suberect to spreading when mature; lamina (4.0–)13.5(–25.0) × (0.5–)1.1(–1.8) mm, bright green to yellow-green, rarely dark green, adaxial surface often glossy when fresh, drying dull, abaxial surface paler; lamina linear, linear-lanceolate, to narrowly lanceolate, straight or with distal quarter weakly recurved, apex acute, sometimes cuspidate, base attenuate; adaxial lamina surface flat to weakly concave, without obvious oil glands, midrib very slightly raised near base, otherwise scarcely evident, basal portion finely and sparsely covered with deciduous, antrorse-appressed sericeous hairs; abaxial surface flat to weakly convex, glandular punctate, oil glands up to 200; midrib glabrous, usually not evident when fresh, sometimes weakly raised just near base, often not evident when dry but sometimes discernible as a slight groove for entire length; lamina margins initially very finely sericeous, becoming glabrate or glabrous; hairs when present antrorse-appressed, forming a fine, often discontinuous band failing just short of lamina apex, otherwise decurrent along leaf bases. *Perules* deciduous or persistent, (0.6–)0.8(–1.5) mm, initially squamiform, becoming foliose toward first flower, dark red-brown, broadly ovate, ovate-lanceolate grading through to lanceolate, rostrate to cuspidate; midrib strongly keeled, with one row of 4–12 oil glands on either side of midrib; glabrous except for the margins and apex; these densely invested in white, sericeous hairs. *Inflorescence* mostly a compact corymbiform to shortly elongate (3–)8(–15)-flowered botryum up to 60 mm long; usually on brachyblasts with the terminal shoot corymbiform or extending as a slightly longer (up to 80 mm long) 6–15-flowered, elongate botryum with flowers usually crowded, terminal portion usually bearing undeveloped flowers and active vegetative growth. Inflorescence axis densely invested with short, weakly divergent silky hairs. *Pherophylls* foliose ± persistent, 1 per flower; lamina (3.0–)6.7(–7.8) × (0.9–)1.1(–1.4) mm, leaf-like pherophylls bright green (rarely dark green) elliptic, lanceolate to narrowly lanceolate, apex acute, base attenuate; adaxial surface weakly concave to flat, oil glands scarcely evident up to 10; midrib scarcely evident at base only, surface glabrous; abaxial surface weakly convex or flat, oil glands up to 30; midrib scarcely evident at base only, lamina margin glabrescent, hairs as for leaf margins. *Pedicels* (1.6–)2.7(–3.8) mm long at anthesis, usually elongating slightly after anthesis, terete, usually glabrous, very rarely sparsely covered with divergent to weakly sericeous hairs. *Flower buds* pyriform to narrowly obconic, apex of mature buds weakly domed to flat, calyx lobes distant, not touching. Fresh flowers when fully expanded (4.1–)6.3(–8.3) mm diam. *Hypanthium* (1.4–)2.1(–3.2) × (1.9–)2.9(–4.1) mm, with free portion (0.4–)0.6(–1.0) mm long, bright green, bronze-green or yellow-green mottled with red; sharply obconic, apex terminating in a usually dark pink or crimson chartaceous rim bearing five persistent suberect to spreading calyx lobes (rim usually drying dark maroon to maroon-black); external hypanthium surface smooth, glabrous (very rarely glabrescent with basal quarter finely and sparsely covered with minute weakly antrorse hairs); oil glands, conspicuous, ± colourless; ribs not evident when fresh, conspicuous (along with venation) when dry. Calyx lobes 5, suberect to spreading, subcoriaceous, (0.4–)0.7(–1.0) × (0.4–)0.8(–1.0) mm, persistent, orbicular, obtuse to broadly deltoid, red-green, pink or crimson, keel not evident in fresh material, becoming prominent when dried, oil glands conspicuous, ± colourless, margins glabrous or finely ciliate; cilia white. Receptacle green or pink at anthesis, darkening to crimson or dark magenta after fertilisation. *Petals* 5, (1.4–)2.2(–2.6) × (1.5–)2.2(–2.9) mm, white (often drying yellow), orbicular, suborbicular to narrowly ovate, spreading, apex rounded, margins often incurved, entire or very finely denticulate, oil glands usually not evident when fresh, ± colourless. *Stamens* (10–)18–24(–34) in 1–2 weakly defined whorls, arising from receptacle rim, filaments white. Antipetalous stamens (2–)3(–5), antisepalous 2–3(–4). Antipetalous stamens outcurved usually with distal portion slightly incurved, on filaments 1.6–2.8 mm long, inner stamens if present, confined to the bases of the outermost antipetalous pair, 0.8–1.2 mm, incurved. Antisepalous stamens shorter than outermost antipetalous stamens, 0.6–1.2 mm, weakly to strongly incurved, rarely erect or outcurved, often in mixtures of both. Anthers dorsifixed, 0.35–0.48 × 0.16–0.24 mm, broadly ellipsoid, latrorse. Pollen white (14.1–)14.6(–17.3) μm. Anther connective gland prominent, pink or pinkish-orange when fresh, drying red to orange, ± spheroidal to pyriform, ± immersed to half of length between anthers, ± coarsely papillate. *Ovary* (4–)5 locular, each with 16–21(–24) ovules in two rows on each placental lobe. Style 1.5–2.2 mm long at anthesis, elongating slightly after anthesis, white, rarely basally flushed pink; stigma capitate, about 1¼ × the style diam., flat, cream or white, flushing pink after anthesis, surface very finely granular-papillate. *Fruits* rarely persistent, (1.9–)2.7(–3.4) × (1.8–)2.8(–3.9) mm, glabrous, initially dark green to reddish-green, maturing brown to grey-brown to grey-black; in all types fading with age to pale greyish-white, cupular, barrel-shaped, shortly cylindrical to hemispherical, calyx valves usually erect with the apices incurved, splits concealed by dried, erect, free portion of hypanthium. *Seeds* 1.00(–1.05) × 0.32(–0.50) mm, usually curved near apex, laterally compressed, 2–3-angled with convex to flattened faces, apex rounded to subacute; base oblique, ± flattened; testa semi-glossy, orange-brown to dark brown, obovoid, oblong, oblong-ellipsoid, or cylindrical and ± curved, surface coarsely reticulate. FL: (Nov–)Dec–Jan(–Mar). FT: Feb–Apr(–Aug). Chromosome Number *n* = 11_II_, 2*n* = 22 (see de Lange and Murray 2004).

#### Representative specimens

**(99 sheets seen).** **New Zealand (South Island).** Wakamarama Range, Knuckle Hill, P. J. de Lange 4953, 10 Jan 2001, (AK 253376, Duplicate: AD, CHR); Whanganui Inlet, P. J. de Lange 4952, 10 Jan 2001, (AK 289162, Duplicate: AD); Wakamarama Range, Mt Burnett, South Peak, P. J. de Lange 4946, 10 Jan 2001, (AK 289159, Duplicate: AD, MEL); Aorere River, H. Talbot s.n., 15 Dec 1959, (CHR 300594); Golden Bay, Wainui Inlet, Takapou Point, P. J. de Lange 4994, 12 Jan 2001, (AK 289173, Duplicate: AD); Waitui Stream, upper Takaka, W. D. Burke s.n., 23 Nov 1979, (WELTU 13480); Kahurangi National Park, Mt Peel, near source of Trilobite Creek, P. J. de Lange 6329 & G. M. Crowcroft, 16 Jan 2001, (AK 289179); Abel Tasman National Park, Astrolabe Roadstead, Adele Island; P. J. de Lange 5001, 13 Jan 2001, (AK 253380, Duplicate: AD, P); Moutere E.D., Upper Moutere, J. F. F. Hobbs s.n., 18 Aug 2002, (NZFRI 25012); Wangapeka River Road, Rolling River, P. J. de Lange 5082, 21 Jan 2001, (AK 253381, Duplicate: AD); Hope River, Sandy Creek, P. J. de Lange 5085, 21 Jan 2001, (AK 287548, Duplicate: AD); Ngatimoti, Haycock's Hill, R. Wilson s.n., Nov 1964, (OTA 13619); Bryant Range, Hackett Creek, near Whispering Falls, P. J. de Lange 5031 & G. M. Crowcroft, 17 Jan 2001, (AK 286127, Duplicate: AD); Golden Downs, Wakefield, Faulkner’s Bush, P. J. de Lange 5073, 21 Jan 2001, (AK 289193, Duplicate: AD); Nelson Lakes National Park, Speargrass Creek, Speargrass Track, P. J. de Lange 5066, 23 Jan 2001, (AK 289190, Duplicate: AD); Owen Valley East Road, Carrol Creek, P. J. de Lange 5136, 21 Jan 2001, (AK 289201, Duplicate: AD); Buller River, near Owen Junction, P. J. de Lange 5137, 21 Jan 2001, (AK 289202, Duplicate: AD, CHR); Lower Buller Gorge, Buller River, P. J. de Lange 4787 & P. I. Knightbridge, 7 Dec 2000, (AK 288294, Duplicate: AD); D'Urville Island, north of Attempt Hill, P. J. de Lange 5053 & G. M. Crowcroft, 19 Jan 2001, (AK 289185); Pelorus Sound, Mahakipawa Inlet, Moenui, P. J. de Lange 4904, 8 Jan 2001, (AK 288400, Duplicate: AD); Mt Freeth, Queen Charlotte Sound, W. R. B. Oliver s.n., 5 Apr 1931, (WELT SP029535); Cloudy Bay, Rarangi - Port Underwood Road, top of Rarangi Zig Zag Track, P. J. de Lange 5116, 23 Jan 2001, (AK 289198, Duplicate: AD).

#### Distribution

**Figure 7.**
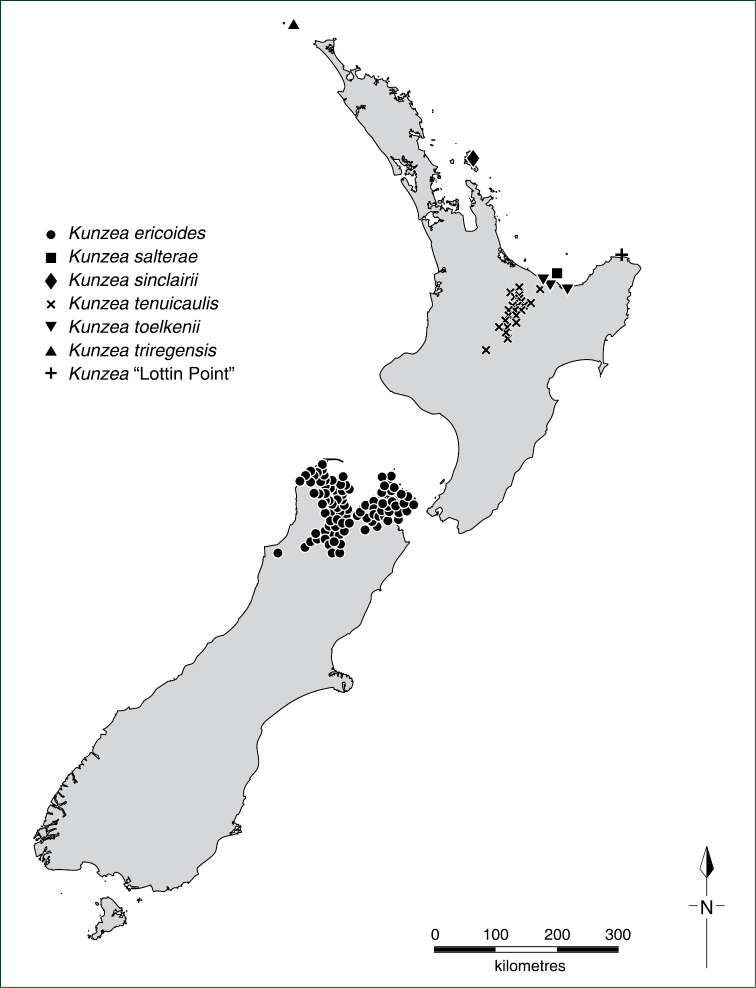
Distribution of *Kunzea ericoides*, *Kunzea salterae*, *Kunzea sinclairii*, *Kunzea tenuicaulis*, *Kunzea toelkenii*, *Kunzea triregensis* and *Kunzea* “Lottin Point”.

**(Fig. 7).** Endemic, New Zealand, South Island (sea level–1600 m a.s.l.). *Kunzea ericoides* is endemic to the northern South Island north of and including the Wairau and Buller River catchments.

#### Recognition.

*Kunzea ericoides* can be easily recognised by its glabrescent, often terminally pendent branchlets (Fig. 6D). The branchlet hairs (Fig. 5A–D, G) are deciduous, consistently divergent, short (up to 0.05 mm), and usually sparse. The leaves of *Kunzea ericoides* are typically bright green, linear, linear-lanceolate, to narrowly lanceolate, glabrate and mostly crowded on brachyblasts (Fig. 6H). Often the brachyblasts are widely spaced along the branchlets. The hypanthium of *Kunzea ericoides* is sharply obconic, glabrous (very rarely sparsely hairy), and copiously dotted with conspicuous colourless oil glands. The fruits of *Kunzea ericoides* are usually glabrous (very rarely sparsely hairy near the base) and are mostly cupular, barrel-shaped, or shortly cylindrical in shape (Fig. 4N). In growth habit *Kunzea ericoides* varies from a trailing, decumbent shrub on ultramafic substrates to a tall tree (up to 18 m tall) but, irrespective of stature, it typically retains a somewhat spreading to slightly pendulous openly branching habit (Fig. 6B–D). Cytologically *Kunzea ericoides* has a similar chromosome complement to *Kunzea amathicola*, *Kunzea triregensis* and *Kunzea sinclairii* (de Lange and Murray 2004). Molecular evidence (rDNA ITS) grouped *Kunzea ericoides* with *Kunzea linearis*, *Kunzea triregensis*, *Kunzea robusta* (all samples), *Kunzea serotina*, and *Kunzea toelkenii* (Table 2; see also de Lange 2007; de Lange et al. 2010). However, the rDNA ETS sequence of *Kunzea ericoides* had one unique cytosine/thiamine mix, shared with *Kunzea linearis* a cytosine, and, with *Kunzea linearis*, *Kunzea robusta* (Mt Egmont samples only), *Kunzea salterae*, *Kunzea serotina* and *Kunzea toelkenii*, a guanine/cytosine mix (Table 2; see also de Lange 2007).

*Kunzea ericoides* is sympatric with *Kunzea amathicola*, *Kunzea robusta* and *Kunzea serotina* and also forms hybrids with them. Of the three, *Kunzea ericoides* is most frequently sympatric with *Kunzea robusta*. Both species naturally meet in the Marlborough Sounds where *Kunzea robusta*, though mainly coastal, is locally common (another occurrence at Totaranui, Abel Tasman National Park, results from the naturalisation from plantings of *Kunzea robusta* within a camp ground). On the south-eastern coast of D’Urville Island and from about the Tory Channel south toward Rarangi, both species are abundant. The form of *Kunzea robusta* present in the north-eastern South Island is easily distinguished from *Kunzea ericoides* as it has extremely hairy branchlets, with the long, silky, antrorse-appressed hairs easily seen by the naked eye or with a 10× hand lens. Aside from branchlet hairs both species have different foliage colour and leaf shape; *Kunzea robusta* mostly has dark green leaves that are oblanceolate, broadly oblanceolate to broadly lanceolate with irregularly long sericeous hairs on the margins. *Kunzea ericoides* has bright green, linear-lanceolate leaves whose margins are sparsely hairy trending toward glabrous (Fig. 4D–H). The hypanthia of both species also differ. In *Kunzea robusta* the hypanthium is broadly obconic to almost turbinate with the external faces copiously covered in short, antrorse-appressed, sericeous hairs. In *Kunzea ericoides* the hypanthium is glabrescent to completely glabrous, and sharply obconic. The fruits show much the same differences, *Kunzea robusta* being broadly obconic to turbinate with the external faces distinctly hairy and *Kunzea ericoides* mostly cupular, barrel-shaped to shortly cylindrical or sometimes hemispherical and glabrous.

In the north-western part of its range *Kunzea ericoides* is frequently sympatric with *Kunzea amathicola* following a line running from the Aorere River west to the Paturau River. Again, both species are easily distinguished due to the very different branchlet indumentum. *Kunzea amathicola* is distinctly more hairy, with the branchlet hairs long, sericeous, and antrorse-appressed, a marked contrast to the glabrescent branchlets, and short divergent hairs diagnostic of *Kunzea ericoides*. The leaves, inflorescence type, flowers and fruits of both species are also very different and further distinctions are given in more detail under *Kunzea amathicola* and in Table 1. However, two critical characters are briefly mentioned here. The inflorescences of *Kunzea amathicola* are always elongate botrya, while those of *Kunzea ericoides* are usually corymbiform (Fig. 4A) though mixtures of corymbiform and shortly elongate botrya are sometimes produced in shaded, stressed or late summer flowering specimens. The pherophylls of *Kunzea ericoides* are always in mixtures of squamiform and foliose, with the foliose ones ranging from elliptic, lanceolate to narrowly lanceolate, they are never oblong to oblong-obovate like those of *Kunzea amathicola*. The other major difference is that the leaf margins of *Kunzea amathicola* are covered in a conspicuous thick, continuous band of long antrorse-appressed, sericeous hairs which reaches the leaf apex. In *Kunzea ericoides* although marginal hairs are usually present these are hardly conspicuous, tend to be shed as the leaf matures, and they normally form a rather thin, often discontinuous band which does not reach the leaf apex (Fig. 4F–G).

Along the Buller River, at the northern end of Nelson Lakes and in the upper Wairau, *Kunzea ericoides* is occasionally syntopic with *Kunzea serotina*. Both species are easily distinguished (Table 1), especially as *Kunzea serotina* has such a distinctive bark type and a pyramidal, columnar, growth habit with obliquely ascending, fastigiate branches. Further, the usual leaf shape of *Kunzea serotina* is shortly linear-oblanceolate to obovate rather than the longer linear-lanceolate condition of *Kunzea ericoides*. However, on the ultramafics of the Red Hills and on Mt Dun, *Kunzea ericoides* tends to be more stunted and in these forms the leaves tend to be half their usual size. While this condition does not appear to have a genetic basis, field distinction can become confusing, especially when such forms grow amongst similarly dwarfed *Kunzea serotina* specimens. In these situations the branchlet hairs are diagnostic: in *Kunzea serotina* they are copious, up to 0.08 mm long with curved or curled apices, whereas the branchlets of *Kunzea ericoides* are glabrescent, with the hairs up to 0.05 mm long and with straight apices (Fig. 5B, D). Flowering material readily separates both species because *Kunzea serotina* has deciduous, mostly spathulate to spathulate-orbicular, rather than persistent, elliptic, lanceolate to narrowly lanceolate pherophylls, and the oil glands in the petals are yellow, rather than ± colourless.

#### Ecology.

Although much has been written about the ecology of *Kunzea ericoides* *sens. lat.* (Burrell 1965; Burrows 1973; Wardle 1991; Smale 1994; Smale et al. 1995) most of that information is based on *Kunzea amathicola*, *Kunzea linearis*, *Kunzea robusta*, *Kunzea salterae*, *Kunzea serotina*, and *Kunzea toelkenii*. My observations suggest that *Kunzea ericoides* is ecologically most similar to *Kunzea robusta* in that it can be found in a wide variety of habitats from early stage seral shrubland (Fig. 6A) through to tall forest, and it has a similar growth habit. However, it attains a much higher altitudinal limit than *Kunzea robusta*, having been collected during this study at 1600 m a.s.l. on the northern slopes of Mt Peel, Kahurangi National Park, north-west Nelson. In montane areas it is frequently found within north-facing canopy gaps developed within montane black beech (*Fuscospora solandri* (Hook.f.) Heenan et Smissen) and mountain beech (*Fuscospora cliffortioides* (Hook.f.) Heenan et Smissen) forests, often close to the upper tree limit for these species in the Richmond Range and parts of north-west Nelson. The species is also commonly encountered in coastal forest, though rarely on sand dunes, and it can be a conspicuous tree of lowland areas, especially along river flats and on outwash gravels within the Waimea Plain and on sections of the northern bank of the Buller River.

No obvious substrate requirement is evident. It appears to avoid permanently waterlogged soils and peat and it is scarce from wetlands, though it may at times be common in the vegetation bordering these habitats. Free draining soils and recent alluvium is commonly colonised, as is open ground within lowland to upper montane forests. On the extensive karstfield of north-west Nelson *Kunzea ericoides* is often prominent, especially in places where there has been a history of logging, mining, farming or frequent fires. *Kunzea ericoides* is also a common component of the ultramafic areas of D’Urville Island, Mt Dun, the Red Hills and upper Takaka.

*Kunzea ericoides* is an important primary tree coloniser of formerly cleared ground in many parts of its range. In these situations, perhaps more than any other, the dense leaf litter produced by the often closely growing trees is ideal for a wide range of terrestrial orchids (especially of the genera *Acianthus* R.Br., *Caladenia* R.Br., *Corybas* Salisb., *Gastrodia* R.Br., and *Pterostylis* R.Br.), and fungi (see McKenzie et al. 2006), while the bark is often colonised by mosses such as *Macromitrium* Brid. spp., *Cryphaea tenella* Müll.Hal., *Distichophyllum pulchellum* (Hampe) Mitt., and *Weymouthia cochlearifolia* (Schwägr.) Dix, and by liverworts of the genera *Frullania* Raddi, *Lejeunea* Lib. and *Metzgeria* Raddi. *Kunzea ericoides* bark also supports a diverse array of lichens in the following genera; *Coccocarpia* Pers., *Heterodermia* Trevis., *Pannaria* Delise ex Bory, *Parmotrema* A.Massal., *Pseudocyphellaria* Vain., *Sticta* (Schreb.) Ach. and *Ramalina* Ach. The upper branches and branchlets are also frequently parasitised by the dwarf mistletoe *Korthalsella salicornioides* (A.Cunn.) Tiegh. and, less commonly, green mistletoe (*Ileostylus micranthus* (Hook.f.) Tiegh.). *Kunzea ericoides*-dominated shrubland and forest also provides an important and at times critical habitat for a range of geckos in the endemic genera *Mokopirirakau* Nielsen et al. 2011, *Naultinus* (Gray, 1842), and *Woodworthia* Garman 1901 (R. Hitchmough pers. comm.).

#### Hybridism.

*Kunzea ericoides* naturally hybridises only with *Kunzea amathicola*, *Kunzea robusta* and *Kunzea serotina*. The most common of these hybrids is *Kunzea ericoides* × *Kunzea robusta*, which is abundant on the south-eastern side of D’Urville Island (especially around Katherine Bay), and along the eastern side of the Marlborough Sounds, particularly east of Picton and south of the Tory Channel to about Rarangi. These areas comprise some of the first sites of European settlement in New Zealand, prior to which they were once heavily populated by Maori (Buick 1976; de Lange et al. 2005). Both cultures extensively modified these landscapes and even in modern times much of this area has been repeatedly burned. These are ideal conditions for hybridism, and in the case of both *Kunzea ericoides* and *Kunzea robusta* it has helped create and maintain an extensive introgressed hybrid swarm.

Recognition of *Kunzea ericoides* × *Kunzea robusta* in the field and in the herbarium is generally easy because both species have such different branchlet hairs. Hybrid plants can usually be recognised by the presence of mixtures of long, silky, antrorse-appressed and very short divergent hairs. Further, *Kunzea ericoides* × *Kunzea robusta* hybrids often have a distinctly pale glaucous sheen to their usually yellow-green leaves. Similar plants were produced in the experimental F_1_ crosses *Kunzea ericoides*^♀^ × *Kunzea robusta*^♂^ and *Kunzea robusta*^♀^ × *Kunzea ericoides*^♂^ and these proved to be fully fertile (de Lange et al. 2005). In most instances the hybrid can be easily recognised but because hybrids are fully fertile, an often bewildering array of introgressants can be found in sites of prolonged disturbance, in some cases almost to the exclusion of either parent. Recognition of hybrids within such populations can at times be difficult. For example, in those hybrids trending toward *Kunzea ericoides* there is a progressive loss of the long, antrorse-appressed sericeous branchlet hairs diagnostic of *Kunzea robusta*. In some examples where the branchlet hairs are virtually dominated by short divergent hairs, a hybrid ancestry may still be elucidated by diligent searching, particularly along the branchlet axis immediately opposite active leaf buds and emergent leaves where a few of the longer, antrorse-appressed sericeous type diagnostic of *Kunzea robusta* are usually retained as sparse patches. In pure *Kunzea ericoides* these are mostly all soon shed. One of the last *Kunzea robusta* traits to be lost is the presence of antrorse-appressed, silky hairs on the external surface of the hypanthia, allowing the hybrid origin of specimens in all other respects matching *Kunzea ericoides* to still be elucidated. Branchlet hairs, or rather their relative abundance also serves to help distinguish introgressants trending toward pure *Kunzea robusta* such that apparently pure specimens of *Kunzea robusta* prove on careful examination of the branchlets to be glabrescent and completely dominated by short, divergent hairs. For *Kunzea ericoides* at least, the branchlet hairs seem to be a long-lived trait traceable in any hybrid swarm it may form. In north-west Nelson from about Golden Bay and the Whanganui inlet north, *Kunzea ericoides* and *Kunzea amathicola* are commonly syntopic and hybrids are frequent in the more heavily modified lowlands and roadsides of this area. Like the *Kunzea ericoides* × *Kunzea robusta* hybrid, the branchlet indumentum enables recognition because it comprises mixtures of both hair types. The inflorescences of *Kunzea amathicola* × *Kunzea ericoides* hybrids are also in mixtures of mostly elongate (typical of *Kunzea amathicola*) and some subcorymbiform to corymbiform botrya (typical of *Kunzea ericoides*). The pherophylls tend to be rather variable in length and size but they are mostly lanceolate to linear-lanceolate like *Kunzea ericoides*. The leaf margins of *Kunzea amathicola* × *Kunzea ericoides* are also distinctive. Initially they are hairy but, like *Kunzea ericoides*, the hairs are progressively shed during leaf maturation. Further, hairs rarely (if ever) meet at the leaf apex.

*Kunzea ericoides* × *Kunzea serotina* is much less common, because the ranges of both species rarely overlap. This hybrid is mostly confined to the upper Buller River and within the Barnicoat Range and Hackett areas of eastern Nelson where the usually montane *Kunzea serotina* extends down into lowland areas and so abuts the main habitats of *Kunzea ericoides*. These are also areas where road construction and maintenance has significantly altered the surrounding indigenous vegetation allowing these species to meet and hybridise. *Kunzea ericoides* × *Kunzea serotina* is easily recognised by the erect, somewhat open, sub-pyramidal growth habit, short, weakly fastigiate branchlets, and bright yellow-green, red-tinged leaves (all features of *Kunzea serotina*). However, as with *Kunzea ericoides*, the leaves tend to be linear-lanceolate, though sometimes quite broadly so. Branchlet indumentum is not particularly useful because both parent species have divergent hairs, though in most examples the hybrid tends to be, like *Kunzea serotina*, distinctly hairy. However, rare glabrescent examples suggest that introgressive hybridisation toward *Kunzea ericoides* is occurring at some sites. The pherophylls of *Kunzea ericoides* × *Kunzea serotina*, like those of *Kunzea serotina*, are not persistent but are mostly shed during flower maturation. Further, the pherophylls of the hybrid are pandurate to elliptic (never spathulate like *Kunzea serotina* or narrowly elliptic, lanceolate to narrowly lanceolate like those of *Kunzea ericoides*). The flowers of *Kunzea ericoides* × *Kunzea serotina* offer another point of recognition. Like *Kunzea serotina* they tend to have more obvious oil glands than is usual for *Kunzea ericoides*, and while those of *Kunzea ericoides* are normally colourless, those of the hybrid vary from colourless through to pale yellow, the latter of which is the usual condition of *Kunzea serotina*.

#### Vernacular names.

Although now universally known as ‘kanuka’, the name recorded for *Kunzea ericoides* from the Nelson area during Dumont d’Urville’s second voyage was ‘manuoea’ (Richard 1832). Based on other specimens lodged at P(!) and elsewhere this species was also known as ‘manuka’, ‘titire’ and ‘atitire’ (for a discussion on the names ‘titire’ and ‘atitire’ see *Kunzea robusta*).

#### Conservation status.

*Kunzea ericoides* is a widespread and abundant species throughout its northern South Island range and is lited as ‘Not Threatened’ by de Lange et al. (2013b).

### 2. Kunzea serotina

Taxon classificationPlantaeMyrtalesMyrtaceae

de Lange et Toelken sp. nov.

urn:lsid:ipni.org:names:77141728-1

A K. ericoide habitu in arboribus juvenalis et juveni-adultis, erecto columnare fastigiato vel pyramidale, transformante in arboribus veterrimis habitu laxe ramoso divergente apice applanata; cortice maturo crisparenti circinanti et decorticanti promte partibus parvis irregularibus chartaceis, habitu fastigiato, pherophyllis spathulatis, hypanthio urceolato vel campanulato, ovariis saepe trilocularibus, glandibus oleosis flavis petalorum differt.

#### Holotype

**Figure 8.**
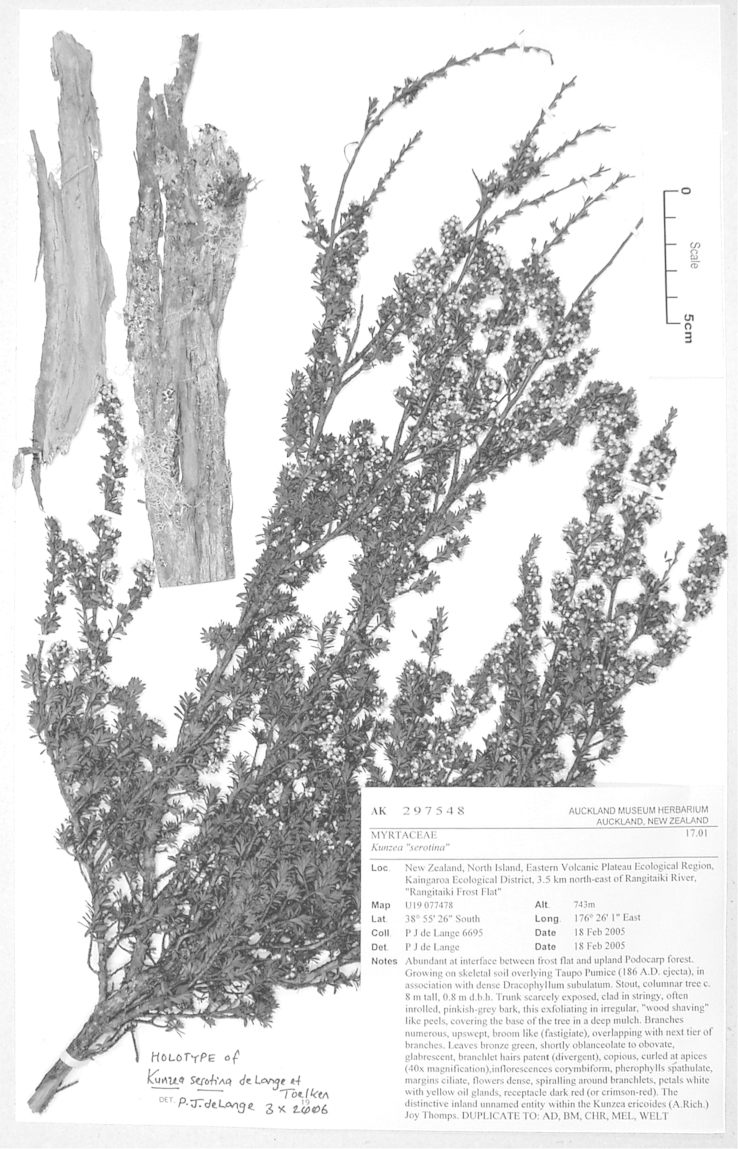
Holotype of *Kunzea serotina* de Lange et Toelken (*P. J. de Lange 6695*, AK 297548).

**(Fig. 8).** **New Zealand:** North Island, 3.5 km north-east of Rangitaiki River, 38°55'26"S, 176°26'1"E, 743 m a.s.l. ‘Abundant at interface between frost flat and upland Podocarp forest. Growing in skeletal soil overlying Taupo Pumice (186 A.D. ejecta), in association with dense *Dracophyllum subulatum*’. *P. J. de Lange 6695*, 18 Feb 2005, AK 297548! Isotypes: AD! BM! CHR! MEL! WELT!

#### Etymology.

The specific epithet *serotina* refers to the later flowering habit of the species (peaking January–February) when compared to *Kunzea robusta* and *Kunzea tenuicaulis*, earlier flowering species (peaking October–December) with which *Kunzea serotina* is sympatric in the North Island.

#### Description

**Figure 9.**
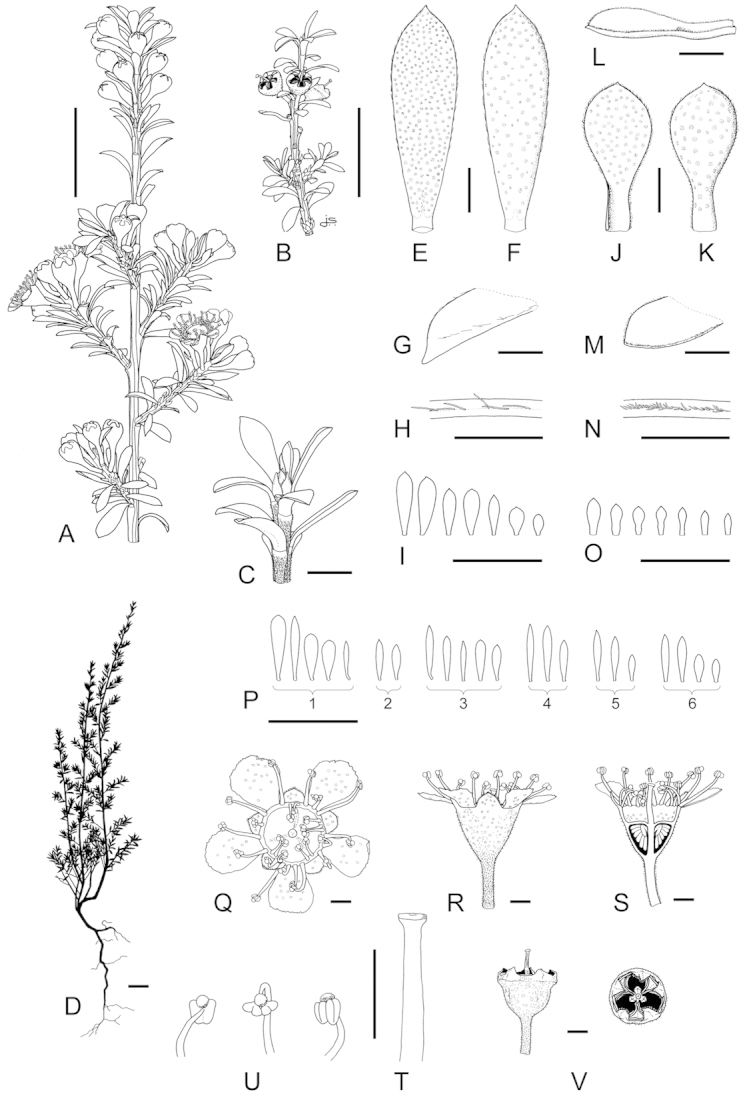
Distinguishing features of *Kunzea serotina*. **A** Flowering branchlet (ex cult. AK 282217) **B** Fruiting branchlet (ex cult. AK 282217) **C** Vegetative bud and branchlet indumentum (ex cult. AK 282217) **D** Seedling (AK 286184) **E** Adaxial leaf surface (ex cult. AK 282217) **F** Abaxial leaf surface (ex cult. AK 282217) **G** Adaxial leaf apex (ex cult. AK 282217) **H** Leaf margin indumentum (ex cult. AK 282217) **I** Leaf variation within the same individual (ex cult. AK 282217) **J** Adaxial surface of pherophyll (ex cult. AK 282217) **K** Abaxial surface of pherophyll (ex cult. AK 282217) **L** Side view of pherophyll (ex cult. AK 282217) **M** Adaxial pherophylls apex (ex cult. AK 282217) **N** Pherophyll margin indumentum (ex cult. AK 282217) **O** Pherophylls variation within the same individual (ex cult. AK 282217) **P** Leaf variation: (**P1**) Mangatoetoenui Stream (AK 288142), (**P2**) Te Porere Redoubt (AK 288140), (**P3**) Medbury Scientific Reserve, (AK 288543), (**P4**) Maruia Springs (AK 289968), (**P5**) Lewis Pass (AK 287555), (**P6**) Bendigo Scenic Reserve (AK 289978) **Q** Flower (top view) (ex cult. AK 282217) **R** Flower and hypanthium (side view) (ex cult. AK 282217) **S** Flower cross section showing anther, style and ovules (ex cult. AK 282217) **T** Style and stigma (ex cult. AK 282217) **U** Stamens (ex cult. AK 282217) **V** Dehisced fruit (ex cult. AK 282217). Scale bars: (**A, B, D, I, O, P**) 10 mm; (**C, E–G, J–M, Q–V**) 1 mm; (H, N) 0.5 mm.

**Figure 10.**
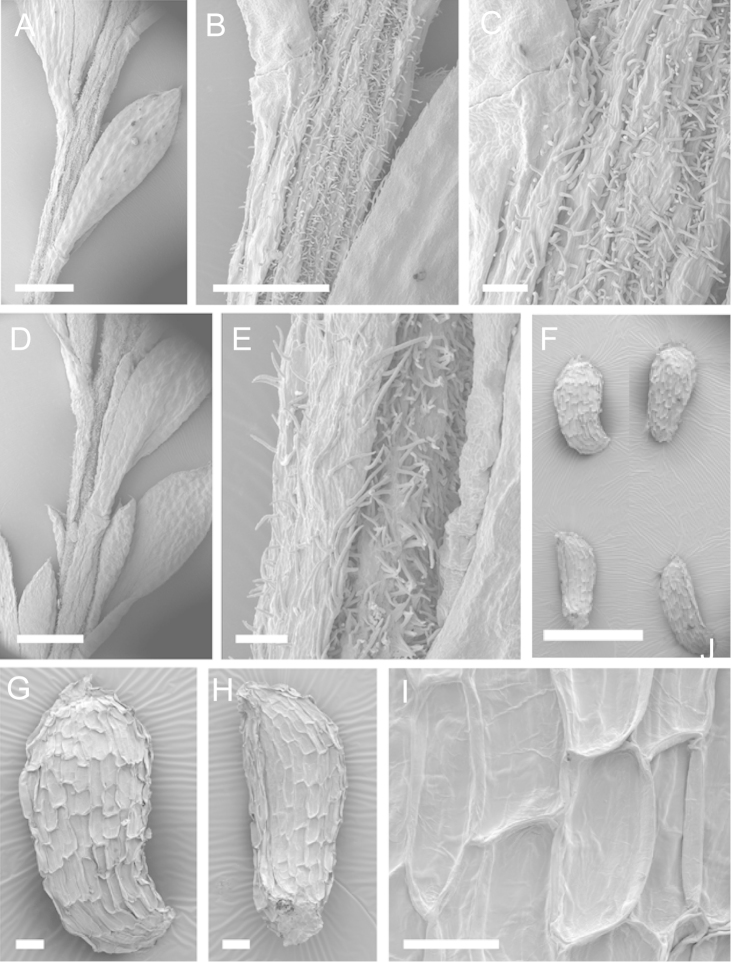
Scanning Electron Micrographs of *Kunzea serotina*. **A–E** Branchlet indumentum (AK 285217) **F–I** Seeds (AK 289978) **I** Testa surface showing reticulum (AK 289978). Scale bars: (**A, D, G**) 1 mm; (**B**) 500 μm; (**C, E, F, H**) 100 μm; (**I**) 50 μm.

**Figure 11.**
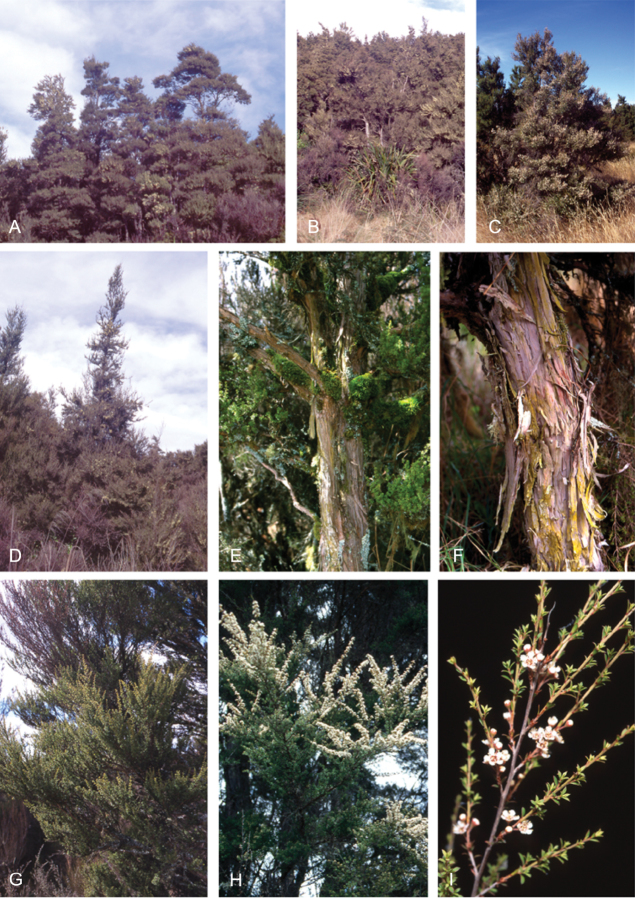
*Kunzea serotina*. **A** *Kunzea serotina* trees on ridge line, North Island, Kaweka Range, upper Makahikatoa Stream (photo: *P. J. de Lange*) **B** *Kunzea serotina* shrubs and trees in frost flat, North Island, Hauhangaroa Range, Moerangi (photo: *P. J. de Lange*) **C** *Kunzea serotina* tree, South Island, Medbury Scientific Reserve (photo: *P. J. de Lange*) **D** *Kunzea serotina* tree showing columnar growth habit and fastigiate, obliquely ascending branches, North Island, Rangitaiki Frost Flats, near Iwitahi (photo: *P. J. de Lange*) **E** Upper trunk bark and branching pattern, North Island, Rangitaiki Frost Flats, near Iwitahi (photo: *P. J. de Lange*) **F** Lower trunk bark, North Island, Rangitaiki Frost Flats, near Iwitahi (photo: *P. J. de Lange*) **G** *Kunzea serotina* showing characteristic branching pattern of the species, North Island, Rangitaiki Frost Flats, near Iwitahi (photo: *P. J. de Lange*) **H** Flowering branches, North Island, Tongariro Forest, Te Porere redoubt, (photo: *P. J. de Lange*) **I** Close up of flowering branchlet, North Island, Tongariro National Park, Oturere Stream (photo: *J. E. Braggins*).

**(Figs 9, 10, 11).** *Growth habit* erect, initially columnar to tightly pyramidal, fastigiate, densely branched shrubs or trees 3–20 × 2–4 m developing with time into less densely branched, open pyramidal crowns; in very stable conditions growth habit changing in aged specimens to a more openly branched, flat-topped, divergent crown, with branches restricted to upper half to third of trunk. *Trunk* usually single, very rarely 2–3 arising from base, 0.10–0.45(–0.86) m d.b.h., often basally buttressed, with basal portion of trunk covered in numerous, curled, chartaceous bark remnants. *Bark* early bark chartaceous, greyish-white to pinkish-white, ± elongate, initially with few transverse cracks but soon becoming heavily cracked (especially on branch flanges and decurrent leaf bases), often crumpled, soon detaching as in-rolled, curled, sinuous, irregular pieces, with ± frayed margins, detached pieces often congregating in branch forks and forming deep drifts at trunk base; old bark similar though more distinctly chartaceous-corky, upper surface with much secondary peeling, transverse cracking and crumpling; detaching readily, with flakes often hanging in loose curled masses beneath main branch forks and along trunk, margins rather irregular, sinuous, very rarely somewhat tabular; early and old bark flakes crumbling readily in hand. *Branches* numerous, usually arising at or near trunk base; short and stout, obliquely ascending, fastigiate; branchlets numerous, very leafy with many brachyblasts, quadrangular, indumentum copious, sericeous; persistent, divergent 0.05–0.06(–0.08) μm, hyaline to translucent (appearing white when young maturing grey) apices curved or slightly curled. *Vegetative buds* usually inconspicuous and obscured by surrounding foliage; at resting stage 0.2–0.5(–1.3) mm diam.; scales scarious, deciduous, (0.3–)0.8(–1.3) mm long, yellow-brown to red-brown, ovate, ovate-deltoid to broadly deltoid-rostrate; midrib prominent, strongly keeled in upper half, prolonged to long cuspidate tip, lateral veins absent, oil glands usually absent, upper half of scale margins, keel, and keel apex ciliate. *Leaves* heterophyllous; seedling, subadult leaves and those of reversion shoots, spreading to patent; lamina (0.8–)5.2(–7.8) × (0.6–)0.8(–1.2) mm, red-green or pale green suffused with red, rarely bright green, linear-lanceolate to lanceolate; flat or involute, apex acute to obtuse, finely cuspidate; adult leaves usually densely aggregated along brachyblasts of branchlets, initially obliquely ascending to suberect, spreading; lamina (2.0–)3.7(–6.3) × (0.8–)1.1(–1.8) mm, dark glossy green or bronze-green, margins and base often flushed red, linear-oblanceolate, oblanceolate to obovate; strongly recurved from about half of total length, apex initially acute to subacute, maturing obtuse to rounded, often cuspidate; base attenuate; adaxial surface concave or flat, glandular punctate; oil glands up to 780, more evident when dry, midrib slightly raised near base, otherwise not evident for rest of length, glabrous, very rarely with fine antrorse hairs near base; abaxial surface convex, glandular punctate, oil glands up to 180, more evident when dry; midrib flush with surface or depressed, glabrous, very rarely with a fine covering of silky, antrorse-appressed, antrorse to patent hairs near base; lamina margin sparsely hairy, hairs finely silky, flexuose, appressed to spreading, antrorse to subantrorse up to 0.08 mm long, hyaline to translucent, appearing as white to naked eye, aligned in one usually interrupted row failing well short of cuspidate leaf apex. *Perules* scarious, persistent, (0.3–)0.6(–1.0) mm; basal ones amber-brown to dark brown, broadly ovate, ovate-oblong, ± rostrate, apex acute, margins flat to involute especially in upper third, midrib weakly keeled, usually prolonged as a very short, deciduous, cuspidate apex, with one row of 4–12 oil glands on each side of midrib, glabrous except for finely ciliate margin and apex; intermediate perules deciduous, chartaceous, (0.2–)0.6(–0.8) mm long, initially pale brown to orange, upper perules usually pinkish-white when fresh, drying amber-brown to amber-orange, ovate to ovate-oblong, apex obtuse often appearing acute due to apical infolding, ± cuspidate, glabrous except for sparsely ciliate margin, weakly keeled, keel ± prolonged. *Inflorescence* a compact (1–)8(–12)-flowered corymbiform botryum up to 25 mm long, mostly borne on alternate, distinctly spiralled, basally densely leafy brachyblasts up to 15 mm long, each often bearing a terminal tuft of pherophylls and emergent leaves at anthesis; brachyblasts near branchlet apex usually subopposite; inflorescences at the ultimate branchlet terminus, uncommon, if present then often rather elongated and bearing well developed terminal vegetative growth. Inflorescence axis densely invested with divergent hairs. *Pherophylls* deciduous (falling very early), mostly foliose (rarely squamiform), 0.9–2.5 mm long, green to bronze-green, spathulate, spathulate-orbicular, rarely pandurate or lanceolate, margins and apex finely ciliate, grading into leaves at inflorescence axis apex. *Pedicels* (3.0–)3.5(–4.8) mm long at anthesis, usually elongating slightly after anthesis, terete, copiously invested in short, divergent to subantrorse, silky hairs. *Flower buds* clavate to pyriform, apex flat to weakly domed prior to bud burst with calyx valves not or scarcely meeting. Fresh flowers when fully expanded (2.8–)5.2(–8.8) mm diam. *Hypanthium* (1.6–)2.0(–3.4) × (1.5–)1.9(–3.8) mm, with free portion 0.4–0.8 mm long, dark green or red-green, if green then basally flushed with red when fresh, drying brown-green to red-brown; urceolate to campanulate terminating in a distinctly thicker rim bearing five persistent calyx lobes; surface smooth, copiously dotted with red oil glands, finely puberulent to ± glabrescent, with weakly defined ridges leading up to calyx lobes (these becoming more distinct upon drying); hairs if present, very short, divergent. *Calyx* lobes 5, upright (not spreading), firmly fleshy, (0.8–)1.0(–1.2) × (0.7–)1.0(–1.2) mm, persistent, ovate to broadly ovate, weakly keeled (keel evident only in dried specimens, where it is seen as a slightly thicker, often pale yellow, green or pink, central ridge), central portion of lobe pale green or yellow-green, with margins usually cream to pale pink, surface glandular punctate, oil glands usually pink in exposed situations otherwise ± colourless, glabrous except for distinctly spreading, ciliate margins. Receptacle usually pink at anthesis, consistently darkening to dark crimson magenta after fertilisation. *Petals* 5(–6), 1.4–1.6(–2.0) × 1.2–1.6(–2.0) mm, white, sometimes basally flushed pink, narrowly orbicular to broadly ovate or cuneate, apex obtuse to rounded, margins ± frayed to finely and irregularly toothed, oil glands yellow when fresh, when dried very pale yellow to colourless. *Stamens* 20–26(–38) in 1(–2) weakly defined whorls, arising from the receptacle rim, filaments white occasionally tinged rose-pink toward base. Antipetalous stamens (2–)3(–4), antisepalous stamens (1–)3(–6). Outermost antipetalous stamens usually weakly incurved, on filaments 0.7–1.9 mm long, inner stamen if present, 0.3–0.8 mm, strongly or weakly incurved, sometimes strongly outcurved, very rarely a further 1–2 strongly incurved stamens, 0.3–0.6 mm long, may be present at the base of the outermost antipetalous pair. Antisepalous stamens much shorter than outermost antipetalous stamens, 0.2–0.6 mm, usually incurved, rarely outcurved or in mixtures of both. Anthers dorsifixed, 0.04–0.06 × 0.02–0.04 mm, testiculate to ellipsoid, latrorse. Pollen white (11.1–)12.4(–13.7) μm. Anther connective gland prominent, orange often flushed with rose when fresh, drying dark orange-brown or purple, spheroidal, distinctly papillate. *Ovary* 3–4(–5) locular, each with 10–18(–23) ovules in two rows on each placental lobe. Style 0.6–0.8(–1.2) mm long at anthesis, often elongating slightly after anthesis, white; stigma capitate, scarcely wider than style, usually flat to very weakly domed along margins with a basal central depression, greenish-white, cream or pale pink, surface finely papillate. *Fruits* rarely persistent (1.2–)2.1(–3.0) × (1.2–)2.1(–3.4) mm, light brown to grey, finely hairy, urceolate to shortly-campanulate, rarely cupular, splits concealed by dried, suberect to erect, free portion of hypanthium and incurved calyx lobes. *Seeds* 0.60–0.90(–1.00) × 0.48–0.50(–0.60) mm, narrowly oblong, oblong, oblong-obovate, curved near apex, laterally compressed, 2–3-angled with convex to flattened faces, apex rounded to subacute; base cuneate to oblique, ± flattened; testa semi-glossy, orange-brown to dark brown, surface coarsely reticulate. FL: (Nov–)Jan–Feb(–May) FT: Jan–Dec. Chromosome Number *n* = 11_II_, 2*n* = 22 (see de Lange and Murray 2004).

#### Representative specimens

**(155 sheets seen):** **New Zealand (North Island).** Waikato River, Lake Waipapa, P. J. de Lange 1197, 6 Jan 1992, (AK 207188, Duplicates AD, CHR); Lake Rerewhakaaitu, W. D. Burke s.n., Jan 1960, (WELTU 3052); Te Kuiti, D. A. Franklin s.n., 1953, (WELTU 3053); Paeroa Range, Te Kopia Road, P. J. de Lange 4701, 16 Nov 2000, (AK 288232, Duplicate: AD); Pureora Forest, Link Road, Mihanga Stream headwaters, P. J. de Lange 4608 & R. O. Gardner, 18 Oct 2000, (AK 288230, Duplicate: AD); Waione Frost Flats, Waione Stream, P. J. de Lange 6440 & P. B. Cashmore, 12 Apr 2005, (AK 289785); Hauhangaroa Range, Waituhi Saddle, P. J. de Lange 4271 & P. de Lange, 31 Jan 2000, (AK 288110, Duplicate: AD); Rangitaiki, Otamatea Plains, A. J. Healy s.n., 19 Feb 1948, (CHR 62349); Puketitiri, Balls Clearing, A. P. Druce s.n., Mar 1973, (CHR 208702); Kaweka Ranges, south-west of Tutaekuri River Gorge, L. R. Perrie 3016, L. Shepherd & M. Shepherd, 14 Dec 2003, (AK 289511); Te Porere Redoubt Historic Reserve, above Waewaeru Stream, P. J. de Lange 4244 & N. J. D. Singers, 27 Jan 2000, (AK 288140, Duplicate: AD, CHR, NSW); Mt Ruapehu, Tukino, upper Mangatoetoenui Stream, P. J. de Lange 5142, 25 Jan 2001, (AK 288142); Kaimanawa Range, Waipahihi Road, P. J. de Lange 5981, 25 Jan 2001, (AK 286070, Duplicate: AD); Rangipo, Waikato Stream, P. J. de Lange 4247 & N. J. D. Singers, 27 Jan 2000, (AK 288133, Duplicates: AD, P); Moawhango – Napier Road, upper Makahikatoa Stream, P. J. de Lange 4382, 10 Aug 2000, (AK 286077, Duplicate: AD); North-West Ruahine Range, Pokopoko Stream, W. D. Burke s.n., 13 Dec 1966, (WELTU 3049); Near Rangiwahia, C. I. Pemberton Memorial Reserve, P. J. de Lange 4376, 10 Aug 2000, (AK 288234, Duplicate: AD); Puketoi Range, near Makuri, P. J. de Lange 6506, 28 May 2001, (AK 289945). **New Zealand (South Island).** Wangapeka Valley, R. Mason s.n., 22 Dec 1946, (CHR 58114); Hope River, Sandy Creek, P. J. de Lange 5086, 21 Jan 2001, (AK 287549); Lake Rotoiti, West Bay, W. Harris s.n., 30 Jan 1987, (CHR 437953); St Arnaud Range, Nocatchem Stream, P. J. de Lange 5129, 23 Jan 2001, (AK 286973, Duplicate: AD); Wairau Valley, between Coldwater and Judges Creeks, A. P. Druce APD1263, Jan 1991, (CHR 471855); Upper Buller River, Dellows Bluff, P. J. de Lange 4792 & P. I. Knightbridge, 7 Dec 2000, (AK 288290, Duplicate: AD); Maruia Springs, Calf Paddock, P. J. de Lange 6509 & P. I. Knightbridge, 7 Dec 2000, (AK 289968, Duplicate: AD); Red Hills, Maitland Creek, P. J. de Lange 5135, 23 Jan 2001, (AK 288548, Duplicate: AD); Awatere River, between the Hodder and Limestone Rivers, L. B. Moore s.n., 16 Feb 1962, (CHR 129190); Lewis Pass, St James Walkway Shelter, P. J. de Lange 5095, 21 Jan 2001, (AK 286185, Duplicate: AD); Hanmer Forest Park, Waterfall Trail, P. J. de Lange 4285 & B. P. J. Molloy, 8 Feb 2000, (AK 286136, Duplicates: AD, CHR); Culverden, Lowry Peaks Road, Culverden Scientific Reserve, P. J. de Lange 4284 & B. P. J. Molloy, 8 Feb 2000, (AK 2888540, Duplicates: AD, NSW, WAIK); Eyrewell Scientific Reserve, B. P. J. Molloy s.n., 30 Jan 1970, (CHR 201642). OTAGO: The Neck, between Lakes Hawea and Wanaka, B. P. J. Molloy s.n., 13 Apr 2006, (AK 296424); Dunstan Range, near Crippletown, Rocky Point (Tarras –Cromwell Road), Bendigo Reserve, B. P. J. Molloy s.n., 15 Mar 2001, (AK 289978, Duplicate: AD); Lake Roxburgh Bluffs, Clutha Valley, K. J. M. Dickinson s.n. & B. D. Rance, 24 Mar 1986, (OTA 43677); McCraes, Nenthorn Region, Manuka Creek, J. P. Burrell s.n., 18 Jan 1962, (OTA 7352).

#### Distribution

**Figure 12.**
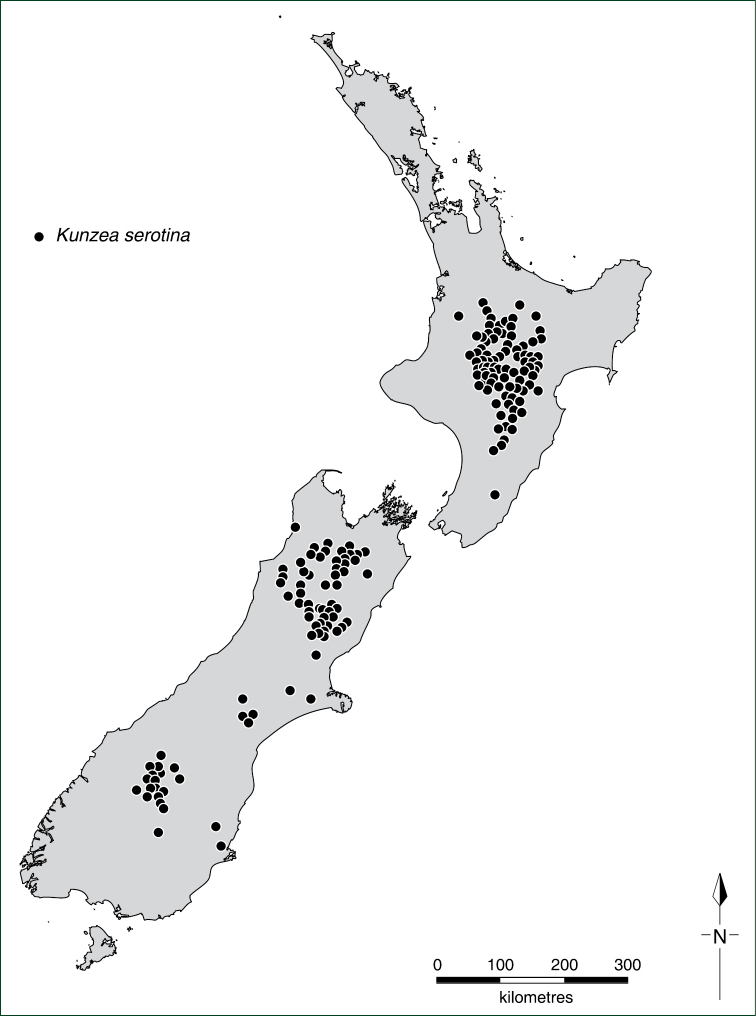
Distribution of *Kunzea serotina*.

**(Fig. 12).** Endemic, New Zealand, North and South Islands (30–2000 m a.s.l.). In the North Island present from about Te Kuiti, the Paeroa Range, Mt Tarawera and Kaingaroa Plain south through the Central Volcanic Plateau to the northern Aorangi Range. Absent from the high country west of Tongariro Forest, including Mt Taranaki/Egmont. In the South Island, present in the east from the upper Wairau River, and west from Karamea and the Wangapeka Valley inland along the upper Buller River and Nelson Lakes area, south through the main axial ranges to Sumner and the upper Hurunui catchment. Extending east into North Canterbury, particularly in the inland Hanmer, Emu and Amuri plains, thence present as isolated remnant stands on the Canterbury Plains. Otherwise apparently absent until Lakes Hawea and Wanaka from where it is locally present through portions of eastern Central Otago to about Roxburgh on the Clutha River and Nenthorn.

#### Recognition.

*Kunzea serotina* is recognised by a combination of characters including growth habit, bark type, branchlet hair, leaf, floral and fruit characters. No other member of the *Kunzea ericoides* complex has the same distinctive narrowly columnar to pyramidal growth habit, with obliquely ascending, fastigiate branches (Fig. 11A–D, G, H), a multitude of such densely leafy brachyblasts, spathulate pherophylls (Figs 9A, J–O, 11G, H), and petals copiously spotted with yellow oil glands (Fig. 9Q–R). The species also generally flowers later than most other species, with flowering tending to peak in January–February, and continuing as late as May.

*Kunzea serotina* was first recognised as distinct by W. Colenso who collected specimens in 1849 from the headwaters of the Makaroro River, eastern Ruahine Range (*Colenso 1618*, K, WELT SP022869—both mixed sheets, with the gatherings cited here being those labelled ‘A’) and who sent these to J.D. Hooker at Kew recommending their formal recognition with the suggested manuscript name “Leptospermum pulchrum”. *Kunzea serotina* was also recognised by Allan (1961) who described it briefly as a ‘thicket-forming variant’ confined to Marlborough, and it was mentioned by Harris (1996) who described it as ‘a small-leaved variant possibly worthy of taxonomic segregation’, and ‘occupying stations south of about Latitude 38°S’. As is often the case for New Zealand vascular plants (see Heenan 1998; Heenan et al. 2001; Heenan and de Lange 2004; Heenan and de Lange 2006) this species’ distinctiveness, so long overlooked by biosystematists, has long been appreciated by horticulturists who, in the North Island especially, have grown it widely, usually under the informal horticultural name *Kunzea* ‘Central North Island’ (R. Mains pers. comm.).

Throughout its range *Kunzea serotina* is sympatric with *Kunzea ericoides*, *Kunzea robusta*, and, in some thermal areas (notably around Lake Taupo), with *Kunzea tenuicaulis*. In these areas despite the widespread sympatry, *Kunzea serotina* mostly forms pure populations with little evidence of hybridism (see below).

*Kunzea serotina* is distinguished from *Kunzea robusta*, by its columnar, pyramidal growth habit and singular bark-type (Fig. 11A–H). However, in very old specimens of *Kunzea serotina* the columnar, pyramidal growth habit tends to be less marked or lost altogether because the lower branches thin with age, leaving trees with usually flat-topped branches crowded toward the top. In these situations, the bark–type will distinguish both species when it is not possible to observe foliage or flowers. *Kunzea serotina*, uniquely of the New Zealand species, has bark that is very easily detached, chartaceous, greyish-white to pinkish-white in colour, with highly irregular and sinuous margins (Fig. 11E–F). The bark is often found hanging partially detached as inrolled or curled up lengths within the branch forks, or it forms piles of curled up ‘wood shavings’ around the trunk base. In contrast, the bark of *Kunzea robusta* has a distinctly coriaceous texture which detaches basally first then peels up the trunk forming long (up to 4 m) broad to narrowly tabular strips with ± smooth, ± entire margins.

The branchlet hairs of *Kunzea serotina* consistently differ from those of most *Kunzea robusta* populations by their smaller (up to 0.08 mm) divergent, apically curved or curled (Fig. 10B, C–E) rather than longer (up to 0.38 mm), weakly flexuose, antrorse-appressed hairs. However, in some parts of the eastern Central Volcanic Plateau the length of the branchlet hairs of *Kunzea robusta* falls within the range cited for *Kunzea serotina*, although in these situations the hairs are still antrorse rather than divergent. In the upper Rangitikei, distinction of seedlings and saplings of *Kunzea robusta* from *Kunzea serotina* can be difficult because in that area young plants of *Kunzea robusta* usually have short divergent hairs of the same size range and form as *Kunzea serotina*. However, adult trees of *Kunzea robusta* from the same area (and those raised from these sites in cultivation) possess the more usual antrorse hairs. In these areas the growth habit of both species immediately distinguishes them. Floral and fruit characters also separate both species. In particular the spathulate to spathulate-orbicular pherophylls of *Kunzea serotina* (Fig. 9L–O) are unique among the New Zealand species and are a marked contrast to the narrowly deltoid, lanceolate, elliptic, oblanceolate to broadly oblanceolate, squamiform and/or foliose pherophylls typical of *Kunzea robusta*. Further, the petals of *Kunzea serotina* are uniquely dotted with yellow rather than the colourless or rose-pink oil-glands typical of other New Zealand species. Although the distributions of both species frequently overlap, *Kunzea serotina* is ecologically distinctive, being a species of inland basins, upland river flats, frost flats, tussock grassland, ridge crests of mountain ranges, and subalpine scrub, favouring sites prone to extremes of climate and often with recent or skeletal soils. *Kunzea robusta* is much more generalist in its habitat preferences but as a rule it is more of a coastal, lowland to montane species favouring better developed soils, less extreme climates, and conditions that tend to suit the development of mixed lowland forest types. While *Kunzea serotina* has been found at an upper altitudinal limit of 2000 m a.s.l. on Mt Ruapehu, *Kunzea robusta* is rarely seen above 800 m a.s.l. Thus, the main areas of sympatry are generally places where *Kunzea serotina* has colonised suitable lowland habitats abutting those dominated by *Kunzea robusta*, e.g., the pseudo-karst ignimbrite country around and north of Lake Taupo toward Te Kuiti and Tokoroa, or the steep-sided river valleys of the Kaimanawa and Kaweka Ranges.

The growth habit of *Kunzea serotina* (Fig. 11A–D) readily distinguishes it from the prostrate/decumbent shrub or multi-trunked widely spreading, flat-topped tree habit of *Kunzea tenuicaulis*. In addition, both species can be distinguished by their branching pattern with the obliquely ascending, shortly fastigiate branches of *Kunzea serotina* a marked contrast to the slender, widely spreading to pendulous branches of *Kunzea tenuicaulis*. The pherophylls are also distinctive; those of *Kunzea tenuicaulis* are mostly oblong, oblong-obovate to oblanceolate while those of *Kunzea serotina* are characteristically spathulate. The calyx lobes of *Kunzea serotina* are weakly keeled and flush with the rest of the hypanthium whereas in *Kunzea tenuicaulis* they are distinctly thickened toward the base, and the external junction with the hypanthium is marked by a faint to prominent groove. The flower petals of *Kunzea serotina* readily distinguish the species due to their being uniquely furnished with yellow rather than colourless oil glands. The fruit of both species is also diagnostic, with those of *Kunzea serotina* mostly urceolate to campanulate (rarely cupular) (Fig. 9V), while the fruits of *Kunzea tenuicaulis* tend to be barrel-shaped to cupular. *Kunzea serotina* and *Kunzea tenuicaulis* are generally separated by their ecology, with the latter endemic to geothermal habitats. Sympatry occurs mostly in the vicinity of LakeTaupo where the ranges of both species overlap, and where on occasion *Kunzea serotina* will colonise the more quiescent ground or cold ‘inliers’ of geothermal fields. Induced sympatry also occurs along the urbanised northern shoreline of Lake Taupo and at Wairakei, Karapiti and Tokaanu, where modification of the geothermal field for tourism has allowed *Kunzea serotina* (and *Kunzea robusta*) to more freely colonise habitats more usually occupied by *Kunzea tenuicaulis*.

The columnar to pyramidal growth habit of *Kunzea serotina* readily separates it from *Kunzea ericoides*. Sometimes, as in shaded, damp or upland areas, the old bark of *Kunzea ericoides* may have secondary peeling, some of which curls in a manner reminiscent of *Kunzea serotina*. In these situations the foliage separates the species from each other. The leaves of *Kunzea ericoides* are much longer (up to 25 mm long), mostly bright green and mostly linear, whereas *Kunzea serotina* has distinctly shorter (up to 6.3 mm long), bronze-green to dark green, oblanceolate to obovate leaves. Both species have divergent branchlet hairs, but in *Kunzea ericoides* they are sparse, shed early in branchlet maturation, smaller (up to 0.05 mm long) and have straight apices; in *Kunzea serotina*, they are copious, persistent, longer (up to 0.08 mm long), with curved or curled apices. The flowers and fruits also differ. The petals of *Kunzea ericoides* are usually sparsely dotted with ± colourless oil glands, and the fruits are slightly larger (up to 3.4 × 3.9 mm), mostly glabrous, typically 5-locular, and mostly cupular, barrel-shaped, shortly cylindrical or hemispherical. The petals of *Kunzea serotina* are copiously dotted with yellow oil glands and the fruits are smaller (up to 3.0 × 3.4 mm), finely hairy, 3–4 locular, and urceolate to shortly-campanulate (Fig. 9V).

Although the other ‘small-leaved’ *Kunzea* species, *Kunzea salterae* and *Kunzea toelkenii*, are allopatric from *Kunzea serotina*, they could be confused in the herbarium. *Kunzea serotina* leaves (up to 6.3 × 1.8 mm) are smaller than the leaves of *Kunzea salterae* (up to 18 × 2 mm), and in *Kunzea serotina* they are oblanceolate to obovate rather than linear-lanceolate to narrowly oblanceolate. *Kunzea serotina* has spathulate pherophylls, and urceolate to campanulate, very rarely cupular, rather than cupular to sub-campanulate fruits. Herbarium material of *Kunzea serotina* differs from *Kunzea toelkenii* by its consistently divergent branchlet hairs rather than admixed large, antrorse-appressed, weakly flexuose, and small, divergent, curled hairs. *Kunzea serotina* also has smaller leaves (up to 6.3 × 1.8 mm) than *Kunzea toelkenii* (up to 8.5 × 2.5 mm) and *Kunzea serotina* lacks functionally male late season flowers. Other differences are summarised by Table 1.

Molecular evidence (rDNA ITS) grouped *Kunzea serotina* with *Kunzea ericoides*, *Kunzea linearis*, *Kunzea triregensis*, *Kunzea robusta*, *Kunzea sinclairii* and *Kunzea tenuicaulis* (Table 2; see also de Lange 2007; de Lange et al. 2010) while the rDNA ETS sequence of *Kunzea serotina* shares a guanine/cytosine mix with *Kunzea ericoides*, *Kunzea linearis*, *Kunzea robusta* (Mt Egmont samples only), *Kunzea salterae* and *Kunzea toelkenii*, and an adenine with *Kunzea salterae*, *Kunzea tenuicaulis* and *Kunzea toelkenii* (Table 2; see also de Lange 2007; de Lange et al. 2010). Although there are no unique characters present in either of these marker regions, the inferred relationship of *Kunzea serotina* with *Kunzea salterae*, *Kunzea tenuicaulis* and *Kunzea toelkenii*, was already noted karyologically by de Lange and Murray (2004). Those data, coupled with the morphology of these ‘small-leaved’ species suggests that they are probably related and may form a natural group.

#### Ecology.

*Kunzea serotina* is an important component of the vegetation of the central North Island ranges. In the South Island *Kunzea serotina* attains local prominence in the montane vegetation of the Marlborough, the northern Southern Alps, and the dry intermontane basins of north Canterbury and eastern Central Otago.

*Kunzea serotina* has the highest altitudinal limit of all the New Zealand *Kunzea*, frequently growing above 1000 m a.s.l., and reaching its maximum recorded elevation as stunted shrubs at 2000 m a.s.l. on steep, sparsely vegetated slopes growing amongst other alpine herbs and grasses in the upper Mangatoetoenui Stream, on the eastern flanks of Mt Ruapehu. This habitat is, however, rather unusual. More typically, in the North Island *Kunzea serotina* grows along the ecotone between tall forest and tussock grassland, wetlands, frost flats or within grey scrub. In some places, most notably within the ignimbritic pseudo-karst of the north and eastern Volcanic Plateau, *Kunzea serotina* forms a distinct, often ecotonal forest along the boundaries of the frost flats formed by numerous low aspect ratio ignimbritic eruptions, mostly sourced from the Taupo Volcanic Centre, (Houghton et al. 1995; Wilson et al. 1995). The South Island equivalent of this habitat seems to be the dry intermontane basins east of the main divide, especially those of north Canterbury, where remnant stands of *Kunzea serotina* are still locally common. In these montane and inland basin habitats, *Kunzea serotina* appears to have a long-term presence, and, if left undisturbed by fire, it probably would form the natural climax woody vegetation, particularly in areas prone to summer drought and extreme frosts and snow falls during winter.

#### Hybridism.

Although *Kunzea serotina* is sympatric with *Kunzea ericoides*, *Kunzea robusta* and *Kunzea tenuicaulis*. It appears to hybridise most frequently with *Kunzea robusta*.

Hybrids with *Kunzea ericoides* are easily recognised in the field and in herbaria because of the obvious differences in branching pattern, leaf colour, size and shape. This hybrid is probably uncommon because the distributions of *Kunzea ericoides* and *Kunzea serotina* rarely overlap. Putative *Kunzea ericoides* × *Kunzea serotina* has been been sparingly collected from Mt Dun, the Barnicoat Range and Hackett areas of eastern Nelson, and from the upper Buller and Sabine Rivers in southern Nelson are recognised. These are all areas where for various reasons the normally more montane and cold tolerant *Kunzea serotina* extends outside its usual range.

*Kunzea ericoides* × *Kunzea serotina* is easily recognised by the erect, somewhat open, sub-pyramidal growth habit, short, weakly fastigiate branchlets, and bright yellow-green, red-tinged leaves which are broadly lanceolate to linear-lanceolate. In most cases the branchlets tend to be distinctly hairy, though they can also be glabrescent. The inflorescences vary from corymbiform to widely spaced, elongate botrya. The pherophylls are deciduous and mostly pandurate to elliptic, and usually intermediate in length between either of the parents.

Recognition of the hybrid *Kunzea serotina* × *Kunzea tenuicaulis* is difficult, and examples of this hybrid have probably gone undetected during this study in herbaria (see *Kunzea tenuicaulis*). The difficulty arises not so much in the field where the intermediate growth habit assists with hybrid recognition, but rather with herbarium material, from which hybridism can be difficult to infer if the accompanying notes are scant. Irrespective, it does seem that this hybrid is genuinely scarce, probably in part because it is only in the southern two-thirds of the Taupo Volcanic Centre that the ranges of either parent species overlap, and then mostly because human disturbance has induced novel habitats that both species (and the ubiquitous *Kunzea robusta*) have utilised. Nevertheless, putative hybrids are represented by three herbarium specimens, from Karapiti (Craters of the Moon), Taupo on the shores of Lake Taupo, and at Tokaanu. Constant human activity at these places has allowed the ranges of these species to overlap and this disturbance has maintained the conditions for them. Beyond these modified habitats, *Kunzea serotina* though frequently found near sites of geothermal activity appears to avoid colonising them. Even in modified geothermal habitats, *Kunzea serotina* remains scarce, favouring local cold spots often on higher ground, well away from the main areas of thermal activity. However, because *Kunzea tenuicaulis* frequently colonises cold spots within thermal areas, it is not unusual to find isolated shrubs or trees of *Kunzea serotina* surrounded by and often partially smothered by *Kunzea tenuicaulis* growth. Under such conditions it is surprising that hybridism between these two species seems to be so uncommon, especially as the flowering times of both species overlap. The scarcity of hybrids possibly reflects something deeper. In the experimental cross *Kunzea serotina*^♀^ × *Kunzea tenuicaulis*^♂^, and its reciprocal, the progeny all had 2*n* = 23 chromosomes (de Lange and Murray 2004; de Lange et al. 2005). Few of these plants flowered. Pollen stainability from these plants was markedly reduced (12–23%). These results suggest that there might be some reproductive barrier in place which could explain the scarcity of this hybrid in the wild. Further investigation into this is needed.

The most commonly encountered hybrid is *Kunzea robusta* × *Kunzea serotina* because the ranges of both species frequently overlap. This is particularly the case in the North Island, around the northern Central Volcanic Plateau, southern Kaweka Ranges, and upper Rangitikei catchment. Hybrid swarms are common in these places, especially in grossly disturbed habitats such as reverting farmland or roadsides. Field recognition of hybrids is aided by the effect of the distinctive growth habit of *Kunzea serotina*, which results in somewhat openly branched, sub-pyramidal to widely spreading, sparingly branched, small spindly trees. The branchlets may be fastigiate or spreading but are usually shorter than in *Kunzea robusta*. The distinctive bark of *Kunzea serotina* is often present to some degree, though it tends to peel off in long tabular strips like *Kunzea robusta* but with much secondary peeling. It is the secondary peels that tend to curl up as *Kunzea serotina* bark normally does. Branchlet hairs are particularly diagnostic because *Kunzea robusta* has antrorse-appressed and *Kunzea serotina* has divergent hairs, with hybrids showing obvious mixtures of both. The pherophylls of *Kunzea robusta*, though very variable in shape and length, are mostly deltoid to lanceolate, and are usually present in mixtures of foliose and squamiform types. In *Kunzea serotina* the pherophylls are usually foliose and spathulate, spathulate-orbicular, only rarely pandurate or lanceolate. Thus, hybrids tend to show mixtures of sparse deciduous, pandurate, and lanceolate foliose and frequent squamiform pherophylls.

Artificial hybrids involving *Kunzea serotina* as staminate or pistillate parent and other New Zealand members of the *Kunzea ericoides* complex were easily produced and showed no obvious reduction in fertility except for crosses involving *Kunzea tenuicaulis*, which were effectively sterile (see de Lange and Murray 2004; de Lange et al. 2005).

#### Vernacular.

*Kunzea serotina* is (or was) locally known to Maori inhabiting the Central North Island as ‘makahikatoa’. Makahikatoa is said to mean ‘white kahikatoa’ referring to the wood colour, and is a name intended to distinguish white-wooded *Kunzea serotina* from the red-wooded ‘warrior-wood’ of kahikatoa (*Leptospermum scoparium*) (W. Kawhaki pers. comm.). A few herbarium gatherings made during the 1800s also refer to this species as ‘manuka’.

#### Conservation status.

*Kunzea serotina* is an abundant and widespread species which should not be regarded as threatened or at risk (a more detailed account of its conservation status, using the criteria of Townsend et al. (2008) at both national and regional levels is provided by de Lange (2007).

### 3. Kunzea tenuicaulis

Taxon classificationPlantaeMyrtalesMyrtaceae

de Lange sp. nov.

urn:lsid:ipni.org:names:77141734-1

A K. ericoide habitu fruticis decumbentis ad serpentis vel arboris erectae sed patentis multicaulis ad pendentibus, caulibus et ramibus multis tenuibus vel ramulibus numerosis patentibus ad pendentibus tenuissimis, ramulibus juvenibus dense tomentosis pilis multis brevibus patentibus, foliis brevioribus oblanceolatis ad obovatis; hypanthio minore cupuliformi campanulato gracile puberulenti; lobis calicis distincte incrassatis basipetale et sulco ad basim, fructibusque minoribis doliiformibus differt. Chromosomatibus constanter parvis et equatis seriebusque singularibus rDNA ITS et ETS differt.

#### Holotype

**Figure 13.**
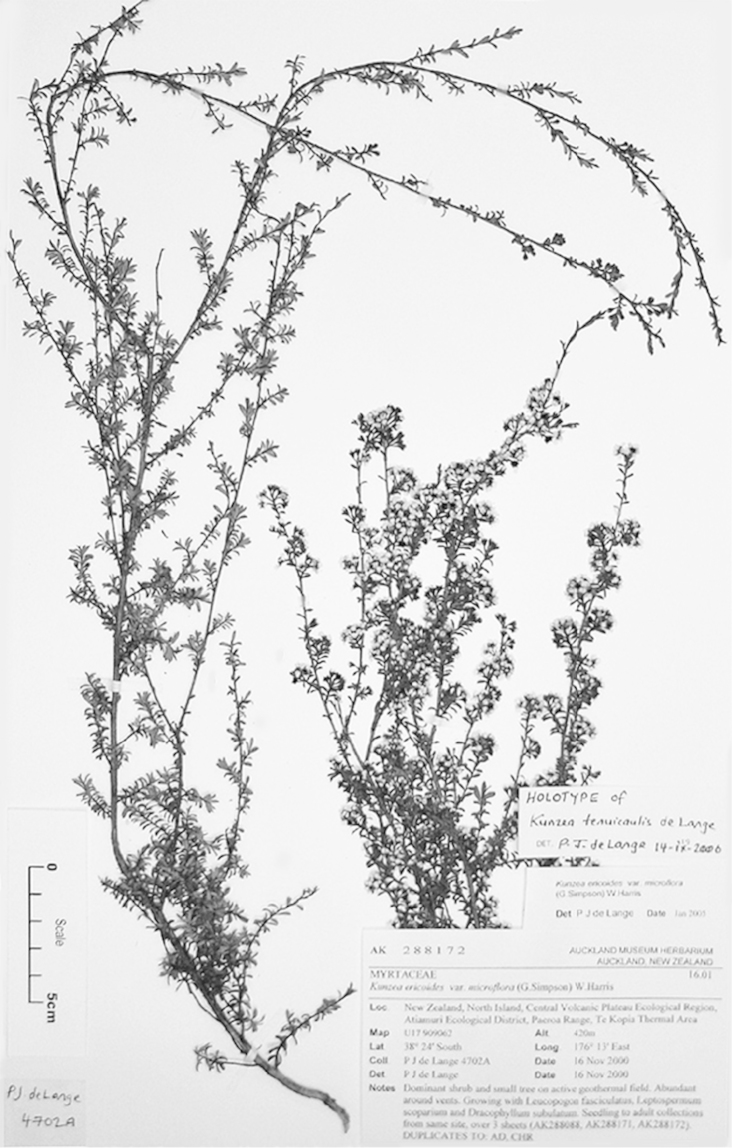
Holotype of *Kunzea tenuicaulis* de Lange (*P. J. de Lange 4702A*, AK 288172).

**Figure 14.**
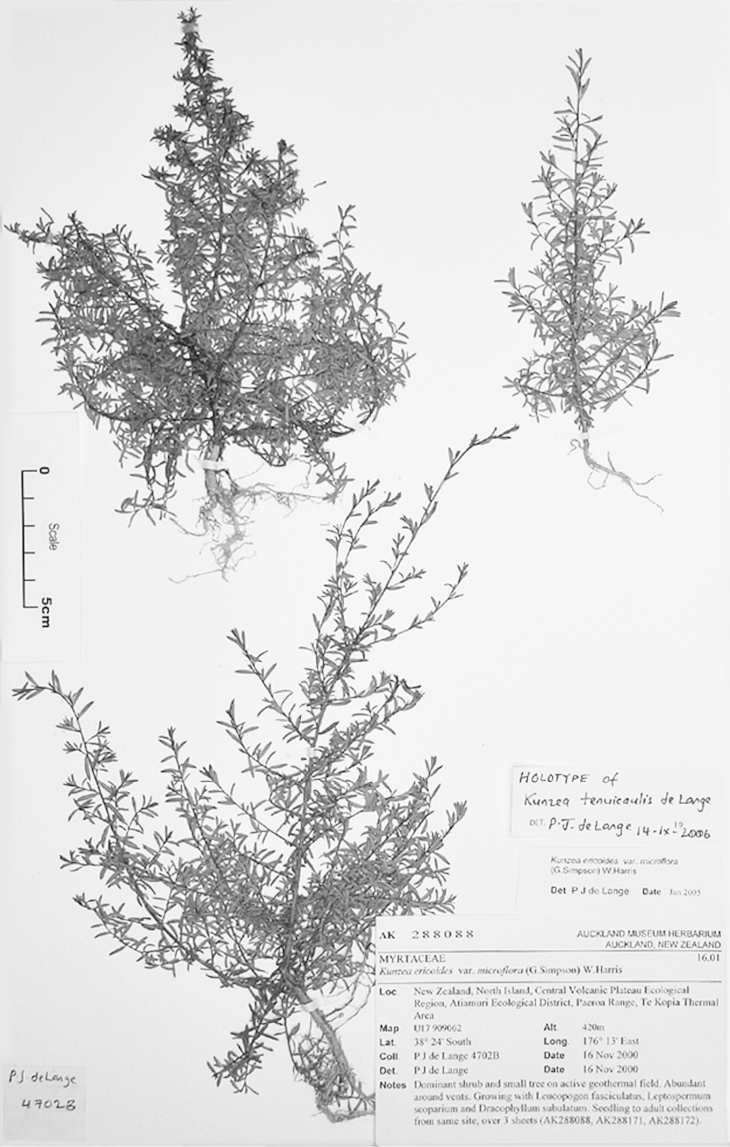
Holotype of *Kunzea tenuicaulis* de Lange (*P. J. de Lange 4702B*, AK 288088).

**Figure 15.**
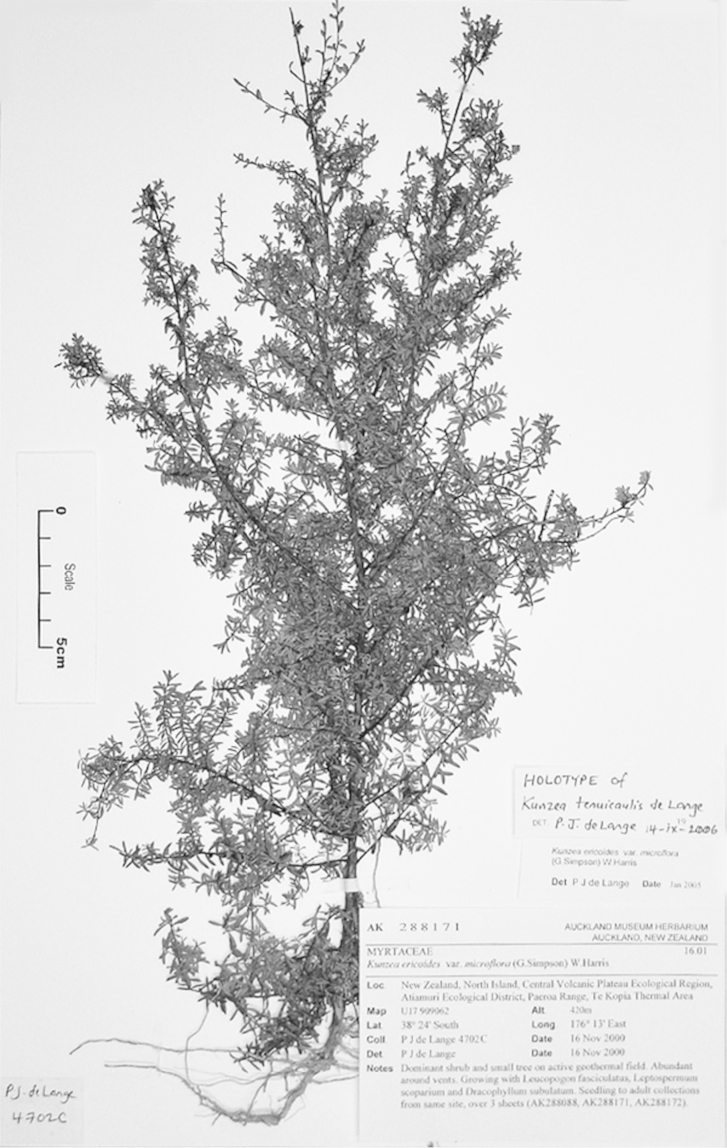
Holotype of *Kunzea tenuicaulis* de Lange (*P. J. de Lange 4702C*, AK 288171).

**(Figs 13, 14, 15)** **(Spread over three sheets).** **New Zealand:** North Island, Central Volcanic Plateau, Paeroa Range, Te Kopia Thermal Area, 38°24'S, 176°13'E, 420m a.s.l. ‘Dominant shrub and small tree on active geothermal field. Abundant around vents. Growing with *Leucopogon fasciculatus*, *Leptospermum scoparium* and *Dracophyllum subulatum*, Seedling to adult collections from same site, over 3 sheets (AK 288088!, AK 288171!, AK 288172!)’. *P. J. de Lange 4702A*, *B*, *C* 16 Nov 2000, AK 288172 (Adult branch in bud, and branch with flowers and buds), AK 288088 (three seedlings), and AK 288171 (One sapling). Isotypi: AD, CHR.

#### Notes.

The holotype gathering of *Kunzea tenuicaulis* comprises six specimens spread over three sheets and lodged at the same herbarium. The two adult specimens mounted on AK 288172, *de Lange 4702A*, come from the same plant, while the three seedlings (AK 288088, *de Lange 4702B*) and sapling (AK 288171, *de Lange 4702C*) were collected from the ground directly beneath that plant.

The manner in which I have collected and designated these sheets as holotype is in accordance with the International Code of Nomenclature (McNeill et al. 2012) Article 8.2 ‘….the holotype may consist of a single plant, parts of one or several plants, or of multiple plants…’ Thus in accordance with Article 8.3, Example 4 of the International Code of Nomenclature (McNeill et al. 2012), my collecting numbers reflects my intended association of these gatherings as type.

*Kunzea tenuicaulis* most probably equates with Kunzea ericoides var. microflora (G.Simpson) W.Harris which is based on Leptospermum ericoides var. microflorum G.Simpson (Simpson 1945). This variety was described from garden specimens sent to George Simpson by Norman Potts of Opotiki, and which, according to Simpson, came from ‘Rainbow Mountain, Nelson’ where its habitat was said to be the ‘Mineral Belt, Nelson’. As noted by Allan (1961; pp. 322–323), no plants matching Simpson’s description occur in the Nelson area or, indeed, the rest of the South Island. Furthermore, there is no Rainbow Mountain in the South Island. However, plants similar to Simpson’s description have been found in active geothermal areas within the Taupo Volcanic Zone of the North Island from a geothermal site near Lake Rotoiti (Tikitere) in the north west and Kawerau in north east to just south of Tokaanu, near Turangi at the southern end of Lake Taupo. These are the plants referred here to *Kunzea tenuicaulis*.

**Figure 16.**
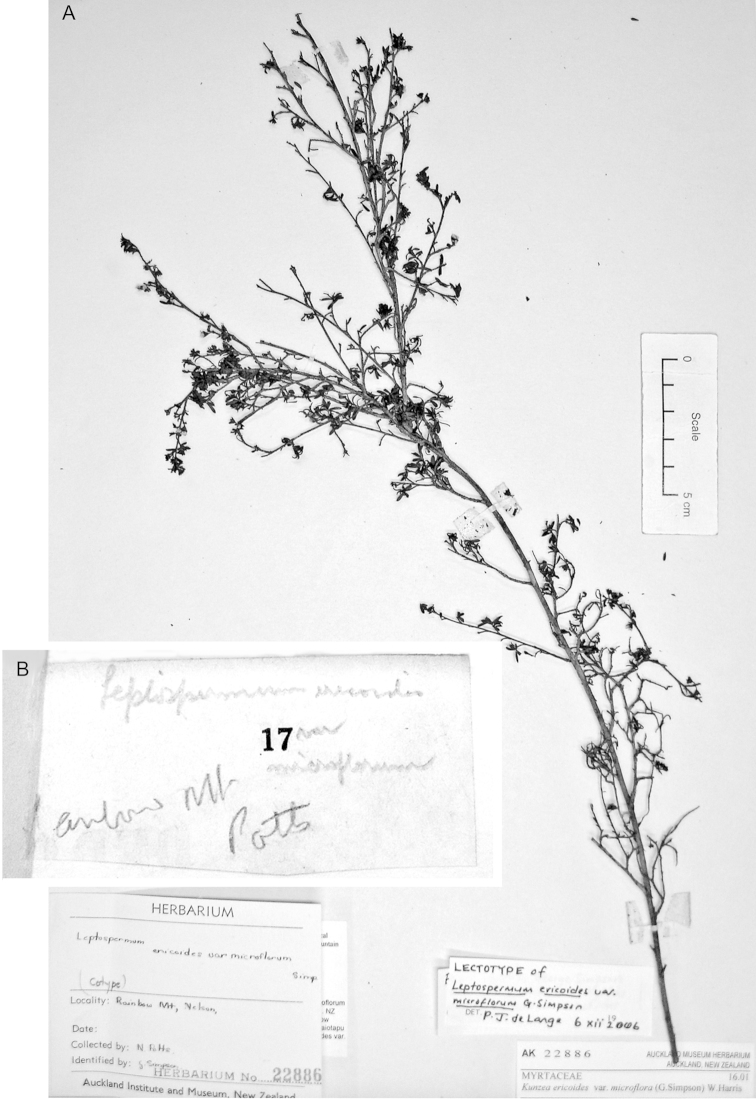
Lectotype of Leptospermum ericoides var. microflorum G.Simpson (*N. Potts s.n.*, AK 22886). **A** Specimen **B** George Simpson’s handwriting on piece of newspaper mounted on AK 22886 over which two AK herbarium labels mounted.

Allan (1961; p. 322) had also recognised this, and sought to rectify what he considered to be a genuine geographic mistake in Simpson’s protologue, by his statement that Simpson’s type locality was in fact ‘Rainbow Mountain near Waiotapu’. However, because Allan’s opinion cannot be matched with any supporting statement or evidence from Simpson, we simply cannot be certain where Simpson’s type (based on Norman Pott’s garden specimens) really came from. Further, the only specimen that I can unequivocally say is labelled by Simpson as Leptospermum ericoides var. microflorum is in extremely poor condition (AK 22886; Fig. 16A, B). The all-important diagnostic branchlet hairs are scant, there are only eight flowers left that are so shrivelled and damaged as to make a proper reconstruction impossible, and most of the foliage is missing. While I am convinced that Simpson’s type matches what I have named *Kunzea tenuicaulis*, I have elected to remove any further ambiguity by redescribing the *Kunzea* endemic to the geothermal fields of the Taupo Volcanic Zone at the rank of species, with a full and detailed description, and an unambiguous wild type and duplicates gathered from a wild legally protected site where the species is uniform and widespread, and hybridism is not evident.

#### Etymology.

The specific epithet *tenuicaulis* refers to the numerous very fine and slender branchlets produced by this species, irrespective of its overall growth habit.

= Leptospermum ericoides var. microflorum G.Simpson in *T.R.S.N.Z.* 75, (1945), 189.

≡ Kunzea ericoides var. microflora (G.Simpson) W.Harris in *N.Z.J.Bot.* 25, (1987), 134.

#### Lectotype

**Figure 17.**
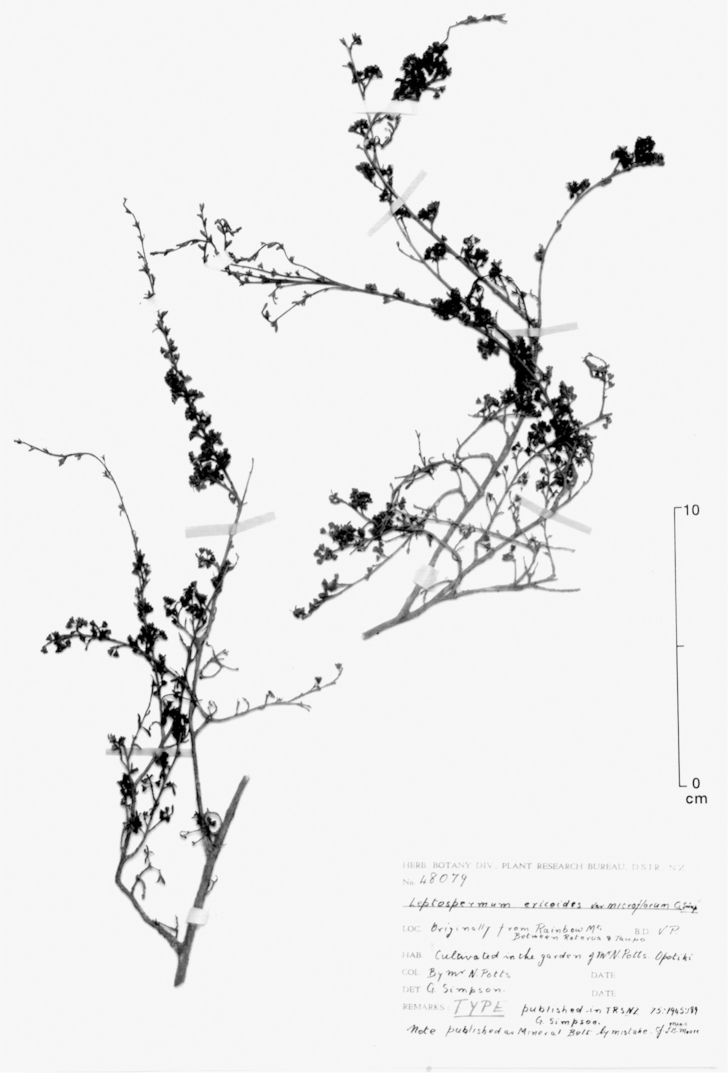
Lectotype of Leptospermum ericoides var. microflora G.Simpson (*N. Potts s.n.*, CHR 48079) as selected by Allan (1961). This sheet has no nomenclatural status as it cannot unequivocally be shown to have been handled by the naming author George Simpson. The label details are written in the hand of T. Rawson then technician to H. H. Allan (P. B. Heenan pers. comm.).

**(here designated)** **(Figs 16, 17).** “Leptospermum ericoides var. microflorum, [R]ainbow Mt, Potts” AK 22886! (label in George Simpson’s hand, written in pencil on back of piece of printed paper (possibly newspaper) (Fig. 16B)).

#### Notes.

Leptospermum ericoides var. microflorum was briefly described by Simpson (1945) and for most of his protologue his intent is clear. Simpson’s type collections were based on a specimen that had been cultivated by Mr N. Potts of Opotiki. Potts’s plant was said to have come from Rainbow Mountain (Simpson 1945). The fact that

Potts, a North Island botanist, wrote to Simpson stating “the plant occupies craters on Rainbow Mountain” strongly suggests that his plant had come from Rainbow (Maungakakaramea) Mountain (38°19'S, 176°23'E), an old volcanic vent and active geothermal field just to the north east of the small settlement of Waiotapu on the western marches of the Kaingaroa State Forest in the North Island.

Despite this, Simpson explicitly states ‘Habitat: Mineral belt, Nelson’ (a South Island location), and further ‘Specimens from a plant in cultivation by Mr N. Potts’ garden at Opotiki, collected at Rainbow Mountain, Nelson’. Subsequently, Allan (1961; p. 322) stated that the variety is ‘known with certainty only from the type locality: Rainbow Mountain near Waiotapu’ and that Simpson has ‘inadvertently given the locality as “Mineral belt, Nelson”’. This, while probably true (see above) is not strictly correct, as Simpson twice cited Nelson as the source of the plants, and as far as I can determine never clarified the apparent ambiguity in subsequent literature or correspondence with Allan or anyone else—though the handwriting on CHR 48079, ‘Note published as Mineral Belt by mistake. cf. Miss L.B.Moore’ suggests that L. B. Moore might have obtained some comment from someone about the original source of Potts’s specimens. Irrespective, based on available evidence we cannot be sure of what Simpson’s statements about the source of his type material really meant.

Simpson (1945) also explicitly states that the ‘*specimens*’ (my emphasis) on which he based his name were lodged in the ‘Herbarium Plant Research Bureau, Wellington’ then known as BD (Botany Division) and now CHR. Allan (1961; p. 322) lectotypified the name (from a specimen in the former Botany Division Herbarium that he evidently considered was part of the type gathering) in the following manner ‘Type: BD 48079, from a plant cultivated by N. Potts’. That specimen (Fig. 17), now CHR 48079, is clearly labelled ‘TYPE’ in red ink, in what is probably the hand of T. Rawson, then Technician to Allan (P. B. Heenan pers. comm.). However none of the associated handwriting on the label is in Simpson’s hand (see Heenan 1995) and, beyond the filing of the specimen in the ‘Simpson Herbarium’ there is nothing to clearly identify this gathering as one that Simpson had actually handled, let alone anything to show that Potts gathered the specimen. For these reasons I reject Allan’s typification.

In AK I have located a further gathering attributed to Simpson, AK 22886, bearing several labels (Fig. 16A, B). The main label is an official one of the type used at AK between 1929 and about the early 1970s (E. K. Cameron pers. comm.) and is labelled in blue ink ‘Rainbow Mt., Nelson, N. Potts’ and ‘cotype’ in handwriting that is most likely that of the herbarium curator at AK toward the end of the 1940s, B. E. Molesworth. Beneath that label, on the back of a small scrap of what appears to be newspaper, is a pencil annotation in Simpson’s distinctive handwriting (Fig. 16B; see also Heenan 1995), ‘Leptospermum ericoides var. microflorum …[R]ainbow Mt…Potts’. As the official AK label was inadvertently glued over part of Simpson’s handwriting, what I regard as the letter ‘R’ has been partially erased by glue and dirt and is thus no longer fully legible (Fig. 16B). There is no doubt in my mind however, that the locality on the label is Rainbow Mountain.

Although Simpson makes it clear that specimens of his new variety were to be found in what is now the Allan Herbarium (CHR), I cannot now find any specimens there that unequivocally show this, and yet, as Simpson indicates that he had lodged ‘specimens’, I feel it unwise to regard the AK specimen as a holotype because other collections may exist. For this reason I select AK 22886 as lectotype. I consider CHR 48079 as having no nomenclatural status because there is no evidence that Simpson ever handled or in anyway used it to describe his variety.

#### Etymology.

The varietal epithet ‘*microflorum*’ was adopted by Simpson (1945) in the mistaken belief that his Leptospermum ericoides var. microflorum had smaller flowers than the type variety.

#### Description

**Figure 18.**
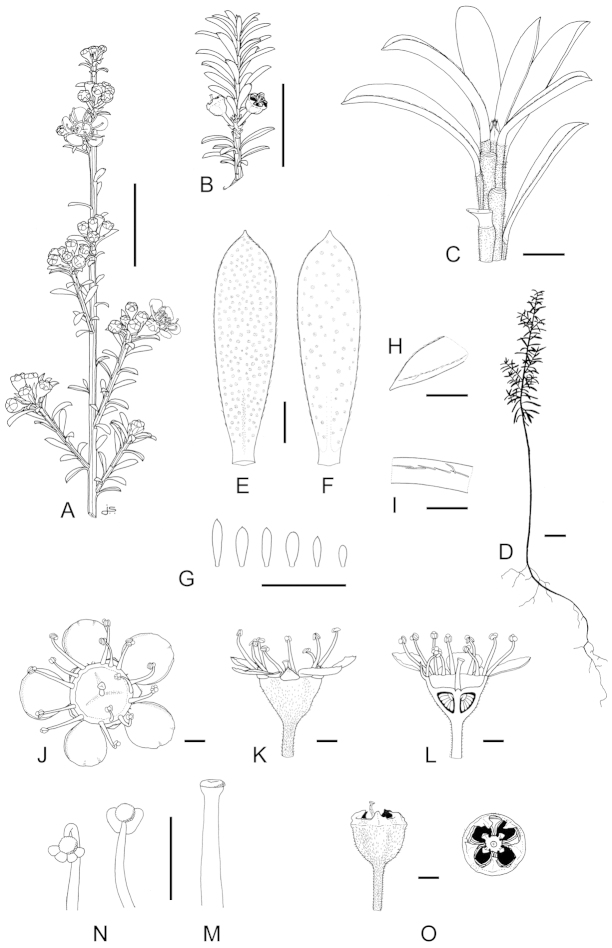
Distinguishing features of *Kunzea tenuicaulis*. **A** Flowering branchlet (ex cult. AK 284554) **B** Fruiting branchlet (ex cult. AK 284554) **C** Vegetative bud and branchlet indumentum (ex cult. AK 284554) **D** Seedling (no voucher, self sown from AK 284554) **E** Adaxial leaf surface (ex cult. AK 284554) **F** Abaxial leaf surface (ex cult. AK 284554) **G** Leaf variation within the same individual (ex cult. AK 284554) **H** Adaxial leaf apex (ex cult. AK 284554) **I** Leaf margin indumentum (ex cult. AK 284554) **J** Flower (top view) (ex cult. AK 284554) **K** Flower and hypanthium (side view) (ex cult. AK 284554) **L** Flower cross section showing anther, style and ovules (ex cult. AK 284554) **M** Style and stigma (ex cult. AK 282217) **N** Stamens (ex cult. AK 282217) **O** Dehisced fruit (ex cult. AK 282217). Scale bars: (**A, B, D, G**) 10 mm; (**C, E, F, J–O**) 1 mm; (**I**) 0.5 mm.

**Figure 19.**
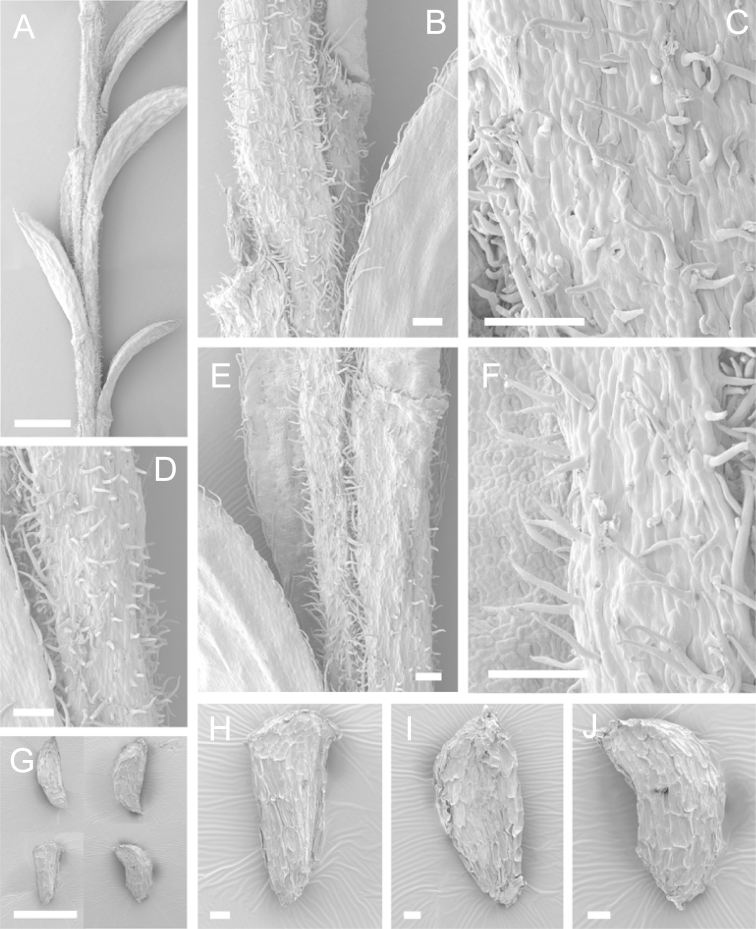
Scanning Electron Micrographs of *Kunzea tenuicaulis*. **A–F** all AK 288105) Branchlet indumentum **G–J** Seeds (AK 286159, AK 288105). Scale bars: (**A, G**) 1 mm; (**B–F, H–J**) 100 μm.

**Figure 20.**
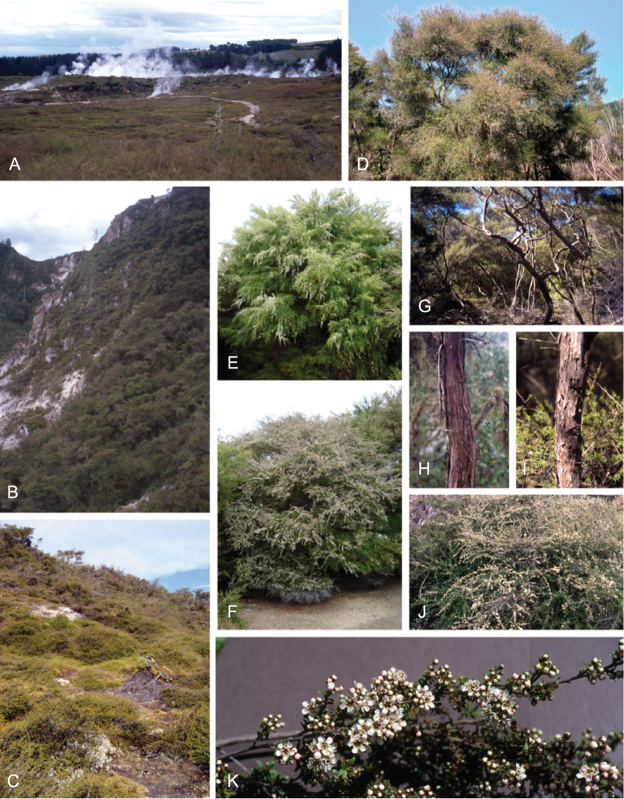
*Kunzea tenuicaulis*. **A** *Kunzea tenuicaulis* habitat, North Island, Karapiti (Craters of the Moon) (photo: *P. J. de Lange*) **B** *Kunzea tenuicaulis* habitat, North Island, Maungakakaramea (Rainbow Mountain) Scenic Reserve, Crater area (photo: *P. B. Cashmore*) **C** *Kunzea tenuicaulis* decumbent form on heated ground, Maungakakaramea (Rainbow Mountain) Scenic Reserve, Crater area (photo: *P. B. Cashmore*) **D** *Kunzea tenuicaulis* tree form showing multi-trunked growth habit and widely spreading, narrow branchlets, North Island, Rotorua, Kuiarau Park (photo: *P. B. Cashmore*) **E** *Kunzea tenuicaulis* tree form showing pendulous growth habit, North Island, Rotorua, Kuiarau Park (photo: *P. B. Cashmore*) **F** *Kunzea tenuicaulis* tree form showing multi-trunked, widely spreading, pendulous growth habit, North Island, Rotorua, Kuiarau Park (photo: *P. B. Cashmore*) **G** *Kunzea tenuicaulis* showing characteristic, flexuose trunks and widely spreading branches; North Island, Tokaanu, Tokaanu Geothermal Reserve, (photo: *P. J. de Lange*) **H** *Kunzea tenuicaulis* trunk and bark, North Island, Tokaanu, Tokaanu Geothermal Reserve, (photo: *P. J. de Lange*) **I** *Kunzea tenuicaulis* trunk and bark, North Island, Paeroa Range, Te Kopia Geothermal Reserve (photo: *P. J. de Lange*); (**J**) *Kunzea tenuicaulis* branches showing distinctive widely spreading, fine, pendulous branchlets, North Island, Tikitere (Hell’s Gate) Thermal Park, (photo: *P. J. de Lange*) **K** *Kunzea tenuicaulis* flowering branchlet showing, compact corymbiform botrya, North Island, Waiotapu Geothermal Park (photo: *G. M. Crowcroft*).

**(Figs 18, 19, 20).** *Growth habit* decumbent, trailing subshrubs, shrubs or small trees 0.1–6.0(–8.0) × 2.0–6.0(–8.0) m. For specimens with a tree habit, crown widely spreading, often arching to somewhat pendulous. For specimens found around active fumaroles or on open, geothermally heated ground, growth habit varying from completely decumbent and densely branched, with stems sprawling across ground, to semi-erect, densely branched, widely spreading, often pendulous. *Trunk* in tree forms (1–)4–6 arising from base, 0.1–0.6 m d.b.h., these branching from close to base, with branches thinning in close canopies only; in decumbent plants trunk virtually indistinguishable, 0.01–0.10 m diam., trailing to semi-erect, curved and somewhat sinuous, in erect plants at first erect, soon widely spreading and curving to somewhat sinuous. *Bark* early bark greyish brown to brown, initially firm, somewhat sinuous-fluted, elongate, over time cracking transversely (especially on branch flanges and decurrent leaf bases), and with margins gradually detaching and rolling-in to present as easily detached, papery, narrowly short to long, somewhat irregular-margined flakes; old bark grey-brown to grey, chartaceous to mildly corky, flaking readily in short to long, usually narrow and slightly sinuous to irregular, tabular shards, these usually remaining attached in several places with the spaces between detached, cracked and more or less raised, upper bark surface often with much secondary peeling and transverse cracking, crumbling in hand easily. *Branches* numerous, rather narrow and long, often weakly flexuose, in decumbent plants prostrate, trailing, otherwise initially ascending, soon suberect to widely spreading, and arching, often pendulous; branchlets numerous, very leafy, rather slender, quadrangular, sericeous, with dense, silky indumentum; hairs persistent, divergent, weakly flexuose, 0.03–0.06(–0.08) mm, hyaline to translucent (appearing white when young maturing grey), hair apices more or less straight. *Vegetative buds* inconspicuous, usually obscured by surrounding foliage; at resting stage 0.5–1.0(–1.6) mm diam.; scales scarious, deciduous, (0.3–)0.8(–1.3) mm long, red-brown to dark brown, initially broadly ovate grading through to broadly lanceolate; midrib prominent, strongly keeled, prolonged to cuspidate tip, with 1–2 lateral veins either side, and two prominent rows of 3–8 oil glands straddling midrib, margins and keel apex ciliate. *Leaves* heterophyllous, seedling and subadult leaves flat or involute, ± spreading to recurved; 0.9–3.0(–4.5) × 0.2–0.4(–0.6) mm, red-green or pale green suffused with red, rarely bright green; lamina finely linear-lanceolate, long persistent in stressed habitats (in damaged plants reversion shoots bearing juvenile foliage frequent); adult leaves ± spreading to patent; lamina (1.1–)4.0(–10.0) × (0.8–)1.3(–2.8) mm, dark glossy green, red-green, to bronze-green, narrowly oblanceolate, oblanceolate, obovate to obovate-rostrate; usually recurved from about half of total length, apex usually obtuse, rounded, rarely subacute, cuspidate; base attenuate; adaxial surface convex, finely glandular punctate; oil glands up to 590, more evident when dry, midrib slightly raised to depressed near base, otherwise depressed for entire length, glabrous, very rarely with fine antrorse hairs near base; abaxial surface slightly concave, finely glandular punctate, oil glands less obvious, up to 280, these more evident when dry; midrib depressed, finely and sparsely covered with sericeous, deciduous, antrorse-appressed hairs, these increasing in density toward base; lamina margin sparsely to densely, finely sericeous, hairy; hairs weakly flexuose, appressed to weakly spreading, antrorse to subantrorse, up to 0.1 mm, hyaline to translucent, appearing as white to naked eye, aligned in 1 row not quite meeting at cuspidate leaf apex. *Perules* scarious, basal ones usually persistent, these 0.4–1.0 mm long, pale brown to brown, broadly oblong to oblong-lanceolate, margins involute especially in upper third, midrib strongly keeled, prolonged as a cuspidate apex, with one row of 4–8 oil glands on each side of midrib, glabrous except for finely ciliate margin and apex; remaining perules deciduous, chartaceous, (0.6–)0.8(–1.4) mm long, pink to pinkish-white when fresh, drying apricot to apricot-brown, ovate to broadly oval, apex obtuse often appearing acute due to apical infolding, ± cuspidate, glabrous except for sparsely ciliate margin, strongly keeled, keel ± prolonged. *Inflorescence* usually a compact, (1–)6(–10)-flowered corymbiform botryum up to 25 mm long, borne on alternate brachyblasts up to 15 mm long, with those near branchlet apex usually subopposite; inflorescences at the ultimate branchlet tips rarely elongated, in which case these are invariably surmounted with terminal vegetative growth. Inflorescence axis densely invested with divergent hairs. *Pherophylls* deciduous (falling very early), tightly clasping pedicels to ± spreading, 0.5–1.0 mm long, initially foliose soon squamiform; foliose pherophylls pale green, oblong, oblong-obovate to oblanceolate, margins and apex finely ciliate; squamiform pherophylls brown or pink, drying apricot-brown or amber, broadly deltoid to oblong-ovate, margins involute especially in upper one-third, midrib strongly keeled, prolonged as cuspidate apex, with one row of 4–8 oil glands on each side; glabrous except for the finely ciliate margin and apex; similar to perules in size and shape at apex. *Pedicels* (1.0–)2.1(–2.4) mm long at anthesis, elongating slightly after anthesis, terete, copiously invested in slightly flexuose, antrorse to subantrorse sericeous hairs. *Flower buds* clavate to pyriform, apex distinctly domed (due to thickened calyx lobes) prior to bud burst with calyx valves ± meeting. Fresh flowers when fully expanded (3.3–)5.5(–9.0) mm diam. *Hypanthium* (1.8–)2.5(–3.3) × (1.7–)2.4(–3.1) mm, with free portion 0.3–0.8(–1.0) mm long, dark green often basally mottled or flushed with red when fresh, drying brown to grey; narrowly cupular to campanulate terminating in a slightly thicker rim bearing five persistent calyx lobes; surface smooth, finely gland-dotted, and puberulent, with weakly defined ridges leading up to calyx lobes (these becoming more distinct upon drying); hairs shortly subantrorse to antrorse. Calyx lobes 5, upright (not spreading), firmly fleshy, 0.4(–0.8) × 0.4(–1.0) mm, persistent, oblong, oblong-ovate to broadly triangular, in longitudinal-section distinctly thicker at base, ± subtended by a faint to prominent groove at the external junction with the hypanthium, otherwise tapering to apex, scarcely keeled (the keel if evident recognisable as a darker green or pink, thicker central prolongation of the hypanthium ridges), margins cream to pale pink, gland-dotted, oil glands usually colourless sometimes pink; otherwise glabrate except for ciliate margins; cilia widely spreading. Receptacle green or pale pink at anthesis, darkening to crimson-red or magenta after fertilisation. *Petals* 5(–6), 1.4–1.6(–2.0) × 1.4–1.6(–2.0) mm, white or pinkish white, usually basally flushed pink, very rarely completely pink, orbicular, sometimes cuneate, apex obtuse to rotund, margins plane or finely crimped 3–12 times, oil glands not evident when fresh, drying colourless. *Stamens* 10–24(–32) in 1(–2) weakly defined whorls, arising from receptacular rim, filaments white often tinged rose-pink toward base. Antipetalous stamens 2(–3), antisepalous 1(–4). Outermost antipetalous stamens usually weakly to strongly incurved, on filaments 0.9–2.2 mm long; inner stamen, if present, 0.6–0.8 mm, strongly incurved; very rarely a further 1–2 strongly incurved stamens, 0.4–0.7 mm long, may be present at the base of the outermost antipetalous pair. Antisepalous stamens much shorter than outermost antipetalous stamens, 0.3–0.8 mm, incurved, rarely outcurved or in mixtures of both. Anthers dorsifixed, 0.04–0.08 × 0.02–0.04 mm, testiculate, latrorse. Pollen white (12.8–)14.7(–16.6) μm. Anther connective gland prominent, orange when fresh, drying pale brown, spheroidal, distinctly papillate. *Ovary* (3–)4(–5) locular, each with 15–18(–22) ovules in two rows on each placental lobe. Style 2.0–2.6(–3.6) mm long at anthesis, often elongating slightly after anthesis, white basally flushed with pink; stigma capitate, scarcely wider than style, domed along margins with a central depression, pale cream to pink, surface papillate to distinctly rugulose. *Fruits* ± persistent, (1.0–)2.3(–3.3) × (1.6–)2.2(–3.2) mm, light brown to grey, usually barrel-shaped, rarely cupular, splits concealed by dried, suberect to erect, free portion of hypanthium. *Seeds* 0.80–1.00 × 0.45–0.50 mm, narrowly oblong, oblong, oblong-obovate to falcate-oblong, curved near apex, laterally compressed, 2–3-angled with convex to flattened faces, apex rounded, base cuneate to oblique, ± flattened; testa semi-glossy, orange-brown, surface coarsely reticulate. FL: (Aug–)Sep–Oct(–Mar) FT: Jan–May(–Nov). Chromosome Number *n* = 11_II_, 2*n* = 22 (de Lange and Murray 2004).

#### Representative specimens

**(80 sheets seen).** **New Zealand (North Island).** Kawerau, Ruruanga Stream (Parimahana Geothermal Field), P. J. de Lange 4628, 7 Nov 2000, (K 288085); Tikitere (Hell’s Gate) Geothermal Field, Upper Waiohewa Stream, P. J. de Lange 4628, 7 Nov 2000, (AK 288086); Rotorua, Kuirau Park, P. J. de Lange 4627, 7 Nov 2000, (AK 286156); Whakarewarewa Park, L. Cockayne s.n., 29 Dec 1905, (WELT SP029450); Waimangu Thermal Area, near Lake Rotomahana, R. J. Chinnock s.n., 17 Oct 1967, (WELTU 9731); Maungakakaramea (Rainbow Mountain), P. J. de Lange 4223, 26 Jan 2000, (AK 286186); Te Kopia Geothermal Area, P. J. de Lange 4700, 16 Nov 2000, (AK 288099, Duplicates: AD, MEL, MO); Paeroa Range, Waikite Geothermal Reserve, P. J. de Lange 4713 & R. O. Gardner, 19 Nov 2000, (AK 286168, Duplicates: AD, WELT); Te Kopia – Waihunuhunu Road, Waihunuhunu Stream, P. J. de Lange 4699, 16 Nov 2000, (AK 288087, Duplicates: AD, FI, HO, MSC, P); Waikato River, Lake Ohakuri, Orakeikorako, P. J. de Lange 4693, 16 Nov 2000, (AK 286170); Waikato River, Wairakei, P. J. de Lange 4683, 10 Nov 2000, (AK 288084, Duplicate: AD); Wairakei Geothermal Field, Karapiti, Craters of the Moon, P. J. de Lange 5765, 10 Nov 2003, (AK 286152, Duplicates: CANB, CANU, MSC, NSW, Z); Lake Taupo, The Spa, D. Petrie s.n., Dec 1895, (WELT SP029561); Tokaanu, Tokaanu Geothermal Reserve, P. J. de Lange 4582, 19 Oct 2000, (AK 288103, Duplicates: AD, HO, MEL, WELT).

#### Distribution

**Figure 21.**
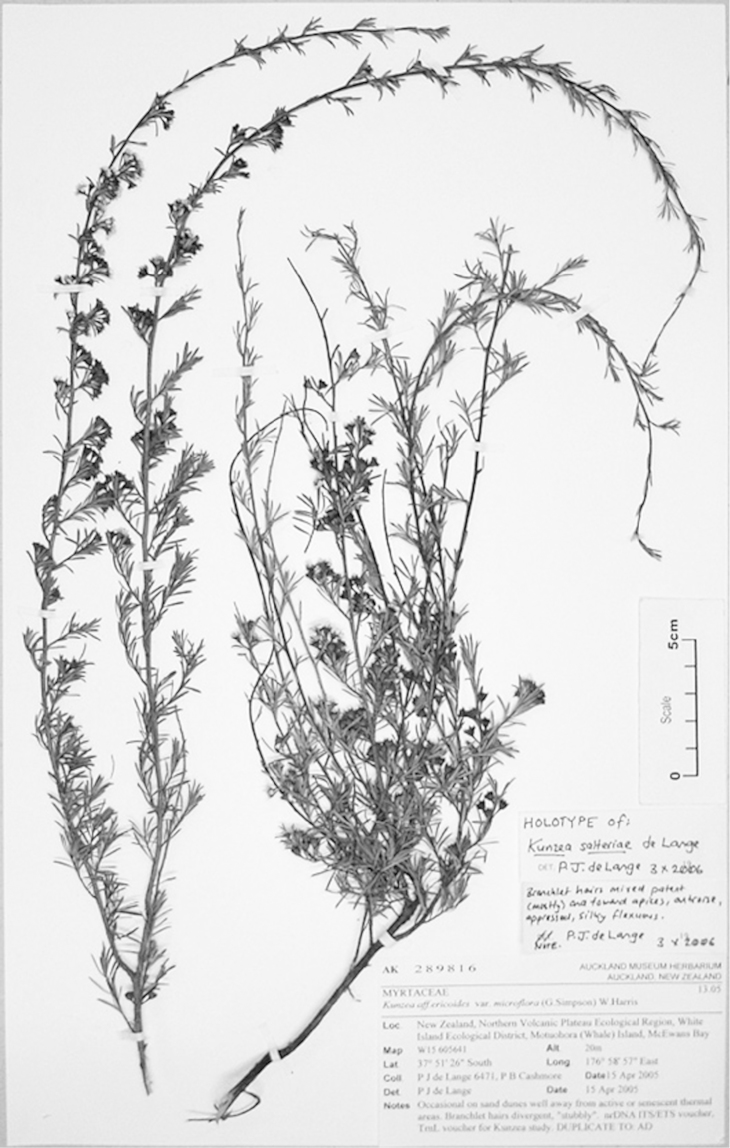
Holotype of *Kunzea salterae* (P. J. de Lange 6471 & P. B. Cashmore, AK 289816).

**(Fig. 7).** Endemic, New Zealand, North Island, Bay of Plenty to the Central North Island (40–580 m a.s.l.). Confined to active geothermal fields (i.e. those with surface expression) of the Taupo Volcanic Zone (for geology see Healy 1992; Houghton et al. 1995; Wilson et al. 1995; Neall 2001) from the vicinity of Kawerau (Parimahana Geothermal Field) and Lake Rotoiti (Tikitere) south to Tokaanu and the hills above Waaihi, Lake Taupo (Figs 20A–C, 21).

#### Recognition.

*Kunzea tenuicaulis* is recognised by a combination of growth habit, branchlet hair and floral characters (Figs 19, 20; see also Table 1) supplemented by cytological and molecular differences. The ITS and ETS sequence data (Table 2) show that *Kunzea tenuicaulis* is the most diverged of the New Zealand members of *Kunzea ericoides* complex (de Lange 2007; de Lange et al. 2010). Uniquely within the *Kunzea ericoides* complex, the ITS sequence of *Kunzea tenuicaulis* possesses two adenine nucleotides rather than the guanine common to all other members of the complex at ITS-1 alignment position 639, and at ITS-2 alignment position 994 (Table 2; see also de Lange 2007). Otherwise it shares a guanine/thiamine mix with *Kunzea salterae* (de Lange 2007). The ETS sequence (Table 2) showed two further unique characters; a thiamine at alignment position 18 (whereas all other members of the complex possess a cytosine), and a cytosine at alignment position 202 (whereas all other members of the complex possess an adenine) (de Lange 2007). Otherwise the aligned ETS sequence of *Kunzea tenuicaulis* has an adenine at position 269 in common with the other ‘small-leaved’ New Zealand *Kunzea*, *Kunzea salterae*, *Kunzea serotina* and *Kunzea toelkenii* (de Lange 2007). In view of the geologically recent (estimated to be a maximum of 2 million years old (Neall 2001; Briggs et al. 2005)) habitats this species occupies, this molecular divergence from all other members of the *Kunzea ericoides* complex is considered remarkable (de Lange 2007; de Lange et al. 2010).

*Kunzea tenuicaulis* seems to be most closely allied to *Kunzea salterae* and *Kunzea serotina*, and, based on the results obtained from experimental hybridisations (de Lange et al. 2005), *Kunzea tenuicaulis* may have had a role in the evolution of *Kunzea salterae* and *Kunzea toelkenii* through hybridisation with *Kunzea linearis* and *Kunzea robusta*. With the exception of *Kunzea salterae* which has linear-lanceolate leaves, *Kunzea serotina*, *Kunzea tenuicaulis* and *Kunzea toelkenii* all possess small oblanceolate to obovate leaves. Branchlet hairs in all four species tend to be copious, short (up to 0.08 mm), divergent, and persistent (Table 1). Further, as reported by de Lange and Murray (2004) and de Lange et al. (2005) *Kunzea tenuicaulis* shares with *Kunzea serotina* and *Kunzea toelkenii* uniformly small chromosomes (0.9–1.0 μm). *Kunzea salterae*, has a similar chromosome complement, though this was not reported previously because that species had not yet been recognised when those papers were published. Despite this Genomic *In Situ* Hybridisation experiments determined that, alone of those taxa analysed, *Kunzea tenuicaulis* has the most diverged genome (de Lange et al. 2005).

As circumscribed here, *Kunzea tenuicaulis* includes a range of prostrate to erect plants found in close proximity to active geothermal vents and fields (Fig. 20D–G, J). Although in the past, (probably because of the past poor circumscription of this plant) field workers had formed the impression that Kunzea ericoides var. microflorum applied only to the flat, prostrate to small decumbent shrubs found near active geothermal vents (e.g., Harris et al. 1992; Harris 1996; Smale 1994). There seems little point in trying to separate these prostrate plants from the erect plants they resemble in all respects except stature. Indeed, stature itself often presents as a gradient from thermally heated to thermally quiescent ground. Cultivation experiments showed that while some seedlings raised from seed sampled from prostrate/decumbent plants retained that growth habit, the majority grew into multi-trunked erect small trees with flat topped, spreading to pendulous crowns typical of *Kunzea tenuicaulis* as defined here. Irrespective of growth habit, all forms of *Kunzea tenuicaulis* are consistently unified by their many, fine, slender branchlets; copious, short, divergent branchlet hairs (Fig. 19A–F); small oblanceolate to obovate leaves (Fig. 18C–G); small cupular to campanulate, finely puberulent hypanthium (Fig. 18K–L); by the calyx lobes which are thickened toward the base and there subtended by a faint to prominent groove along the external junction with the hypanthium; and by the smaller, barrel-shaped fruit (Fig. 18O). Furthermore the ITS and ETS sequence data obtained from multiple samples spanning the range and variation of this species were consistent, and readily distinguished *Kunzea tenuicaulis* from the rest of the *Kunzea ericoides* complex (de Lange 2007; de Lange et al. 2005).

Provided that care is taken to note the growth habit, collect old bark, new season’s growth, and emergent flowers *Kunzea tenuicaulis* is easily separated from the other New Zealand *Kunzea*. This is important for although *Kunzea tenuicaulis* is distinguished ecologically because it is endemic to active geothermal fields (Figs 20A–C, 21), within these habitats it may be found sympatric (or even syntopic) on associated ‘cool’ sites with *Kunzea serotina* and *Kunzea robusta*.

*Kunzea tenuicaulis* can be distinguished from *Kunzea serotina* by its growth habit, which is either prostrate/decumbent or multi-trunked, with widely spreading pendulous, mostly flat-topped, branches, producing numerous spreading, slender, long branchlets (Fig. 20D–G, J; Table 1). This contrasts with the strictly upright columnar to pyramidal growth habit, with short, obliquely ascending, fastigiate branches of *Kunzea serotina*. The bark of both species is also diagnostic. The old bark of *Kunzea tenuicaulis* is grey-brown to brown, readily detached, somewhat corky-chartaceous, flaking in narrow, short to long, slightly sinuous to irregular, tabular shards (Fig. 20H–I; Table 1). The old bark of *Kunzea serotina* is greyish-white to pinkish-white, and presents as inrolled and curled ‘wood shavings’, these having little if any discernible shape, with highly irregular, sinuous, often frayed margins. Although both species have similar leaves, in *Kunzea serotina* the leaves are less widely spaced, and tend to be densely clustered around the branchlets. Branchlet hairs however are not overly diagnostic as both species have similarly sized divergent hairs. In general though, those of *Kunzea serotina* tend to have more curly apices and *Kunzea tenuicaulis* less so (Fig. 19C–F). The pherophylls, if present, serve to separate both species: those of *Kunzea tenuicaulis* are mostly foliose oblong, oblong-obovate to oblanceolate while those of *Kunzea serotina* are characteristically spathulate to spathulate-orbicular. The calyx lobes of *Kunzea tenuicaulis* are especially diagnostic when fresh because they are distinctly thickened toward the base below which is a faint to prominent groove at the external junction with the hypanthium. Those of *Kunzea serotina* are slightly keeled and flush with the rest of hypanthium. The petals of *Kunzea tenuicaulis* are broadly orbicular and have colourless oil glands (often not evident until the petals have dried) while those of *Kunzea serotina* are narrowly orbicular to broadly ovate and typically have pale yellow oil glands. The mature fruits of *Kunzea tenuicaulis* tend to be barrel-shaped to cupular, those of *Kunzea serotina* urceolate to campanulate or, rarely, cupular (Table 1).

*Kunzea tenuicaulis* is distinguished from *Kunzea robusta* by its smaller size (up to 8 m cf. up to 30 m in *Kunzea robusta*) and growth habit (see Table 1). *Kunzea robusta* is mostly an arborescent species, and so usually forms a single-trunked tall tree, with a broad trunk, stout, ascending to spreading branches, and a very wide, spreading, multi-tiered canopy. However, occasionally *Kunzea robusta* can be a low (up to 2 m tall) shrub with prostrate to pendulous branches, or be a tree with entirely pendulous branches, in which case leaf size, shape and branchlet hair type serve to distinguish it from *Kunzea tenuicaulis*. The bark of *Kunzea robusta* is particularly distinctive, being very coriaceous, long persistent, typically detaching with age as long (up to 4 m), broad to narrowly tabular strips, with ± smooth, ± entire margins, which when deliberately bent and snapped have ± entire or weakly frayed broken surfaces. In contrast, the bark of *Kunzea tenuicaulis* is not long persistent, readily detaches, and is distinctly corky-chartaceous, flaking as rather narrow, much shorter (up to 100 mm long) tabular shards with slightly sinuous to irregular margins. The oblanceolate to lanceolate leaves of *Kunzea robusta*, up to 20.1 × 3.0 mm, are usually much larger and broader than the leaves of *Kunzea tenuicaulis* which grow to 4.5 × 0.6 mm. A key difference between these species is the branchlet hairs. In *Kunzea robusta* populations that occur within the range of *Kunzea tenuicaulis*, branchlet hairs are mostly antrorse-appressed, larger (up to 0.38 mm) and straight to weakly flexuose. *Kunzea robusta* also has larger inflorescences containing more flowers (up to 30, more usually 12) than *Kunzea tenuicaulis*. The inflorescences of *Kunzea robusta* typically progress from a compact corymbiform botryum at the onset of flowering to an elongated botryum as the flowering season progresses due to activation of the apical vegetative bud. In *Kunzea tenuicaulis* this very rarely happens, and then only to the terminal inflorescence, those of the brachyblasts tending to remain as compact corymbiform botrya bearing far fewer (up to 10, more usually six) flowers (Figs 18A, 20K). The flowers of *Kunzea robusta* tend to have a greater overall diameter (up to 12.0 mm, more usually 7.7 mm cf. up to 9.9 mm, more usually 5.5 mm) and in the field more stamens (up to 60 cf. up to 32 in *Kunzea tenuicaulis*) (see Table 1). The fruits of both species are also rather different, those of *Kunzea tenuicaulis* tending to be smaller (up to 3.3 × 3.2 mm )and barrel-shaped to cupular (Fig. 18B, O), while those of *Kunzea robusta* are much larger (up to 4.6 × 5.3 mm), and mostly obconic, broadly obconic to ± turbinate (see Table 1).

Although *Kunzea salterae* and *Kunzea toelkenii* are allopatric from *Kunzea tenuicaulis*, they could be confused in the herbarium. The oblanceolate to obovate leaves of *Kunzea tenuicaulis*, which grow up to 4.5 × 0.6 mm, are smaller than the 18 × 2 mm, linear-lanceolate to narrowly oblanceolate leaves of *Kunzea salterae*. Furthermore, the fruits of *Kunzea tenuicaulis* are barrel-shaped to cupular rather than cupular to subcampanulate (see Table 1). The consistently divergent branchlet hairs of *Kunzea tenuicaulis* are distinct from the admixed large, antrorse-appressed, weakly flexuose, and small, divergent, curled hairs of *Kunzea toelkenii*. The leaves of *Kunzea tenuicaulis* are also smaller than those of *Kunzea toelkenii*, which grow to 8.5 × 2.5 mm, and *Kunzea tenuicaulis* lacks functionally male late season flowers, unlike *Kunzea toelkenii* (see Table 1).

#### Ecology.

*Kunzea tenuicaulis* is the dominant woody plant on the active geothermal fields within the Taupo Volcanic Zone (Fig. 20A–C) where it colonises not only heated ground but quiescent and/or ‘cool’ ground associated with each geothermal field. In these ‘cool’ peripheral situations it may be dominant, though it usually associates with *Leptospermum scoparium*, *Weinmannia racemosa* L.f., *Knightia excelsa* R.Br. and *Kunzea robusta*. The understorey of this peripheral vegetation is usually dominated by shrubs and ferns such as *Leptecophylla juniperina* (J.R.Forst. et G.Forst.) C.M.Weiller, *Leucopogon fasciculatus* (G.Forst.) A.Rich., *Dracophyllum subulatum* Hook.f., *Pteridium esculentum* (G.Forst.) Cockayne, *Histiopteris incisa* (Thunb.) J.Sm., and *Lycopodiella cernua* (L.) Pic.Serm. Toward the active geothermal vents, where surface temperatures can abruptly rise to as much as 90 °C (Burns 1997) and most woody vegetation becomes scarce, *Kunzea tenuicaulis* is the dominant macro-vegetation. In these habitats, which may include extensive areas of steam field, heated mud pools and hot springs, active and quiescent hydrothermal explosion craters, and fumaroles, the low shrub or prostrate trailing form of *Kunzea tenuicaulis* is best developed (Fig. 20A, C, J). It is this form which may be found flowering at less than 40 mm tall and which is the plant referred to in past literature as Kunzea ericoides var. microflora (see Given 1980a; Harris et al. 1992; Smale 1994; Harris 1996; Burns 1997). However, in many geothermal areas (e.g., Tikitere, Kuiarau Park andTokaanu) this decumbent form of *Kunzea tenuicaulis* is mostly replaced by multi-trunked, erect to suberect trees of *Kunzea tenuicaulis* (see Fig. 20D–G) identical to those seen growing in peripheral ‘cold’ and/or thermally quiescent areas in more active fields. Further study is needed to determine why the low shrub form of *Kunzea tenuicaulis* seems to be favoured in the more unstable geothermal systems, though physiological stress and aluminium toxicity has been suggested as a partial explanation (Burns 1997). Irrespective, in these geothermally more active habitats *Kunzea tenuicaulis* frequently associates with the ferns *Dicranopteris linearis* (Burm.f.) Underw., *Nephrolepis flexuosa* Colenso, *Cheilanthes distans* (R.Br.) Mett. and Cheilanthes sieberi Kunze subsp. sieberi, sparse, stunted *Leucopogon fasciculatus* and *Dracophyllum subulatum* shrubs, the lilies *Dianella nigra* Colenso, *Dianella haematica* Heenan et de Lange and the exotic love grass *Eragrostis brownii* (Kunth) Wight. Underneath *Kunzea tenuicaulis* shrubs a ground cover of liverworts and mosses dominated by *Chiloscyphus semiteres* (Lehm.) Lehm. and *Campylopus pyriformis* (Schultz) Brid. is usually present. In these habitats and also in the peripheral cool soils, ectomycorrhizal fungi of the genus *Pisolithus* Alb. et Schwein. (see Burns 1997; Moyersoen and Beever 2004) have been found in exclusive association with *Kunzea tenuicaulis* (McKenzie et al. 2006 as Kunzea ericoides var. microflora). *Kunzea tenuicaulis* is also occasionally, and at times rather heavily, parasitised by the dwarf mistletoe *Korthalsella salicornioides*.

#### Hybridism.

The putative hybrids *Kunzea robusta* × *Kunzea tenuicaulis* and *Kunzea serotina* × *Kunzea tenuicaulis* have been collected throughout the range of *Kunzea tenuicaulis*. However, of these hybrids, only *Kunzea robusta* × *Kunzea tenuicaulis* is commonly encountered, because *Kunzea robusta* is more frequently sympatric with *Kunzea tenuicaulis* along the margins of that species’ geothermal habitats, and in the plantation forests abutting many of the geothermal fields within the Rotorua Volcanic Centre (for geology see Briggs et al. 2005; Neall 2001). Putative gatherings of *Kunzea serotina* × *Kunzea tenuicaulis* are less common because the ranges of those species rarely overlap (except around Karapiti and Tokaanu). These hybrids are discussed in detail under *Kunzea serotina*.

Hybrids involving *Kunzea robusta* are best recognised by the presence of mixtures of long, appressed, weakly flexuose and shortly divergent branchlet hairs. However, in the field they can be distinguished by their general tendency to form single trunked, weakly spreading trees, with fewer branches and branchlets that are often somewhat slender and semi-pendulous to pendulous. *Kunzea robusta* × *Kunzea tenuicaulis* is usually present as introgressive hybrid swarms because most of the geothermal habitats are now extensively modified.

Artificial hybrids involving *Kunzea tenuicaulis* as staminate or pistillate parent and other New Zealand members of the *Kunzea ericoides* complex were easily produced, and showed no obvious reduction in fertility, except for crosses involving *Kunzea serotina*, which were sterile (de Lange and Murray 2004; de Lange et al. 2005).

#### Vernacular name.

No specific Maori name for this species seems to have been recorded.

#### Conservation status.

Currently, as Kunzea ericoides var. microflora, *Kunzea tenuicaulis* is appropriately listed as ‘At Risk/Naturally Uncommon’ qualified ‘RR’ [Range Restricted] by de Lange et al. (2013b).

### 4. Kunzea salterae

Taxon classificationPlantaeMyrtalesMyrtaceae

de Lange sp. nov.

urn:lsid:ipni.org:names:77141729-1

A K. tenuicauli foliis constanter longioribus angustioribus lineo-lanceaceolatis, hypanthio maiore glabrato anguste obconico vel infundibuliforme, stigmate plano anguste capitato, lobisque antherae profunde sulcatis non testiculatis differt.

#### Holotypus

**(Fig. 21).** **New Zealand:** North Island, Bay of Plenty, Moutohora (Whale Island), McEwans Bay, 37°51'26"S, 176°58'57"E, 20m a.s.l. ‘Occasional on sand dunes well away from active or senescent thermal areas’. P. J. de Lange 6471 & P. B. Cashmore, 15 Apr 2005, AK 289816! Isotype: AD!

#### Etymology.

The specific epithet *salterae* commemorates my colleague and botanical illustrator for this monograph Josh Salter (1946–), whose critical attention to detail when illustrating specimens of *Kunzea salterae* proved invaluable in deciding on an appropriate taxonomic rank.

#### Description

**Figure 22.**
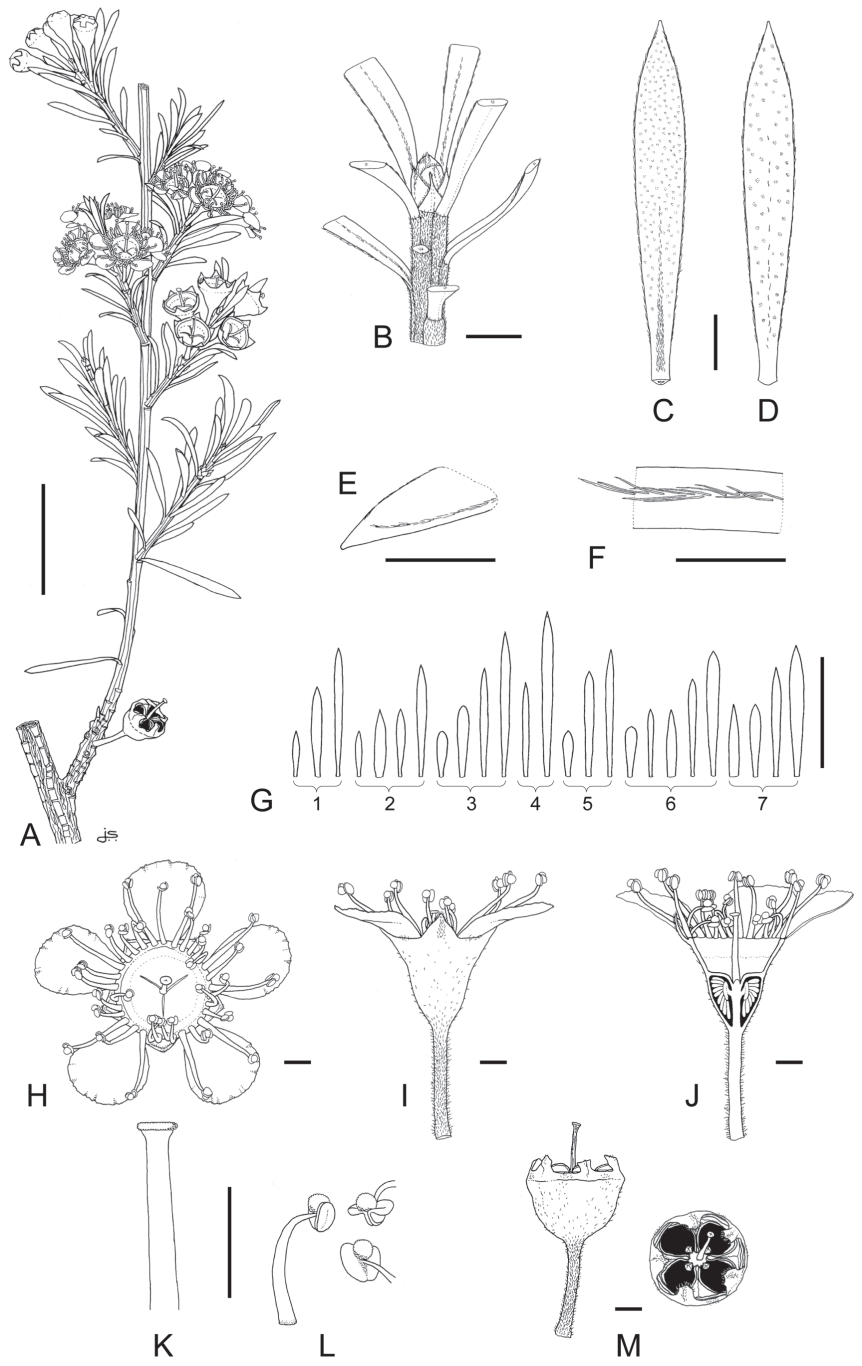
Distinguishing features of *Kunzea salterae*. **A** Flowering branchlet (AK 289816) **B** Vegetative bud and branchlet indumentum (AK 289816) **C** Adaxial leaf surface (AK 289816) **D** Abaxial leaf surface (AK 289816) **E** Adaxial leaf apex (AK 289816) **F** Leaf margin indumentum (AK 289816) **G** Leaf variation, all from Moutohora (Whale Island): (**G1**) (AK 185215), (**G2**) Boulder Bay (AK 288250), (**G3–5**) Sulphur Bay (AK 284105, AK 283253, AK 289814), (**G6**) Summit Hill Saddle (AK 289815), McEwans Bay (AK 289816) **H** Flower (top view) (AK 289816) **I** Flower and hypanthium (side view) (AK 289816) **J** Flower cross section showing anther, style and ovules (AK 289816) **K** Style and stigma (AK 289816) **L** Stamens (AK 289816) **M** Dehisced fruit (AK 289816). Scale bars: (**A, G**) 10 mm; (**B–E, H–M**) 1 mm; (**F**) 0.5 mm.

**Figure 23.**
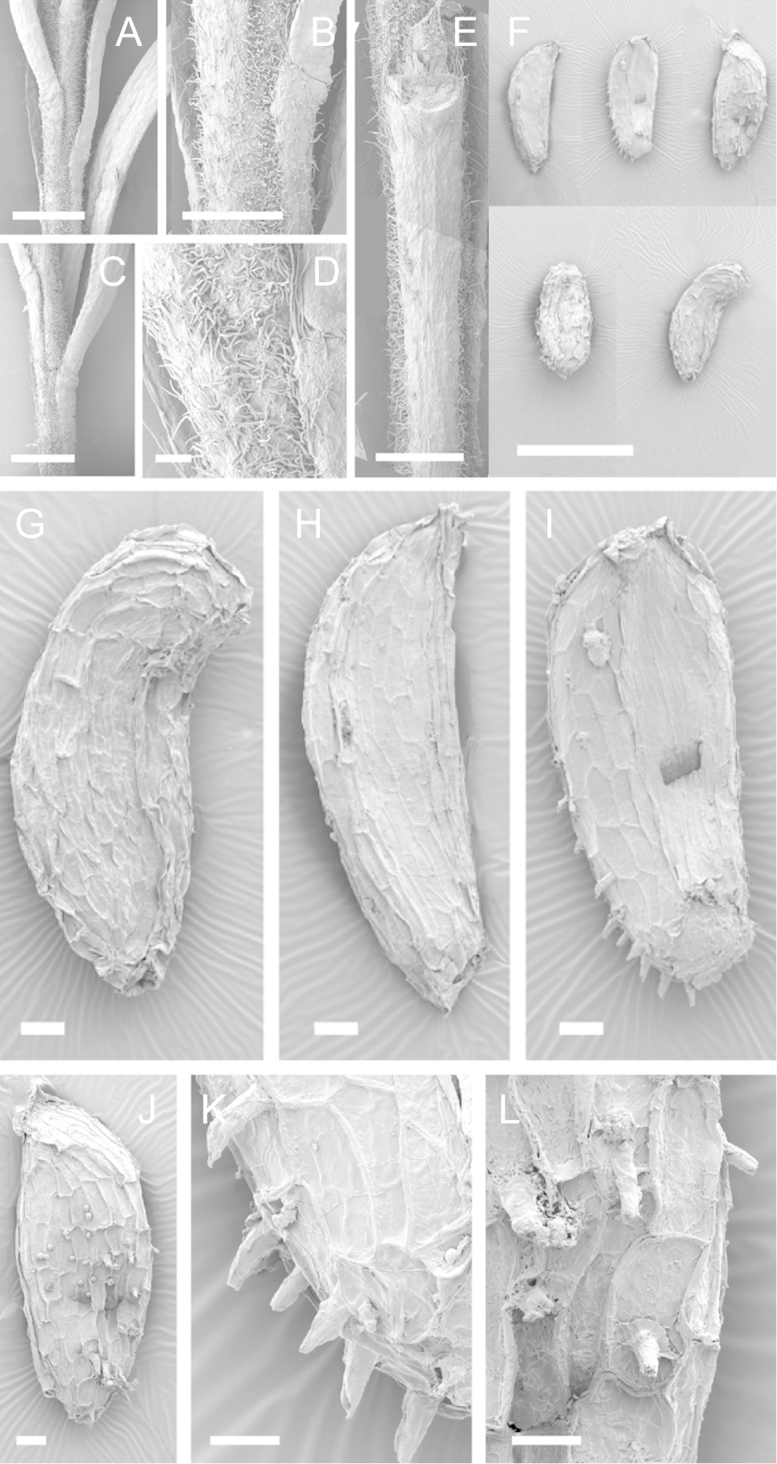
Scanning Electron Micrographs of *Kunzea salterae*. (**A–E** all AK 284105) Branchlet indumentum **F–L** Seeds (AK 283253, AK 289815) **K–L** Close up of reticulum showing spines. Scale bars: (**A, C, F**) 1 mm; (**B, E**) 500 μm; (**D, F–J**) 100 μm; (**K, L**) 50 μm.

**Figure 24.**
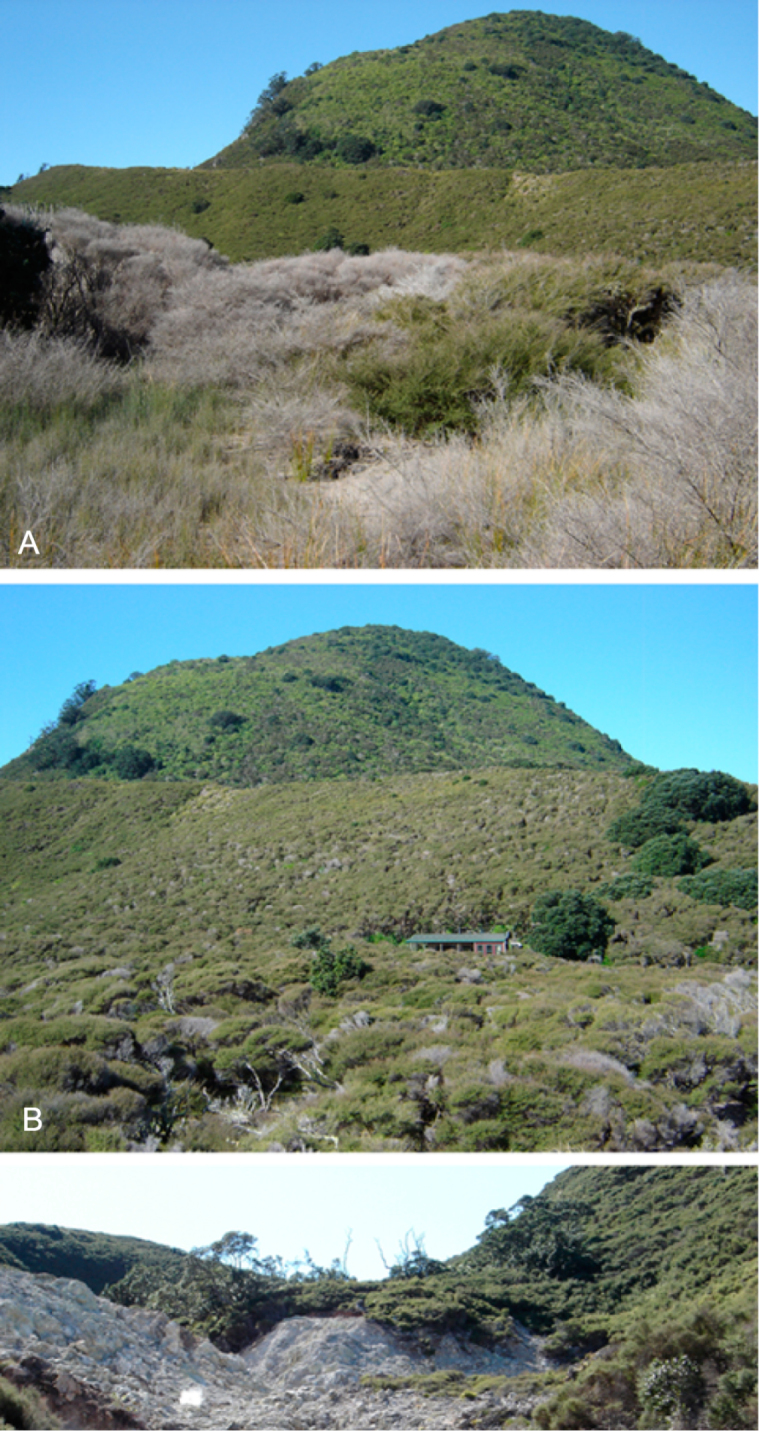
Habitats of *Kunzea salterae* on Moutohora Island (photos: *P. B. Cashmore*). **A** Sand dunes and early stage successional forest leading to Summit Hill **B** Stable sand dunes and early stage forest surrounding Department of Conservation Hut and ride line leading to Summit Hill **C** Active geothermal vents within Sulphur Valley.

**(Figs 22, 23, 24).** *Growth habit* shrubs to small trees 0.1–6(–10) × 2–4(–6) m with broad, spreading to somewhat pendulous crowns, rarely plants completely decumbent, sprawling across ground. *Trunk* usually multi-trunked from base, up to 0.3 m d.b.h., these mostly widely spreading to suberect, flexuose, often basally buttressed, branches frequent from base in exposed sites, otherwise naturally thinning in the lower half of the trunk. *Bark* early bark brown, initially firm, somewhat sinuous-fluted, elongate, over time cracking transversely (especially on branch flanges), and with apices gradually detaching and raising to present as small lunate (in profile) flakes, old grey-brown bark flaking readily in small, somewhat irregular tabular shards, often with small lunate secondary peeling; somewhat corky to chartaceous. *Branches* Two to many, suberect to widely spreading, rarely ascending, mostly pendulous, branchlets numerous and very leafy, rather slender, initially subterete soon becoming quadrangular; sericeous, indumentum initially copious rarely glabrate to glabrous, hairs on young rapidly growing apices, copious, sericeous, straight, antrorse-appressed up to 0.55 mm, these soon falling; other mostly divergent hairs long persistent, (especially opposite leaf buds and expanding foliage), 0.04–0.08(–0.1) mm, hyaline to translucent (appearing white when young maturing grey), apices ± curled, often admixed (particularly toward branchlet apices and near decurrent leaf bases) with deciduous antrorse-appressed, straight to somewhat sinuous hairs up to 0.28 mm. *Vegetative buds* inconspicuous at resting stage 0.5–1.0 mm diam.; scales deciduous; (0.6–)1.2–2.3 mm long, stramineous to pale brown, initially broadly ovate to ovate-lanceolate grading through broadly lanceolate to narrowly lanceolate, midrib strongly keeled, prolonged to apiculate tip, with one prominent row of 4–10 oil glands on either side of midrib, margins, apex, apiculus and keel finely ciliate. *Leaves* ± spreading to patent; lamina (4–)10(–18) × (0.6–)1.2(–2.0) mm, bright glossy green, yellow-green, bronze-green to dark green; linear-lanceolate to narrowly oblanceolate, flat not recurved, apex acute to subacute, cuspidate, rarely obtuse to rounded; base attenuate; adaxial surface slightly concave to flat, finely glandular punctate; oil glands 180(–280), more evident when dry; midrib slightly raised to depressed near base otherwise depressed for entire length, initially densely covered in fine, antrorse-appressed silky hairs up to 0.22 mm, becoming glabrescent; abaxial surface slightly convex, finely glandular punctate, oil glands less obvious when fresh than when dry, up to 100, with the larger glands aligned longitudinally along midrib; midrib slightly raised, usually glabrous, sometimes with a fine weft of silky, deciduous, antrorse-appressed hairs near base; lamina margin sparsely to densely, finely sericeous, hairs mostly antrorse-appressed, up to 0.5 mm, hyaline to translucent, appearing as white to naked eye; hairs in 1–2 somewhat irregular rows just failing to meet short of cuspidate leaf apex. *Perules* scarious, basal ones usually persistent, 1.2–1.4 mm long, stramineous to brown, broadly to narrowly lanceolate, involute, midrib strongly keeled prolonged as a cuspidate apex, with one row of 4–8 oil glands on either side of midrib, lower two-thirds glabrous, upper one-third finely ciliate; remaining perules deciduous, chartaceous, 0.6–1.4 mm long, pale pink to pinkish-white when fresh, drying apricot to apricot-pink, broadly oval, ovate to rhomboid, finely and copiously ciliate, strongly keeled, keel prolonged, apiculate, margins and keel more distinctly ciliate. *Inflorescence* a (2–)4(–8)-flowered corymbiform botryum up to 45 mm long, usually on brachyblasts, rarely on long shoots in which case invariably terminal (only very rarely with terminal vegetative growth). Inflorescence axis densely invested with mostly divergent hairs. *Pherophylls* deciduous (falling very early), mostly squamiform, rarely foliose, spreading, 0.6–1.8 mm long; squamiform pherophylls brown or amber, sometimes pink, drying apricot-brown, broadly deltoid to oblong-ovate, margins involute especially in upper one-third, midrib strongly keeled prolonged as cuspidate apex, with one row of 4–8 oil glands on each side of midrib; glabrous except for the finely ciliate margin and apex; foliose pherophylls bright green, linear, margins and apex finely ciliate; both types grading into chartaceous, into perules and/or leaves at inflorescence terminus. *Pedicels* (1.1–)2.6(–3.0) mm long at anthesis and elongating slightly after anthesis, terete, finely invested in divergent to subantrorse sericeous hairs. *Flower buds* pyriform to clavate, apex domed with calyx valves not or scarcely meeting. Fresh flowers when fully expanded up (9–)10(–12) mm diam. *Hypanthium* (2.1–)2.2(–3.8) × (1.8–)2.2(–3.2) mm, with free portion 1.0–1.6 mm long, reddish-brown when fresh, drying resinous brown to grey; narrowly obconic to funnelform terminating in a slightly thicker rim bearing five persistent calyx lobes; surface smooth, finely glandular punctate, sparsely hairy to glabrate, with five rather weakly defined ridges leading up to calyx lobes (these becoming more distinct upon drying); hairs scattered, subantrorse to antrorse, flexuose. Calyx lobes 5, upright (not spreading), 0.6(–0.9) × 1.1(–1.3) mm, persistent, broadly to narrowly triangular, weakly and broadly keeled (the keel though ill-defined in fresh specimens recognisable as a dark pink to red, thicker central prolongation of the hypanthium ridges), margins cream to pale yellow, gland-dotted, subcoriaceous, glabrate except for distinctly ciliate apex. Receptacle dark red at anthesis. *Petals* 5, spreading, 1.4–1.6 × 1.4–1.6 mm, white, rarely basally flushed pink, orbicular to suborbicular, apex obtuse to rotund, margins usually finely crimped, oil glands colourless or rose-pink, scarcely evident when fresh. *Stamens* 28–36(–38) in 1–2 weakly defined whorls, adnate to receptacular rim, filaments white rarely tinged rose-pink toward base. Antipetalous stamens 3(–5) antisepalous 3(–4). Outermost antipetalous stamens strongly outcurved, on filaments 2.5–3.25 mm long, inner stamen 1.8–2.2 mm, outcurved, on occasion a further 1–2 incurved or outcurved, stamens 0.8–1.0 mm long, positioned at the base of the outermost antipetalous pair. Antisepalous stamens much shorter than antipetalous, 0.6–0.9(–1) mm, incurved, outcurved or in mixtures of both. Anthers dorsifixed, 0.11–0.16 × 0.10–0.14 mm, scutiform to ovoid, latrorse, each anther deeply and longitudinally furrowed, with one anther lobe in each pair fused at right angles along inner margin with adjoining anther lobe to form a prominent ‘pinched’ longitudinal ridge. Pollen white, (10.2–)14.7(–16.6) μm. Anther connective gland prominent, pale orange to pink when fresh, drying orange-brown, spheroidal, finely papillate, somewhat farinose. *Ovary* (3–)4 locular, each locule with 8–10 ovules in two rows on each placental lobe. Style 2.1–3.2 mm long at anthesis, white basally flushed with pink; stigma capitate, up to 1× style diam., flat, abruptly broadened, pale cream, finely papillate rugulose. *Fruits* rarely persistent, (2.0–)2.2(–2.7) × (2.0–)2.9(–4.0) mm, light brown to grey, cupular to suburceolate, splits concealed by dried, erect, free portion of hypanthium. *Seeds* 0.80–1.00 × 0.45–0.48 mm, narrowly oblong, oblong, oblong-obovate to falcate-oblong or elliptic, curved near apex, laterally compressed, 2–3-angled with convex to flattened faces, apex rounded; base cuneate to oblique, ± flattened; testa semi-glossy, orange-brown; surface coarsely reticulate, ridges prominent, central portion of some cells furnished with short, tubular-spiny, protuberances. FL: Aug–Apr FT: Aug–Sep. Chromosome Number *n* = 11_II_, 2*n* = 22 (AK 283253, *P. B. Cashmore s.n.*, AK 298088, *P. J. de Lange*.

#### Representative specimens

**(15 Sheets seen).** **Moutohora (Whale Island):** P. Hynes s.n., 28 Aug 1970, (AK 185215); Sulphur Bay, Geothermal Area, P. B. Cashmore s.n., 4 Sep 2002, (AK 297561); Boulder Bay, P. B. Cashmore s.n., 4 Sep 2002, (AK 283250); Sulphur Bay, Thermal Area (Active), P. J. de Lange 6469 & P. B. Cashmore, 15 Apr 2005, (AK 289814); Pa Hill/Summit Hill Saddle, P. J. de Lange 6469 & P. B. Cashmore, 15 Apr 2005, (AK 289815); Summit Hill (southern slopes), P. J. de Lange 6472 & P. B. Cashmore, 15 Apr 2005, (AK 289817, Duplicate AD).

#### Distribution

**(Fig. 7).** Endemic, New Zealand, North Island, Bay of Plenty, Moutohora (Whale Island) (sea level to 220 m a.s.l.).

#### Recognition.

*Kunzea salterae* is recognised at species rank because it forms a true-breeding, morphologically stable population, recognised here by a combination of growth habit, branchlet hair and floral characters (see Table 1) as well as minor but consistent DNA sequence differences in the ETS marker region (Table 2; see also de Lange 2007; de Lange et al. 2010). It is further distinguished ecologically by its preference for sand dune and geothermal habitats (Fig. 24A–C), and also by its sympatry/syntopy with *Kunzea robusta*, from which it is isolated morphologically, and from which I saw no field evidence of hybridism (see below). The presence of *Kunzea salterae* on Moutohora, a small (143 ha) volcanic island estimated to be 36 000 years BP (see Ramsay and Hayward 1971) is as remarkable as it is unexpected. Whether the species evolved *in situ*, is a remnant population that persisted there following the extinction of other populations that had colonised the lowered shore line of the Bay of Plenty prior to the sea level rise that occurred at the end of the last glacial maximum, or has recently colonised the island from another as yet unrecognised mainland location remains to be determined.

In past literature *Kunzea salterae* has usually been recorded as *Kunzea ericoides* (e.g., Parris 1971 (as *Leptospermum ericoides*); Ogle 1990; Smale 1994). However, plants found growing within the geothermally active part of the island have also been referred to Kunzea ericoides var. microflora (= *Kunzea tenuicaulis* of this revision) (Wildlands Consultants Limited 2005) probably because of their low stature, apparent habitat preferences, and the widely held but largely mistaken belief (see *Kunzea tenuicaulis*) that any decumbent *Kunzea* found near active fumaroles was that variety.

*Kunzea salterae* has some similarity to *Kunzea tenuicaulis*. In particular the ability to grow in geothermal habitats (Fig. 24C), the characteristically multi-trunked growth habit, broadly spreading canopy, and numerous rather fine, often pendulous branches and branchlets are typical of both species, while the abundance of short, divergent branchlet hairs (Fig. 23A–E) is shared otherwise only with the allied *Kunzea serotina*. *Kunzea salterae*, like *Kunzea tenuicaulis*, has a tendency to produce numerous semi-erect, somewhat trailing or completely decumbent plants in the vicinity of or around active fumaroles (Fig. 24C). From *Kunzea tenuicaulis*, *Kunzea salterae* is distinguished by its longer (up to 18 mm cf. up to 10 mm), linear-lanceolate (Fig. 22A, C–G) rather than oblanceolate to obovate leaves, by its slightly larger (range 2.1–3.8 mm long) and glabrate, rather than small (range 1.8–3.1 mm long) and puberulent, narrowly obconic to funnelform (Fig. 22I–J) rather than cupular to campanulate hypanthium, by its flat, narrowly capitate rather than slightly domed centrally depressed stigma (Fig. 22K), and by the non-testiculate, deeply furrowed thecae (Fig. 22L). Only one mitotic count was obtained from *Kunzea salterae* and this matched *Kunzea tenuicaulis*, *Kunzea serotina* and *Kunzea toelkenii* in having uniformly small chromosome complements. Further observations using different plants are needed to confirm this.

Aside from *Kunzea tenuicaulis*, the narrowly linear-lanceolate foliage of *Kunzea salterae* is similar to that of *Kunzea linearis* and *Kunzea ericoides*. However, the shorter glabrate leaves of *Kunzea salterae* are distinct from the leaves of *Kunzea linearis*, and branchlet hairs of *Kunzea salterae* are short and divergent rather than long, silky and antrorse. Further, the inflorescences of *Kunzea salterae* are corymbiform rather than spiciform, and the individual flowers are distinctly pedicellate, never sessile to subsessile. Both species are also allopatric, the nearest occurrence of *Kunzea linearis* to *Kunzea salterae* being the Tairua Peninsula on the eastern side of the Coromandel Peninsula some 145 km north-west of Moutohora.

*Kunzea salterae* can be distinguished from *Kunzea ericoides* by its copiously hairy branchlets furnished with much longer divergent hairs than are on *Kunzea salterae* branchlets. Further both species are allopatric and have different ITS and ETS sequences (Table 2).

On Moutohora, *Kunzea salterae* is sympatric with *Kunzea robusta*, (e.g., P. J. de Lange 6473 & P. B. Cashmore (AK 289818)), which grows locally on the southern slopes of Summit Hill. The typically multitrunked, pendulous growth habit, consistently narrow linear-lanceolate leaves, and abundance of short, divergent branchlet hairs easily distinguish *Kunzea salterae* from *Kunzea robusta*, which has long, silky, antrorse-appressed, branchlet hairs. *Kunzea robusta* is the less common of the two species on Moutohora, and is absent from sites of geothermal activity there.

*Kunzea salterae* appears to combine the narrow linear leaves typical of *Kunzea linearis*, with the growth habit of *Kunzea tenuicaulis*. Interestingly, artificial F_1_ hybrids (see de Lange et al. 2005) using *Kunzea tenuicaulis* as the pistillate parent, (e.g. P. J. de Lange 5816 (AK 285268)), are a close morphological match for *Kunzea salterae*. Based on current herbarium and field evidence, *Kunzea linearis* is not known from the Bay of Plenty (see above). Nevertheless, the extremely similar morphology exhibited between the aforementioned F_1_ hybrids and *Kunzea salterae* is rather striking. Further research into the possible hybrid origin of *Kunzea salterae*, particularly whether it is derived from past hybridism between *Kunzea linearis* and *Kunzea tenuicaulis*, would be worthwhile.

The rDNA ITS and ETS sequence data (Table 2) showed that *Kunzea salterae* was most similar to *Kunzea tenuicaulis* (de Lange 2007). Otherwise, despite its narrow linear-leaves, it consistently clustered with the other ‘small-leaved’ *Kunzea*, *Kunzea tenuicaulis*, *Kunzea toelkenii* and *Kunzea serotina* (de Lange 2007). ETS sequence data also indicated a relationship with *Kunzea ericoides*, *Kunzea linearis*, *Kunzea tenuicaulis*, *Kunzea toelkenii*, *Kunzea serotina* and Mt Egmont samples of *Kunzea robusta* all of which share a guanine/cytosine mix (de Lange 2007; de Lange et al. 2010). Otherwise *Kunzea salterae* differs from all other *Kunzea* taxa within the *Kunzea ericoides* complex by having a unique cytosine/thiamine mix in its ITS-2 sequence (Table 2; see also de Lange 2007; de Lange et al. 2010).

The identity of *Kunzea* on Tuhua (Mayor Island) was discussed by de Lange (2007) who noted that one collection from the ‘crater rim’ of that island (AK 262432, *G. W. Mason s.n.*), approached *Kunzea salterae* in general branching habit, leaf shape and size, and by the numerous small, corymbiform inflorescences. Although this specimen was in poor condition, de Lange (2007) noted that the branchlet indumentum comprised mainly fine, somewhat wispy, appressed antrorse hairs, and that the anthers lacked the deep furrow and fused ‘pinched’ ridge typical of *Kunzea salterae*. Subsequent field work on that island has shown that the *Kunzea* there forms a uniform population matching the ‘eastern North Island variant’ of *Kunzea robusta* which is common on the adjacent eastern side of the Coromandel Peninsula (see below) (Wilcox et al. 2012).

#### Ecology.

*Kunzea salterae* is a widespread and at times dominant woody shrub or tree of the coastal forest, geothermal field, cobble beach and sand dune vegetation of Moutohora (Fig. 24A–C). As Kunzea ericoides var. ericoides, Smale (1994) described in detail the vegetation associations, population structure and dynamics of *Kunzea salterae*. He concluded (p. 441) that this species ‘may replace itself indefinitely on the unstable dunes on Whale [Moutohora] Island, where the community is still expanding’. Smale’s study was confined to *Kunzea* ‘heaths’ developed over sand dunes, and so he did not appraise the associations formed by *Kunzea salterae* within the geothermally active parts of Moutohora. From observations outside the sand dune habitat, I suggest that *Kunzea salterae*, being a species evidently favouring frequent disturbance, will also have a long standing presence in the geothermally active parts of Moutohora, where it is the dominant vascular plant species. Indeed, in its abundance and growth within the thermal areas on this island, it behaves very much as *Kunzea tenuicaulis* does in similar mainland habitats within the Taupo Volcanic Zone of the North Island (Burns 1997). *Kunzea salterae* is currently often the dominant canopy cover outside the sand dune and geothermal areas of Moutohora (Fig. 24B), but it is expected to decline as the coastal forest regenerates and other larger, coastal forest species attain local dominance.

Smale (1994) observed that the sand dune vegetation dominated by *Kunzea salterae* was species poor, recording 29 vascular plant taxa within what he regarded as ‘older’ stands (i.e. ≥ 27 years of age). The same is the case for the thermal areas, where, aside from dense coverings of the mosses *Isopterygium albescens* (Hook.) A.Jaeger, *Campylopus pyriformis*, *Dicranella dietrichiae* (Müll.Hal.) A.Jaeger, *Philonotis tenuis* (Taylor) Reichardt and Hypnum cupressiforme Hedw. var. cupressiforme (Beever and Brownsey 1990), vascular plants (other than *Kunzea*) are extremely scarce. Smale observed that inland from the dune systems his “Kunzea ericoides var. ericoides” stands changed from the multi-stemmed semi-prostrate growth habit (the *Kunzea salterae* of this treatment) to erect single-stemmed trees. From my observations this transition is not nearly as clear cut as he described, with *Kunzea salterae* growing in most situations across the island with a consistently multi-stemmed habit. However, occasional larger erect trees do occur toward the back of the dune systems at Boulder Bay and these are not *Kunzea salterae* but *Kunzea robusta*, a species that avoids thermal areas, and favours more stable habitats, overlying better developed soils, within the more mature successional coastal forest on the island.

#### Hybridism.

No putative wild hybrids have been observed on Moutohora, and putative hybrids were not evident in the 15 *Kunzea* herbarium specimens examined from that island. The distinctiveness of *Kunzea salterae* was recognised too late in this revision to include it in hybridisation experiments (de Lange et al. 2005).

#### Vernacular name.

No specific name for *Kunzea salterae* has been recorded.

#### Conservation status.

Currently the species, as Kunzea aff. ericoides var. microflora has been appropriately assessed by de Lange et al. (2013b) as ‘At Risk / Naturally Uncommon’ qualified ‘IE’ (Island Endemic) and ‘OL’ (One Location).

### 5. Kunzea toelkenii

Taxon classificationPlantaeMyrtalesMyrtaceae

de Lange sp. nov.

urn:lsid:ipni.org:names:77141730-1

A K. tenuicaulis habitu late expanso (usque ad 6 m lato), brevi (usque ad 4 m alto), valido multicauli, caulibus pertortis torsivis et flexis; surculibus frequentibus perfecte prostratibus ad 4 m e basi trunci expositis; ramis et ramulis superis pendulis, faragine pilorum longorum leniter flexuorum antrorum et brevium divergentium crisporum circinatorum; seriebus rDNA ITS et ETS differt.

#### Holotypus

**Figure 25.**
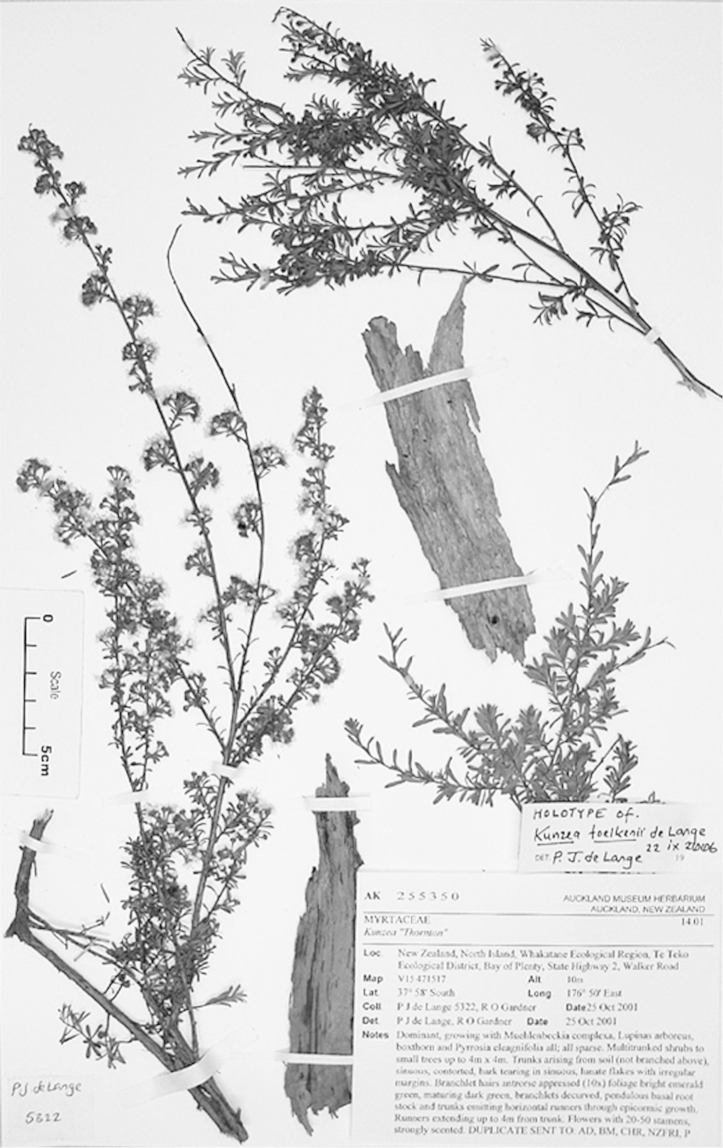
Holotype of *Kunzea toelkenii* (P. J. de Lange 5322 & R. O. Gardner, AK 255350).

**(Fig. 25).** **New Zealand (North Island).** Bay of Plenty, State Highway 2, near Thornton (Wahieroa Dunes), Walker Road, 37°58'27"S, 176°50'11"E, 10 m a.s.l. ‘Dominant, growing with *Muehlenbeckia complexa*, *Lupinus arboreus*, boxthorn and *Pyrrosia eleagnifolia* as sparse associates. Multi-trunked shrubs to small trees up to 4 × 4 m’. P. J. de Lange 5322 & R. O. Gardner, 25 Oct 2001, AK 255350! Isotypes: AD! BM! CHR! NZFRI! P!

#### Etymology.

The specific epithet *toelkenii* honours Australian *Kunzea* expert Hellmut Toelken (1939–).

#### Description

**Figure 26.**
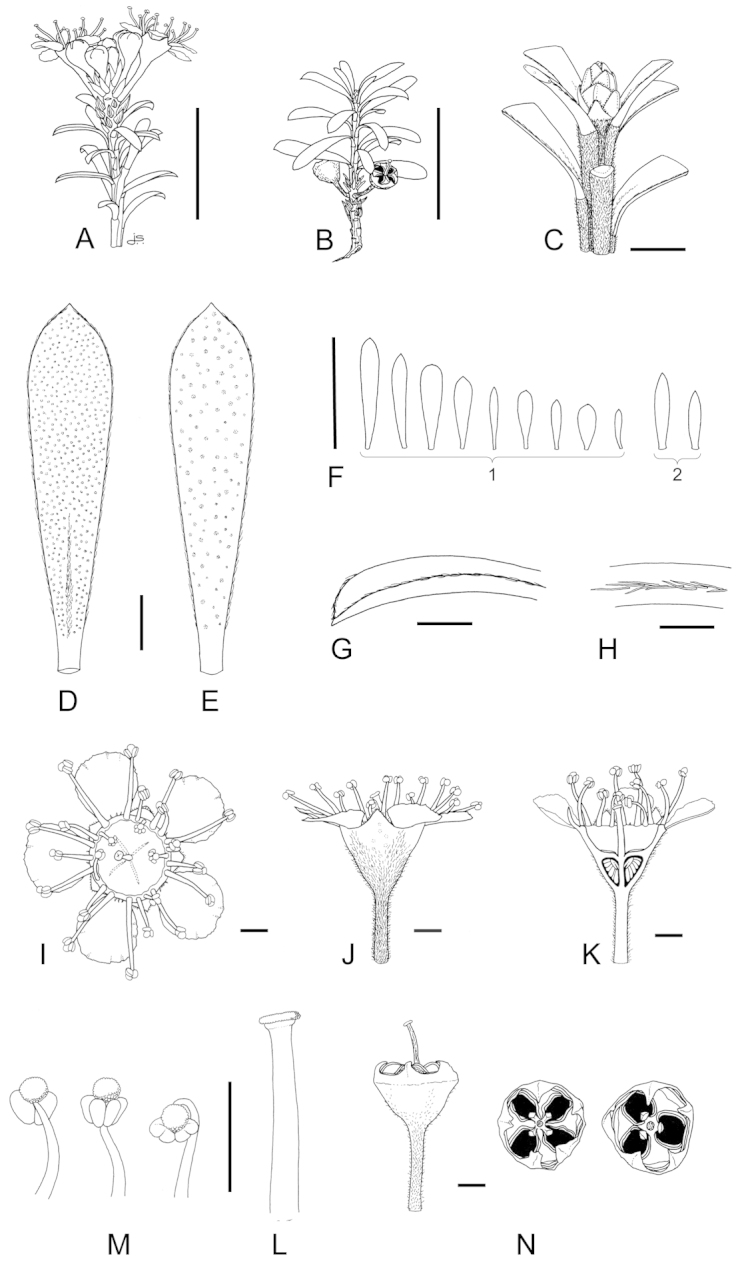
Distinguishing features of *Kunzea toelkenii*. **A** Flowering branchlet (ex cult. AK 284553) **B** Fruiting branchlet (ex cult. AK 284553) **C** Vegetative bud and branchlet indumentum (ex cult. AK 284553) **D** Adaxial leaf surface (ex cult. AK 284553) **E** Abaxial leaf surface (ex cult. AK 284553) **F** Leaf variation: (F1) Walker Road (AK 255350), (F2) Seacombes Canal (AK 287042) **G** Adaxial leaf apex (ex cult. AK 289816) **H** Leaf margin indumentum (ex cult. AK 284553) **I** Flower (top view) (ex cult. AK 284553) **J** Flower and hypanthium (side view) (ex cult. AK 284553) **K** Flower cross section showing anther, style and ovules (ex cult. AK 284553) **L** Style and stigma (ex cult. AK 284553) **M** Stamens (ex cult. AK 284553) **N** Dehisced fruit (ex cult. AK 284553). Scale bars: (**A, B, F**) 10 mm; (**C–E, G, I–N**) 1 mm; (**H**) 0.5 mm.

**Figure 27.**
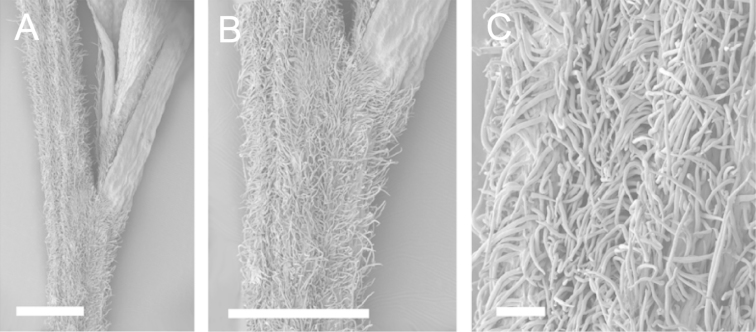
Scanning Electron Micrographs of *Kunzea toelkenii* (all AK 255350). **A–C** Branchlet indumentum. Scale bars: (**A, B**) 1 mm; (**C**) 100 μm.

**Figure 28.**
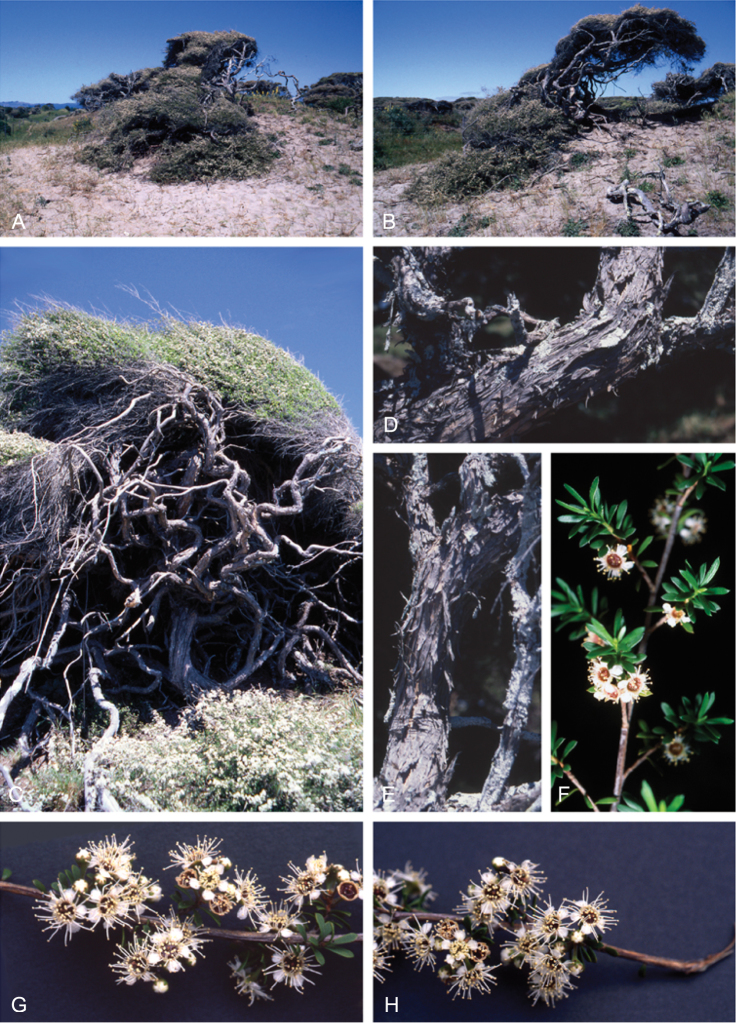
*Kunzea toelkenii* at type locality, Walker Road (photos: *P. J. de Lange*). **A** Growth habit of *Kunzea toelkenii* within sand dunes at type locality, note extensive suckering growth at base of shrub **B** Side view of the same *Kunzea toelkenii* as (**A**) showed tortured growth and root suckers **C** Close up of distinctive branching pattern developed by mature *Kunzea toelkenii* at type locality **D–E** Bark of *Kunzea toelkenii* **F** Late season functionally male flowers of *Kunzea toelkenii* **G–H** Flowering branchlet of *Kunzea toelkenii* (an example with longer than usual stamens).

**(Figs 26, 27, 28).** *Growth habit* shrubs up to 4 × 6 m; ‘juveniles’ usually prostrate and trailing up to 4 m diam., often flowering, taking 2–4 years to develop several, usually central, ascending branches; ‘adults’ forming widely spreading (up to 2 m diam.), characteristically flat-topped shrubs, with pendulous branches and branchlets; branches confined to upper 30–50% of shrub, basal 50–70% usually completely devoid of branches and vegetative growth, sometimes bearing completely pendulous growth; trunk bases usually bearing epicormic, prostrate growth spreading up to 4 m diam. from point of origin; this growth occasionally layering and producing further trunks. *Trunk* (1–)6(–10), ascending to suberect, serpentine, highly contorted, twisted, bent, and spiralled, 0.10–0.25(–0.40) m d.b.h.; mostly arising from the top of a broad rootstock, and also from layered masses of prostrate epicormic growth; in all cases basal portions of trunks covered with numerous semi-detached, sinuous, rather corky, lengths of bark. *Bark* early bark firmly coriaceous, grey or grey-brown, ± elongate, initially with few transverse cracks, soon becoming heavily cracked (into highly irregular pieces with rather sinuous margins (especially on branch flanges and decurrent leaf bases) but remaining firmly attached; old bark similar though more distinctly coriaceous-corky, upper surface often deeply corrugated and cracked but not peeling; detaching inwards readily but usually remaining centrally firmly attached; margins sinuous to lunate, often highly irregular and frayed, rarely shortly tabular; early and old bark flakes firm, scarcely crumbling in hand. *Branches* of trunks numerous, usually confined to the upper 30–50% of trunk; widely spreading, ± serpentine, flexuose, often pendulous and interwoven; branchlets numerous, slender, usually apically pendulous, very leafy, with few to many brachyblasts; those of epicormic growth, straight not flexuose or serpentine, prostrate or pendulous if arising from basal half of trunk, widely spreading; in all cases quadrangular, sericeous, indumentum copious; hairs persistent, of two types: long, appressed often flexuose hairs up to 0.26 mm long, and smaller divergent hairs, with strongly curled and spiralled apices 0.04–0.10(–0.18) mm hyaline to translucent (appearing white when young maturing grey). *Vegetative buds* conspicuous; at resting stage 1.2(–1.8) mm diam.; scales scarious, deciduous, (0.4–)0.9(–1.2) mm long, brown to red-brown, broadly ovate to ovate-deltoid, apex obtuse to rounded; midrib prominent, strongly keeled in upper half, occasionally prolonged to a long cuspidate tip, lateral veins usually absent, oil glands usually absent, upper half of scale margins, keel, and keel apex ciliate. *Leaves* well spaced along branchlets, spreading, patent to recurved; lamina (2.6–)5.7(–8.5) × (0.6–)1.6(–2.5) mm, dark glossy green or bright-green, margins and base usually flushed red; spreading, obovate, clavate, to broadly oblanceolate; weakly to strongly recurved from about 30–50% of total length; apex sharply acute to apiculate, base attenuate; adaxial surface concave very rarely flat, finely glandular punctate; oil glands up to 280, more evident when dry; midrib slightly raised near base, otherwise not evident for rest of length, finely covered in deciduous, sericeous, antrorse-appressed, hairs in lower half otherwise glabrous; abaxial surface convex, glandular punctate, oil glands up to 180, more evident when dry; midrib raised for most of length, glabrous; lamina margin finely to densely sericeous, hairs weakly flexuose, antrorse, subantrorse to spreading, up to 0.5 mm long, hyaline to translucent, appearing white to naked eye, aligned in 1–2 uninterrupted rows meeting just short of leaf apiculus. *Perules* scarious, persistent, (0.5–)1.0(–1.8) mm; basal ones dark brown to red-brown, broadly ovate, ovate, ovate-rostrate, to lanceolate, without oil glands, margins involute, ciliate, midrib strongly keeled with 1–2 usually finely ciliate lateral veins on each side, keel prolonged as a short to long, deciduous, obtuse-tipped, densely ciliate, cuspidate apiculus; remaining perules similar but smaller, chartaceous, (0.3–)0.8(–1.0) mm long. *Inflorescence* a compact, (1–)7(–10)-flowered corymbiform botryum up to 40 mm long, mostly borne on alternate, distinctly spiralled, basally densely leafy, brachyblasts up to 12 mm long; inflorescences at the ultimate branchlet terminus uncommon (except in trailing epicormic growth), if present, often rather elongated (up to 80 mm long) and bearing well developed terminal vegetative growth, often with the uppermost flowers in elongated shoots male. Inflorescence axis densely invested with divergent hairs. *Pherophylls* deciduous (falling very early), initially foliose, soon squamiform, tightly clasping pedicel or spreading, 0.4–1.6 mm long, foliose pherophylls green to bronze-green, shortly lanceolate to obovate, squamiform pherophylls amber-brown to brown, narrowly deltoid to ovate, both types adaxially deeply concave, margins and apex finely ciliate, grading into leaves at inflorescence axis apex. *Pedicels* (1.6–)2.9(–3.8) mm long at anthesis, usually elongating slightly after anthesis, terete, copiously invested with short, divergent to subantrorse, silky hairs. *Flower buds* bluntly clavate to obconic, rarely pyriform, apex flat prior to bud burst with calyx valves not meeting. Fresh flowers when fully expanded (3.6–)6.8(–9.0) mm diam., often functionally male toward end of flowering season. *Hypanthium* (1.7–)2.4(–3.2) × (2.8–)3.6(–4.3) mm, with free portion 0.6–0.9 mm long, green, dark green or red-green; obconic to funneliform, terminating in light-green to pink-green membranous rim bearing five persistent calyx lobes; surface smooth when fresh somewhat wrinkled when dry, with weakly defined ridges leading up to calyx lobes; sparingly dotted with pink or colourless oil glands otherwise with basal half finely and rather densely puberulent with areas leading to calyx lobes distinctly glabrescent; hairs silky, spreading, subantrorse to antrorse-appressed, often with smaller divergent hairs underlying larger appressed ones. Calyx lobes 5, upright (not spreading), submembranous, (0.8–)1.0(–1.2) × (0.7–)1.0(–1.2) mm, persistent, ovate, broadly ovate to ovate-deltoid, of uniform thickness in transverse section, without keel, often uniformly green, otherwise with central portion of lobe darker green or pinkish green, with margins usually pale green to green flushed with pink, surface somewhat glandular punctate, oil glands inconspicuous, ± colourless, otherwise glabrous except for distinctly spreading, ciliate margins. Receptacle usually pink at anthesis, consistently darkening to dark magenta or maroon-black after fertilisation. *Petals* 5(–6), 1.5–1.9(–2.8) × 1.5–1.9(–2.6) mm, white, orbicular to very broadly ovate, apex obtuse to rotund, margins ± entire, often finely folded or crimped 1–5 times, oil glands colourless. *Stamens* 20–36(–50) in 1(–3) weakly defined whorls, arising from receptacular rim, filaments white. Antipetalous stamens (2–)3(–6), antisepalous (1–)3(–8). Outermost antipetalous stamens weakly incurved or outcurved, on filaments 1.2–3.6 mm long, inner stamen if present, 0.8–1.2 mm, incurved or outcurved, a further 1–3 stamens, of similar length are very rarely present at the base of the outermost antipetalous pair. Antisepalous stamens usually shorter than outermost antipetalous stamens, sometimes of comparable length, generally 0.6–3.2 mm, mostly incurved, outcurved or in mixtures of both. Anthers dorsifixed, 0.06–0.09 × 0.05–0.08 mm, testicular-oval to testicular-ellipsoid, latrorse. Pollen white (12.2–)13.6(–17.8) μm. Anther connective gland prominent, pale lemon to pink when fresh, drying yellow to pale orange, spheroidal, finely papillate. *Ovary* absent in males flowers, otherwise 3–4(–5) locular, each with 12–20(–24) ovules in two rows on each placental lobe. Style absent in male flowers, otherwise 1.0–1.4(–1.8) mm long at anthesis, elongating slightly after anthesis, white; stigma capitate, scarcely wider than style, flat, greenish-white, cream or pale pink, surface papillate. *Fruits* rarely persistent, (2.1–)2.6(–3.0) × (2.5–)3.0(–3.7) mm, light brown to grey, obconic, broadly obconic, to cupular, splits concealed by dried, suberect to erect, free portion of hypanthium. *Seeds* 0.50–1.00(–1.02) × 0.52–0.60(–0.68) mm, oblong, oblong-obovate, curved near apex, laterally compressed, 2–3-angled with convex to flattened faces, apex rounded to subacute; base oblique, ± flattened; testa semi-glossy, amber, orange-brown to brown, surface coarsely reticulate. FL: (Sep–)Oct–Nov. FT: Oct–Sep. Chromosome Number *n* = 11_II_, 2*n* = 22 (see de Lange and Murray 2004).

#### Representative specimens

**(16 sheets seen).** **New Zealand (North Island).** State Highway 2, near Seacombes Canal, P. J. de Lange 5324 & R. O. Gardner, 25 Oct 2001, (AK 287042, Duplicate: AD); State Highway 2, Walker Road, P. J. de Lange 5314, 29 Sep 2001, (AK 287049); State Highway 2, Walker Road, P. J. de Lange 5323 & R. O. Gardner, 25 Oct 2001, (AK 287045, Duplicate: CHR); 1.75 km east of Rangitaiki River Mouth, Thornton Wildlife Management Reserve, eastern end of lagoon, P. B. Cashmore s.n., 7 Jun 2007, (AK 299633); 3.3 km East of Rangitaiki River Mouth, near Whakatane Airport Buildings, P. B. Cashmore s.n., 7 Jun 2007, (AK 299634); Whakatane, Piripai Spit, East of Coastlands Subdivision, P. B. Cashmore s.n., 12 Jul 2007, (AK 300903); Ohiwa Harbour, Whangakopikopiko (Tern Islet), P. J. de Lange 7247 & P. B. Cashmore, 5 Dec 2007, (AK 301682, Duplicate: CHR).

#### Distribution

**(Fig. 7).** Endemic, New Zealand, North Island, Bay of Plenty (2–10 m a.s.l.). *Kunzea toelkenii* is known from a small strip of sand dune country (the Wahieroa Dunes) between the eastern bank of the Tarawera River mouth and the west bank of the Rangitaiki River mouth, near Thornton, and from another small population on a barrier sandspit island, Whangakopikopiko (Tern Islet), at the mouth of the Ohiwa Harbour. The current distribution is undoubtedly relict; the habitats occupied are remnants of indigenous woody sand dune vegetation that formerly extended as far west as Papamoa. Although I have been unable to find any supporting herbarium evidence, locals recollect that much of the sand country between Papamoa and Pikowai beach once supported dense ‘kanuka [*Kunzea*] shrublands’ (G. Wrigley pers. comm.). From the descriptions given by these people which include such phrases as ‘tortured growths....pendulous shrubs.......... suckering stems’ it is quite likely that *Kunzea toelkenii* was the species involved, and that it was once a locally important species of the Bay of Plenty sand dune country.

#### Recognition.

*Kunzea toelkenii* is recognised by its uniquely suberect, sprawling growth habit, typically extensive suckering (Fig. 28A–C), by its mixed branchlet hairs (Fig. 27A–C), tendency to produce late season functionally male flowers (Fig. 28F), and also by its restriction to active sand dunes (Fig. 28A–B). Further differences are given in Table 1. The distinctiveness of *Kunzea toelkenii* was probably first recognised in the mid 1980s by the late Mr Derek Gosling of Whakatane who cultivated it, while the unusual ecology of the species was first noted and described in detail by Smale (1994 as Kunzea ericoides var. ericoides).

*Kunzea toelkenii* is distinguished from the other New Zealand members of the *Kunzea ericoides* complex by its unique growth habit, in particular the spiralled, often tortured multi-trunked (Fig. 28C) growth habit, and ability of the trunk base to produce numerous, completely prostrate, widely spreading epicormic branches. This growth habit has previously been interpreted as habitat induced (Smale 1994). However, cultivation trials initiated by Mr Gosling showed that the distinctive multi-trunked shrub habit has a genetic basis. In cultivation, seedlings are completely prostrate but within 2–4 years of germination most develop one or more suberect trunks. Interestingly, Smale (1994) described the same development (what he termed ‘semi-prostrate candelabra’ habit) in specimens as ‘early as 6 years’ of age. The ability of *Kunzea toelkenii* to sucker from the trunk base is similar to the growth habit of five of the seven endemic Australian members of the *Kunzea ericoides* complex (de Lange 2007). However, while these Australian species all possess a distinctly bulbous, fire-resistant lignotuber (with at least one of these species, *Kunzea leptospermoides* F.Muell. ex Miq. also having a rhizomatous habit), such structures are absent in *Kunzea toelkenii*.

The branchlet indumentum of *Kunzea toelkenii* is also distinctive, comprising mixtures of sparse, long antrorse-appressed, somewhat flexuose hairs, and more numerous, somewhat shorter, divergent, often curled and spiralled hairs (Fig. 27C). No other New Zealand *Kunzea* species has such distinctive curled and spiralled divergent hairs, although mixtures of appressed and divergent hair types is a feature common to the hybrid *Kunzea robusta* × *Kunzea tenuicaulis*. Although a hybrid origin for *Kunzea toelkenii* seems likely, and experimental examples of *Kunzea robusta* × *Kunzea tenuicaulis* (AK 286145) are very similar to it, experimental hybrids of this cross lacked the curled and spiralled branchlet hairs and distinctive growth habit of *Kunzea toelkenii*.

Ecologically, *Kunzea toelkenii* is further distinguished as the only member of the *Kunzea ericoides* complex truly endemic to sand dune systems (cf. *Kunzea amathicola* described later). Within its sand dune habitat *Kunzea toelkenii* is known to occur sympatrically only with *Kunzea robusta* and even then scarcely so (e.g., Coastlands, Whakatane (P. B. Cashmore s.n., (AK 300902)) and Whangakopikopiko (Tern Islet), Ohiwa Harbour (P. J. de Lange 7248 & P. B. Cashmore, (AK 301683)). However, this pattern more probably reflects past land clearance patterns because elsewhere in the Bay of Plenty *Kunzea robusta* is widespread, with a range that extends to sand dunes, e.g., Waihi Beach, Matakana Island.

A final peculiarity is the tendency of *Kunzea toelkenii*, uniquely amongst the New Zealand species, to produce functionally male flowers (Fig. 28F). In most cases these flowers had almost vestigial, non-functional stigmas, though occasionally even these are absent. Such flowers have been described for New Zealand examples of *Leptospermum scoparium* (Primack and Lloyd 1980) and are now known from at least five other Australian species of that genus (Andersen 1990; O’Brien 1994) but as far as I am aware they have not been reported previously for *Kunzea*. As with the *Leptospermum* examples studied (see in particular Andersen 1990), functionally male flowers appear toward the end of the flowering season. In *Kunzea toelkenii* they appear to be consistently produced in wild and cultivated plants, though in varying degrees and not necessarily on every plant.

*Kunzea toelkenii* has the same ITS and ETS sequences (Table 2) as *Kunzea serotina* (de Lange 2007; de Lange et al. 2010). No variable sites are present in the ITS sequence, while the ETS sequence of both species, together with *Kunzea ericoides*, *Kunzea robusta* (Mt Egmont samples only) *Kunzea salterae* and *Kunzea tenuicaulis*, share a guanine/cytosine mix at ETS alignment position 232 (de Lange 2007). Otherwise the ETS asequence of *Kunzea toelkenii* shares an adenine nucleotide with *Kunzea salterae*, *Kunzea serotina*, and *Kunzea tenuicaulis* (de Lange 2007).

#### Ecology.

The ecology of *Kunzea toelkenii* was described in detail by Smale (1994 as Kunzea ericoides var. ericoides). The extant consolidated and semi-consolidated foredune and dune swale habitats of *Kunzea toelkenii* near Thornton are estimated to be less than 700 years old (Pullar and Selby 1971), while Whangakopikopiko (Tern Islet), a barrier-spit island at the Ohiwa Harbour mouth, is probably even younger. *Kunzea toelkenii* is the dominant woody species within these habitats and associated species are scarce. Smale (1994) recorded 17 vascular flora associates from the Thornton site, the majority of which were exotic naturalised species, and only five were woody trees or shrubs. Of the naturalised species at Thornton, Smale (1994) identified boxthorn (*Lycium ferocissimum* Miers) as potentially invasive and a possible threat to *Kunzea toelkenii*. He also observed that the *Kunzea toelkenii* population appeared to form a single distinct cohort with a mean age of 45 years, though specimens up to 70 years of age were occasionally encountered, and that the stands sampled appeared to have arisen through invasion of open sand dune vegetation, possibly after fire had removed the previous vegetation cover. Smale (1994) was of the opinion that the *Kunzea* stands (here *Kunzea toelkenii*) would replace themselves indefinitely, partly because Thornton is so isolated from other seed sources of potential successors. A similar, though less dense population to that seen at Thornton is present at Whangakopikopiko (Tern Islet), and there the impression is of a very recent establishment. If so, this suggests that at some stage *Kunzea toelkenii* was present on the adjoining sand spits of Ohope and Ohiwa, all now extensively developed and housed, and from where only scattered individuals of *Kunzea robusta* are now known.

#### Hybridism.

Within its habitat *Kunzea toelkenii* very rarely associates with other *Kunzea* species and thus far wild hybrids have not been found. Nevertheless experimental hybrids were readily produced using *Kunzea toelkenii* as pistillate or staminate parent (de Lange et al. 2005, as Kunzea aff. ericoides (d)).

#### Vernacular name.

No specific vernacular appears to be in use for *Kunzea toelkenii*.

#### Conservation status.

As Kunzea aff. ericoides (a) (AK 255350; Thornton) *Kunzea toelkenii* is appropriately assessed by the New Zealand Threatened Vascular Plant Panel (de Lange et al. 2013b) as ‘Acutely Threatened/Nationally Vulnerable’, qualified ‘Range Restricted (RR)’.

### 6. Kunzea linearis

Taxon classificationPlantaeMyrtalesMyrtaceae

(Kirk) de Lange et Toelken comb. et stat. nov.

urn:lsid:ipni.org:names:77141738-1

Leptospermum ericoides var. linearis Kirk in *For. Fl.* (1889), 125, Plate LXIX (t.69), f.2
Leptospermum lineatum (Kirk) Cockayne in *Rep. Dune Area N.Z.*, (1911), 38.
Kunzea ericoides var. linearis (Kirk) W.Harris in *N.Z.J.Bot.* 25, (1987), 134.

#### Holotype

**Figure 29.**
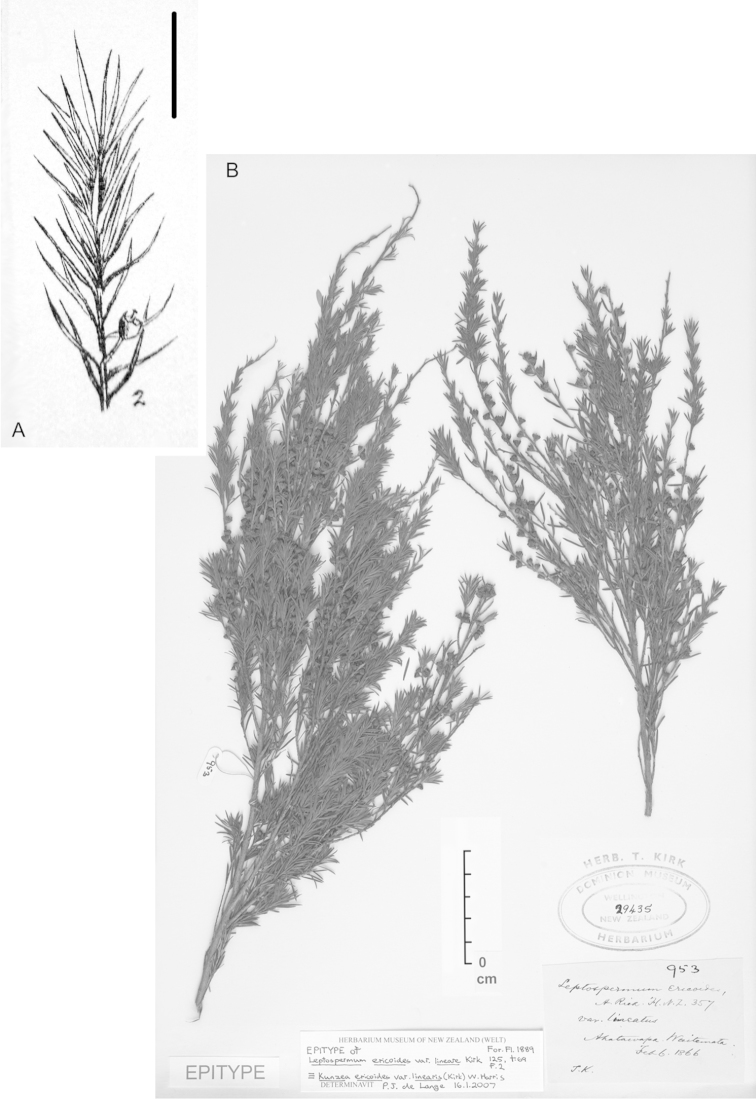
Holotype and epitype of Leptospermum ericoides var. lineare Kirk. **A** Holotype of Leptospermum ericoides var. lineare Kirk, illustration t.69 (f.2) in Kirk (1889) **B** Epitype of Leptospermum ericoides var. lineare Kirk (WELT SP029435). Scale bar: (**A**) 10 mm.

**(Fig. 29A).** T. Kirk, The Forest Flora of New Zealand (1889), Plate LXIX (t.69), f.2.

#### Epitype

**(here designated)** **(Fig. 29B).** Ahatawapa, Waitemata T. K[irk] 953, Feb 6 1866, WELT SP29435! Labelled by Kirk as Leptospermum ericoides A. Rich. Fl. N. Z. 357 var. lineatus [*sic*].

#### Notes.

Kirk (1889; p. 125) published Leptospermum ericoides var. linearis with a brief description which is given here in full: ‘var. β, *linearis*. Young shoots, leaves, and calyces silky; branchlets densely crowded; leaves linear and pungent, ^1^/_40_ in. wide, margins slightly recurved; calyx with more acute teeth; petals very small, crumpled. Calyx-teeth erect in fruit. This is probably a distinct species.’ This description was accompanied by a small, somewhat stylised illustration (‘f.2’) of a fruiting sprig (Fig. 29A). No locations were given or other specimens cited. Therefore, as the sole element accompanying the protologue I regard this illustration as the holotype (see Article 9.1, Note 1, especially the statement: ‘if the author used only one element, it must be accepted as the holotype’). This is because, despite the wealth of collections in the Kirk herbarium at WELT and additional gatherings at K, all labelled in Kirk’s hand with his new name (though often spelled var. lineatus, and/or or var. lineatum (see also Kirk 1899)), Kirk (1899) did not cite any of these in his protologue, leaving the illustration, which accompanies the description and its direct citation by the naming author (e.g., ‘f.2’), as the only possible choice. However, as the holotype is stylised it is inadequate to allow a precise application of the name Leptospermum ericoides var. linearis, therefore in accordance with Article 9.8 of the International Code of Nomenclature (McNeill et al. 2012) I designate WELT SP029435 as epitype (Fig. 29B). This sheet comprises two fruiting specimens which clearly show the densely crowded branchlets, linear leaves, and fruits bearing persistent, erect calyx lobes. These are some of the distinguishing characters mentioned in Kirk’s protologue for his new variety (Kirk 1889; p. 125). Further, the specimens were clearly collected by and labelled in Kirk’s hand as ‘Leptospermum ericoides var. lineatus’.

#### Etymology.

The specific epithet *linearis* refers to the linear leaves of this species, a condition much remarked upon by Thomas Kirk on his herbarium specimens, and to a lesser extent in his descriptions (Kirk 1889, 1899).

#### Description

**Figure 30.**
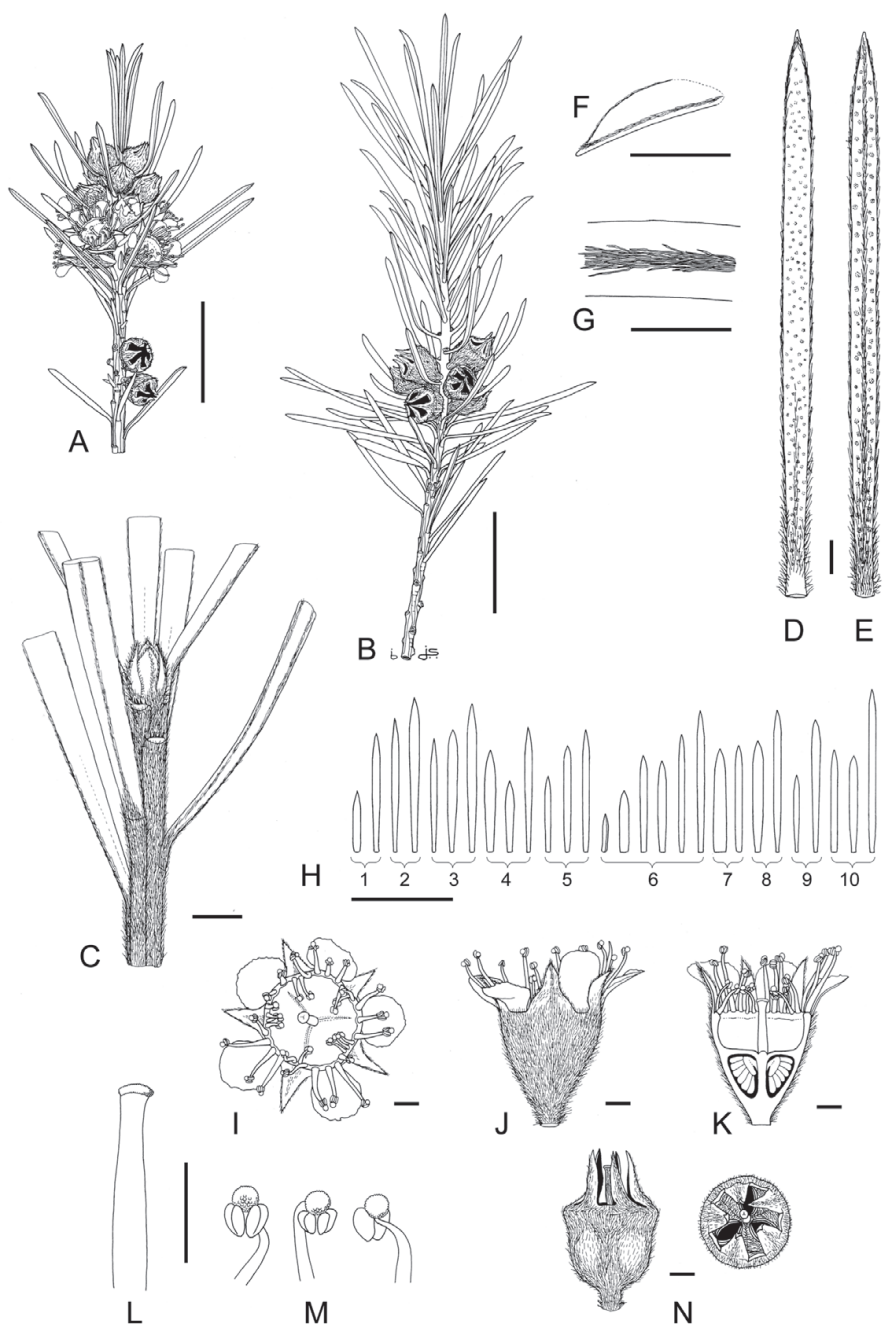
Distinguishing features of *Kunzea linearis*. **A** Flowering branchlet (ex cult. AK 287881) **B** Fruiting branchlet (ex cult. AK 287881) **C** Vegetative bud and branchlet indumentum (ex cult. AK 287881) **D** Adaxial leaf surface (ex cult. AK 287881) **E** Abaxial leaf surface (ex cult. AK 287881) **F** Adaxial leaf apex (ex cult. AK 287881) **G** Leaf margin indumentum (ex cult. AK 287881) **H** Leaf variation: (**H1**) Surville Cliffs (Glabrescent form, AK 287872), (**H2**) Surville Cliffs (Hairy Form) (AK 287955), (**H3**) North Island, Te Paki, Taumatatotara Flat (AK 287953), (**H4**) North Island, Houhoura Harbour, Perpendicular Point (AK 211064), (**H5**) North Island, Karikari Peninsula, Lake Waiporohita (AK 287886), (**H6**) Waipapa Stream (AK 288775), (**H7**) North Island, Raetea Forest (AK 206328), (**H8**) North Island, Waipu Cove Road (AK 287889), (**H9**) North Island, Northcote, Ahatawapa (AK 288766), (**H10**) North Island, Hauraki Plains, Waikumete Stream (AK 286054) **I** Flower (top view) (ex cult. AK 287881) **J** Flower and hypanthium (side view) (ex cult. AK 287881) **K** Flower cross section showing anther, style and ovules (ex cult. AK 287881) **L** Style and stigma (ex cult. AK 287881) **M** Stamens (ex cult. AK 287881) **N** Dehisced fruit (ex cult. AK 287881). Scale bars: (**A, B, H**) 10 mm; (**C–F, I–N**) 1 mm; (**G**) 0.5 mm.

**Figure 31.**
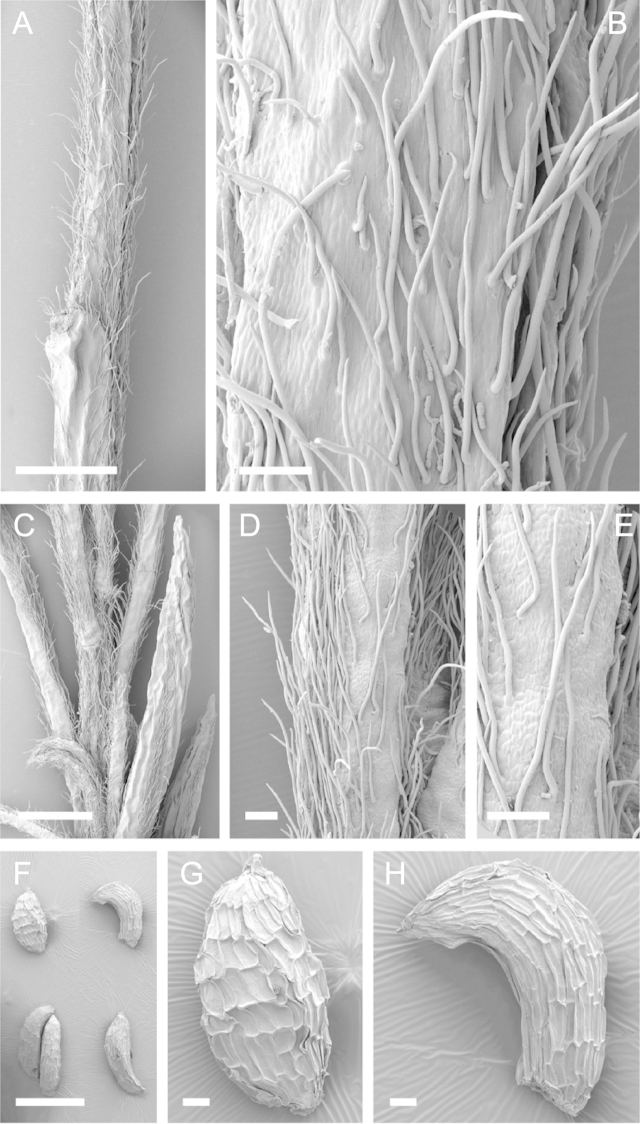
Scanning Electron Micrographs of *Kunzea linearis*. (**A–E** all AK 287954) Branchlet indumentum **F–H** Seeds (AK 206336). Scale bars: (**A, C, F**) 1 mm; (**B, D, E, G, H**) 100 μm.

**Figure 32.**
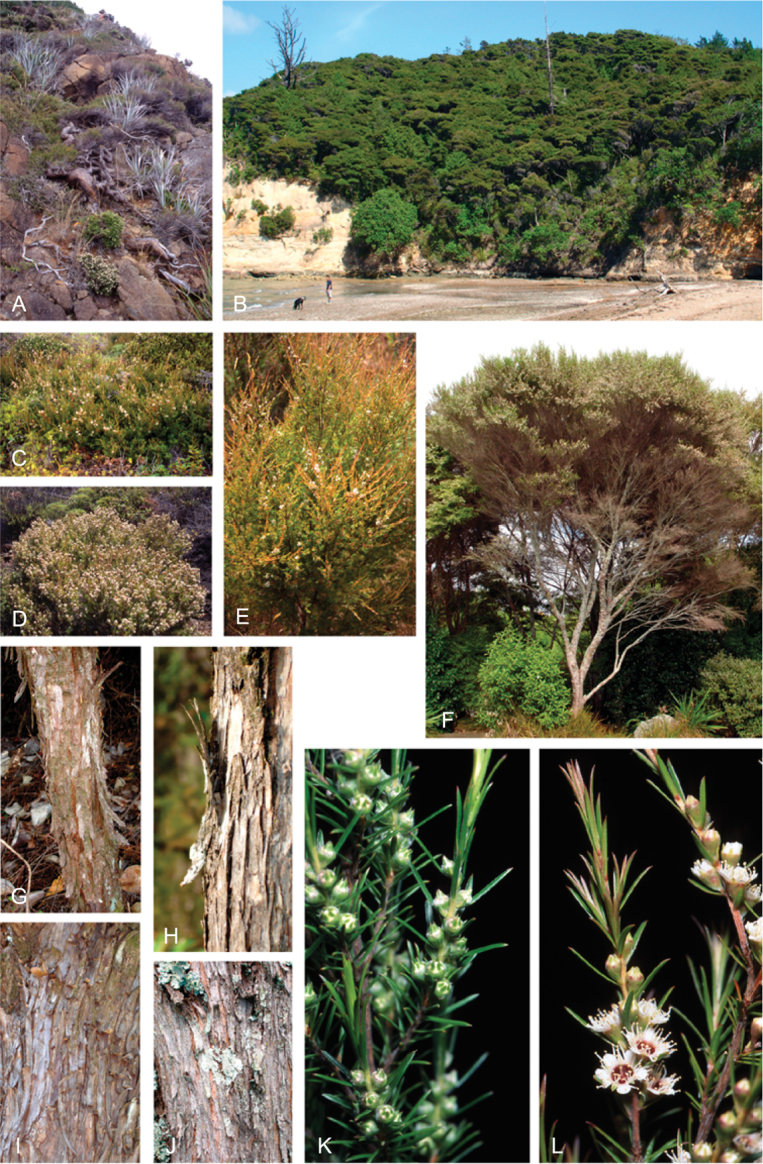
*Kunzea linearis*. **A** *Kunzea linearis* sprawling form developed on windswept ultramafic rocks, North Island, North Cape Scientific Reserve, Surville Cliffs, (photo: *P. J. de Lange*) **B** Coastal shrubland developed on steep turbidite cliffs, North Island, Auckland, Waitemata Harbour, Kendal’s Bay (photo: *P. J. de Lange*) **C–D** Decumbent shrub form developed on ultramafic soils North Island, North Cape Scientific Reserve, Surville Cliffs, (photo: *P. J. de Lange*) **E** Adult plant exhibiting the erect growth habit usually seen throughout range, North Island, Te Aupouri Peninsula, Te Kao, (photo: *P. J. de Lange*) **F** Adult tree showing ascending, plumose branching pattern; North Island, Auckland City, Western Springs (photo: *P. J. de Lange*) **G–J** Bark showing the characteristic tessellated pattern and lunate flakes typical of this species, North Island, Auckland, Waitemata Harbour, Kendal’s Bay (photo: *P. J. de Lange*) **K** Spiciform botrya of *Kunzea linearis* showing buds with the distinctive erect calyx lobes, North Island, Karikari Peninsula, Lake Ohia (photo: *J. E. Braggins*) **L** Flowering spiciform botrya of *Kunzea linearis*, note position of petals and presence of active vegetative growth at inflorescence apex, North Island, Karikari Peninsula, Lake Ohia (photo: *J. E. Braggins*).

**(Figs 30, 31, 32).** *Growth habit* erect shrubs or small trees up to 12 m forming dark green to silvery-grey, erect but more or less rounded, plumose, densely branched canopies up to 2 m diam., sometimes (usually on ultramafic rocks) decumbent and/or trailing. *Trunk* 1(–4 or more), mostly erect but in trailing specimens distinctly serpentine, 0.10–0.46(-0.60) m d.b.h.; basal portion of trunks initially covered with rather thick, firm, stringy, brown to brownish grey coriaceous bark. *Bark* early bark firmly coriaceous, dark brown to brown, ± elongate, usually bearing a few transverse cracks (especially on branch flanges and decurrent leaf bases) otherwise remaining firmly attached, margins elongate sinuous, ± entire with scarcely any flaking; old bark similar though more distinctly corky-coriaceous, coarsely tessellated and remaining firmly attached, if detaching then usually doing so along transverse cracks, and peeling inwards to leave distinct layers of chartaceous, lunate, flakes that are centrally attached; flakes usually with highly irregular, frayed and shattered apices, otherwise margins ± entire; upper surface of bark flakes tessellated; upper trunk bark crumbling readily in hand, shattering if pulled hard into numerous, small, tabular flakes. *Branches* numerous, usually present from close to or at trunk base, but becoming progressively confined with age to the upper half of trunk; ascending to upright, very rarely spreading (usually in decumbent plants), usually distinctly plumose and often bearing old fruits; branchlets numerous, plumose, rather slender, ± quadrangular to subterete, leaves crowded along stems; branchlets sericeous, indumentum copious, hairs antrorse-appressed, weakly flexuose, up to 0.68 mm long, hyaline to translucent (appearing silvery when young, maturing silver-grey). *Vegetative buds* inconspicuous, usually obscured from view by surrounding leaves; at resting stage 0.2–0.8 mm diam. narrowly ovoid; scales deciduous; (0.2–)1.2 mm long, stramineous to pale brown, broadly ovate-lanceolate grading through lanceolate to narrowly lanceolate; midrib strongly keeled, prolonged to apiculate tip, often with one prominent row of 2–6 oil glands on either side of midrib; scales initially completely obscured by long silky silvery-white hairs, becoming glabrate, with hairs progressively confined to scale margins, midrib, and keel prolongation. *Leaves* not heterophyllous, sessile, usually hairy, very rarely glabrous, densely crowded along branchlets, particularly toward apices, initially obliquely ascending, subappressed to suberect, basally often spreading to weakly recurved in distal one-third; lamina (9.3–)12.7(–19.5) × (0.3–)0.7(–1.2) mm, initially silvery-grey (due to dense hair covering), maturing dark green to glaucous green above (as hairs are shed) with a dull not glossy surface, paler beneath; lamina linear, distal one-third sometimes weakly recurved, apex sharply acute, cuspidate, base attenuate (with adaxial surface often glabrous, abaxial densely hairy); adaxial lamina surface flat to weakly concave, glandular punctate, with oil glands evident when fresh or dry (though more conspicuous when dry), up to c.300, midrib very slightly raised near base, otherwise only evident for c. one-third of length as a conspicuous line of silvery-grey antrorse-appressed, silky hairs up to 0.8 mm long; abaxial surface flat to weakly convex, glandular punctate, oil glands up to 300; midrib raised for entire length, densely sericeous to just short of leaf apex, hairs as for adaxial midrib and lamina margins; lamina margins copiously covered in silvery-grey hairs, these forming a thick band and fusing with the abaxial midrib hairs just short of lamina apex, and along decurrent leaf bases. *Perules* deciduous, (0.3–)1.8(–2.3) mm long, straminaceous to pale brown, narrowly ovate, ovate-lanceolate grading through to narrowly lanceolate; midrib strongly keeled, cuspidate, with an obscure row of 2–8 oil glands on either side of midrib; lamina initially obscured by long silky silvery-white hairs, becoming glabrate, with hairs progressively confined to scale margins, midrib, and keel prolongation. *Inflorescence* mostly compact, spiciform (3–)8(–12)-flowered botrya 20–80 mm long; usually on brachyblasts with the terminal shoot either bearing a slightly longer (up to 180 mm) compact 6–15-flowered, spiciform botryum, or a greatly elongated, spiciform, 10–40-flowered botryum up to 180 mm long. Flowers of smaller botrya crowded, those of elongated botrya regularly spaced up to 20 mm apart; terminal portion of both short and elongated spiciform botrya inflorescence types often bearing undeveloped flowers and active vegetative growth. Inflorescence axis densely invested in antrorse-appressed, weakly flexuose, silky hairs. *Pherophylls* persistent, leaf-like, 1–2 per flower, closely clasping hypanthium base, usually hairy, very rarely glabrous; lamina (6.0–)9.8(–12.8) × (0.9–)1.8(–2.2) mm, dark silvery-green, silvery-grey or glaucous (depending one extent of hair covering), linear to linear-falcate; linear-falcate pherophylls with basal portion sharply bent almost at right angles to inflorescence axis, otherwise obliquely ascending to suberect, or spreading; apex acute, base attenuate; adaxial surface usually deeply concave to weakly so, glandular punctate, oil glands up to c.100 (usually fewer); midrib slightly raised near base, otherwise indistinct, bearing antrorse-appressed, silky, hairs for whole length or glabrous; abaxial surface deeply convex, glandular punctate, oil glands up to 100 (usually fewer); midrib scarcely evident especially if glabrous, otherwise mostly evident as a dense line of antrorse-appressed, silky hairs continuing to the apex, lamina margin usually densely covered by antrorse-appressed, sericeous hairs, sometimes glabrous. *Pedicels* sessile to subsessile, up to 1.2 mm long at anthesis, scarcely elongating after anthesis, terete, copiously invested with silky, antrorse-appressed, weakly flexuose, hairs. *Flower buds* ovoid, double conic to pyriform, apex sharply erect; calyx lobes pinched at apex inwards, and touching prior to bud burst. Fresh flowers when fully expanded (1.9–)3.9(–5.7) mm diam. *Hypanthium* (2.0–)2.8(–4.0) × (2.5–)3.4(–4.1) mm, with free portion 0.6–0.9 mm long, silvery-white to silvery-grey due to copious covering of hairs or dark red-green if glabrous; barrel-shaped, cupular or narrowly campanulate, terminating in scarcely defined chartaceous rim bearing 5 persistent sharply erect calyx lobes; hypanthium surface smooth, usually completely covered in a dense covering of long, silky, antrorse-appressed silvery hairs; ribs scarcely evident. Calyx lobes 5, erect, subcoriaceous, (1.0–)1.3(–1.6) × (0.2–)0.4(–0.6) mm, persistent, narrowly deltoid to deltoid with acute tips, red-green, weakly keeled or not, lobes densely covered in long, silky, silvery, antrorse-appressed, hairs or glabrous; margins green flushed pink or red, oil glands evident only in glabrous forms, rather inconspicuous, ± colourless. Receptacle green or pink at anthesis, usually darkening to crimson after fertilisation. *Petals* 5(–6), (0.9–)1.4(–2.0) × (0.7–)1.4(–1.9) mm, cream, pale pink or cream basally flushed pink, narrowly ovate to suborbicular, suberect, upper one-third sometimes weakly recurved, apex rounded, margins ± finely and irregularly crumpled, sometimes denticulate, oil glands colourless. *Stamens* 32–46(–60) in 1–2 weakly defined whorls, arising from receptacular rim, filaments cream. Antipetalous stamens (2–)3(–6) sometimes petaloid, antisepalous (3–)4(–7). Outermost antipetalous stamens initially erect with the upper portion often incurved, more rarely outcurved, on filaments 1.2–1.8 mm long, inner stamen if present, 0.9–1.6 mm, erect or incurved, often a further 1–3 stamens, of similar length to inner stamens may be present at the base of the outermost antipetalous pair. Antisepalous stamens shorter than outermost antipetalous stamens, 0.8–1.0 mm, erect or weakly to strongly incurved, rarely outcurved, usually in mixtures of both. Anthers dorsifixed, 0.04–0.06 × 0.02–0.04 mm, testiculate, latrorse. Pollen white (13.2–)16.2(–21.0) μm. Anther connective gland prominent, pale pink or golden-yellow when fresh, drying yellow to pale orange, spheroidal, finely to coarsely papillate. *Ovary* (3–)4(–5) locular, each with 18–26(–30) ovules in two rows on each placental lobe. Style 0.8–2.0 mm long at anthesis, elongating after anthesis, cream or pale pink; stigma narrowly capitate, as wide as, or slightly wider than style, ± flat, greenish-white or pink, flushing red after anthesis, surface finely granular-papillate. *Fruits* long persistent, (1.6–)2.3(–2.9) × (2.3–)3.0(–4.1) mm, initially silvery-white or silvery-grey due to dense hair covering, maturing grey-brown to grey-black depending on degree of hair loss, sometimes completely glabrous in which case dark brown; in all types fading with age to pale grey in exposed situations or grey-black in shade, barrel-shaped to narrowly obconic, rarely campanulate to cupular, calyx valves prominently erect, splits concealed by dried, erect, free portion of hypanthium. *Seeds* 0.50–1.00(–1.10) × 0.48–0.63(–0.70) mm, obovoid, oblong, oblong-ellipsoid, or cylindrical and ± curved; usually curved near apex, laterally compressed, 2–3-angled with convex to flattened faces, apex rounded to subacute; base oblique, ± flattened; testa semi-glossy, orange-brown to dark brown, surface coarsely reticulate. FL: (Jul–)Nov–Jan(–May). FT: Jun–May. Chromosome Number 2*n* = 22 (see de Lange and Murray 2004).

#### Representative specimens

**(148 sheets seen):** **New Zealand (North Island).** Te Paki, North Cape Scientific Reserve, Surville Cliffs, P. J. de Lange 1250 & G. M. Crowcroft, 30 Jan 1992, (AK 207192, Duplicates: AD, CHR); Te Paki, Tom Bowlings Bay, H. Carse s.n., Dec 1926, (CHR 296369); Te Paki, Spirits Bay/Kerr Point Road junction, R. Cooper s.n., 30 Oct 1969, (AK 121371, Duplicate: CHR); Te Aupouri, Te Kao (near school/Te Ahu road junction), P. J. de Lange 4164, 18 Jan 2000, (AK 287887; Duplicate: AD, NSW); Te Aupouri, Mt Camel, near Perpendicular Point, P. J. de Lange 1865, 15 Nov 1992, (AK 211064, Duplicates: AD, CHR); Rangaunu Harbour, Kaimaumau, R. Cooper s.n., 7 Nov 1966, (AK 117773); Waipapakauri, H. Carse s.n., 7 Jan 1902, (WELT SP077488); Kaitaia, H. B. Matthews s.n. & H. Carse, Dec 1918, (CHR 296350); Ahipara Gumfields, Waitaha Stream, P. J. de Lange 4146, 17 Jan 2000, (AK 287957); Mangonui, Rangiawhia School, R. Cooper s.n., 25 Aug 1965, (AK 123157); Mangamuka, Raetea Forest, L. J. Forester s.n., 5 Mar 1992, AK 206336; Whangaroa Harbour, Wainui Road, Waitapu Bay, P. J. de Lange 5987 & P. B. Heenan, 1 Apr 2004, (AK 286197, Duplicate: AD); Russell, Bay of Islands, D. Petrie s.n., May 1897, (WELT SP029463); Between Waimate and the Bay of Islands, W. Colenso 182, 30 Jul 1844, (WELT SP022866, Duplicate: K); Kai Iwi Lake, R. Cooper s.n., 13 Nov 1968, (AK 120232); Whangarei, near Marsden Point, R. O. Gardner 10178, 24 May 2000, (AK 251630); Pouto Peninsula, Sail Point, above Clarkes Bay, P. J. de Lange 6288 & R. O. Gardner, 10 Aug 1995, (AK 288776); Mangawhai, Molesworth Drive, P. J. de Lange 5537 & G. M. Crowcroft, 4 Oct 2002, (AK 283238); Te Arai Point Road, Te Arai, P. J. de Lange 5534 & G. M. Crowcroft, 3 Oct 2002, (AK 283237, Duplicate: CHR); Takatu Peninsula, Million Bay, Campbells Beach, P. J. de Lange 6330, 12 Jan 2005, (AK 289208); Northcote, Waitemata Harbour, North Block, ‘Aha Tawa Pa’ (Tennyson Road), P. J. de Lange 6284, 15 Nov 2004, (AK 288766, Duplicates: AD, CHR, K, MEL, NSW, NZFRI, WAIK, WELT); Birkdale, H. B. Matthews s.n., 1919, (AK 102429); Auckland, near Cox’s Creek, T. Kirk s.n., n.d., (K); Maramarua – Matamata Road (State Highway 27), 800 m north of Waikumete Stream, P. J. de Lange 4625, 7 Nov 2000, (AK 286054, Duplicates: AD, CHR, WAIK); Hapuakohe Range, Wai Iti Road, above Ohinekaua Stream, P. J. de Lange 4707, 16 Nov 2000, (AK 288490, Duplicates: AD, CHR); North Wairarapa, 1 mile west of Kupukore, A. P. Druce s.n., May 1965, (CHR 132842). **Poor Knights Islands:** Aorangi, western ridge of Tatua Peak, P. J. de Lange 6875, 14 Jan 2007, (AK 298368, Duplicate: CHR).

#### Distribution

**Figure 33.**
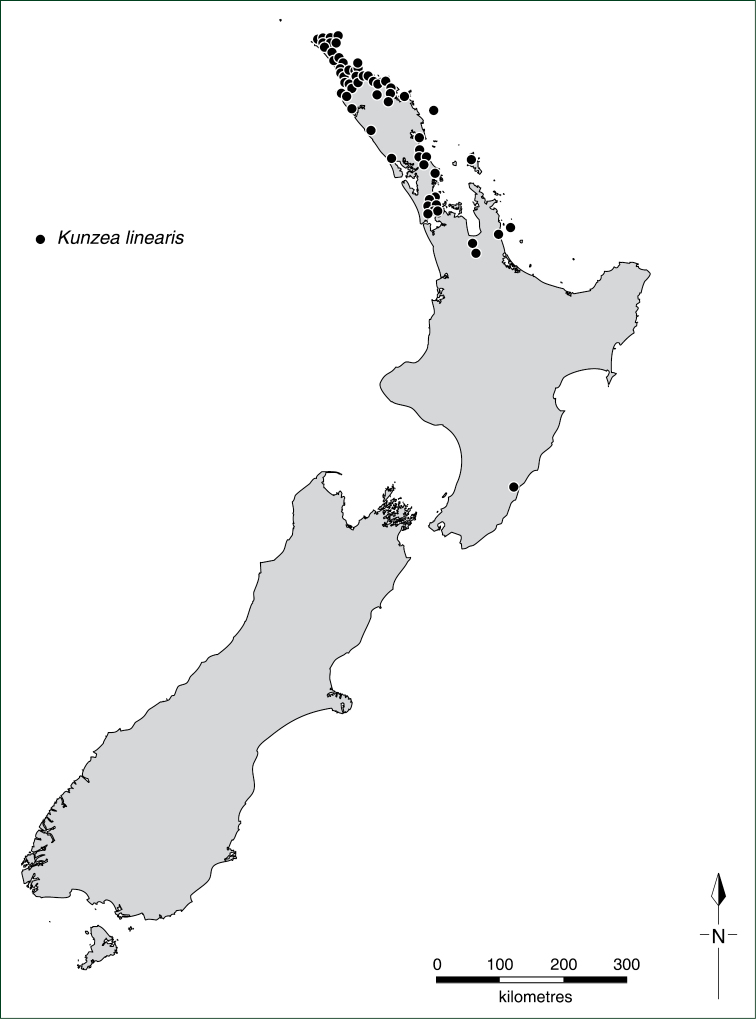
Distribution of *Kunzea linearis*.

**(Fig. 33).** Endemic. New Zealand, North Island (sea level – 310 m a.s.l.). Recorded from Te Paki south to the Ahipara Gumlands and the Karikari Peninsula. South of there it is sporadic and mainly coastal to the Waitemata Harbour. Also present on the western side of Aotea (Great Barrier Island), the eastern side of the Coromandel Peninsula (near Tairua), on the western margin of the Hauraki Plains just north of Kaihere, and within the foothills of the Hapuakohe Range. South of there *Kunzea linearis* is known only from a single, highly disjunct collection made by A. P. Druce (CHR 132842) from near Mt Kupukore, in the northern Wairarapa. Although I have seen no other specimens from the southern half of the North Island, I accept this record, because the collector A.P. [Tony] Druce, was a well known, cautious botanical explorer not prone to making labelling errors, and with a critical eye for the unusual (Atkinson 1999). Also, at the time of that specimen’s collection in May 1965, Druce was unfamiliar with *Kunzea linearis* (he had labelled his specimen ‘*Leptospermum ericoides*’). In fact it was not until May 1987, 22 years later that he made his next herbarium collection of *Kunzea linearis* from Ahipara (CHR 469707), and that gathering Druce labelled as an ‘unnamed’ species (*Kunzea* “Ahipara” (Druce 1993)), apparently not realising that it already had a formal name within the genus. Although subsequent searches of Mt Kupukore made at my request in 2007 by Mr Pat Enright (*in litt.*) failed to find *Kunzea linearis* there, hybrids between it and *Kunzea robusta* were present, suggesting its past, or continuing presence in the area.

#### Recognition.

*Kunzea linearis* is the most distinctive of the New Zealand *Kunzea* species (see Table 1). Its discovery by Thomas Kirk at Ahatawapa and Cox’s Creek, Auckland was remarked upon by Hooker (1867; p. 728) who noted it’s distinctiveness in his treatment of *Leptospermum ericoides* but elected not to name it because ‘the species of this genus are, however, so variable that I do not venture to make a new one of this’. Perhaps swayed by Hooker’s views, Kirk (1899) did not name it at species rank. Nevertheless in his protologue he remarked (p. 125) that ‘this is probably a distinct species’. No other species has the same combination of densely crowded erect, plumose, dark green to silvery grey branches and branchlets (Fig. 32F), covered in masses of hairy linear leaves, sessile to subsessile small flowers with suberect, crumpled petals that are borne on mainly spiciform, condensed botrya, with long linear to linear-falcate pherophylls (Figs 30A–B, 32K–L). Herbarium specimens of *Kunzea linearis* are particularly distinctive because they usually turn silvery-grey on drying, a colour caused by the abundance of light-reflecting silky hairs on the branchlets and leaves. In addition to these differences, *Kunzea linearis* is further distinguished by its unique chromosome complement comprising eight ‘large’ (1.2–1.5 μm), and three small (0.8–0.9 μm) chromosome pairs. Of the sequence regions investigated (see de Lange 2007; de Lange et al. 2010), ETS was the only site showing variation (Table 2), with *Kunzea linearis* differing from all other Kunzea Subgen. Niviferae at alignment positions 41 and 259 where a unique guanine nucleotide and guanine/adenine mix are present (de Lange 2007). Otherwise, *Kunzea linearis* shares with *Kunzea ericoides* a cytosine nucleotide at alignment position 269 (Table 2), and with Mt Egmont samples of *Kunzea robusta*, and multiple samples of *Kunzea ericoides*, *Kunzea salterae*, *Kunzea serotina* and *Kunzea toelkenii* a guanine/cytosine mix at position 232 (Table 2).

*Kunzea linearis* is frequently sympatric with *Kunzea amathicola* and *Kunzea robusta*, and less commonly with *Kunzea sinclairii* on Aotea (Great Barrier Island). It is easily distinguished from all three species in the field and the herbarium by the linear leaves, inflorescence type, pherophylls and floral features (Figs 30A–B, 32K–L; Table 1). *Kunzea linearis* has a superficial resemblance to *Kunzea ericoides*, because both species have somewhat similar long narrow leaves, such that they have been confused in past literature e.g., de Lange et al. (1997). *Kunzea linearis* differs from the allopatric South Island endemic *Kunzea ericoides* by its long, silky, antrorse-appressed, weakly flexuose branchlet hairs (Figs 30C, 31A–E), consistently dark green to almost glaucous linear leaves densely crowded toward the branchlet apices, usually condensed spiciform botrya (Figs 30A–B, 32K–L), sessile to subsessile flowers with the calyx lobes of the mature bud erect, apically pinched inwards and touching just prior to bud burst (Figs 30A, I–K, 32K–L), suberect petals, and by the usually hairy hypanthia and fruits (Fig. 30J, N).

*Kunzea linearis* has some similarity to the allopatric Three Kings Island group endemic *Kunzea triregensis*, especially as the latter sometimes has flower buds with suberect touching calyx lobes. Although the two species never meet in the wild, they have been confused in herbaria. Differences between both species are discussed in more detail under *Kunzea triregensis*.

#### Ecology.

*Kunzea linearis* is primarily a species of coastal to lowland shrubland habitats overlying impoverished soils (Fig. 32B) and peat bogs. It is only very rarely found at any distance inland. The sole exception appears to be Te Paki where it is virtually the only *Kunzea* species present and so seems to occupy a much greater range of habitats than it would usually (e.g., Fig. 32A). Elsewhere within its range, even in apparently suitable inland gumland scrub habitats overlying leached soils, and on the clay podzols of the Northland Peninsula, it is usually replaced by *Kunzea robusta*. *Kunzea linearis* seems to reach its greatest abundance on sand podzols overlying older usually Pleistocene-aged sand dunes, especially in places where these grade into peat. Because it is tolerant of seasonal flooding, waterlogged soils and extreme drought *Kunzea linearis* is usually the dominant species on the sand country of the Te Aupouri Peninsula, as well as the acidic leached clays and older sand soils of Te Paki. It is also the dominant woody shrub on the margins of the oligotrophic peat bogs and lakes of the Taumatatotara Flats (Te Paki), the Motutangi-Kaimaumau Peat Bog, Lake Ohia, Karikari Peninsula lakes and in parts of the Ahipara Gumlands. Outside these habitats *Kunzea linearis* has been found growing within shell banks and low-lying clay banks subject to saline inundation within the mangrove (Avicennia marina subsp. australasica (Walp.) J.Everett) swamps of the upper Whangaroa Harbour. In western Northland it may occasionally colonise mobile sand where it is then usually sympatric with and often out-competed by *Kunzea amathicola*. In parts of Te Paki and also on the Poor Knights Islands, *Kunzea linearis* can sometimes be found in abundance within mixed indigenous forests, though mostly then on skeletal soils developed on outcrops of hard volcanic rock or on deeply leached clay podzols (usually in association with kauri (*Agathis australis* (D.Don) Lindl.)). These situations are exceptional and, as a rule, *Kunzea linearis* is not found in mature forests. South of the Pouto Peninsula and Te Arai, *Kunzea linearis* has a very patchy. In these areas it is usually found on cliff faces growing amongst pohutukawa (*Metrosideros excelsa* Sol. ex Gaertn.). In places where the cliffs abut land that has been frequently fired, *Kunzea linearis* may be a local component of the fire-induced gumland vegetation. The peculiar disjunct distribution of *Kunzea linearis* south of its main Northland occurrences, and in particular the close association of the Waitemata Harbour populations with sites of former Maori habitation and fortifications, e.g., Ahatawapa and Kendal’s Bay (Fig. 32B), and some of the original sites of European settlement e.g., Devonport, Cox’s Creek, led de Lange (2006) to suggest that these *Kunzea linearis* populations were not natural and may have resulted from the accidental spread of seed from firewood bought by Maori to the Waitemata Harbour from the eastern part of coastal Northland during the musket wars that raged between 1810 and the close of the 1830s. While this requires further study, the majority of these southerly occurrences are in habitats not usually occupied by the species in the main part of its range, and that also invariably occur on or close to cultural sites. Alternatively it could be natural to these areas, and may have temporarily expanded its range during the initial settlement phase of Auckland to occupy freshly cleared land. However, this explanation does not address the peculiar patchy distribution of the species on Aotea (Great Barrier Island), the Coromandel Peninsula, western Hauraki Plains and the foothills of the Hapuakohe Range, where successional habitats are still common, nor its peculiar disjunction to Mt Kupukore in the eastern Wairarapa (see Fig. 33).

*Kunzea linearis* is sometimes heavily parasitised by the hemiparasitic dwarf mistletoe *Korthalsella salicornioides*. In the northern part of its range it is often festooned in dense tangles of the lauraceous hemiparasitic taihoa (*Cassytha paniculata* R.Br. and *Cassytha pubescens* R.Br.). Around Te Paki *Kunzea linearis* provides an important habitat for an unnamed green gecko (*Naultinus* “Te Paki”), and elsewhere in Te Aupouri the Northland green gecko (*Naultinus grayi* Bell, 1843) (R. Hitchmough pers. comm.) whilst around Auckland it is a favoured habitat for another gecko, *Naultinus elegans* (Gray, 1842). Two geckos of the genus *Dactylocnemis* Fitzinger, 1861 (*Dactylocnemis pacificus* (Gray, 1842) and *Dactylocnemis* “North Cape”) and one of *Mokopirirakau* (*Mokopirirakau granulatus* (Gray, 1845)) are also commonly found sheltering under the bark of this species (R. Hitchmough pers. comm.).

#### Hybridism.

*Kunzea linearis* is a widespread species of northern New Zealand, and it is frequently sympatric with *Kunzea amathicola* in the western part of its range and with *Kunzea robusta* in the east. Throughout this range, but especially in places of prolonged human disturbance, the putative hybrids *Kunzea amathicola* × *Kunzea linearis* and *Kunzea linearis* × *Kunzea robusta* can be abundant. This observation is borne out by artificial hybridisation which showed that, whether used as a staminate or pistillate parent, *Kunzea linearis* readily formed hybrids with five of the seven other New Zealand *Kunzea* used in that study (de Lange et al. 2005).

Because *Kunzea linearis* hybrids are fully fertile there is a tendency for introgressed populations to develop, especially where local habitat conditions are prone to regular disturbance. Thus, complex introgressive hybrid swarms may occur in places that are frequently burned, subject to plantation forestry, coastal subdivision or urban development. Where conditions are extreme, such as the heavily developed northern shores of the Waitemata Harbour, Auckland, it is now difficult to find ‘pure’ examples of *Kunzea linearis*, as introgressed hybrid plants are dominant over much of that area.

The most commonly encountered hybrid is *Kunzea linearis* × *Kunzea robusta*. This is recognised by its foliage, which tends to be ascending rather than spreading, dark green, linear-oblanceolate rather than linear, and which has obtuse rather than acute apices. Foliar hair distribution is also markedly more variable on hybrid specimens, ranging from glabrate to distinctly sericeous hairy but with the hairs generally more restricted to the leaf margins and abaxial midribs. All putative hybrids, when fresh, have glossy leaves rather than the more usual dull dark green to silvery-grey leaf surfaces typical of *Kunzea linearis*. Flowering material is especially diagnostic, with the inflorescences on single individuals varying from elongate spiciform to compact corymbiform. The flowers tend to be shortly pedicellate, never sessile to subsessile, and the hypanthia broadly obconic to broadly barrel-shaped rather than barrel-shaped to sharply obconic. The hypanthia and fruit surfaces usually show a mixture of the short, antrorse-appressed hairs typical of *Kunzea robusta* and the long, sericeous, weakly flexuose, antrorse-appressed hairs of *Kunzea linearis*. In some examples the hypanthium surface may even be glabrate. An important distinction is the shape of the calyx lobes in mature buds. In *Kunzea linearis* these are consistently narrowly deltoid with distinctly acute apices, and in *Kunzea robusta*, broadly obtuse to rounded. In the hybrid they tend to be broadly deltoid with subacute to rounded apices. As with *Kunzea robusta*, the calyx lobes of the mature flower buds in hybrids tend to lie flat, though a few may be suberect, and, unlike *Kunzea linearis*, the lobes are rarely touching at bud burst. The petals of the hybrids tend to be larger than the range seen in *Kunzea linearis* and spreading rather than suberect, but, as with *Kunzea linearis*, they are often flushed pink or off-white with the margins finely crumpled. Depending on the degree of introgression, most hybrids can be readily identified by these characters.

The hybrid *Kunzea amathicola* × *Kunzea linearis* is common only in a small area between Waipapakauri, Ahipara and the adjacent, heavily modified Ahipara Plateau. Although this hybrid is fully described under *Kunzea amathicola*, some of the key diagnostic features are noted here to assist with distinguishing it from *Kunzea linearis*. *Kunzea amathicola* × *Kunzea linearis* is best recognised vegetatively by its leaves which are narrow to broadly lanceolate rather than linear to oblong, oblong-obovate to elliptic. Also they tend to be less evenly spaced than is usual for *Kunzea amathicola*, and, as in *Kunzea linearis*, are more crowded toward the branchlet apices. The shape of the pherophylls is diagnostic. Unlike *Kunzea linearis* which has linear to linear-falcate, ascending to spreading pherophylls, or *Kunzea amathicola* which has oblong, oblong-obovate, to elliptic, recurved ones, those of the hybrid are linear-oblong and spreading to weakly falcate. The flowers of *Kunzea linearis* are sessile to subsessile, and those of *Kunzea amathicola* are distinctly long pedicellate; hybrid flowers show a gradation from subsessile to shortly pedicellate (often on the same plant), and the hypanthium, calyx lobes and petals are also intermediate (see under *Kunzea amathicola*). The most critical difference is the shape and position of the calyx lobes, which are narrowly deltoid and erect in *Kunzea linearis*, broadly obtuse to rounded and suberect or spreading in *Kunzea amathicola*, and narrowly obtuse and suberect to erect in the hybrid. Further, as with *Kunzea amathicola*, the calyx lobes of fruiting hybrids are incurved from the base.

The hybrid *Kunzea linearis* × *Kunzea sinclairii* is very uncommon. Four specimens have been found on the western side of Aotea (Great Barrier Island), two flowering examples collected at “Fitzroy” (W. R. B. Oliver s.n. (WELT SP029478), W. R. B. Oliver s.n. (WELT SP029494)), and two sterile gatherings, one each from near Mt Young and Maungapiko. Oliver’s gatherings are the only wild flowering specimens of this hybrid known. The other examples are sterile but their hybrid status is evident by their distinctive foliage, and, in the one wild example I found, weakly erect, spreading, small tree habit. The foliage of all four specimens is distinctly narrow-lanceolate to almost linear, reddish silvery-grey, and copiously covered in long silky hairs. The leaf apices are sharply acute, and the margins have distinctly longer hairs than the rest of the lamina. Artificially raised hybrids of this combination were fully fertile (e.g., P. J. de Lange 5776 (AK 284581)), and produced shortly pedicellate flowers on somewhat spiciform inflorescences. The pherophylls ranged from broadly elliptic to lanceolate, and, as in *Kunzea sinclairii*, they are quickly shed, being present only in the early stages of floral bud development. The flowers of wild and experimental *Kunzea linearis* × *Kunzea sinclairii* hybrids are smaller than is usual in *Kunzea sinclairii* with hypanthia that are more narrowly obconic to campanulate, red-pigmented and copiously covered in long, antrorse-appressed hairs. The calyx lobes are suberect to erect, broadly deltoid with acute apices and very hairy margins. The lobes are very hairy along the centre, either side of which is a glabrous pale pink band. Often there is a small, deciduous apiculus.

#### Vernacular names.

Until recently northern Maori (specifically Te Rarawa of Te Aupouri and Ngati Kuri of Te Paki), did not recognise the name ‘kanuka’ for any species of *Kunzea*. All species of *Kunzea* from that region were universally known there as ‘manuka’, while *Leptospermum scoparium* (usually known outside this area now as ‘manuka’) is known there as ‘kahikatoa’ (G. Neho pers. comm.). While Ngati Kuri usually refer to *Kunzea linearis* as ‘manuka’ it is also known there by the name ‘rawiri’ (W. Murray pers. comm.). Rawiri was also a Nga Puhi name recorded on specimens of this species collected by the Cunningham’s from either the Bay of Islands or the Hokianga (Cunningham 1839; p. 111).

#### Conservation status.

*Kunzea linearis* is appropriately listed as ‘At Risk/Declining’ by de Lange et al. (2013b).

### 7. Kunzea amathicola

Taxon classificationPlantaeMyrtalesMyrtaceae

de Lange et Toelken sp. nov.

urn:lsid:ipni.org:names:77141731-1

A K. ericoides habitu heterophyllo, ramulis juvenilibus persistentibus plerumque fores efferentibus, indumento ramulorum persistenti longo sericeo antrorso appresso, indumento foliorum sericeo e vittis marginalibus et costis abaxialibus, inflorescentibus elongatis, bracteis floralibus oblongis vel late obovatis vel ellipticis differt. Etiam ordine rDNA ETS unico in sectione Niviferae recedit.

#### Holotype

**Figure 34.**
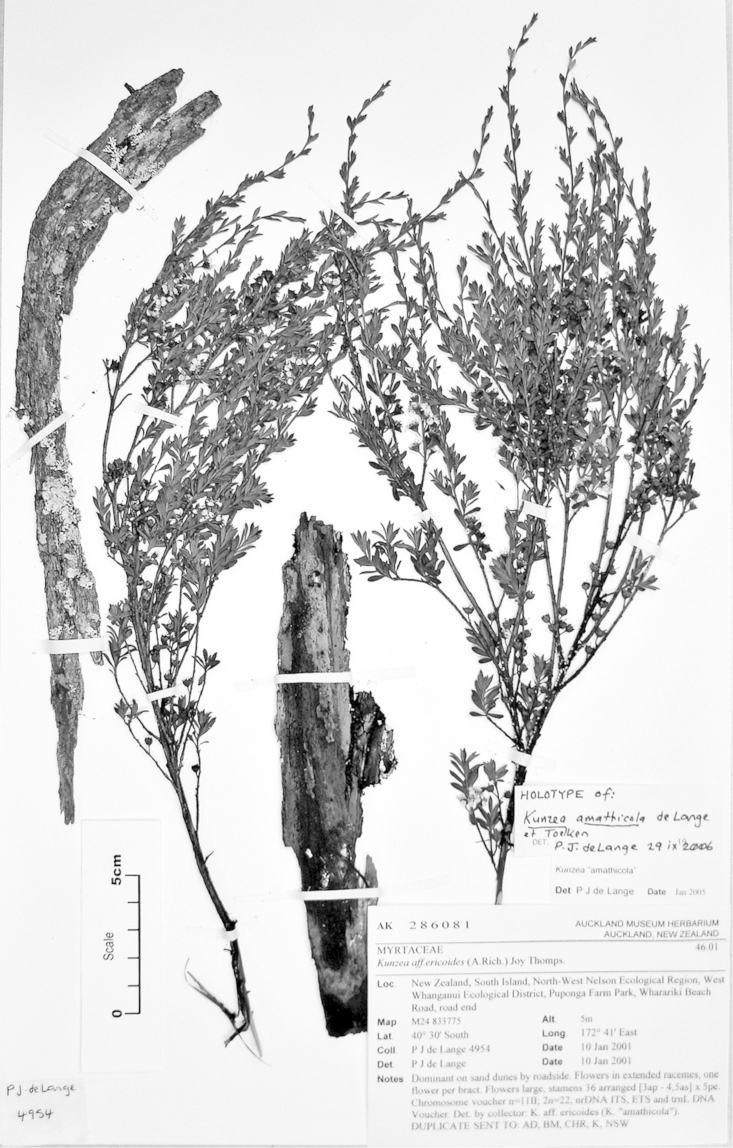
Holotype of *Kunzea amathicola* de Lange et Toelken (*P. J. de Lange 4954*, AK 286081).

**(Fig. 34).** New Zealand, South Island, Puponga Farm Park, Wharariki Beach Road, road end, 40°30'S, 172°41'E, 5 m a.s.l. ‘Dominant on sand dunes by roadside. Flowers in extended racemes, one flower per bract’. P. J. de Lange 4954, 10 Jan 2001, AK 286081! Isotypes: AD! BM! CHR! K! NSW!

#### Etymology.

The specific epithet *amathicola* meaning ‘sand dwelling’, alludes to the mainly sand-dominated habitat preferentially occupied by this species.

#### Description

**Figure 35.**
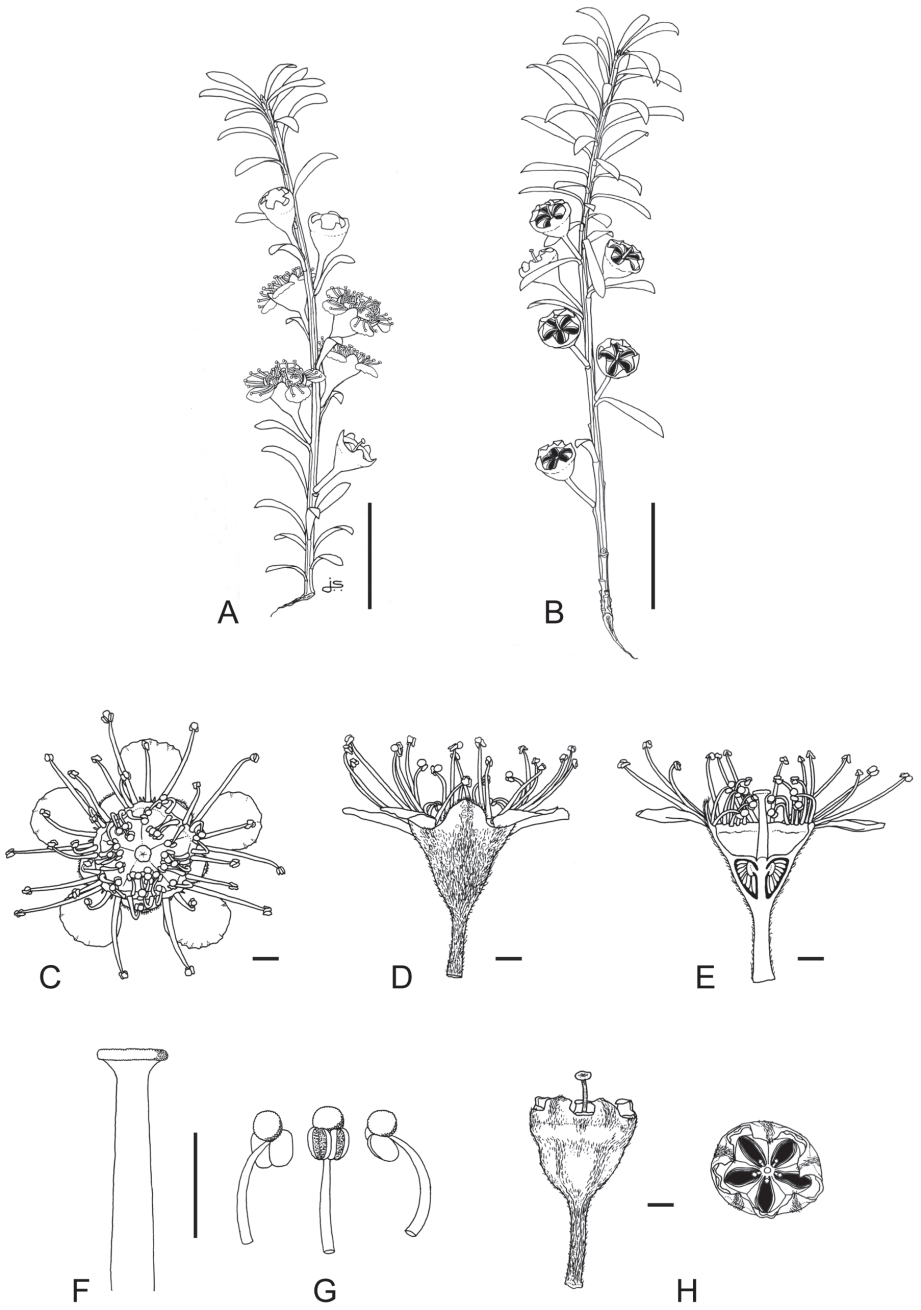
Distinguishing features of *Kunzea amathicola*. **A** Flowering branchlet (no voucher, North Island, South Kaipara Peninsula, Kaipara Harbour) **B** Fruiting branchlet (no voucher, North Island, South Kaipara Peninsula, Kaipara Harbour) **C** flower (top view) (no voucher, North Island, South Kaipara Peninsula, Kaipara Harbour) **D** Flower and hypanthium (side view) (no voucher, North Island, South Kaipara Peninsula, Kaipara Harbour) **E** Flower cross section showing anther, style and ovules (no voucher, North Island, South Kaipara Peninsula, Kaipara Harbour) **F** Style and stigma (no voucher, North Island, South Kaipara Peninsula, Kaipara Harbour) **G** Stamens (no voucher, North Island, South Kaipara Peninsula, Kaipara Harbour) **H** Dehisced fruit (no voucher, North Island, South Kaipara Peninsula, Kaipara Harbour). Scale bars: (**A, B**) 10 mm; (**C–H**) 1 mm.

**Figure 36.**
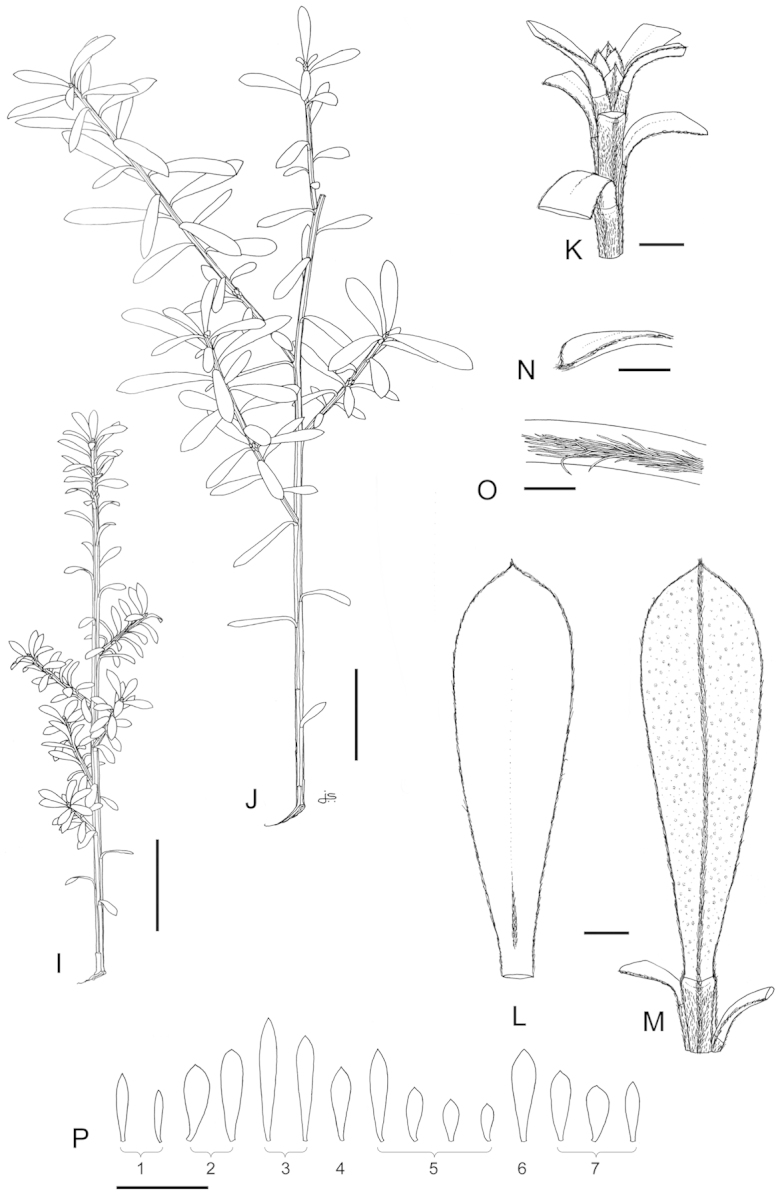
Distinguishing features of *Kunzea amathicola* continued (see Fig. 35). **I** Juvenile foliage (AK 289328) **J** Adult foliage (AK 289679) **K** Vegetative bud and branchlet indumentum (no voucher, North Island, South Kaipara Peninsula, Kaipara Harbour) **L** Adaxial leaf surface (no voucher, North Island, South Kaipara Peninsula, Kaipara Harbour) **M** Abaxial leaf surface (no voucher, North Island, South Kaipara Peninsula, Kaipara Harbour) **N** Adaxial leaf apex (no voucher, North Island, South Kaipara Peninsula, Kaipara Harbour) **O** Leaf margin indumentum (no voucher, North Island, South Kaipara Peninsula, Kaipara Harbour) **P** Leaf variation: (**P1**) North Island, Hokianga (AK 282676), (**P2**) North Island, Kaipara (AK 289669), (**P3**) North Island, Te Toto Gorge (AK 284417), (**P4**) North Island, Hokio (AK 286079), (**P5**) North Island, Hokio—last two leaves from a juvenile reversion shoot (AK 289679), (**P6**) South Island, Farewell Spit (AK 289243), (**P7**) South Island, Wharariki (AK 286081). Scale bars: (**I, J, P**) 10 mm; (**K, N, L, M**) 1 mm; (**O**) 0.5 mm.

**Figure 37.**
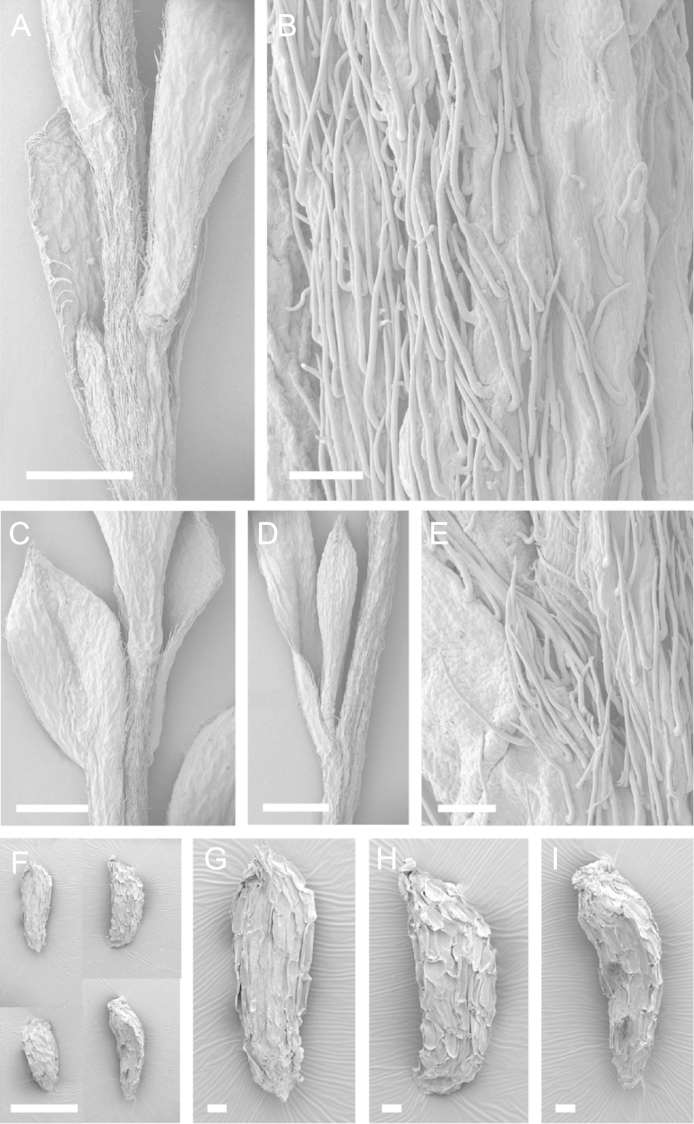
Scanning Electron Micrographs of *Kunzea amathicola*. (**A–E** all AK 286079) Branchlet indumentum **F–H** seeds (AK 289669). Scale bars: (**A, C, D, F**) 1 mm; (**B, E, G–I**) 100 μm.

**Figure 38.**
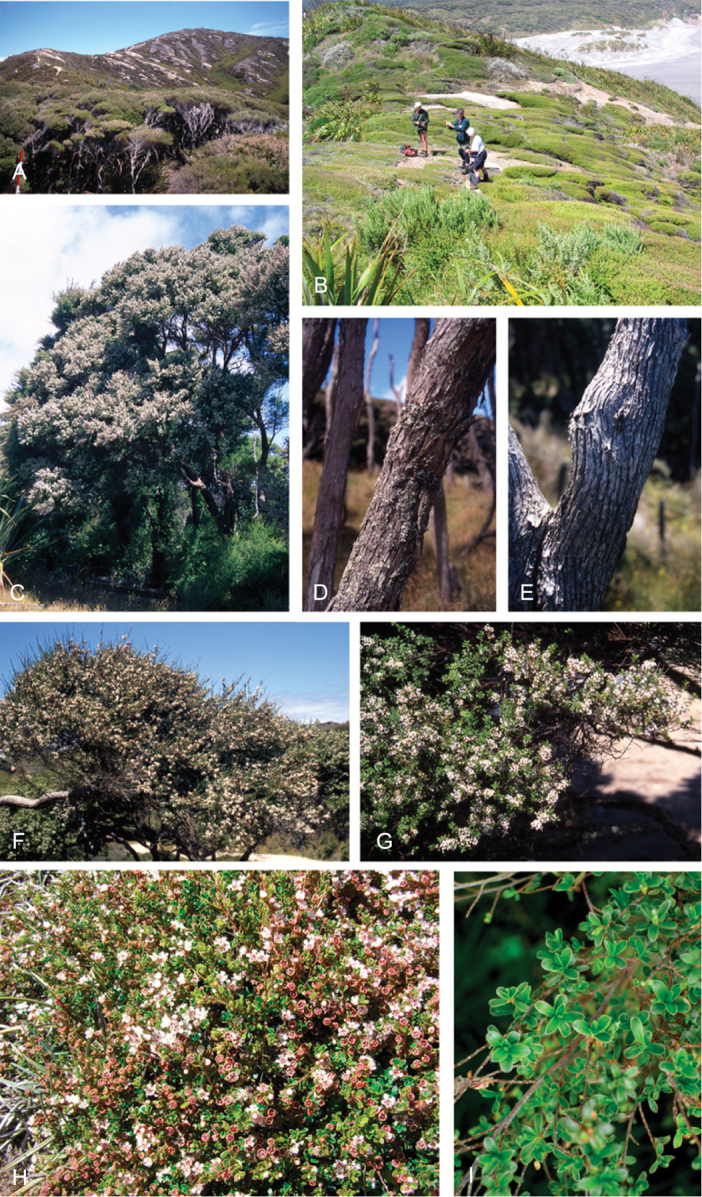
*Kunzea amathicola*. **A** *Kunzea amathicola* forming dominant vegetation on impoverished ‘badlands’ that developed after coal mining operations, South Island, north-west Nelson, Puponga, track to Pillar Light (photo: *P. J. de Lange*) **B** Decumbent, permanently juvenile shrubs of *Kunzea amathicola* growing on hard clays overlying calcareous mudstones on a small islet, South Island, north-west Nelson, Wharariki Beach (photo: *M. D. Wilcox*) **C** Adult tree of *Kunzea amathicola* in full flower, South Island, north-west Nelson, at base of Farewell Spit (photo: *G. M. Crowcroft*) **D–E** Bark of *Kunzea amathicola*, South Island, north-west Nelson, Kaihoka Lakes (photo: *P. J. de Lange*) **F–G** Flowering adult *Kunzea amathicola* branches of holotype at type locality, South Island, north-west Nelson, Puponga Farm Park, Wharariki Beach Road (photo: *P. J. de Lange*) **H** Juvenile flowering branches of *Kunzea amathicola*, on a small islet, South Island, north-west Nelson, Wharariki Beach (photo: *M. D. Wilcox*) **J** *Kunzea amathicola* foliage, North Island, Kaipara Harbour, near Kaukapakapa (photo: *P. J. de Lange*).

**(Figs 35, 36, 37, 38).** *Growth habit* Shrubs or trees up to 15 m tall; heterophyllous (bearing distinct juvenile and adult foliage types). Those with persistent juvenile foliage mostly present in exposed conditions and unstable habitats, or at the margins of adult stands, usually forming domed, spreading shrubs up to 2 × 3 m with numerous erect to ascending, often interwoven branches; those with adult foliage forming single to multi-trunked trees up to 18 × 8 m, with very broad, spreading canopies. Irrespective of growth habit, plants flowering at a young age (1–2 years old). *Trunk* 1(–2) in juveniles usually branched from or close to base, in adults usually devoid of branches in lower 30–50%; 0.10–0.60(–0.85) m d.b.h., initially erect but soon arching outwards; basal portion covered with firm to semi-detached, tessellated, short to long, tabular to ± irregularly tabular lengths of corky-coriaceous bark. *Bark* early bark chartaceous to subcoriaceous, grey or grey-brown, ± elongate, usually bearing a few transverse cracks (especially on branch flanges and decurrent leaf bases) otherwise remaining firmly attached, margins elongate sinuous, ± entire with scarcely any flaking; old bark similar though distinctly corky-coriaceous, usually tessellated, firmly attached, detaching basally with age, and peeling upwards along trunk in broad, tabular strips, margins ± entire to weakly irregular; upper surface often deeply corrugated and cracked but not peeling; margins somewhat sinuous to ± straight; early and old bark flakes firm, not crumbling in hand, snapping with ± entire margin. *Branches* juvenile branches numerous, erect to suberect not spreading, often interwoven; adult branches usually confined to the upper 30–50% of trunk; initially suberect, soon arching and spreading, often weakly flexuose; branchlets numerous, slender, ± quadrangular to subterete, branchlet indumentum copious, persistent; hairs silky, antrorse-appressed, usually flexuose, (0.23–)0.38(–0.50) mm long, hyaline to translucent (appearing white when young, maturing grey). Juvenile branchlets numerous, erect to suberect, often interwoven, leaves ± evenly spaced along length or, in exposed situation, crowded toward apices; adult branchlets clustered toward branch ends, weakly flexuose, with leaves ± evenly spaced along length. *Vegetative buds* conspicuous; at resting stage 0.8–1.0 mm diam.; scales scarious, deciduous, 0.5–0.8 mm long, amber to red-brown, broadly ovate, ovate-deltoid to rostrate; midrib prominent, strongly keeled in upper half, prolonged to short cuspidate tip, lateral veins absent, oil glands few, scattered, colourless, drying dull yellow; scale margins, keel, and keel apex copiously covered in long, white, silky hairs. *Leaves* sessile to shortly petiolate, well-spaced to crowded along branchlets, spreading, sub erect to patent, strongly recurved in distal 30–50%, dark glossy green above, much paler beneath with margins and abaxial midrib distinctly white-coloured due to dense hair growth. Juvenile lamina (2.4–)3.4(–5.3) × (1.2–)1.9(–2.3) mm, ovate, broadly ovate, rhomboid to obovate, adult lamina (6.0–)8.2(–12.5) × (1.8–)2.6(–3.8) mm, oblong, oblong-obovate, broadly oblanceolate to broadly lanceolate; apex of both juvenile and adult lamina obtuse, rounded to subacute, rostrate, base attenuate to narrowly attenuate; adaxial surface convex, weakly plicate, or strongly v-shaped in distal recurved portion, oil glands not evident when fresh, midrib very slightly raised near base, otherwise not evident for rest of length, basally finely covered in antrorse-appressed, silky hairs, otherwise glabrous; abaxial surface slightly to prominently concave in distal recurved portion otherwise weakly concave, finely glandular punctate, oil glands sparse 80–200, more evident when dry; midrib slightly raised for entire length, prolonged slightly at apex, densely sericeous, hairs continuing to leaf apex, hairs weakly flexuose, antrorse, subappressed, up to 0.3 mm long, hyaline to translucent, appearing as white to naked eye; lamina margin completely obscured by a dense covering of antrorse-appressed hairs aligned in a thick, up to 0.6 mm wide, white, plumose band meeting with abaxial midrib hairs at the leaf apex. *Perules* deciduous, rarely persistent, squamiform; scales scarious, 0.5–0.8 mm long, amber to red-brown, broadly ovate, ovate-deltoid to rostrate; midrib prominent, strongly keeled in upper half, prolonged to short cuspidate tip, lateral veins absent, oil glands few, scattered, colourless, drying dull yellow, scale margins, keel, and keel apex copiously covered in long, white, silky hairs. *Inflorescence* Usually a well-spaced, elongate, (5–)12(–20)-flowered botryum up to 200 mm long, in adverse conditions sometimes becoming a condensed raceme 30–60 mm long, with the flowers shortly spaced and overlapping; in non-stressed conditions the terminal portion of the inflorescence comprising an indeterminate length of vegetative growth and sometimes a few undeveloped flowers. Inflorescence axis densely invested with silky, antrorse-appressed, weakly flexuose hairs. *Pherophylls* persistent, foliose, spreading, dark glossy green, oblong, oblong-obovate, broadly obovate to elliptic; strongly recurved, to about half of total length or flat; juvenile lamina (2.0–)3.4(–5.3) × (1.2–)1.9(–2.3) mm, adult lamina (4.1–)5.4(–6.0) × (1.6–)2.3(–3.1) mm; apex obtuse, cuspidate, base attenuate; adaxial surface usually convex to weakly plicate, oil glands not evident when fresh or dry, midrib slightly raised near base, otherwise not evident for rest of length, basally covered in a dense weft of antrorse-appressed, silky hairs; abaxial surface flat or weakly convex, glandular punctate, oil glands 20–40, more evident when dry; midrib raised for most of length, densely covered in antrorse-appressed, sericeous hairs to apex, lamina margin obscured by dense covering of antrorse-appressed hairs. *Pedicels* (1.3–)3.4(–4.9) mm long at anthesis, usually elongating slightly after anthesis, terete, sparsely to densely invested in antrorse-appressed, weakly flexuose, silky hairs. *Flower buds* pyriform to hemispherical, apex usually flat or weakly domed prior to bud burst; calyx valves not meeting. Fresh flowers when fully expanded (6.8–)11.6(–12.5) mm diam., usually reducing in size toward end of flowering season. *Hypanthium* (1.9–)2.8(–4.0) × (3.0–)4.0(–5.6) mm, with free portion 0.7–1.3 mm long, dark green or red-green, drying green-brown or red-brown; broadly obconic, turbinate to hemispherical, terminating in dark-green to red-green coriaceous rim bearing five persistent suberect to spreading calyx lobes; fresh hypanthium surface faintly ribbed and sparingly dotted with pink or colourless oil glands, these drying dull yellow, ribs and veins usually densely covered in silky, antrorse-appressed hairs, sometimes glabrous; dry hypanthium surface similar though with the ribs more strongly defined, clearly leading up to calyx lobes. Calyx lobes 5(–8), suberect to spreading, coriaceous, (0.6–)1.2(–1.4) × (0.6–)1.0(–1.8) mm, persistent, ovate, ovate-truncate to broadly obtuse, pale green to red-green, weakly to strong keeled, external face of keel usually obscured by a broad band of antrorse-appressed, silky, white hairs, otherwise glabrous; margins white, pale green often flushed pink, surface somewhat sparsely glandular punctate, oil glands ± colourless when fresh drying dull yellow, otherwise (aside from keel) glabrescent. Receptacle green at anthesis, consistently darkening to crimson after fertilisation. *Petals* 5(–8), (1.8–)2.6(–3.7) × (2.0–)2.7(–4.0) mm, white (often drying butter yellow), spreading, orbicular to broadly ovate, apex rounded, margins ± finely and irregularly denticulate or crimped 1–6 or more times, oil glands colourless, drying opaque. *Stamens* 38–60(–90) in 2(–3) weakly defined whorls, arising from receptacular rim, filaments white. Antipetalous stamens 3–5(–6) sometimes petaloid, antisepalous stamens (5–)8(–10). Outermost antipetalous stamens usually outcurved, sometimes weakly incurved or in mixtures of both on filaments 1.5–2.4 mm long, inner stamens usually at the base of the outermost antipetalous pair (0.6–)0.8–1.2 mm long, weakly incurved. Antisepalous stamens mostly shorter than outermost antipetalous stamens, sometimes of comparable length, generally 0.6–1.2 mm long, weakly to strongly incurved, very rarely a few outcurved. Anthers dorsifixed, 0.40–0.60 × 0.20–0.35 mm, ellipsoid, ovoid-ellipsoid or broadly scutiform, latrorse. Pollen white (9.9–)14.8(–18.9) μm. Anther connective gland prominent, deep golden-yellow to orange when fresh, drying orange to pink, spheroidal, rather finely papillate, sometimes absent. *Ovary* 5(–6) locular, each with 23–28(–42) ovules in two rows on each placental lobe. Style 2.0–2.5(–3.2) mm long at anthesis, elongating slightly after anthesis, white or pinkish-white; stigma broadly capitate, at least 1.5× width of style, flat, greenish-white or pale pink, flushing red after anthesis, surface finely granular-papillate. *Fruits* long persistent, (2.4–)3.9(–4.8) × (3.6–)4.8(–6.0) mm, initially dark green to chesnut-brown fading with age to grey, broadly obconic, turbinate or hemispherical, rarely broadly cupular; veins and ribs conspicuous on drying, these finely hairy to glabrescent, hairs antrorse-appressed; calyx valves incurved, splits concealed by dried, erect, free portion of hypanthium. *Seeds* 1.2–1.5(–1.7) × 0.3–0.4(–0.6) mm, testa semi-glossy, orange-brown to dark brown, oblong, oblong-obovate, narrowly ellipsoid to cylindrical, ± curved near apex, laterally compressed, 2–3-angled with convex to flattened faces, apex rounded to subacute; base oblique, ± flattened. Surface coarsely reticulate. FL: (Jul–)Nov–Jan(–Jun). FT: (Aug–)Nov–Jan(–Jun). Chromosome Number *n* = 11_II_, 2*n* = 22 (see de Lange and Murray 2004).

#### Representative specimens

**(88 sheets seen).** **North Island.** Te Aupouri Peninsula, Hukatere, K. G. Matthews s.n., 30 Aug 2005, (AK 293310); Ahipara, Shipwreck Bay, P. J. de Lange 4144, 17 Jan 2000, (AK 287967, Duplicate: AD); Hokianga, Outer South Head Walkway, P. J. de Lange 5397 & T. T. J. B. Armstrong, (AK 282676); Pouto, near Lake Whakaneke, L. J. Forester s.n., 16 Nov 2000, (AK 252352); Kaipara Harbour, Okahukura Peninsula, Kahutaewao Creek, P. J. de Lange 6709, T. J. de Lange & F. J. T. de Lange, 29 Sep 2006, (AK 297616); Woodhill, Kaipara, W. R. B. Oliver s.n., 26 Dec 1912, (WELT SP029495); Port Waikato, R. Cooper s.n., 13 Feb 1965, (AK 121924); Te Rere Farm Station, Taranaki Bluffs, south of Rengarenga Stream mouth, P. J. de Lange 6882, 7 Feb 2002, (AK 298389); Kawhia Harbour, Puti Point Scenic Reserve, P. J. de Lange 5293, 26 Jun 2001, (AK 254924, Duplicate; AD); Awakino River, near Awakino, P. J. de Lange 6328 & T. T. J. B. Armstrong, 14 Sep 2001, (AK 289178); Horowhenua, Hokio, Hokio Beach Road, P. J. de Lange 4895, 7 Jan 2001, (AK 289230, Duplicate: AD); Kapiti [Island], W. R. B. Oliver s.n., 16 Jan 1935, (WELT SP06700); Pautahanui Inlet, Plimmerton Hills, near Cambourne Walkway, P. J. de Lange 4902, 8 Jan 2001, (AK 289332, Duplicate; AD). **South Island.** Farewell Spit, Bush End Point, P. J. de Lange 5015 & G. M. Crowcroft, 15 Jan 2001, (AK 289243, Duplicate: AD, MEL); Farewell Spit, Lagoon Creek, P. J. de Lange 5016 & G. M. Crowcroft, 15 Jan 2001, (AK 289691); Puponga Farm Park, Stone Bridge, P. J. de Lange 4973, 11 Jan 2001, (AK 286080, Duplicate: AD); Whanganui Inlet, Kaihoka Lakes, P. J. de Lange 4911, 9 Jan 2001, (AK 286083, Duplicate: CHR); Aorere Inlet, P. J. de Lange 4981, 11 Jan 2001, (AK 289242); Wainui Bay, Takapou inlet, P. J. de Lange 4993, 12 Jan 2001, (AK 289688, Duplicate: AD); Anatori River Mouth, P. J. de Lange 4913, 9 Jan 2001, (AK 289235, Duplicate: AD).

#### Distribution

**Figure 39.**
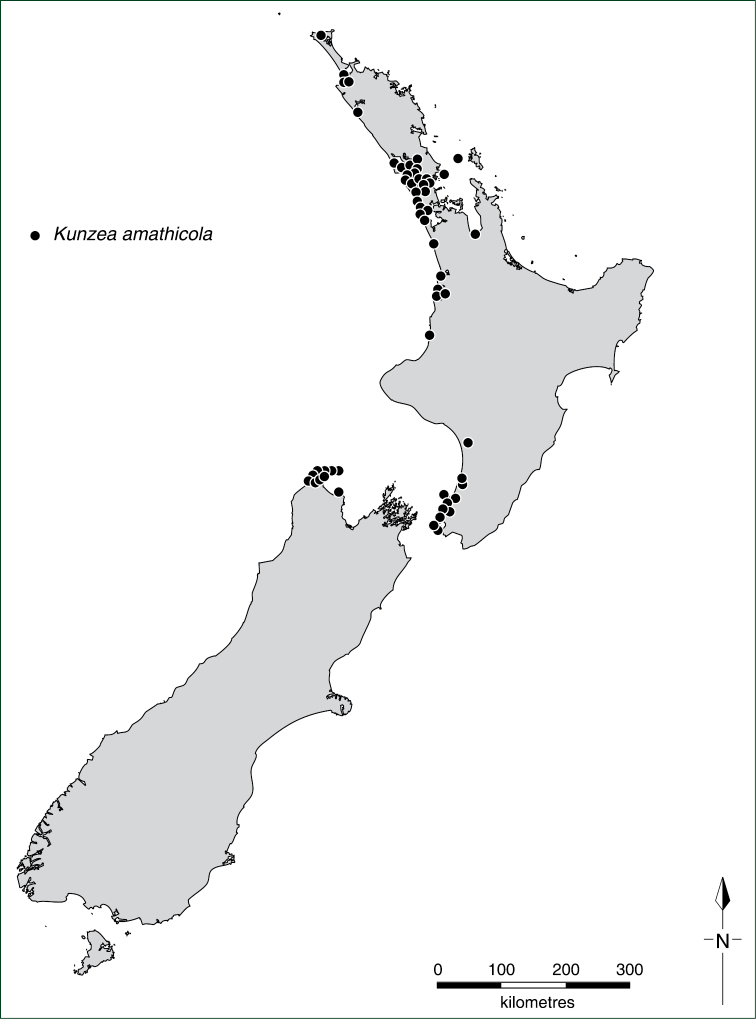
Distribution of *Kunzea amathicola*.

**(Fig. 39).** Endemic, New Zealand, North and South Islands (sea level – 320 m a.s.l.). In the North Island *Kunzea amathicola* is found mainly in the west, locally from Unuwhao Bush, Te Paki, south to Wellington City. In the South Island *Kunzea amathicola* is common in north-west Nelson from Farewell Spit to the Whanganui Inlet, and along the tidal reaches of the Aorere River. South of there it is confined to the Kaihoka–Kahurangi coastline. *Kunzea amathicola* has also been collected along the eastern side of the Kaipara Harbour where it extends up the main river valleys a considerable distance. This species has also been collected once from Kawau Island (*L. Esler s.n.*, AK 215754) and, from a tidal creek on the Hauraki Plains near Waitakaruru (e.g., *R. Mason s.n.*, CHR 112646).

#### Recognition.

*Kunzea amathicola* differs from the other New Zealand *Kunzea* species by its heterophyllous habit (with different juvenile and adult foliage types and the tendency for apparent juveniles to flower and fruit (Fig. 38B, H)), by the obovate leaves with distinctly hairy leaf margins and midribs (with the hairs meeting at the leaf apex rather than just short of it), and distinctive elongate inflorescences (Figs 35–36, 38; Table 1). *Kunzea amathicola* usually has a larger flower than the other New Zealand species, and it may also be found in flower throughout the year. Some of these distinctions are shared with *Kunzea linearis* and *Kunzea triregensis*, species with which it seems to be morphologically allied. Phylogenetically, *Kunzea amathicola* is sister to the other New Zealand taxa (de Lange 2007; de Lange et al. 2010). Indeed, the ITS sequence of *Kunzea amathicola* differs consistently (based on 15 samples spanning the species’ range) from all other Australian and New Zealand members of the *Kunzea ericoides* complex by one unique character (in ITS-1), a thiamine nucleotide at alignment position 671 (Table 2; see also de Lange 2007). Otherwise, at ITS-1 alignment position 742, *Kunzea amathicola* shares with *Kunzea salterae* and *Kunzea tenuicaulis*, a guanine/thiamine mix (Table 2). The ETS sequence of *Kunzea amathicola* has no unique characters. However, it shares a guanine nucleotide at position 68 with the Australian Niviferae subsect. Niviferae (de Lange 2007) and a single sample of a New Zealand *Kunzea* of uncertain status from Lottin Point near East Cape (Table 2). It also shares a guanine nucleotide at position 269 with *Kunzea sinclairii* and *Kunzea robusta* (de Lange 2007; de Lange et al. 2010).

*Kunzea amathicola* is widely sympatric (and often syntopic) with *Kunzea ericoides* (South Island, north-west Nelson only), *Kunzea linearis* (northern North Island only) and *Kunzea robusta* (throughout its range). Hybrids involving these species and *Kunzea amathicola* are uncommon in sites of sympatry, unless they are from places subjected to frequent disturbance, such as in areas of plantation forestry overlying dune fields, within coastal subdivisions or along roadsides,(see below). Throughout large parts of its range, particularly from the Waitakere coastline north to the northern end of the Pouto Peninsula, *Kunzea amathicola* is the dominant species of the sand country and associated peripheral hill country. In these areas *Kunzea amathicola* may exist in sometimes extensive, evidently self-perpetuating forests (see Smale et al. 1995 as Kunzea ericoides var. ericoides).

Field and herbarium recognition of *Kunzea amathicola* is straight forward (Table 1). Irrespective of whether plants have juvenile or adult leaves, the species can be recognised by the distinctive obovate to broadly elliptic, yellow-green to dark green leaves which are consistently glossy above and paler beneath, and by the lamina margins and the abaxial (and sometimes adaxial midribs) which are densely covered in white, silky, antrorse-appressed, weakly flexuose hairs, which meet at the leaf apices (Figs 35–36, 38II; Table 1). The leaf shape coupled with the characteristically elongate inflorescences, and broadly obovate to elliptic, persistent, foliose pherophylls are also diagnostic (Fig. 35A–B). The only other species to have consistently elongate inflorescences is the allopatric Three Kings endemic *Kunzea triregensis*, which has lanceolate to elliptic pherophylls, and inflorescences that may branch toward the base or near the apices into smaller elongate lateral or more rarely 3-flowered subcorymbiform botrya.

*Kunzea amathicola* is also the only heterophyllous New Zealand *Kunzea* that commonly flowers during its juvenile foliage phase.

*Kunzea amathicola* is distinguished from the linear-leaved *Kunzea linearis* by its leaf shape, heterophyllous growth habit, elongate inflorescences and widely spaced, pedicellate flowers. In examples of *Kunzea linearis* where the spiciform inflorescence has become elongated, the long-pedicellate condition of *Kunzea amathicola* is a major distinction from the sessile to subsessile flowers of *Kunzea linearis*. The flowers of both species also differ, those of *Kunzea amathicola* have spreading rather than suberect petals, and antipetalous stamens that have markedly longer filaments than those of *Kunzea linearis*.

*Kunzea amathicola* is distinguished from *Kunzea robusta* by its leaf shape and indumentum and by the consistently elongate botryum. Although *Kunzea robusta* is an extremely variable species, its leaves are rarely obovate, being mostly oblanceolate to lanceolate or linear-lanceolate, and, although some populations (e.g., the upper Rangitikei Valley and Mt Egmont – eastern Taranaki area) are markedly heterophyllous, none have the small obovate leaves of juvenile *Kunzea amathicola* plants. While the leaf margins of *Kunzea robusta* are usually hairy, the hairs are aligned in 1–2(–3) irregular rows, rather than the thick band of plumose hairs seen in *Kunzea amathicola*, and they never meet at the leaf apex. Further, as the leaves of *Kunzea robusta* mature, these marginal hairs are progressively shed so that in most cases they are present only in the lower one-third of the leaf. The inflorescence of *Kunzea robusta* is also mostly corymbiform, though in good flowering years or shade plants these corymbiform botrya usually partially elongate but never to the extent seen in *Kunzea amathicola*. Corymbiform botrya are never seen in *Kunzea amathicola*, and, although occasional plants may have shorter more ‘condensed’ inflorescences than is usual for this species, the flowers are always subtended by a persistent leaf-like, narrowly to broadly obovate pherophyll. The pherophylls of *Kunzea robusta*, are both squamiform and foliose (Table 1), with foliose ones mostly oblanceolate, broadly lanceolate to lanceolate, rather than mostly oblong, oblong-obovate, to elliptic (only rarely broadly lanceolate, as in *Kunzea amathicola*), and they are mostly shed during the early stages of flowering. Ecologically *Kunzea robusta* is a widely ranging species found from the coast through to montane areas, while *Kunzea amathicola* is mostly confined to coastal areas. Finally, *Kunzea robusta* is usually a tall forest tree commonly exceeding 20 m tall whereas *Kunzea amathicola* is a smaller tree or shrub rarely exceeding 12 m tall.

In the north-west Nelson part of its range, *Kunzea amathicola* is the only *Kunzea* species present from the tip of Farewell Spit to the northern end of the Whanganui inlet across to about Pakawau. South of there it is sympatric with *Kunzea ericoides*. While both species show some ecological partitioning, the hybrid *Kunzea amathicola* × *Kunzea ericoides* is common in more disturbed places where logging and past fires have significantly disrupted the vegetation (such as along roadsides, within coastal subdivisions, and along the north-eastern parts of the Whanganui Inlet). Distinction between *Kunzea ericoides* and *Kunzea amathicola* is straightforward (Table 1). Both species have markedly different branchlet indumentum. That of *Kunzea amathicola* is long, silky, antrorse-appressed and clearly visible to the naked eye, while *Kunzea ericoides* tends to be glabrate, the divergent hairs are minute and scarcely distinguishable without a 20× magnification lens. *Kunzea ericoides* is homophyllous; it has linear to linear-lanceolate, glabrate leaves, glabrate to glabrous obconic hypanthia, and much smaller flowers (up to 16 mm diameter) than *Kunzea amathicola*, with consistently fewer stamens (up to 34, usually 18). *Kunzea amathicola* is heterophyllous; it has obovate to elliptic, hairy leaves, and very hairy obconic hypanthia. The flowers of this species are much larger (up to 20 mm diameter) and in the field they have consistently more stamens (usually more than 40 and up to 80, rather than mostly 18 rarely up to 34).

#### Ecology.

*Kunzea amathicola* is primarily a coastal species of mobile sand and, usually Pleistocene-aged, stable sand dune systems. In the south-western North Island and north-western South Island, however, it also colonises greywacke soils, calcareous rocks, coal measures and their associated clay soils (Fig. 38A–B). It also colonises tidal river banks, coastal freshwater wetlands, estuaries (where it usually grows in the upper reaches of salt marshes with species such as *Olearia solandri* (Hook.f.) Hook.f. and *Plagianthus divaricatus* J.R.Forst. et G.Forst.), and may be prominent on exposed coastal headlands, cliff faces, and slip scars. More rarely it extends inland along river valleys where it colonises alluvial terraces. It reaches its maximum altitudinal limit on the windswept gumland scrub of the Ahipara Plateau where it has spread from the adjoining sand country up on to the figau. This habitat is probably more induced than truly natural, because at this location *Kunzea amathicola* is occupying ground that was once covered in kauri forest and which was burned repeatedly from the mid 1800s to early 1900s to facilitate better access for gum diggers (Sale 1978).

*Kunzea amathicola* is often the dominant tree species of dune systems in the western part of the North and northern South Island, where it appears routinely to form a distinct, stable vegetation type. *Kunzea amathicola* is well adapted for the sand environment. Plants grow quickly to form a dense ball of branchlets with no obviously dominant stem. Plants bearing juvenile foliage and flowers and fruits have been collected on mobile sand, on exposed coastal headlands, or even as part of the shrub tier under adult stands of the same species (e.g., P. J. de Lange 4341 & A. J. Townsend (AK 289328)).

*Kunzea amathicola* is sometimes parasitised by the green mistletoe (*Ileostylus micranthus* (Hook.f.) Tiegh.), dwarf mistletoe (*Korthalsella salicornioides*) and both species of taihoa (*Cassytha paniculata* and *Cassytha pubescens*).

#### Hybridism.

*Kunzea amathicola* is sympatric with and hybridises freely with *Kunzea ericoides*, *Kunzea linearis* and *Kunzea robusta*.

Recognition of *Kunzea amathicola* × *Kunzea ericoides* and *Kunzea amathicola* × *Kunzea linearis* in the field or herbarium is easy because both *Kunzea ericoides* and *Kunzea linearis* have linear, linear-lanceolate to narrowly lanceolate leaves and the leaves of hybrids are intermediate between those species and *Kunzea amathicola*. Further, because *Kunzea ericoides* has glabrescent branchlets usually sparingly covered in divergent hairs, hybrids with the distinctly hairy *Kunzea amathicola*, whose branchlets are copiously covered in long, silky, antrorse-appressed hairs, are easily recognised by the obvious mixtures of both hair types on the branchlets. Also, the elongate inflorescences of *Kunzea amathicola* are carried through in the hybrid such that plants have mixtures of subcorymbiform to completely elongate botrya. The leaf margins of *Kunzea amathicola* × *Kunzea ericoides* are also distinctive, typically rather hairy at first but with the hairs soon shedding, and rarely (if ever) meeting at the leaf apex. Foliage colour in hybrids also tends to retain the bright green typical of *Kunzea ericoides*, rather than the glossy dark green more usual for *Kunzea amathicola*. *Kunzea amathicola* × *Kunzea ericoides* is common around the more modified parts of the South Island at Golden Bay and the northern Whanganui Inlet. In particular there are complex introgressed swarms around Waikato, (to the north of the Aorere Lagoon, north-west Nelson), and between Pakawau and the north eastern reaches of the Whanganui Inlet. Otherwise, this hybrid is rarely seen, mainly because *Kunzea ericoides* rarely reaches the coast within the South Island range of *Kunzea amathicola*, and in the majority of places where it does reach the coast, *Kunzea amathicola* is absent.

Leaf, pherophyll, flower and hypanthia offer a wealth of useful characters enabling hybrid recognition of *Kunzea amathicola* × *Kunzea linearis*. However, because both parents have similar branchlet indumentum and the normally condensed spiciform inflorescences of *Kunzea linearis* may elongate toward the end of the flowering season, recognition of hybrids can be difficult. The leaves of *Kunzea amathicola* × *Kunzea linearis* hybrids are narrow to broadly lanceolate, and less evenly spaced than in *Kunzea amathicola* and, like *Kunzea linearis*, they tend to be more crowded toward the branchlet apices. The shape of the pherophylls is also diagnostic. In *Kunzea linearis* they are linear to linear-falcate and ascending to spreading; in *Kunzea amathicola* they are usually oblong, oblong-obovate, or elliptic, (rarely broadly lanceolate) and recurved. In the hybrid they tend to be linear-oblong and spreading to weakly falcate. Another distinction is the flowers. As the flowers of *Kunzea linearis* are sessile to subsessile, and those of *Kunzea amathicola* distinctly long-pedicellate, the hybrid can be recognised by the mixtures of sessile, subsessile to shortly pedicellate flowers. The hypanthia of *Kunzea amathicola* is typically broadly obconic, turbinate to hemispherical, while the flowers are up to 12.5 mm diameter, with white, orbicular to broadly ovate, spreading petals up to 3.7 × 4.0 mm. The antipetalous stamens of *Kunzea amathicola* are spreading and typically longer than the antisepalous stamens, while the style of *Kunzea amathicola* is very broad, and the capitate stigma obviously wider than the style diameter. *Kunzea linearis* has barrel-shaped, cupular or narrowly campanulate hypanthia up to 4.0 × 4.1 mm, much smaller flowers (up to 5.7 mm diameter), and cream, narrowly ovate to suborbicular, suberect to slightly recurved petals up to 2.0 × 1.9 mm. The stamens are mostly of similar length and tend to be erect rather than spreading, while the style of *Kunzea linearis* is rather narrow and the capitate stigma scarcely wider than the style diameter. Hybrids consequently tend to have broadly obconic to narrowly obconic or cupular hypanthia, of intermediate size ranges, and equally intermediate flower diameters, and petal sizes. The flower colour tends toward cream with the petals suberect to spreading, and longer than those of *Kunzea linearis*, with weakly spreading to strongly spreading, unevenly sized antipetalous stamens. The stigma of hybrid plants, as is typical of *Kunzea amathicola*, is mostly broadly capitate. The calyx lobes of the fruits of both species are also useful in distinguishing the hybrid. In *Kunzea linearis* the calyx lobes are narrowly deltoid, erect, or basally incurved toward the style remnant, while those of *Kunzea amathicola* are broadly obtuse to rounded and apically incurved toward the style remnant. In the hybrid the calyx lobes tend to be narrowly obtuse, suberect to erect and, as in *Kunzea amathicola*, they are apically incurved toward the style remnant. In many respects *Kunzea amathicola* × *Kunzea linearis* look morphology similar to *Kunzea triregensis*, and, as discussed under that species, it is postulated that *Kunzea triregensis* may have a hybrid ancestry involving both these species.

*Kunzea amathicola* × *Kunzea linearis* is mainly found in the far north of the North Island from Waipapakauri south to the Ahipara Gumlands. *Kunzea amathicola* × *Kunzea linearis* can be difficult to recognise on the gumlands because a third species, *Kunzea robusta*, is also present, and, together with *Kunzea amathicola* and *Kunzea linearis*, it has contributed to a complicated hybrid swarm around the old gum workings and roadsides. This bewildering array of hybrids was first discovered by A. P. Druce, who thought that some of the extremes represented a potentially new species, calling these “*Kunzea* Ahipara” (Druce 1993, CHR!). Further research using more discriminating molecular markers than that used for this study (de Lange 2007) is needed to determine the extent of introgression that has gone on between *Kunzea amathicola*, *Kunzea linearis* and *Kunzea robusta* on the Ahipara Plateau.

*Kunzea amathicola* × *Kunzea robusta* is less easily recognised than the other two hybrids because both parents have similar growth habits, bark types, and leaves. *Kunzea amathicola* is most likely to hybridise with *Kunzea robusta*, because they were, at least until recently, widely sympatric throughout much of the North Island range of *Kunzea amathicola*. This hybrid is best recognised by the hairs of the leaf margins which, though of varying thickness, rarely reach the leaf apex. The hairs tend to be shed from the apex to the base (a feature of *Kunzea robusta*) as the leaf matures, such that older leaves are either completely glabrous or only sparsely hairy. Leaf shape in some hybrids is distinctly narrowly oblanceolate to lanceolate, with an acute rather than obtuse to rounded apex. The pherophylls of the hybrid tend to be deciduous rather than persistent, and rather variable in size and shape, recalling the usual condition of *Kunzea robusta*. The inflorescences though elongated tend to be compact. In practice this serves as a good field character, although some specimens of *Kunzea amathicola* can have reduced inflorescences, in which case recourse to the shape and degree of persistence of the pherophylls and the leaf laminal hairs is needed. In most cases *Kunzea amathicola* can be recognised by its ecology, because *Kunzea robusta* tends to avoid the active sand and sand dune habitats it prefers. Further, in the majority of locations where *Kunzea amathicola* is present, it now occurs in complete isolation from *Kunzea robusta* as a result of habitat destruction. Nevertheless this is not the case along large parts of the Kaipara Harbour and western Waikato coastline, where both species grow together and where the habitats have, and most cases continue to be, severely disrupted. Thus it is possible that some plants collected from these areas that I have assigned to *Kunzea amathicola* may ultimately prove to be hybrids.

#### Vernacular names.

Beyond the ubiquitous ‘kanuka’ this species is known to Muriwhenua (Ngati Kuri and Te Rarawa) and Nga Puhi Maori as ‘manuka’ and ‘rawiritoa’. Rawiritoa serves to distinguish *Kunzea amathicola* from the allied ‘rawiri’ (*Kunzea linearis*) and ‘rawirinui’ (*Kunzea robusta*) (W. Murray, G. Neho, and L. Foley pers. comm.).

#### Conservation status.

*Kunzea amathicola* as Kunzea aff. ericoides (a) (AK 286081; “sand”) is appropriately listed under Appendix 2 of the New Zealand threatened and uncommon plants as ‘At Risk / Declining’ (de Lange et al. 2013b).

### 8. Kunzea triregensis

Taxon classificationPlantaeMyrtalesMyrtaceae

de Lange sp. nov.

urn:lsid:ipni.org:names:77141732-1

A K. linearis foliis lanceolatis vel anguste lanceolatis, inflorescentibus elongatis, bracteis floralibus ellipticis vel lanceolatis effusis, hypanthio late obconico vel campanulato differt. Etiam ordine rDNA ETS a K. linearis recedit.

#### Holotype

**Figure 40.**
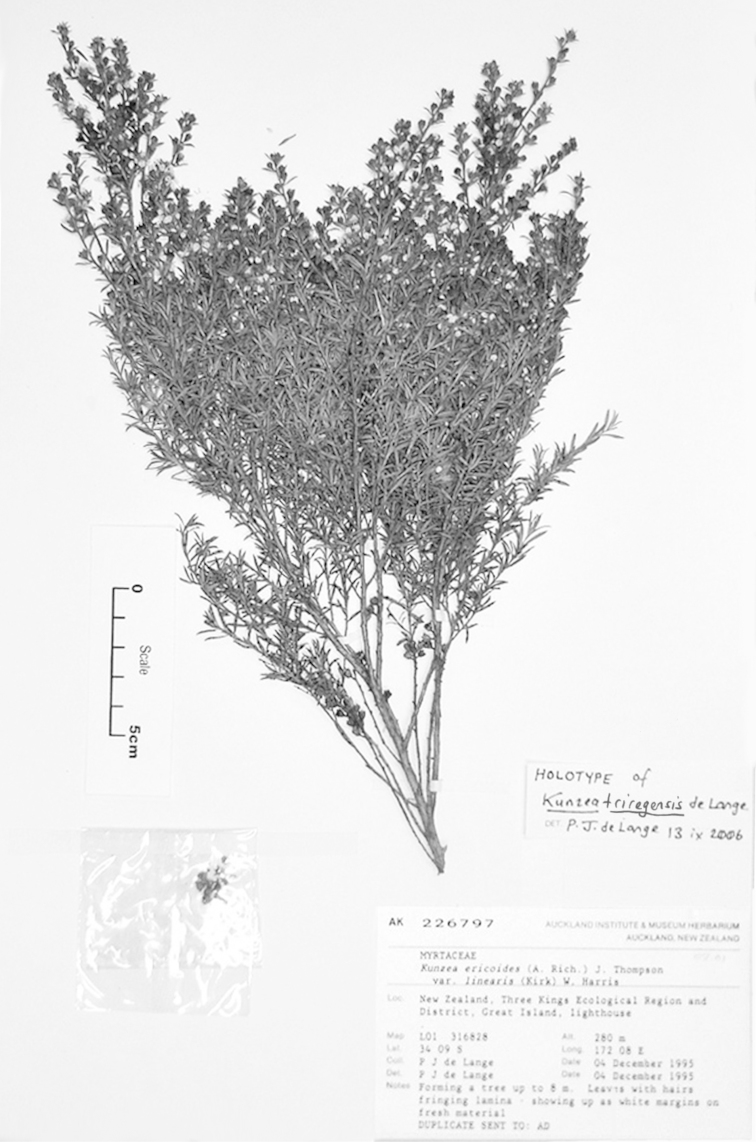
Holotype of *Kunzea triregensis* de Lange (*P. J. de Lange s.n.*, AK 226797).

**(Fig. 40).** New Zealand, Three Kings Islands group, Great Island, Lighthouse, 34°9'S, 172°8'E, 280 m a.s.l. ‘Forming a tree up to 8 m. Leaves with hairs fringing lamina, showing up as white margins on fresh material’ P. J. de Lange s.n., 4 Dec 1995, AK 226797! Isotypes. AD!

#### Etymology.

The specific epithet *triregensis* refers to this species being endemic to the Three Kings Island group. The recognition of *Kunzea triregensis* brings to 15 the number of endemic vascular plant taxa recognised for the Three Kings Island group.

#### Description

**Figure 41.**
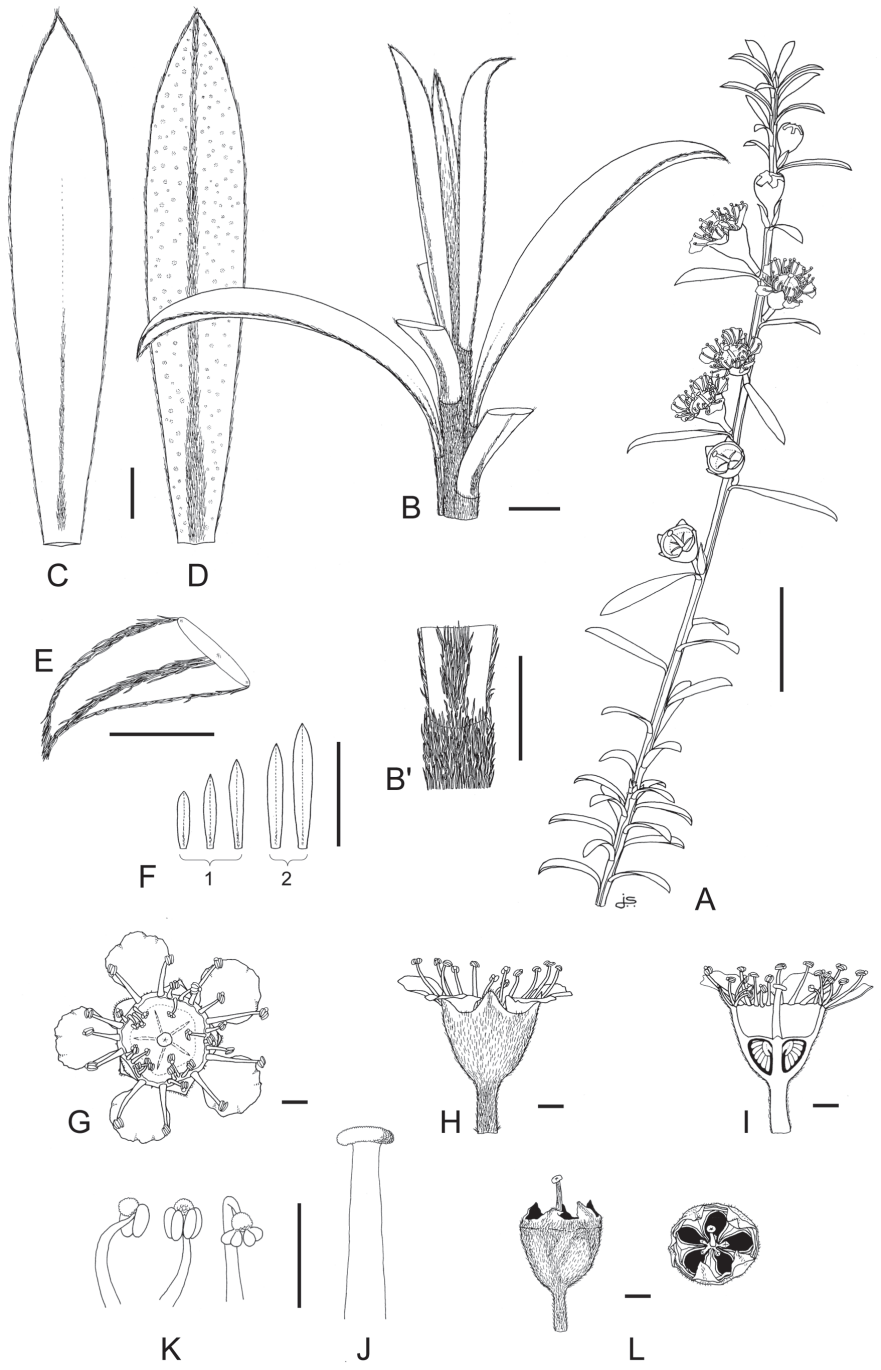
Distinguishing features of *Kunzea triregensis*. **A** Flowering branchlet (ex cult. AK 246881) **B, B1** Vegetative bud and branchlet indumentum (ex cult. AK 246881) **C** Adaxial leaf surface (AK 246881) **D** Abaxial leaf surface (ex cult. AK 246881) **E** Adaxial leaf apex and leaf margin indumentum (ex cult. AK 246881); (**F1**) Pherophylls (ex cult. AK 46881); (**F2**) Vegetative leaves (ex cult. AK 246881) **G** Flower (top view) (ex cult. AK 246881) **H** Flower and hypanthium (side view) (ex cult. AK 246881) **I** Flower cross section showing anther, style and ovules (ex cult. AK 246881) **J** Style and stigma (ex cult. AK 246881) **K** Stamens (ex cult. AK 246881) **L** Dehisced fruit (ex cult. AK 246881). Scale bars: (**A, F**) 10 mm; (**B–E, G–L**) 1 mm.

**Figure 42.**
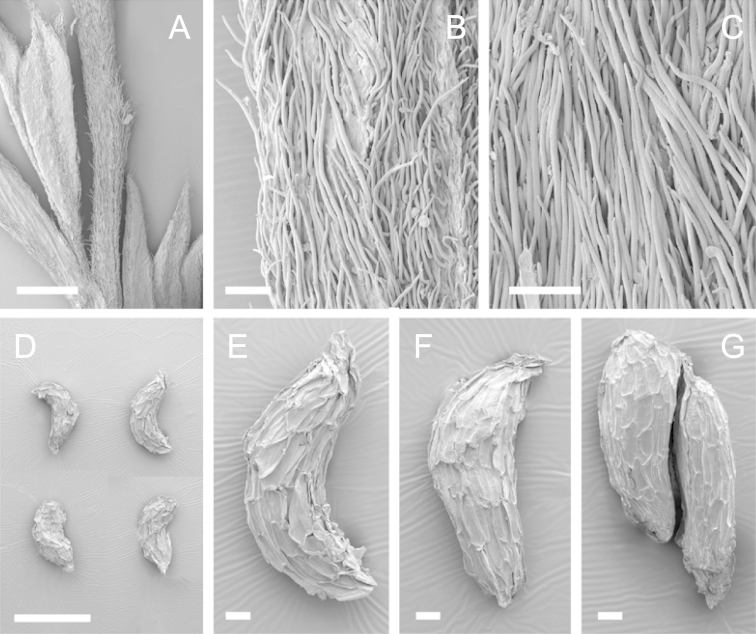
Scanning Electron Micrographs of *Kunzea triregensis*. (**A–C** all AK 207160) Branchlet indumentum **D–G** Seeds (AK 289067). Scale bars: (**A, D**) 1 mm; (**B, C, E–G**) 100 μm.

**Figure 43.**
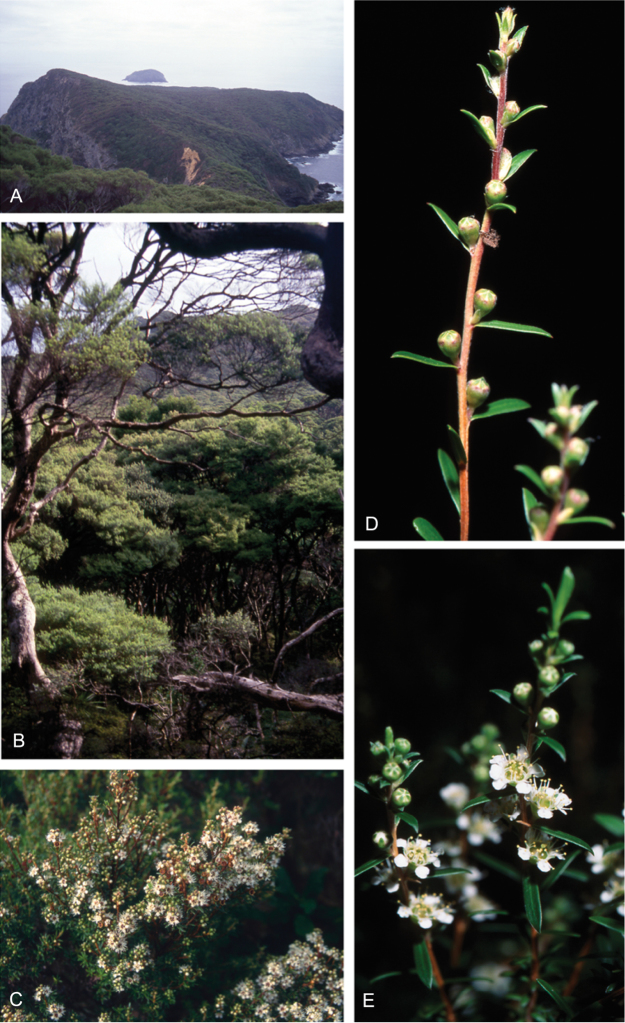
*Kunzea triregensis*. **A** *Kunzea triregensis* forest, Three Kings Islands group, Manawatawhi / Great Island looking east to North East Island (photo: *P. J. de Lange*) **B** Interior of *Kunzea triregensis* forest, Three Kings Island group, Manawatawhi / Great Island, Tasman Stream (photo: *P. J. de Lange*) **C** *Kunzea triregensis* in full flower, Three Kings, Manawatawhi / Great Island, near Lighthouse (photo: *P. J. de Lange*) **D** *Kunzea triregensis* showing elongate botryum, pherophylls and buds just prior to bud burst (photo: *J. E. Braggins*); *Kunzea triregensis* showing elongate botryum, pherophylls, buds and flowers (photo: *J. E. Braggins*).

**(Figs 41, 42, 43).** *Growth habit* mostly trees up to 18 × 3 m, forming a broadly rounded to somewhat spreading canopy with the lower 50–70% of the trunk usually completely devoid of branches. *Trunk* 1(–4), 0.10–0.60(–0.85) m d.b.h., mostly erect; basal portion of trunks covered with numerous semi-detached, long somewhat tabular lengths of rather corky-coriaceous bark. *Bark* early bark firmly coriaceous, grey or grey-brown, ± elongate, usually bearing a few transverse cracks (especially on branch flanges and decurrent leaf bases) otherwise remaining firmly attached, margins elongate sinuous, ± entire with scarcely any flaking; old bark similar though more distinctly corky-coriaceous, tessellated, firmly attached, detaching basally with age, and peeling upwards along trunk in broad, tabular strips, margins ± entire to weakly irregular; upper surface often deeply corrugated and cracked but not peeling; margins sinuous to lunate; early and old bark flakes firm, not crumbling in hand, snapping with a ± entire margin. *Branches* numerous, usually confined to the upper 30–50% of trunk; upright to somewhat spreading; branchlets numerous, slender, ± quadrangular to subterete, leaves ± evenly spaced along length; branchlets sericeous, indumentum copious; hairs long appressed, usually flexuose (220–)480(–520) μm long, hyaline to translucent (appearing white when young, maturing grey). *Vegetative buds* conspicuous; at resting stage 1.0(–2.2) mm diam., narrowly lanceolate; scales absent. *Leaves* sessile, well spaced along branchlets, spreading, patent to recurved; lamina (6.0–)10.0(–13.5) × (1.1–)1.8(–2.3) mm, dark glossy green above, paler beneath with leaf margins and midrib appearing distinctly white because of dense hair growth; lamina lanceolate to narrowly lanceolate; usually strongly recurved for about half of total length; apex acute to narrowly acute, base attenuate; adaxial surface usually deeply concave to weakly so, very rarely flat, oil glands not evident when fresh, conspicuous when dry, up to c.200; midrib slightly raised near base, otherwise not evident for rest of length, finely covered in antrorse-appressed, silky hairs in lower 50–70% otherwise glabrous; abaxial surface convex to v-shaped, glandular punctate, oil glands up to 200, more evident when dry; midrib raised for most of length, densely silky hairy to leaf apex, hairs weakly flexuose, antrorse-appressed, up to 0.8 mm long, hyaline to translucent, appearing as white to naked eye; lamina margin completely obscured by dense covering of antrorse-appressed hairs aligned in a thick, up to 0.6 mm wide, almost plumose, white band meeting at leaf apex and continuous down branchlets along decurrent leaf bases. *Perules* squamiform, ± persistent grading into pherophylls, (4.0–)8.2(–11.8) × (0.9–)1.6(–2.2) mm; dark glossy green, broadly oblong to oblanceolate, usually strongly recurved, weakly concave, oil glands not evident when fresh, conspicuous when dry, up to c.80, margins ± flat, margins and midrib densely covered in sericeous, appressed, hairs, midrib weakly keeled. *Inflorescence* an elongated (3–)10(–20)-flowered botryum up to 200 mm long, basal portion sometimes bearing compact, lateral 3-flowered corymbiform botrya, or with the basal and terminal portions occasionally bearing lateral elongate botyra; distal 70% often interrupted by sections of leafy perules between which are spaced further flowers; or interrupted by short floral shoots bearing elongated 3–6-flowered botrya up to 20 mm long; terminal portion often bearing undeveloped flowers and vegetative terminal growth. Inflorescence axis densely invested in antrorse-appressed, weakly flexuose, hairs. *Pherophylls* persistent, foliose, (6.0–)9.8(–12.8) × (0.9–)1.8(–2.2) mm, dark glossy green, elliptic, broadly lanceolate to lanceolate; strongly recurved, to about half of total length or flat; apex acute, base attenuate; adaxial surface usually deeply concave to weakly so, oil glands not evident when fresh, conspicuous when dry, up to c. 80 (usually fewer); midrib slightly raised near base, otherwise not evident for rest of length, finely covered in antrorse-appressed, silky hairs for whole length; abaxial surface deeply convex, glandular punctate, oil glands up to 100 (usually fewer), more evident when dry; midrib raised for most of length, densely covered in antrorse-appressed, silky hairs to apex, lamina margin obscured by dense covering of antrorse-appressed, silky hairs. *Pedicels* subsessile to pedicellate (0.4–)1.3(–3.7) mm long at anthesis, usually elongating slightly after anthesis, terete, copiously invested in antrorse-appressed, weakly flexuose, silky hairs. *Flower buds* double-conic to ovoid, calyx lobes prior to bud burst mostly not or scarcely meeting, held flat across bud surface, occasionally suberect with lobes ± meeting. Fresh flowers when fully expanded (6.3–)10.2(–12.3) mm diam., usually reducing in size toward end of flowering season. *Hypanthium* (1.6–)2.8(–4.4) × (2.0–)3.0(–4.6) mm, with free portion 0.6–0.8 mm long, dark green or red-green, drying green-brown or red-brown; hemispherical to broadly obconic, sometimes campanulate or rarely cupular, terminating in dark-green to red-green coriaceous rim bearing five persistent erect calyx lobes; hypanthium surface when fresh, smooth to faintly ribbed, faintly and sparingly dotted with pink or colourless oil glands, densely to sparsely covered in silky, appressed antrorse hairs; similar when dry though with the ribs more strongly defined and clearly leading up to calyx lobes. Calyx lobes 5, erect, coriaceous, (0.5–)0.9(–1.3) × (0.3–)0.5(–0.8) mm, persistent, deltoid to ovate-deltoid, green to red-green, prominently keeled, with keel usually slightly darker-coloured and densely covered in antrorse-appressed, hairs; margins pale green often flushed pink, glabrescent, surface somewhat glandular punctate, oil glands inconspicuous, ± colourless. Receptacle green at anthesis, consistently darkening to crimson after fertilisation. *Petals* 5(–6), (1.3–)2.8(–4.3) × (1.9–)2.8(–4.8) mm, white, orbicular to broadly ovate, apex rounded, margins ± finely and irregularly denticulate, often when fresh appearing to be finely folded or crimped 1–3 or more times, oil glands colourless. *Stamens* 30–46(–53) in 1(–3) weakly defined whorls, arising from receptacular rim, filaments white. Antipetalous stamens (2–)3(–5) sometimes petaloid, antisepalous stamens (3–)4(–6). Outermost antipetalous stamens incurved or weakly outcurved, on filaments 1.0–3.8 mm long, inner stamen if present, 0.9–1.8 mm, incurved, with a further 1–3 stamens, of similar length to inner stamen often present at the base of the outermost antipetalous pair. Antisepalous stamens usually shorter than outermost antipetalous stamens, but sometimes of comparable length, generally 0.9–3.8 mm, weakly to strongly incurved, rarely outcurved, usually in mixtures of both. Anthers dorsifixed, 0.05–0.10 × 0.06–0.08 mm, testicular-ellipsoid, latrorse. Pollen white (12.0–)13.8(–16.0) μm. Anther connective gland prominent, pink or golden-yellow when fresh, drying yellow to pale orange, spheroidal, finely to coarsely papillate. *Ovary* 4(–5) locular, each with 20–24(–38) ovules in two rows on each placental lobe. Style (1.9–)2.8(–3.1) mm long at anthesis, elongating after anthesis, white or pinkish-white; stigma broadly capitate, conspicuously wider than style, ± flat, greenish-white or pale pink, flushing red after anthesis, surface granular-papillate. *Fruits* long persistent, (1.9–)3.2(–5.2) × (2.0–)3.1(–4.9) mm, initially dark chestnut-brown to almost black, fading with age to grey, hemispherical, broadly obconic, campanulate to cupular; calyx valves usually prominently erect to suberect, rarely incurved, splits concealed by dried, erect, free portion of hypanthium. *Seeds* 0.50–1.00(–1.10) × 0.50–0.60(–0.80) mm, oblong, oblong-obovate, curved near apex, laterally compressed, 2–3-angled with convex to flattened faces, apex rounded to subacute, base oblique, ± flattened; testa semi-glossy, orange-brown to dark brown; surface coarsely reticulate. FL: (Oct–)Dec(–May). FT: Oct–May. Chromosome Number *n* = 11_II_, 2*n* = 22 (see de Lange and Murray 2004).

#### Representation specimens

**(30 sheets seen).** **New Zealand, Three Kings Island group.** North East Island, G. F. Buddle s.n., 31 Dec 1947, (AK 24092); Manawatawhi / Great Island, T. F. Cheeseman s.n., Nov 1889., (AK 5516); Manawatawhi / Great Island, W. R. B. Oliver s.n., 20 Feb 1939, (WELT SP029481); Manawatawhi / Great Island, North East Bay, Isthmus Summit, P. J. de Lange 1105, 16 Oct 1991, (AK 207160, 207317, Duplicates: AD, CHR); Manawatawhi / Great Island, Tasman Stream, M. J. Thorsen s.n., 8 Apr 2000, AK 289060-289063; South-West Island, G. T. S. Baylis s.n., 10 Jan 1950, (OTA 3806); West Island, P. J. de Lange 3180, 5 Dec 1996, (AK 231919, Duplicate: HO).

#### Distribution

**(Fig. 7).** Endemic. Three Kings Island group (sea level – 296 m a.s.l.).

#### Recognition.

*Kunzea triregensis* a Three Kings Island group endemic, is the only New Zealand *Kunzea* to be truly allopatric.. It is recognised here at species rank through a combination of morphological, reproductive and molecular characters (de Lange et al. 2005; de Lange 2007; Table 1). Morphologically, the distinctive elongate botrya (Fig. 41A, 42D–E) of the species is seen otherwise only in *Kunzea amathicola*, a species from which *Kunzea triregensis* differs by its rDNA ETS sequence (de Lange 2007), homophyllous growth habit, and lanceolate to narrowly lanceolate leaves (Fig. 41B–F; Table 1). The peculiar ability of the *Kunzea triregensis* inflorescence to produce, albeit infrequently, additional lateral elongate or reduced corymbiform botrya from the base and terminus of the main botryum further distinguishes it from *Kunzea amathicola*. Prior to this treatment Cheeseman (1906, 1914, 1925) and some later authors who appear to have uncritically followed him (e.g., Poole and Adams 1963; Wardle and Platt 2011) had referred *Kunzea triregensis* to the Aotea (Great Barrier Island) endemic *Kunzea sinclairii* (Kirk) W.Harris. Cheeseman’s error is difficult to understand. *Kunzea triregensis* has no close morphological or molecular affinity to *Kunzea sinclairii*, and although Cheeseman never saw *Kunzea sinclairii* in the wild he was furnished with specimens by Thomas Kirk, and indeed had it illustrated from these in Cheeseman (1914). The confusion may have arisen because on Cheeseman’s visit to Manawatawhi / Great Island, the largest island in the Three Kings Island group, he saw what he described as ‘suberect’ plants growing on the ‘declivities leading down to the cliffs’ (Cheeseman 1914). Further, because the leaf margins and abaxial midribs of *Kunzea triregensis* are copiously covered in white to silvery-white plumose hairs, and Kirk (1899; p.158) had emphasised ‘white silky hairs’ in his description of the leaves of Leptospermum (Kunzea) sinclairii, this may have influenced Cheeseman in his decision. It is also clear that Cheeseman (1914; caption facing *Leptospermum sinclairii*, pl. 47) had doubts as to the validity of *Kunzea sinclairii*, though he did conclude that Three Kings Islands plants had ‘leaves [that] were slightly narrower than in the Barrier plant’. Whatever the reason for Cheeseman’s decision, *Kunzea triregensis* differs markedly from *Kunzea sinclairii*, indeed it was referred by Oliver (1948) to *Kunzea ericoides*.

Nevertheless, to clarify any further ambiguity, some distinctions between *Kunzea triregensis* and *Kunzea sinclairii* are here offered (see also Table 1). *Kunzea triregensis* is usually a forest tree rather than a scrambling shrub. Although prostrate forms of *Kunzea triregensis* are known from the wild, cultivation has shown that these are environmentally induced, unlike the genetically fixed scrambling condition of *Kunzea sinclairii*. These two species differ markedly by their leaf colour, shape and degree of investiture (in *Kunzea triregensis* the leaves are dark green, lanceolate to narrowly lanceolate with the lamina margins and abaxial midrib copiously covered in white hairs, and the intervening lamina glabrous; in *Kunzea sinclairii* the leaves are consistently grey-green to grey, broadly lanceolate, elliptic to oblanceolate and completely hairy). Another key difference is the inflorescence. In *Kunzea triregensis* these are consistently in the form of an elongate botryum with leafy pherophylls, while *Kunzea sinclairii* has corymbiform botrya with small, deciduous pherophylls (Table 1).

*Kunzea triregensis* has also been confused with *Kunzea linearis*. *Kunzea tiregensis* differs from *Kunzea linearis* by its more openly vegetated, less densely crowded branchlets, and by the leaves which in *Kunzea triregensis* are consistently lanceolate to narrowly lanceolate rather than linear (Fig. 41C–F). Further, in *Kunzea triregensis* the thick bands of marginal and abaxial midrib hairs meet at the leaf apex, whereas in *Kunzea linearis* the marginal hairs meet just short of the adaxial face of the apex (Fig. 41E) and the abaxial midrib hairs stop short of the apex. The inflorescence of *Kunzea triregensis* is consistently elongated and the flowers are usually widely spaced, only in stressed conditions becoming more crowded (Figs 41A, 43C, E). In contrast, the inflorescence of *Kunzea linearis* is usually a condensed, densely packed spiciform botryum. However, in shade forms or late season flowering specimens of *Kunzea linearis* the inflorescences may elongate considerably, in which case distinction between *Kunzea triregensis* and *Kunzea linearis* using inflorescence type is less clear. In shade specimens, the early season inflorescences of *Kunzea linearis* will still show the typically condensed spiciform condition, and the flowers are mostly sessile to subsessile rather than the mostly pedicellate to subsessile condition of *Kunzea triregensis*. The pherophylls of both species are also diagnostic; those of *Kunzea linearis* are consistently linear while those of *Kunzea triregensis* are elliptic or broadly lanceolate to lanceolate (Fig. 41A). While the the calyx lobes of mature flower buds of *Kunzea triregensis* may sometimes be suberect and touching, and therefore resemble those of *Kunzea linearis*, the usual condition is that the calyx lobes are held flat or curved across the domed bud surface, and do not touch (see Figs 41A, 43D). Cytologically the chromosome karyotype of *Kunzea triregensis* has none of the marked size differences typical of *Kunzea linearis* (see de Lange and Murray 2004). Further, the ETS sequence of *Kunzea linearis* is particularly distinctive and that species shows no obvious relationship to *Kunzea triregensis* (see *Kunzea linearis*) (de Lange 2007). It is because of these distinctions that allopatric *Kunzea triregensis* is treated at species rank.

Five collections of *Kunzea* from the two main islands of the Poor Knights Island group, Aorangi (e.g., L. B. Moore s.n. & L. M. Cranwell (AK 102471)) and Tawhiti Rahi (e.g., B. S. Parris s.n. (AK 128064); A. E. Wright 3970 (AK 155364); A. E. Wright 11413 (AK 201664); E. K. Cameron 10274 (AK 252512)), are morphologically similar to *Kunzea triregensis*. The Tawhiti Rahi specimens differ from *Kunzea triregensis* mainly by their extremely linear leaves which are densely crowded along the branchlets, and which range from being rather hairy to almost glabrous. Otherwise the plants have elongate botrya similar to those of *Kunzea triregensis*. However, the fruits of these specimens are mostly barrel-shaped to cupular and vary from glabrate to distinctly hairy. These are features of *Kunzea linearis*, from which they differ by their shorter (up to 8 mm long) mostly spreading rather than ascending leaves, and shortly pedicellate rather than sessile fruits. The sole gathering from Aorangi is even more like *Kunzea triregensis* in that it has much broader lanceolate leaves but the fruits differ in that they are glabrate, up to 5 × 5 mm, mostly barrel-shaped (with a very few broadly obconic), and more or less consistently long-pedicellate. As both *Kunzea linearis* and *Kunzea robusta* have been collected from the Poor Knights Island group, these five gatherings are most likely examples of an introgressed hybrid swarm involving these two species. The distinctly linear-leaved Tawhiti Rahi plants are closer to *Kunzea linearis* than *Kunzea robusta*, while the Aorangi specimen is closer to *Kunzea robusta*. Seed that I have germinated from Aorangi Island (Poor Knights Island group) examples of these plants suggested they are also hybrids, as the seedlings showed clear segregation to both the postulated parents. It is plants such as these that appear to be the basis for the erroneous statement by Hynes (1950) that *Kunzea sinclairii* was found on the Poor Knights Islands (see de Lange and Cameron 1999; p. 464).

The DNA sequence data placed *Kunzea triregensis* next to *Kunzea robusta* (de Lange 2007), from which it differs only by the presence of an indel within the ETS sequence at alignment position 269 (Table 2). The same indel is universal to all the Australian members of the *Kunzea ericoides* complex but was found in New Zealand otherwise only in a single sample from a *Kunzea* of uncertain status sampled from Lottin Point, East Cape (de Lange 2007; Table 2).

#### Ecology.

*Kunzea triregensis* is the dominant woody tree on the Three Kings Island group where it occurs from near sea level to the summits of North East, Manawatawhi / Great Island (Fig. 43A, B) and West Islands. On South West Island it seems to be naturally uncommon. The flora of Manawatahi / Great Island is an assemblage of what survived after at least 60 years of intensive goat (*Capra aegagrus hircus* (Linnaeus, 1758)) browse (Baylis 1948, 1951; Oliver 1948), thus it retains few truly tree-forming species, as these were mostly extirpated. Of the few that still exist, most have fruits that depend on large birds for dispersal, and, as these are now absent from the Three Kings, such trees remain trapped in places where the goats could not reach (mostly cliff refugia), and so are unable to spread. For this reason, following the eradication of goats from the island, *Kunzea triregensis*, which would more usually be a short-lived successional forest species, has formed what is probably a self-sustaining forest type (Fig. 43B).

As a result, *Kunzea triregensis* is the dominant tree on Manawatawhi / Great Island, being scarce or absent only from steep, sparsely vegetated coastal cliffs, and boulder beaches. It is also common on North-East Island, though there it is being slowly replaced by *Meryta sinclairii* (Hook.f.) Seem. forest. On the exposed wind shorn cliff tops of Manawatawhi / Great Island, *Kunzea triregensis* presents often as environmentally induced decumbent to semi-erect, widely spreading bushy shrubs

On Manawatawhi / Great Island, *Kunzea triregensis* has been recorded as the host species for the threatened polypore fungus *Dichomitus newhookii* P.K.Buchanan et Ryvarden (McKenzie et al. 2006). The bark of the mature trees also provides a refuge for an unnamed gecko (*Woodworthia* “Three Kings”) endemic to the Three Kings Island group (R. Hitchmough pers. comm.).

#### Hybridism.

*Kunzea triregensis*, being allopatric from the other New Zealand members of the *Kunzea ericoides* group does not naturally form hybrids. However, experimental hybrids were readily produced using *Kunzea triregensis* as pistillate or staminate parent (de Lange et al. 2005, as Kunzea aff. ericoides (e)).

Although the hybrid *Kunzea amathicola* × *Kunzea linearis* was not synthesised (de Lange et al. 2005), putative wild hybrids show a great similarity to *Kunzea triregensis*, suggesting that *Kunzea triregensis* may be a stable hybrid between these two species. Future research into the possible past hybrid origin of *Kunzea triregensis*, including the synthesis of *Kunzea amathicola* × *Kunzea linearis* would be worthwhile.

#### Vernacular name.

*Kunzea triregensis* appears to have no specific Maori name.

#### Conservation status.

*Kunzea triregensis* as Kunzea aff. ericoides (e) (AK 226797; Three Kings) is appropriately listed by de Lange et al. (2013b) as ‘At Risk/Naturally Uncommon’ qualified ‘IE’ (Island Endemic) and ‘OL’ (One Location) because the species is confined to one island group.

### 9. Kunzea sinclairii

Taxon classificationPlantaeMyrtalesMyrtaceae

(Kirk) W.Harris

Leptospermum sinclairii Kirk in *Stud. Fl. N.Z.*, (1899), 158

#### Lectotype

**Figure 44.**
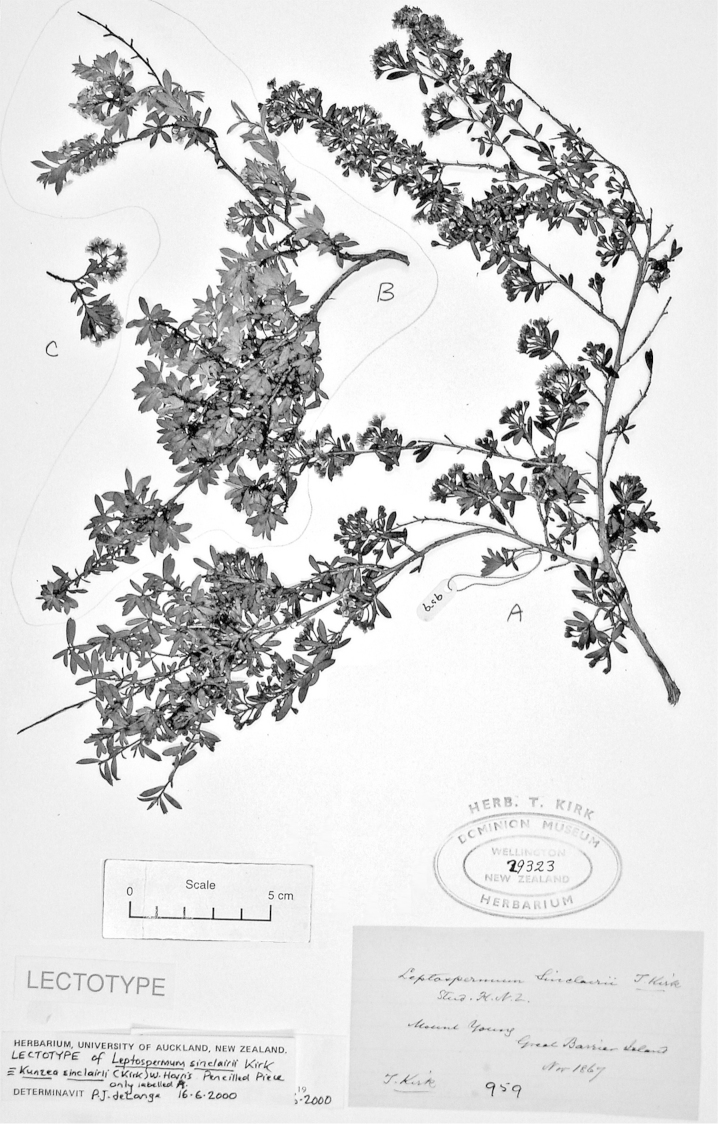
Lectotype of *Leptospermum sinclairii* Kirk (*T. Kirk 959*, WELT SP029323 (piece labelled in pencil “A”).

**(here designated)** **(Fig. 44).** *Leptospermum sinclairii* T.Kirk, *Stud. Fl. N.Z.* Mount Young, T. Kirk 959, 20/11/67, WELT SP029323! (piece labelled ‘A’)

Paralectotypes (here designated). WELT SP029323! (piece labelled ‘B’), WELT SP029323C! (piece labelled ‘C’), WELT SP29321!, WELT SP29322!

#### Notes.

**Figure 45.**
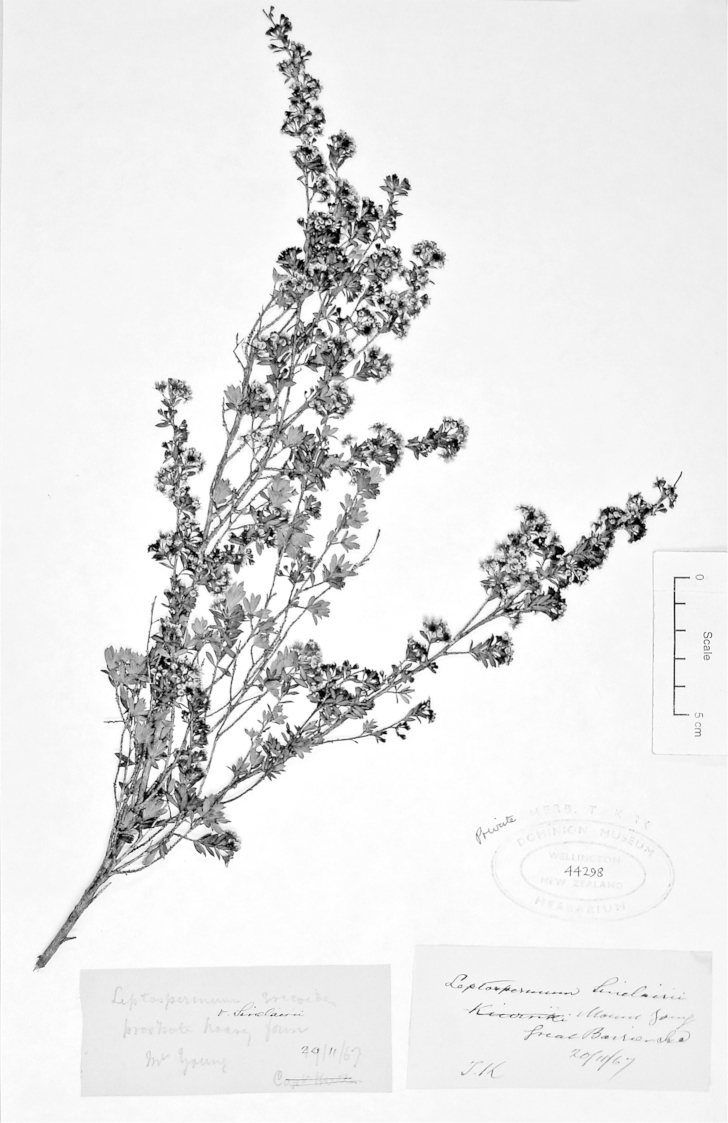
*Kunzea sinclairii* specimen from the private T. Kirk Herbarium (WELT SP044298) bearing two conflicting labels, one by Hutton and the other by Kirk.

Kirk visited Aotea (Great Barrier Island) between 15 November and 19 December 1867 (Hamlin 1965) during which time he visited a number of locations where *Kunzea sinclairii* (≡ *Leptospermum sinclairii*) is known to occur. In his protologue (1899; p. 158) he gave a brief description, attributing its original discovery to Dr Andrew Sinclair, and noted its distribution as ‘NORTH Island [*sic*]: Great Barrier Island, *Hutton* and *Kirk*. Sea Level to 1,800 ft’ but he did not specify locations or specimens. Allan (1961; p. 323) typified the name by his statement ‘Type: W[ELT], Herb. Kirk, *Hutton* and *Kirk*’. This action constitutes lectotypification under Article 9 of the International Code of Nomenclature (McNeill et al. 2012). However, there are no specimens in WELT collected jointly by Hutton and Kirk, but there are five specimens lodged there, and labelled by Kirk as *Leptospermum sinclairii*. Four of these were collected by Kirk and one possibly by Hutton (Fig. 45). Therefore I suggest that Allan’s typification was based either on a literal interpretation that Kirk (1899; p. 158) was citing a jointly collected specimen or that he was repeating Kirk’s protologue and indicating where type material might be found (which accords with remarks on typification made in the introduction to Allan 1961). Either way, Kirk’s statement may also have meant that he and Hutton collected specimens, but not necessarily together. Because of this uncertainty and because Allan did not specify which of the five specimens in WELT is the Lectotype, I regard his lectotypification as incomplete.

A major problem in typifying Kirk taxa named in the Students’ Flora is that this publication, was published some six years after Kirk’s death and is incomplete, comprising mostly those sections regarded as finished by an unknown individual or individuals tasked with assembling Kirk’s Students’ Flora for the then Education Department, Wellington, New Zealand in 1899 (see ‘Introductory Note’ in Kirk 1899). This has meant that typification of taxa published within the Students’ Flora requires thorough examination of the exact wording used by Kirk in each instance, in conjunction with the specimens he used (in this regard see also comments with respect to the typification of *Lepidium oleraceum* varieties established by Kirk (1899) in de Lange et al. (2013a)). Although Kirk had no type concept for his New Zealand Flora treatment, he was obviously aware of the need to indicate which specimens were representative of the taxa he was naming or treating for his flora. From critical study of his herbarium material, it seems that for his Student Flora of New Zealand, Kirk showed this by annotating those specimens he had finished working with ‘Stud. Fl. N.Z.’ (Fig. 44), and, as a further measure, somewhat unorthodoxly discarding the original label details, an action which often included effacing the actual collector’s name in whose place he usually wrote his own (see comments by de Lange and Gardner 2002; de Lange et al. 2013a). Therefore, I have used this pattern of labelling as an indication of his intent when naming new taxa.

**Figure 46.**
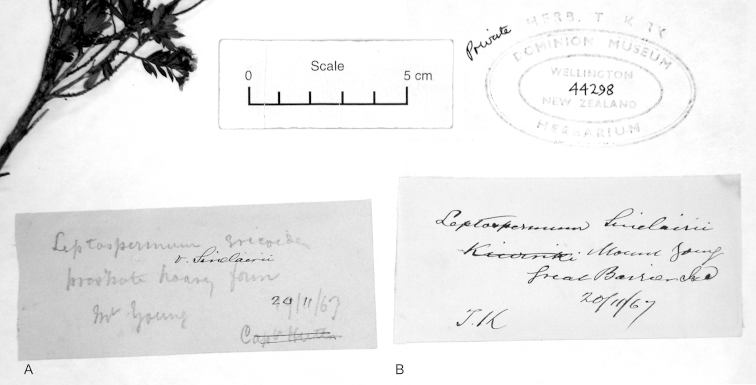
Details of labels on herbarium sheet WELT SP044298. **A** Pencil label on blue paper in handwriting of Captain F. W. Hutton bearing annotations in Indian ink by T. Kirk—annotations comprise the manuscript name ‘v. Sinclairii’, a change of collection date and the crossing out of Hutton’s name as collector **B** Second label on herbarium paper bearing the handwriting of T. Kirk in Indian ink. Neither label can be matched with certainty to the specimen mounted on WELT SP044298.

Of the five specimens labelled by Kirk as “*Leptospermum sinclairii*”, and gathered from Mt Young, only three, WELT SP029321 (T. Kirk 960), WELT SP029322 (T. Kirk 958) and WELT SP029323 (T. Kirk 959), are clearly annotated ‘*Leptospermum sinclairii* T.Kirk Stud. Fl. N.Z.’. On each sheet there are several pieces, some of sufficient morphological difference to have come from different plants. In all cases one piece on each sheet has a small jeweller’s label attached to it bearing a collection number written by Kirk that matches the number on his specimen labels. WELT SP044298 from the ‘Private Herb. Kirk’ is the only specimen to bear a label in what seems to be Captain F. W. Hutton’s handwriting (Figs 45, 46A–B). Hutton’s assumed label is on blue paper and is mounted on the left hand side of the sheet. It is written in pencil and reads ‘*Leptospermum ericoides* prostrate hoary form M^t^ Young 19/11/67 Capt^n^ Hutton’. Kirk has annotated the label in Indian ink, with ‘v. Sinclairii’, changed the date to ‘20/11/67’ and crossed out Hutton’s name (Fig. 46A). A second label (Fig. 46B) written by Kirk in Indian ink on the paper he usually used, is mounted to the right of Hutton’s label and reads ‘*Leptospermum Sinclairii* Kiwiriki, Mount Young, Great Barrier Isd T. K. 20/11/67'The name ‘Kiwiriki’ though legible, is struck out, and ‘Mount Young’, the date and Kirk’s initials have been added at a later date as is evident by the lighter coloured ink. The final specimen, WELT SP044299, also from the ‘Private Herb. Kirk’ is labelled by Kirk ‘*Leptospermum Sinclairii* Great Barrier Island 20/11/67’. Significantly all five sheets are mounted on paper different from that on which the original Kirk labels were written. This probably happened when what has come to be called the ‘Kirk Herbarium’, and which was originally held loosely in newspapers (N. M. Adams and F. Pitt pers. comm.) was eventually mounted, a process that was undertaken at various times from the 1930s onwards, and often by summer students employed for the task (J. E. Braggins pers. comm.). Thus it is possible that some specimen labels may not necessarily match the associated plant specimens. This is a major problem when undertaking typifications. It could also explain the different labels on the specimen I have attributed to Hutton, but is something I have been unable to resolve.

Under these circumstances, and in consideration of all the available evidence, I designate one element of WELT SP029323 (T. Kirk 959) the lectotype of *Leptospermum sinclairii* Kirk (Fig. 44). WELT SP029323 matches Kirk protologue as to location, condition and collector, is one of three gatherings labelled by Kirk in his hand ‘*Leptospermum sinclairii* T.Kirk’ and significantly it is also annotated by him ‘Stud. Fl. N.Z.’ showing in my opinion (see above) that Kirk considered it representative of his new species for his publication. Because the specimen comprises three pieces (which I have labelled in pencil A, B, and C) and due to the history of the curation of the ‘Kirk Herbarium’, which was received, largely uncurated in several consignments dating from Thomas Kirk’s death in 1898 until the 1930s (N. M. Adams and F. Pitt pers. comm.) I cannot be sure if these three pieces came from the same plant or even the same gathering. I also harbour grave doubts over the validity of the claim that those Kirk specimens currently held in WELT constitute the ‘Kirk Herbarium’ as Kirk’s collections, including a great many specimens that may be considered as types occur throughout the world’s herbaria, and it was Kirk’s practice as a private collector (in modern terms a ‘botanical consultant’) to carry his herbarium with him from which he routinely parcelled up and gifted (or traded) examples of his new species (effectively type material) to colleagues and herbaria throughout the world. Therefore, one can never sure if Kirk collections held at WELT are truly the syntypes on which he founded his names, or even parts thereof. Ideally, typification of Kirk names requires the reassembling of his specimens which are usually scattered worldwide—a difficult prospect indeed. Thus, for all these reasons, I conservatively designate the piece labelled ‘A’, and bearing a small jeweller’s label numbered ‘959’ in Kirk’s hand, as the lectotype because that number is cross-referenced to Kirk’s specimen label mounted on the same sheet. The other two pieces I designate paralectotypes because they are part of the type collection but because of the way in which the Kirk herbarium was curated, their relationship to the lectotype is unclear. Similarly WELT SP029322 (T. Kirk 958), WELT SP029321 (T. Kirk 960) are designated paralectotypes because they are part of the type collection matching the protologue as to collector and/or they show the naming author’s intent with regard to publication of the name. WELT SP044299, although labelled by Kirk has no reference to his place of publication (Stud. Fl. N.Z.). Therefore I exclude it from this typification. Further, because of the uncertainty over the labels, I also exclude WELT SP044298 (Fig. 45) from this typification because I cannot be sure that Hutton’s adulterated label or even, for that matter, Kirk’s label are related to the same gathering, and, as Hutton’s label carries Kirk’s manuscript name ‘v. Sinclairii’ (Fig. 46A) and Kirk’s label is not annotated ‘Stud. Fl. N.Z.’, I believe Kirk did not intend this specimen to be ‘representative’ of his new species. WELT SP029316 in ‘Herb. D. Petrie’ also deserves mention. This collection comprises a small flowering piece of *Kunzea sinclairii* and bears an ‘Herb. T. F. Cheeseman’ label which is written on by both Cheeseman and Petrie. Cheeseman’s Indian ink label reads ‘*Leptospermum Sinclairii* T. Kirk, Great Barrier Island, T. Kirk’. To this Petrie has annotated the top of the label in blue ink ‘the Dominion (the word ‘Dominion’ is struck out but legible) Petrie Herbarium’ and at the bottom he has written ‘Dominion Museum, Wellington’. Attached to the stem of the specimen is a small paper slip also labelled by Petrie in blue ink ‘Type T. Kirk’. As there is nothing to associate this specimen with the original Kirk type collections I regard WELT SP029316 as having no nomenclatural status.

#### Etymology.

The specific epithet *sinclairii* honours Dr Andrew Sinclair (1794–1861) who, according to Kirk (1869, 1899), first discovered the species during a brief visit to the island. I have been unable to determine how Kirk came by that information, or when Sinclair’s visit to Aotea (Great Barrier Island) happened, or why such an assiduous plant collector as Sinclair, so prominent in the early annals of early New Zealand botany (Hooker 1867; Cheeseman 1906, 1925), failed to procure specimens.

= Leptospermum ericoides var. pubescens Kirk in *T.P.N.Z.I.* 1 (1869), 146–147.

#### Lectotype

**Figure 47.**
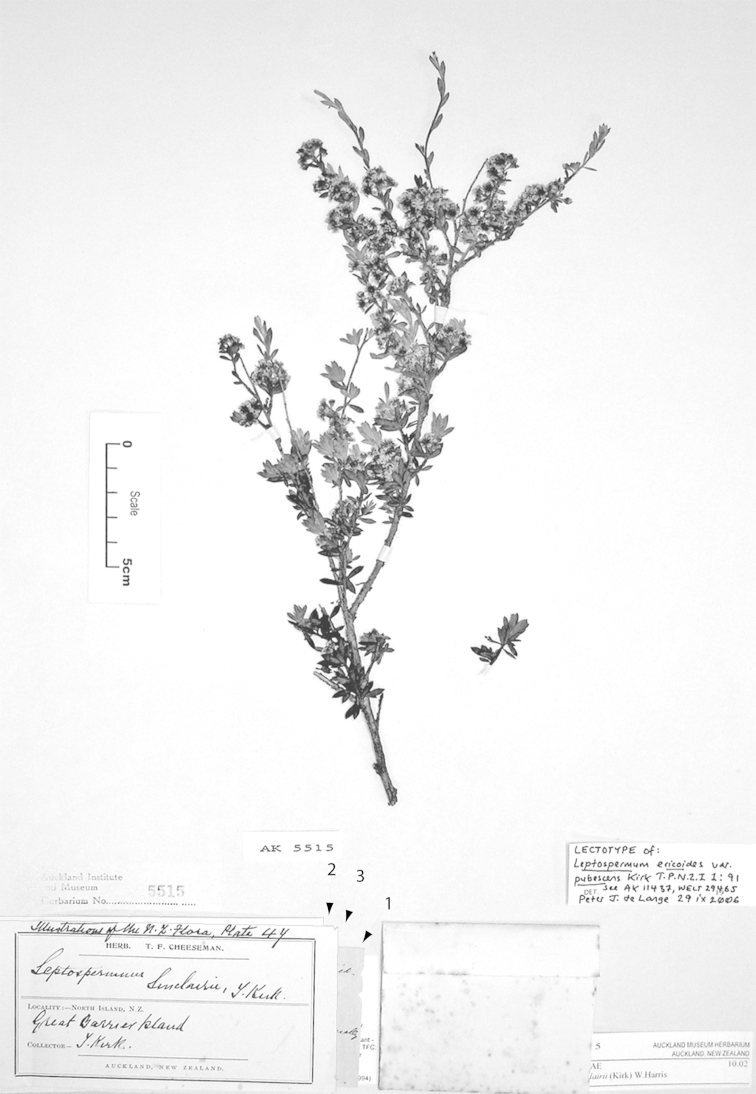
Lectotype of Leptospermum ericoides var. pubescens Kirk (*T. Kirk s.n.*, AK 5515).

**(here designated)** **(Fig. 47).** ‘Leptospermum ericoides A.Rich. v. pubescens - Great Barrier Island. T.K[irk]. Sometimes 3ft high - but usually prostrate, sometimes closely appressed to the rocks. Flowers fragrant in immense profusion’. AK 5515! Paralectotypes (here designated). AK 11437!, K (T. Kirk 176)!, WELT SP029465!

#### Notes.

**Figure 48.**
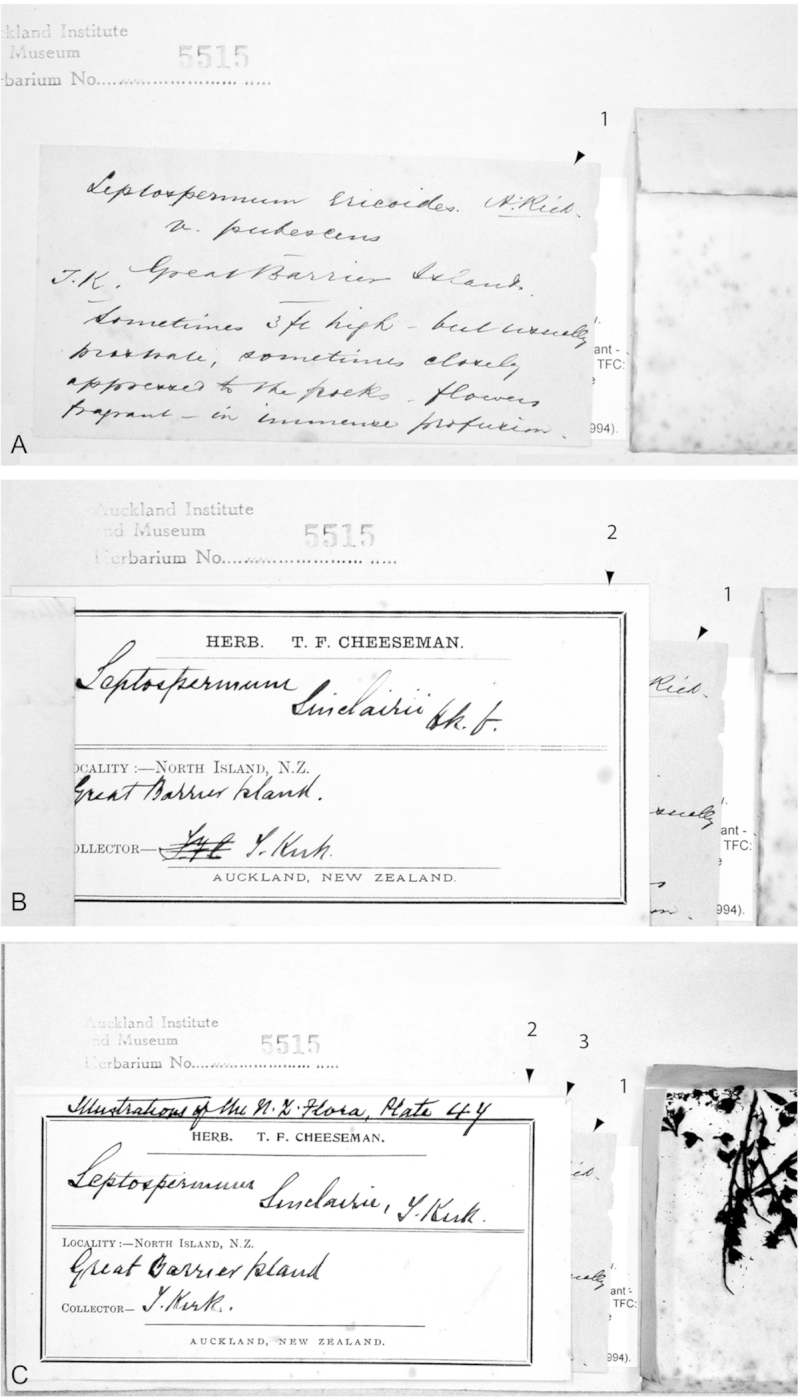
Label details of the lectotype of Leptospermum ericoides var. pubescens Kirk (AK 5515). **A** Bottom-most label written by T. Kirk and including critical details from the protologue of var. pubescens **B** Second label from bottom in handwriting of T. Cheeseman **C** Top most label recording use of specimen AK 5515 for Cheeseman’s Illustrations of the New Zealand Flora (Cheeseman 1914). Arrows indicate position of the preceding labels.

In his paper on the botany of Aotea (Great Barrier Island), Kirk (1869, pp. 146–147) published a brief description of Leptospermum ericoides var. pubescens which is given here in full. ‘Leptospermum ericoides A. Rich. var. pubescens – A prostrate or sub-erect shrub, sometimes 3 feet high, at others appressed to the rock, like an alpine plant; leaves more or less pubescent and ciliate; flowers fragrant, produced in immense profusion, sometimes concealing the leaves; pedicels and calyx downy. This would make a valuable bedding plant for the culturist. It was originally observed on the island by the late Dr Sinclair, but I am not aware of its occurrence elsewhere’. Later in the same publication Kirk provides a list of the vascular plants he saw on the island, in which he records it as ‘*Leptospermum ericoides* var.’ (Kirk 1869; p. 150). Although no specimens were cited or a location given, Kirk’s description is both valid and effective under the terms and conditions of the International Code of Nomenclature (McNeill et al. 2012). There are at least three herbarium specimens that are annotated by Kirk ‘Leptospermum ericoides var. pubescens’, WELT SP029465!, AK 5515!, and AK 11437! These I regard as part of his type collections. A further gathering held at K! (T. Kirk 176) is also labelled ‘Leptospermum ericoides var. pubescens’, though not in Kirk’s hand although it is clear it was sent by Kirk to Kew as a gift because it carries the same Kirk collection number (176) as AK 11437. I therefore regard it also as part of his original type collection. Indeed it is also the only clear duplicate among these specimens. One of the AK specimens, T. Kirk s.n. AK 5515 (Fig. 47), was used as the basis for the illustration of *Leptospermum sinclairii* by Kew botanical illustrator Matilda Smith in Cheeseman (1914, pl. 47). This sheet carries three labels; the first is by Kirk which reads ‘Leptospermum ericoides A.Rich. v. pubescens - Great Barrier Island. T.K. Sometimes 3ft high - but usually prostrate, sometimes closely appressed to the rocks. Flowers fragrant in immense profusion’ (Fig. 48A). The second (Fig. 48B), written by Cheeseman, states ‘*Leptospermum sinclairii* Hk.f. Great Barrier Island’, and the third, also in Cheeseman’s hand (Fig. 48C), records its use for his illustrated flora (Cheeseman 1914) thus: ‘Illustrations of the N.Z. Flora, Plate 44 *Leptospermum sinclairii* T. Kirk. Great Barrier Island, T. Kirk’ (the image ultimately became Plate 47). I designate this sheet Lectotype of Leptospermum ericoides var. pubescens Kirk because Kirk’s label details match his protologue (Kirk 1869; P. 146–147) more closely than the other specimens available, with respect to plant height (‘3 feet’), its growth habit (‘appressed to rocks’), flower scent (‘fragrant’) and abundance (‘in immense profusion’). Although some may feel it is desirable to select a specimen from the naming author’s herbarium, Kirk at the time of his death (8 March 1898) had no official herbarium (Brown 1968). Further, as I have noted above, the subsequent placement of the majority of his collections in WELT as the ‘Kirk Herbarium’ does not by default indicate that this is, therefore, *the* Kirk herbarium. The ‘Kirk Herbarium’ at WELT came about partly because of the circumstances surrounding Kirk’s death during the preparation of the Student Flora of New Zealand (see Introductory Note, Kirk (1899)) and the later desire of his descendants to gift what they had retained of their father’s herbarium (distinguished at WELT as the ‘Private Herb. T. Kirk’) to the place where he was mostly working at the time of his death, WELT (Moore 1973; F. Pitt and J. Fox pers. comm.).

The remaining specimens (AK 11437, K (T. Kirk 176), WELT SP29465) I designate Paralectotypes. This is because, although part of the type collection, I cannot determine what date they were collected, or even whether they came from the same plant.

#### Etymology.

The varietal epithet *pubescens*, though not elaborated on by Kirk (1869), probably refers to leaves, which he described in his paper as ‘more or less pubescent’.

#### Description

**Figure 49.**
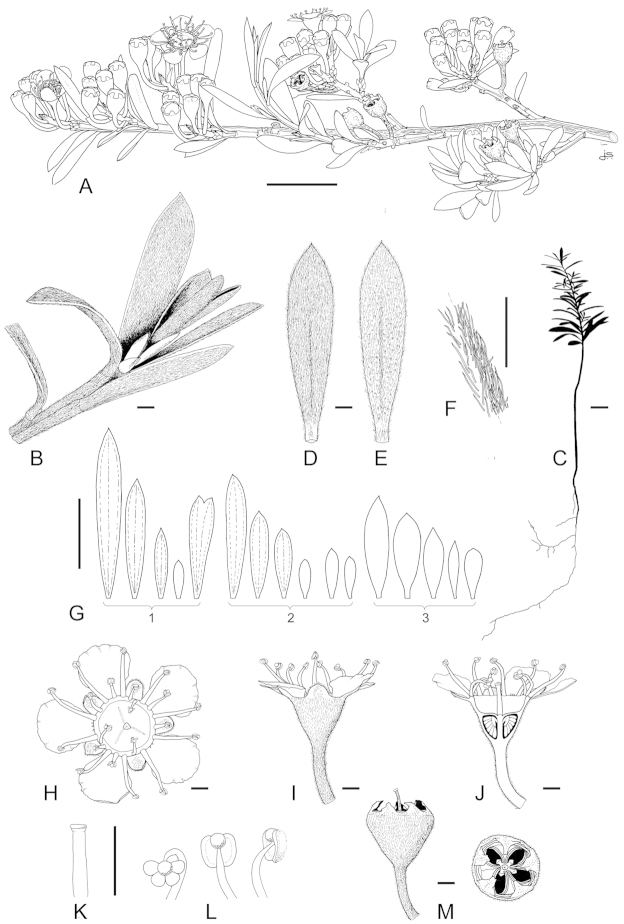
Distinguishing features of *Kunzea sinclairii*. **A** Flowering branchlets (ex cult. AK 246813) **B** Vegetative bud, leaf and branchlet indumentum (ex cult. AK 246813) **C** Three year old seedling (no voucher, ex cult. Aotea (Great Barrier Island), Mt Young) **D** Adaxial leaf surface (ex cult. AK 246813) **E** Abaxial leaf surface (ex cult. AK 246813) **F** Leaf margin indumentum (ex cult. AK 246813) **G** Leaf variation from seedling to adult (taken from (**C**) above): (**G1**) glabrous leaves of seedling (first year of growth), (**G2**) second year transitional leaves, first three w, next three hairy; (**G3**) third year adult leaves (no voucher, ex cult. Aotea (Great Barrier Island), Mt Young) **H** Flower (top view) (ex cult. AK 246813) **I** Flower and hypanthium (side view) (ex cult. AK 246813) **J** Flower cross section showing anther, style and ovules (ex cult. AK 246813) **K** Style and stigma (ex cult. AK 246813) **L** Stamens (ex cult. AK 246813) **M** Dehisced fruit (ex cult. AK 246813). Scales bars: (**A, C, G**) 10 mm; (**D, D, E, H–M**) 1 mm; (F) 0.5 mm.

**Figure 50.**
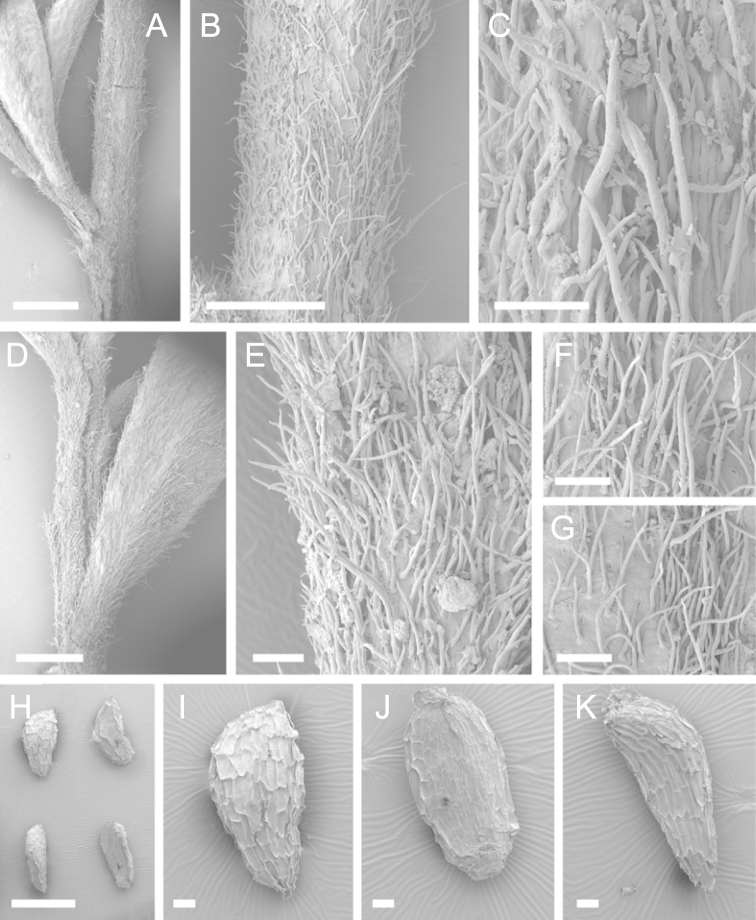
Scanning Electron Micrographs of *Kunzea sinclairii*. (**A–G** all AK 140485) Branchlet indumentum **H–K** Seeds (AK 278809). Scale bars: (**A, D, H**) 1 mm; (**B**) 500 μm; (**E–G, I–K**) 100 μm.

**Figure 51.**
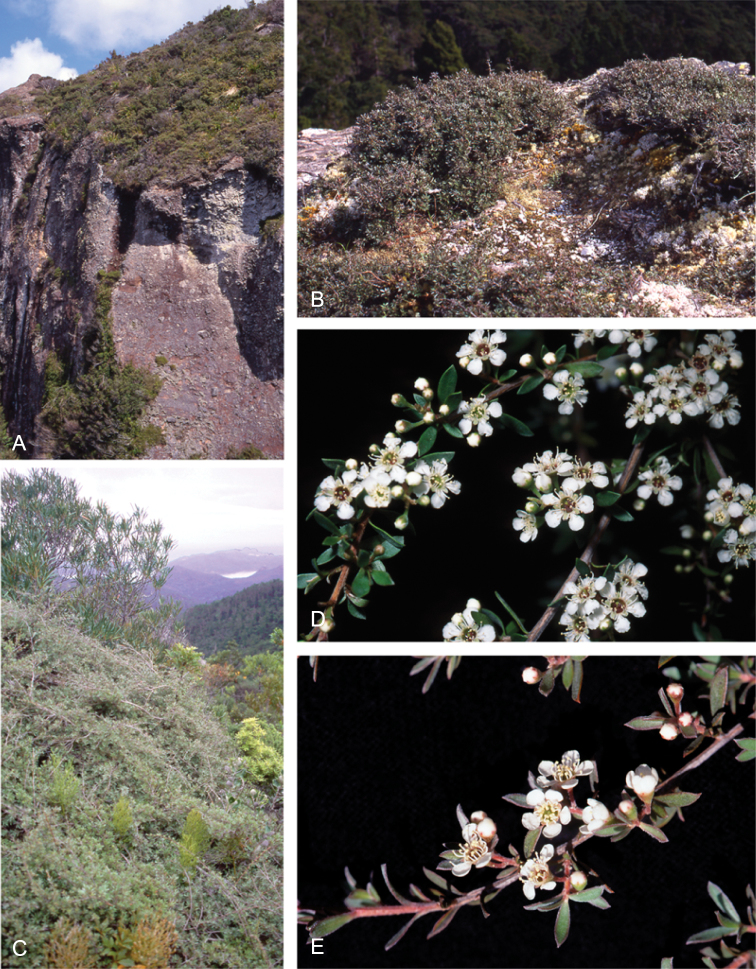
*Kunzea sinclairii*. **A** Rhyolite rock canyons on Aotea (Great Barrier Island), providing one of the key habitats for *Kunzea sinclairii* (which is the dominant shrub in the image), Aotea (Great Barrier Island), Windy Canyon (photo: *P. J. de Lange*) **B** Decumbent *Kunzea sinclairii* shrubs on rhyolitic saprolite at the type locality for the species, Aotea (Great Barrier Island), Mt Young (photo: *P. J. de Lange*) **C** Typical long trailing form of *Kunzea sinclairii* cascading down Rhyolite cliffs; Aotea (Great Barrier Island), near Mt Young **D** *Kunzea sinclairii* in full flower, ex cult. Aotea (Great Barrier Island), Mt Young (photo: *J. E. Braggins*) **E** *Kunzea sinclairii* freshly opened flowers, flower buds, and bud just prior to bud burst, Aotea (Great Barrier Island), Mt Heale (photo: *G. M. Crowcroft*).

**(Figs 49, 50, 51).** *Growth habit* mostly decumbent, trailing, silvery grey to grey, reddish-grey or grey-green, shrubs up to 3 × 1 m, very rarely forming a small tree up to 6 m tall; irrespective of stature, branches widely spreading and densely leafy, sometimes rooting on contact with soil or rock. *Trunk* 1(–4 or more), usually shortly erect between 0.2–1.0 m tall before branching but sometimes indistinguishable due to branches arising at ground level, 0.05–0.12(–0.16) m d.b.h.; basal portion of trunks covered with layers of somewhat firm to loose, stringy, pale grey to light brown chartaceous bark. *Bark* early bark dark brown to brown; firmly stringy, chartaceous to subcoriaceous, ± elongate, lying in numerous overlapping strips; usually bearing a few transverse and many longitudinal cracks (especially on branch flanges and decurrent leaf bases), otherwise firmly attached; margins elongate, sinuous, ± entire with scarcely any flaking; old bark initially dark brown to grey-brown, soon becoming covered in crustose lichens and sparse liverwort growth; coarsely stringy to tessellated and distinctly corky-coriaceous, usually remaining firmly attached, if detaching, then usually doing so along transverse cracks; flakes usually centrally attached, margins somewhat tabular with entire margins and coarsely frayed apices; upper surface of bark flakes coarsely tessellated, sometimes flaking secondarily as small tabular shards; upper trunk bark breaking into smaller pieces in hand but not crumbling. *Branches* numerous, usually present from close to or at trunk base, prostrate and widely spreading, new growth subscandent (in rare tree forms this habit is retained resulting in arching, pendulous branches); branchlets numerous, widely spreading to subscandent, often coarsely interwoven, initially red, ± quadrangular to subterete, leaves usually densely crowded along stems and brachyblasts, though in vigorous new growth sometimes widely spaced; branchlets sericeous, indumentum copious, silky, hairs antrorse-appressed, weakly flexuose up to 0.06 mm long, hyaline to translucent (appearing silvery-white when young, maturing silver-grey). *Vegetative buds* inconspicuous, usually obscured from view by surrounding leaves; at resting stage 0.3–0.8 mm diam. narrowly ovoid to ellipsoid; scales deciduous; (0.3–)1.2 mm long, pale yellow-brown to reddish brown, broadly to narrowly ovate-lanceolate grading through lanceolate to narrowly lanceolate; midrib ± keeled, sometimes prolonged to apiculate tip, otherwise apex obtuse to subacute or acute; oil glands inconspicuous, sparse, scattered in irregular lines either side of midrib; lamina initially completely invested by long silky silvery-white hairs, becoming glabrate, with hairs progressively confined to scale margins, midrib, and keel prolongation (if present). *Leaves* heterophyllous, weakly to strongly spicy-scented when crushed, mostly sessile, sometimes shortly petiolate (up to 1.6 mm long). Seedling and juvenile leaves dark green to glaucous, glabrous up to 25.0 × 3.5 mm, oblanceolate to lanceolate, apex acute, often shortly mucronate, base attenuate, lateral veins evident, especially on seedling leaves, both surfaces distinctly gland-dotted, oil glands up to 480 on either surface. Mature leaves soon developing (depending on degree of exposure), densely crowded along branchlets and brachyblasts, particularly toward apices, initially obliquely ascending, soon suberect to widely spreading usually weakly recurved in distal 30%; lamina (5.6–)14.5(–20.6) × (2.0–)3.2(–4.5) mm, initially appearing silvery-white (due to dense hair covering), maturing silvery-grey to reddish grey (as some hairs are shed), usually paler beneath; lamina broadly lanceolate, elliptic to obovate, rarely oblong-obovate, apex sharply acute, often cuspidate, base attenuate; adaxial lamina surface flat to weakly concave, glandular punctate, with oil glands scarcely evident when fresh due to dense hair covering, becoming more obvious in old leaves and in dried specimens, up to c.380, midrib slightly raised for c. 70% of leaf length; abaxial surface flat to weakly convex, usually densely covered in hairs, sometimes glabrate in old leaves, glandular punctate, oil glands up to 300; midrib raised for entire length; lamina margins distinctly less hairy than lamina surface; hairs of midribs and margins converging at leaf apex. *Perules* deciduous (shedding very early in inflorescence maturation), (0.8–)1.2(–1.4) × (0.8–)1.0(–1.2) mm, orange brown to amber with a broad pale brown margin (this reducing in thickness toward apex); broadly ovate grading through to ovate-lanceolate, apex cuspidate; lamina 6–8-nerved with poorly defined midrib and bearing up to 10–20 oil glands between nerves; lamina surface initially sparsely covered in deciduous long silky silvery-white hairs, soon becoming glabrate, except for a distinct stout weft on the cuspidate apex. *Inflorescence* mostly a compact, corymbiform (4–)9(–20)-flowered botryum 7.0–20.0 mm long and usually terminated by a tuft of leaves and a semi-dormant vegetative bud; inflorescences initially present on brachyblasts in the distal one-third of the active branchlets, increasing in abundance and soon dominating all the distal terminal and lateral growth; on occasion inflorescences may extend to elongated botrya on late season’s vegetative growth. Inflorescence axis densely invested with antrorse-appressed, weakly flexuose, silky hairs. *Pherophylls* deciduous (shed early during bud maturation), rarely present at flowering, foliose or squamiform, basal portion tightly clasping pedicel base; (1.0–)1.2 × (0.2–)0.4 mm; foliose pherophylls pale green to red-green, oblong to oblong-lanceolate, very rarely broadly spathulate, cuspidate, deeply concave in cross section, with the abaxial surface copiously invested in sericeous, antrorse-appressed hairs; oil glands scarcely evident, up to 10 (usually less); midrib not evident; squamiform pherophylls tightly clasping pedicels, 0.3–1.0 × 0.4–0.8 mm, red-brown to brown, broadly to narrowly ovate or lanceolate, apex acute, subacute to obtuse, weakly keeled, margins and distal portion of keel finely ciliate. *Pedicels* (2.8–)5.7(–7.3) mm long at anthesis, scarcely elongating after anthesis, terete, initially invested with silky, antrorse-appressed, weakly flexuose, hairs becoming glabrate. *Flower buds* (2.3–)3.8(–4.9) × (2.1–) 3.1(–4.2) mm, ovoid to pyriform, apex flat to weakly domed prior to bud burst with calyx lobes held flat across surface, rarely meeting. Fresh flowers when fully expanded (5.7–)8.1(–10.2) mm diam. *Hypanthium* (1.9–)2.6(–3.6) × (2.1–)3.1(–4.2) mm, with free portion 0.4–0.7 mm long, silvery-white to silvery grey or reddish-grey due to copious covering of hairs; narrowly obconic to obconic or cupular, terminating in a scarcely defined chartaceous rim bearing five persistent calyx lobes; hypanthium surface smooth when fresh becoming irregularly wrinkled when dry, somewhat finely glandular punctate, oil glands scarcely evident due to dense covering of long, silky, antrorse-appressed silvery hairs, ribs not evident. Calyx lobes 5, initially erect to suberect, sometimes spreading, submembranous, (1.1–)1.3(–1.6) × (0.9–)1.2(–1.8) mm, broadly obtuse, red-green to pale green with a white or pink membranous margin, not obviously keeled, sparsely and finely gland-dotted, oil glands ± colourless; lobe margins finely ciliate, hairs eglandular, central portion of lobes densely covered in short silky, antrorse-appressed hairs. Receptacle greenish pink or pink at anthesis, darkening to crimson after fertilisation. *Petals* 5(–6), (2.0–)2.9(–3.6) × (2.1–)2.7(–3.3) mm, white, very rarely basally flushed pink, broadly ovate, suborbicular to orbicular, rarely ± cuneate-truncate, spreading, upper third often weakly recurved, apex rounded, margins ± finely and irregularly crumpled or frayed, oil glands not evident in fresh or dried material. *Stamens* 18–38(–46) in 1–2 weakly defined whorls, arising from receptacular rim, filaments white. Antipetalous stamens (2–)3(–4), antisepalous (2–)4(–6). Outermost antipetalous stamens outcurved, widely spreading, more rarely slightly incurved, on filaments 2.0–3.6 mm long, inner stamen if present, 0.4–0.9 mm, outcurved or incurved. Antisepalous stamens usually shorter than outermost antipetalous stamens, 0.6–3.6 mm, weakly incurved or outcurved, usually in mixtures of both. Anthers dorsifixed, 0.06–0.1 × 0.06–0.09 mm, broadly ellipsoid to scutiform, latrorse. Pollen white (11.9–)15.4(–19.9) μm. Anther connective gland pale pink when fresh, drying pale orange, spheroidal, coarsely papillate. *Ovary* (3–)4(–5) locular, each with 18–22(–34) ovules in two rows on each placental lobe. Style 1.8–2.5(–3.0) mm long at anthesis, elongating slightly after anthesis, white basally flushed pink or pale pink; stigma narrowly capitate, as wide as or scarcely wider than style, ± flat, greenish-pink or pink, flushing red after anthesis, surface finely granular-papillate. *Fruits* long persistent, copiously covered in short, silky, antrorse-appressed hairs; (2.2–)3.0(–3.6) × (2.7–)3.2(–3.9) mm, initially graphite grey, maturing to charcoal at dehiscence, and in old dehisced capsules fading to greyish-white; narrowly obconic to obconic, rarely cupular, calyx valves persistent, incurved, somewhat chartaceous, splits concealed by dried, erect, free portion of hypanthium. *Seeds* 0.52–1.04(–1.09) × 0.38–0.58(–0.72) mm, obovoid, oblong, or oblong-ellipsoid, usually curving toward apex, laterally compressed, 2–3-angled with convex to flattened faces, apex rounded to subacute; base oblique, ± flattened; testa semi-glossy, orange-brown to dark brown, surface coarsely reticulate. FL: (Sep–)Nov–Jan(–Mar). FT: Feb–May(–Jul). Chromosome Number n = 11_II_, 2*n* = 22 (see de Lange and Murray 2004).

#### Representative specimens

**(111 sheets seen).** **Aotea (Great Barrier Island).** Kaiarara Plateau, R. C. Lloyd s.n., Nov 1950, (CHR 92451); Perry's Hill, R. C. Lloyd s.n., Oct 1973, (AK 133455, 133456); South slope of Mt Hobson, C. C. Ogle 470, 13 Apr 1980, (CHR 367190); Mt Hobson - Windy Canyon Track, P. J. de Lange 4537 & B. G. Murray, 7 Mar 1999, (AK 287195, Duplicates: CANB, HO, NSW, Z); Overton (Harataonga) Access Road, P. J. de Lange 4562 & G. M. Crowcroft, 7 Oct 2000, (AK 255943, Duplicate: AD); Mt Heale, Eastern Side, P. J. de Lange 4867, 18 Dec 2000, (AK 286128, Duplicates: AD, MEL, P); Peach Tree Track, P. J. de Lange 6266 & D. A. Norton, 18 Apr 2002, (AK 288493, Duplicate: CHR); Motukaikoura Island, North side of main ridge, south-west of Bradshaw Cove, E. K. Cameron 14105, 17 Dec 2006 (AK 298033).

#### Distribution

**(Fig. 7).** Endemic, New Zealand, Aotea (Great Barrier Island) (20–510 m a.s.l.). On Aotea (Great Barrier Island) found mostly on the rhyolite outcrops of the central high points and western slopes (de Lange and Norton 2004).

#### Recognition.

As discussed under *Kunzea triregensis*, there has been much confusion over the exact circumscription and identification *Kunzea sinclairii*. Nevertheless, as defined here *Kunzea sinclairii* is endemic to Aotea (Great Barrier Island). It differs from all other New Zealand *Kunzea* by its usually prostrate trailing habit (Figs 49A, 51C), and its consistently silvery-grey, uniformly hairy leaves (see Table 1). It is also the only *Kunzea* endemic to rhyolitic rock (de Lange and Norton 2004). In the field, *Kunzea sinclairii* is sympatric (and often syntopic) with *Kunzea robusta* and, occasionally, *Kunzea linearis*, particularly along track sides, forestry roads, and formerly forested, now open clay pans and associated rock exposures. In these places it forms extensive hybrid swarms, mostly with *Kunzea robusta* but also, rarely, with *Kunzea linearis* and, apparently uniquely amongst New Zealand *Kunzea*, with *Leptospermum scoparium* (de Lange et al. 2005).

Kirk (1869) quickly recognised the novelty of *Kunzea sinclairii*. Many of his herbarium specimens are annotated with detailed enthusiastic descriptions of the species. For example, T. Kirk 75 (K!) carries the following observations ‘suckering or prostrate – producing its flowers in such immense abundance as often to hide the leaves. Often closely pressed to the rocks like an alpine plant and resembling in foliage *Phlox verna*! & [*sic*] might easily be taken for an herbaceous plant – small patches from 3 inches broad in the faces of the rocks were simply patches of flowers. Flowers fragrant’. While on the label of a specimen in MEL(!) (T. Kirk 399) Kirk waxes lyrical about the flowers, their profusion and concludes that the species is ‘a capital bedding plant! However, as is evident from his herbarium specimens at WELT and those he lodged overseas, he remained uncertain as to what rank seemed most appropriate for the plant until his final 1899 posthumous publication of it at species rank (Kirk 1899).

This uncertainity is reflected in Kirk’s vague distinctions. For example in his original diagnosis (Kirk 1869) where he treated the species as Leptospermum ericoides var. pubescens, he provided few distinguishing characters. His subsequent diagnosis of *Leptospermum sinclairii* (Kirk 1899; p. 158), although more detailed, still offered few discriminating characters (‘...distinguished at sight by the white silky leaves, larger flowers, longer pedicels. The ovary is sunk fully one-third below the narrow calyx-tube, while the sepals and petals are narrower, and the style extremely slender. The flowers are deliciously fragrant.’). This admittedly vague diagnosis may have prompted Cheeseman’s standard cynical remark, which he used ‘politely’ when dealing with taxa he thought dubious (see de Lange et al. 2014) that it was ‘a somewhat critical’ species (Cheeseman 1914). Whatever his misgivings, Cheeseman nevertheless still accepted the species, as did all subsequent authors until the perfunctory treatment of Thompson (1983) who merged it (along with several other distinct Australia species and New Zealand varieties) in the oldest available name *Leptospermum ericoides* A.Rich. as *Kunzea ericoides* (A.Rich.) Joy. Thomps.

Harris (1987) did not agree with the circumscription of *Kunzea ericoides* offered by Thompson (1983) and he made the necessary combination at species rank in *Kunzea* for *Leptospermum sinclairii*. However, in that account he again offered little basis for that decision. Subsequently, Harris et al. (1992) discussed the species in more detail, though, oddly, as neither he nor his co-authors had seen the species in the wild, their perceptions of its ecology, and statements about its growth habit were based entirely on the behaviour of cultivated specimens supplemented by field observations gleaned from herbarium labels and comments made by other people who had actually seen it in the wild (B. P. J. Molloy pers. comm.). Although their conclusions as to rank are accepted here, I provide a more detailed description of the species based on direct field observations and wild collected specimens. Unique defining characters of *Kunzea sinclairii* include its usually prostrate widely spreading habit (Figs 49A, 51C–E), tendency for the branches to layer on contact with the soil, broadly lanceolate, elliptic to obovate, uniformly sericeous hairy leaves (Fig. 49E–G), and the unusual inflorescence type, which is an aggregated corymbiform conflorescence (see Briggs and Johnson 1979). The leaves and young branchlets of *Kunzea sinclairii* differ from all other New Zealand *Kunzea* in that they are often strongly red-pigmented, particularly along the lamina margins, and it is this dark colour which, when overlaid by the copious light reflecting antrorse-appressed hairs (Fig. 49A–D), helps to impart the characteristic silvery colour of the plant. As Kirk (1869, 1899) observed, the new growth appears white, due to the dense covering of hairs, which are partially shed as the leaf matures, thereby causing the white ‘colour’ to fade first to silver, and ultimately to the more usual silver-grey typical of mature foliage. Although the flowers of *Kunzea sinclairii* were referred to by Kirk as larger than *Kunzea ericoides*, borne on longer pedicels and ‘deliciously fragrant’ is not confirmed. In fact the flower size of *Kunzea sinclairii* falls within the range commonly seen in *Kunzea robusta*, *Kunzea triregensis* and *Kunzea amathicola* (see Table 1), species which, in Kirk’s time were included in *Kunzea ericoides*. Nevertheless, they are larger than the range usually seen in *Kunzea ericoides* *s.s.* (see Table 1). Similarly, pedicel length is not diagnostic, the length varying with the growing conditions of the plant. Finally, the majority of the fresh flowering specimens of *Kunzea sinclairii*, in common with the rest of *Kunzea ericoides* group treated here, had little or no discernible scent. However, in one respect the flowers of *Kunzea sinclairii* are distinctive: the external hypanthium surface, calyx-lobes and receptacular disc are usually strongly pigmented red or pink, a background colour which helps highlight the copious covering of silky hairs on the external face of the hypanthium.

The ITS and ETS sequence data obtained for *Kunzea sinclairii* is unremarkable (de Lange 2007; de Lange et al. 2010). A relationship to *Kunzea amathicola* and *Kunzea robusta* is inferred in the ETS sequence at alignment position 269 (Table 2) where all three taxa share a guanine (de Lange 2007). *Kunzea sinclairii* also has the same chromosome karyotype as that seen in *Kunzea amathicola* and *Kunzea triregensis* (de Lange and Murray 2004). Irrespective, *Kunzea sinclairii* is easily distinguished from all three species (Table 1). The confusion of this species with *Kunzea triregensis* has already been discussed (see above). More puzzling by far is that *Kunzea sinclairii* is frequently confused with the very different with *Kunzea linearis*. Notably, Webb and Simpson (2001; p. 239, pl. 96), despite accurately recording the distribution of *Kunzea sinclairii*, based their seed description, illustration and voucher on a Bay of Islands specimen of *Kunzea linearis* (WELT SP029320!) that had been incorrectly identified by its collector, D. Petrie as *Kunzea sinclairii*. *Kunzea sinclairii* is easily distinguished from *Kunzea linearis* by its usually prostrate, trailing rather than erect tree habit, and uniformly hairy, silvery-grey, broadly lanceolate, oblanceolate to elliptic leaves (Fig. 48A–B, D–G, 51B–E). The flowers of *Kunzea sinclairii* are produced in mostly corymbiform rather than spiciform inflorescences (Fig. 48A, 51D–E), and are distinctly pedicellate rather than sessile to subsessile. The pherophylls are deciduous, usually short, broadly rhomboid, elliptic to lanceolate rather than long, linear to linear falcate, and persistent (as in *Kunzea linearis*). Further details are provided in Table 1.

In its natural habitat the only *Kunzea* commonly found in association with *Kunzea sinclairii* is *Kunzea robusta*. Because *Kunzea sinclairii* is usually a prostrate shrub, and has uniformly, silvery-grey hairy leaves, confusion with the usually arborescent (up to 30 m tall) dark to light green, glabrescent leaved *Kunzea robusta* is unlikely. Additional distinctions are provided in Table 1.

#### Ecology.

The ecology of *Kunzea sinclairii* is described by de Lange and Norton (2004). They found that the species is naturally confined to rhyolitic rock of the central Hirakimata massif of Aotea (Great Barrier Island). Here it is primarily a rock tor, cliff and gorge endemic (Fig. 51A–C) occupying an estimated 90.5 ha (or 0.3% of the extent of Aotea (Great Barrier Island).

*Kunzea sinclairii* is usually the dominant woody plant within its preferred rock habitat. It rarely grows on well-developed soils, preferring fissures within rock outcrops, and their associated saprolite and/or skeletal soils (Fig. 51A–C). These habitats are characterised by being extremely infertile and with low species diversity (Harris et al. 1992; de Lange and Norton 2004). In their study de Lange and Norton (2004) found that in the main rock habitats *Kunzea sinclairii* generally grew in association with stunted kahikatoa (*Leptospermum scoparium* agg.), Phormium cookianum subsp. hookeri (Hook.f.) Wardle, and another Aotea (Great Barrier Island) rhyolite endemic, *Olearia allomii* Kirk, the lichen *Cladia retipora* (Labill.) Nyl. and the moss *Ptychomnion aciculare* (Brid.) Mitt.

The bark and twigs of well-established plants of *Kunzea sinclairii* are often festooned with corticolous and epiphyllous liverworts, particularly from the genus *Frullania*, and the trunk bases and exposed roots are often encrusted by the liverworts *Cuspidatula monodon* (Lehm.) Steph., *Jamesoniella colorata* (Lehm.) Schiffn., and *Harpalejeunea filicuspis* (Steph.) Mizut. Occasionally the branches and branchlets of *Kunzea sinclairii* are heavily parasitised by the dwarf mistletoe *Korthalsella salicornioides*.

#### Hybridism.

It was the widely reported, putative, ‘rife’ hybridism with other *Kunzea* that was the basis for initial conservation concerns that *Kunzea sinclairii* might be at risk of extinction (Dopson et al. 1999; de Lange and Norton 2004). In the wild, *Kunzea sinclairii* is currently commonly sympatric (and syntopic) with *Kunzea robusta* and, much less frequently, *Kunzea linearis*. It is likely that the high level of sympatry between these species is not natural, and is the direct consequence of widespread habitat destruction that resulted from kauri logging of the island (Ogden 2001; de Lange and Norton 2004). Logging and the associated deliberate and accidental burning of the forest left large areas of bare land ideally suited to the establishment of primary colonisers, including *Leptospermum scoparium*, *Kunzea linearis*, *Kunzea robusta* and *Kunzea sinclairii*. Today, *Kunzea robusta* and *Leptospermum scoparium* form the dominant woody vegetation over most of the southern two thirds of Aotea (Great Barrier Island) (Ogden 2001). Widespread and frequent disturbance favours the proliferation of hybrids in *Kunzea* (de Lange et al. 2005), and this is especially so on Aotea (Great Barrier Island) where the putative hybrids *Kunzea linearis* × *Kunzea sinclairii*, *Kunzea robusta* × *Kunzea sinclairii* and the intergeneric ×*Kunzspermum hirakimata* W.Harris (*Kunzea sinclairii* × *Leptospermum scoparium*) have all been collected. All these hybrids were successfully generated by de Lange et al. (2005) who found that *Kunzea sinclairii*, whether as pistillate and staminate parent, readily crossed with seven New Zealand *Kunzea* species, and, only as the pistillate parent, with *Leptospermum scoparium*. No reduction in seed viability was recorded with those crosses involving *Kunzea* species but the intergeneric hybrids proved to be aneuploid and sterile. They also found that the progeny raised through two generations (F_2_, F_3_) from their F_1_ hybrid, *Kunzea sinclairii* × *Kunzea robusta* (as *Kunzea sinclairii* × Kunzea aff. ericoides (b) in their paper), showed clear segregation toward both parents, such that F_3_ individuals were scarcely distinguishable from the original parents used for the initial cross (de Lange et al. 2005). This suggests that not only can hybridism happen readily but that introgressed swarms can segregate quickly (within 5–8 years) to their constituent parents. In their ecological study de Lange and Norton (2004) concluded that hybridisation was not a threat to *Kunzea sinclairii*, which they considered to form ‘pure’ populations within its preferred rhyolitic rock outcrop habitats. They argued that hybrid swarms outside these rupestral habitats were the product of past human-induced disturbance and that over time they would be lost through succession to forest. While this appears to be happening over large parts of the island, field botanists are still faced with an often baffling array of hybrids, whose distinction from *Kunzea sinclairii* can be difficult.

Of the three naturally occurring hybrids involving *Kunzea sinclairii* recorded from Aotea (Great Barrier Island), the greatest difficulty is experienced recognising *Kunzea robusta* × *Kunzea sinclairii*. This is because the hybrid is present throughout large parts of the island and the adjoining islands as a complex array of introgressants. Further, *Kunzea sinclairii* has been inadequately circumscribed in the past, resulting in many herbarium specimens collected as this species proving on inspection to be mixed gatherings of *Kunzea sinclairii* and *Kunzea robusta* × *Kunzea sinclairii*, or just the hybrid *Kunzea robusta* × *Kunzea sinclairii*.

*Kunzea robusta* × *Kunzea sinclairii* is also far more wide ranging than *Kunzea sinclairii*. For example, it has been collected from Aiguilles Island at the northern end of Aotea (Great Barrier Island), some 50 km north of the northern-most known *Kunzea sinclairii* occurrence on Aotea (Great Barrier Island). In most cases the hybrid presents an intermediate growth habit, typically as an erect shrub to small tree, characteristically with glaucous green, pinkish-green or yellowish-green, oblanceolate to lanceolate leaves. The red or pink tones typical of the branchlets and foliage of *Kunzea sinclairii* are expressed in the hybrids as pale pink or even yellowish-pink. This is particularly evident in herbarium specimens where the hybrids tend to dry yellow-green or dark green, never the diagnostic greyish-white to silvery-white of *Kunzea sinclairii*. This colour change is accentuated by differences in the density of leaf indumentum which in the hybrid tends to be much less than is typical of *Kunzea sinclairii* though more copious than is usual for *Kunzea robusta*. Another difference is that, like those of *Kunzea robusta*, the hairs tend to be shed more freely as the leaf matures until they are mainly found on the abaxial surface; though in some examples they can form a sparse covering on the adaxial surface, particularly in the lower half to one-third of the leaf. Because the flowers of *Kunzea robusta* and *Kunzea sinclairii* are so similar, they offer little of diagnostic value. Similarly there is little consistent difference in fruit shape or size, indumentum cover or seed size.

The other two hybrids *Kunzea linearis* × *Kunzea sinclairii* and the intergeneric ×*Kunzspermum hirakimata* are very uncommon. *Kunzea linearis* × *Kunzea sinclairii* is discussed under *Kunzea linearis* while ×*Kunzspermum hirakimata* was described in detail by Harris et al. (1992) and Harris (1993).

#### Vernacular name.

No specific Maori name for this species has been recorded.

#### Conservation Status.

*Kunzea sinclairii* is appropriately assessed as ‘At Risk/Naturally Uncommon’ qualified ‘IE’ (Island Endemic) and ‘RR’ (Range Restricted) by de Lange et al. (2013b).

### 10. Kunzea robusta

Taxon classificationPlantaeMyrtalesMyrtaceae

de Lange et Toelken sp. nov.

urn:lsid:ipni.org:names:77141733-1

A K. ericoides (A. Rich.) Joy Thomps. habitu heterophyllo, indumento in ramulis adultis persistenti sericeo abundanti plerumque antrorso-appresso raro interdum pilis divergentibus, fructibus late obconicis vel turbinatis raro cupulatis plerumque pubescentibus differt. Etiam propriis chromosomatibus recedit.

#### Holotype

**Figure 52.**
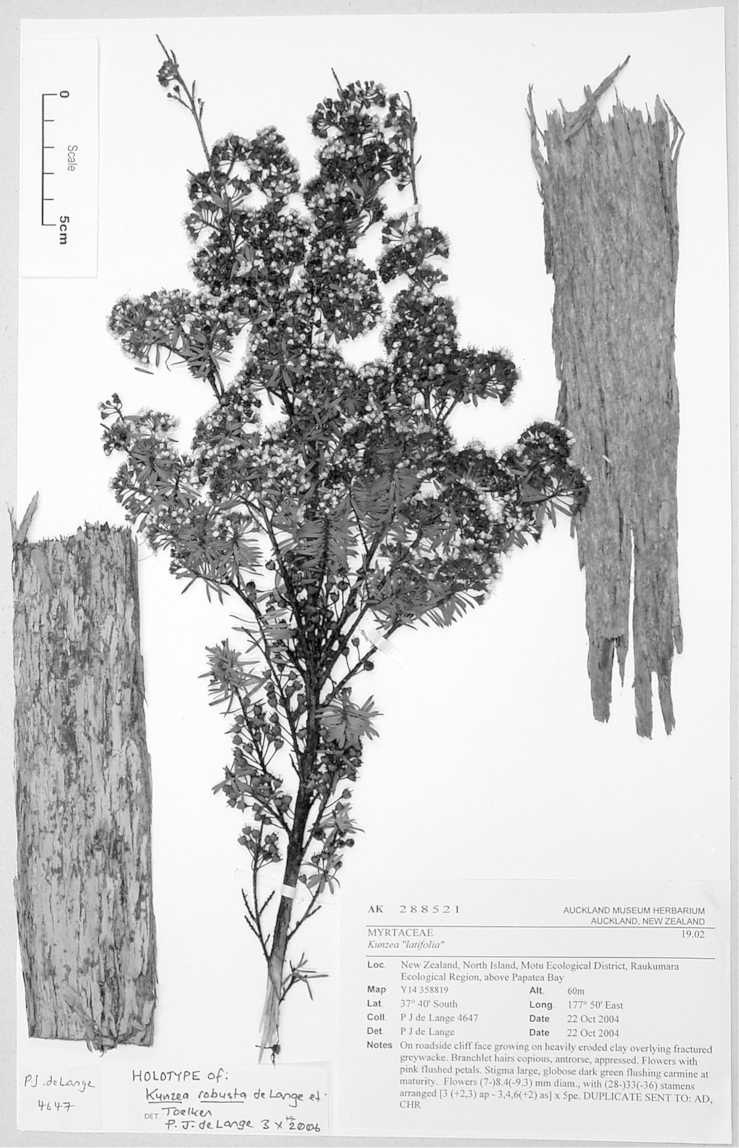
Holotype of *Kunzea robusta* de Lange et Toelken (*P. J. de Lange 4647*, AK 288521).

**(Fig. 52).** New Zealand, North Island, Raukumara Ecological Region, Motu Ecological District, above Papatea Bay, 37°40'S, 177°50'E, 60 m a.s.l. ‘On roadside cliff face growing on heavily eroded clay overlying fractured greywacke’. P. J. de Lange 4647, 22 Oct 2004, AK 288521! Isotype AD! CHR!

#### Etymology.

The specific epithet *robusta* alludes to the stature of mature trees of this species which can, under stable conditions, attain 30 m tall and trunks of up to 1 m d.b.h.

#### Description

**Figure 53.**
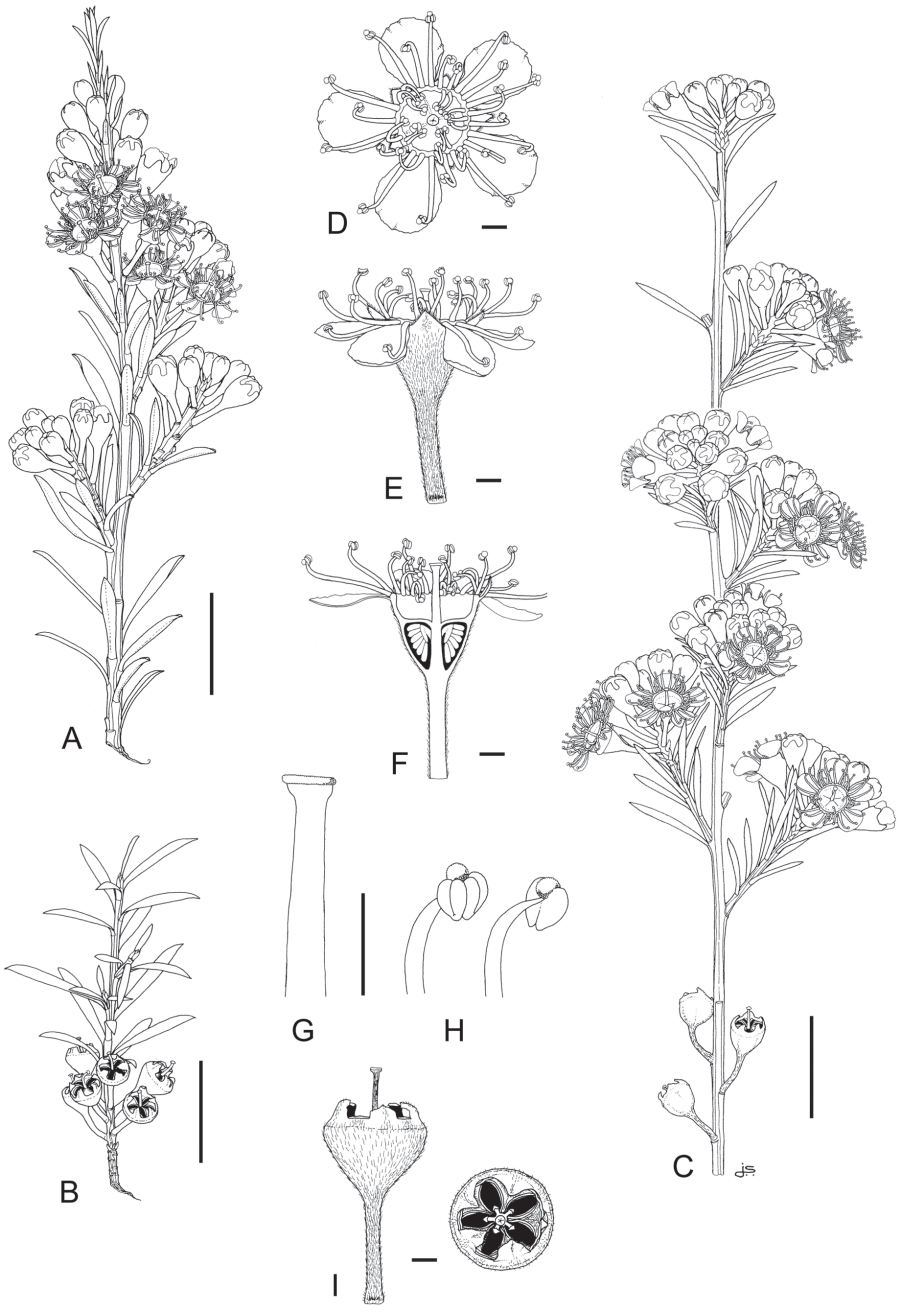
Distinguishing features of *Kunzea robusta*. **A** Flowering branchlets of common variant (no voucher, North Island, Auckland, Green Bay) **B** Fruiting branchlet (AK 285561) **C** Flowering branchlets of eastern North Island variant, (AK 288499) **D** Flower (top view) (ex cult. AK 285561) **E** Flower and hypanthium (side view) (ex cult. AK 285561) **F** Flower cross section showing anther, style and ovules (ex cult. AK 285561) **G** Style and stigma (ex cult. AK 285561) **H** Stamens (ex cult. AK 285561) **I** Dehisced fruit (ex cult. AK 285561). Scale bars: (**A–C**) 10 mm; (**D–I**) 1 mm.

**Figure 54.**
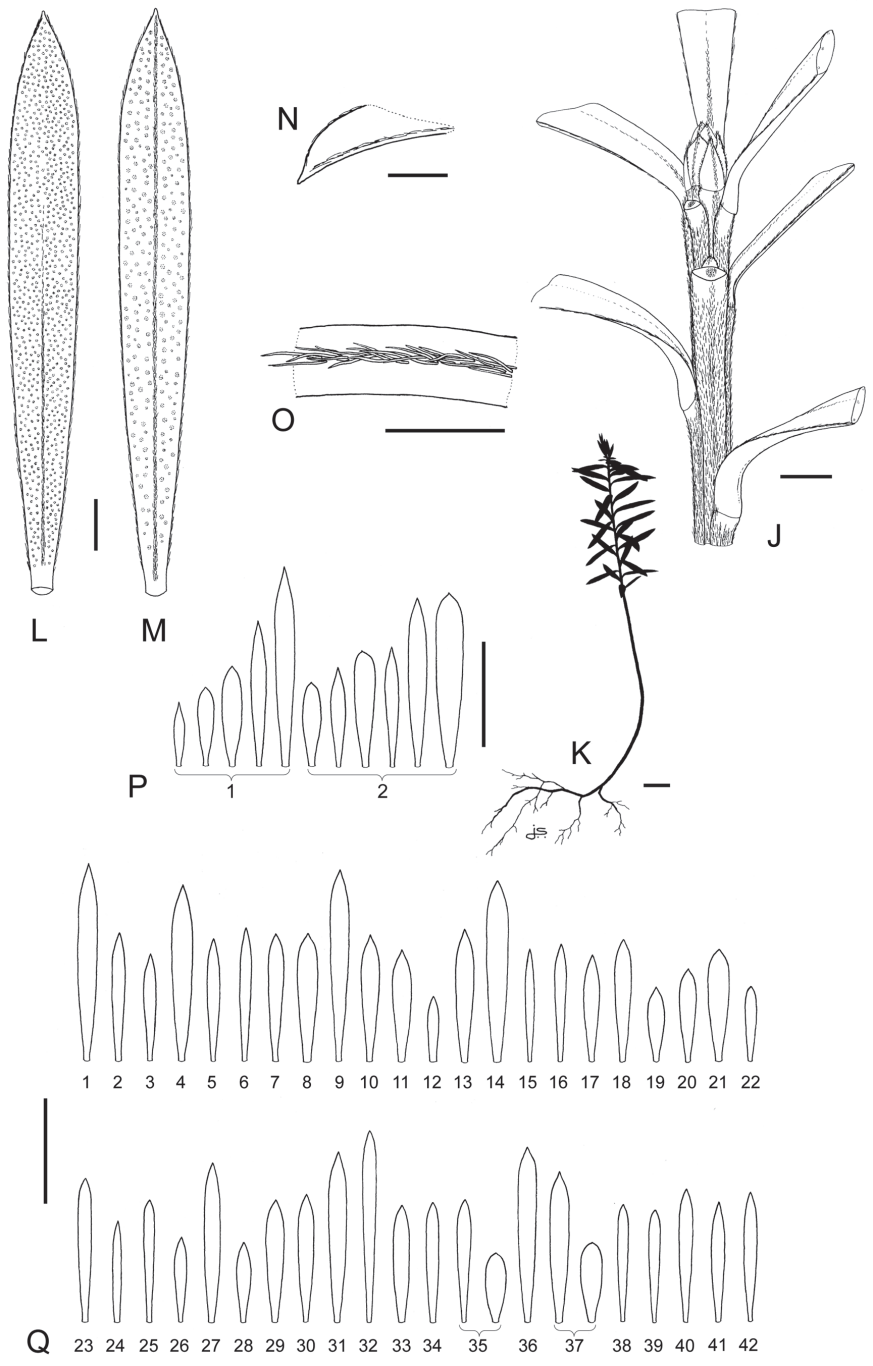
Distinguishing features of *Kunzea robusta* continued. **J** Vegetative bud and branchlet indumentum (no voucher, North Island, Auckland, Green Bay) **K** Seedling of common variant (no voucher, North Island, Albany Scenic Reserve) **L** Adaxial leaf surface (no voucher, North Island, Auckland Green Bay) **M** Abaxial leaf surface (no voucher, North Island, Auckland Green Bay) **N** Adaxial leaf apex (no voucher, North Island, Auckland Green Bay) **O** Leaf margin indumentum (no voucher, North Island, Auckland Green Bay) **P** Leaf variation within two individuals (**P1**) North Island, Auckland, Green Bay (no voucher), (**P2**), North Island, Hapuakohe Range, Wai Iti Road, (ex cult. AK 285561) **Q** Leaf variation: (**Q1**) North Island, Cavalli Island (AK 150268), (**Q2**) North Island, Whangaroa Harbour (AK 226190), (**Q3**) North Island, Puketi (AK 169749), (**Q4**) North Island, Mangatoa Stream (AK 254925), (**Q5**) North Island, Mokohinau Islands group (AK 226069), (**Q6**) North Island, Puhoi (AK 250787), (**Q7**) North Island, Waikawau Bay (AK 245109), (**Q8**) North Island, Mangatawhiri Valley (AK 208449), (**Q9**) North Island, Kauaeranga Valley (AK 242671), (**Q10**) North Island, Whangamarino (AK 242673), (**Q11**) North Island, Hamilton, Hammond Bush (AK 207190), (**Q12**) North Island, Kohioawa Beach (AK 287041), (**Q13**) North Island, Moutohora (Whale Island) (AK 289818), (**Q14**) North Island, Whakatane, Kohi Point (AK 289950), (**Q15**) North Island, Torere (AK 289977), (**Q16**) North Island, Hicks Bay (AK 285565), (**Q17**) North Island, Haupara Point (AK 288506), (**Q18**) North Island, Ruatoria (AK 286087), (**Q19**) North Island, Awaroa Scenic Reserve (AK 287864), (**Q20**) North Island, Lake Okataina (AK 288229), (**Q21**) North Island, Whakamaru (AK 288041), (**Q22**) North Island, Lake Waikaremoana (AK 287026), (**Q23**) North Island, Tangarakau River (AK 286129), (**Q24**) North Island, Kaweka Range (AK 288045), (**Q25**) North Island, Mahia Peninsula (AK 286160), (**Q26**) North Island, Frasertown (AK 287040), (**Q27**) North Island, Tangoio (AK 286251), (**Q28**) North Island, Kawhatau River (AK 288075), (**Q29**) North Island, Oroua (AK 288048), (**Q30**) North Island, Pohangina River (AK 288047), (**Q31**) North Island, Foxton (AK 288695), (**Q32**) North Island, Mangatainoka River (AK 289513), (**Q33**) North Island, Upper Tauweru River (AK 288023), (**Q34**) North Island, Putangirua Pinnacles (AK 287531), (**Q35**) South Island, D’Urville Island (AK 288513), (**Q36**) South Island Port Underwood (AK 288592), (**Q37**) South Island, Waima River (AK 286221), (**Q38**) South Island, Clarence River (AK 288569), (**Q39**) South Island, Happy Valley (AK 285567), (**Q40**) South Island, Banks Peninsula (AK 286135), (**Q41**) South Island, Buller River, near Westport (AK 288441), (**Q42**) South Island, Dunedin (AK 288441). Scale Bars: (**J, L–N**) 1 mm; (**K, P, Q**) 10 mm; (**O**) 0.5 mm.

**Figure 55.**
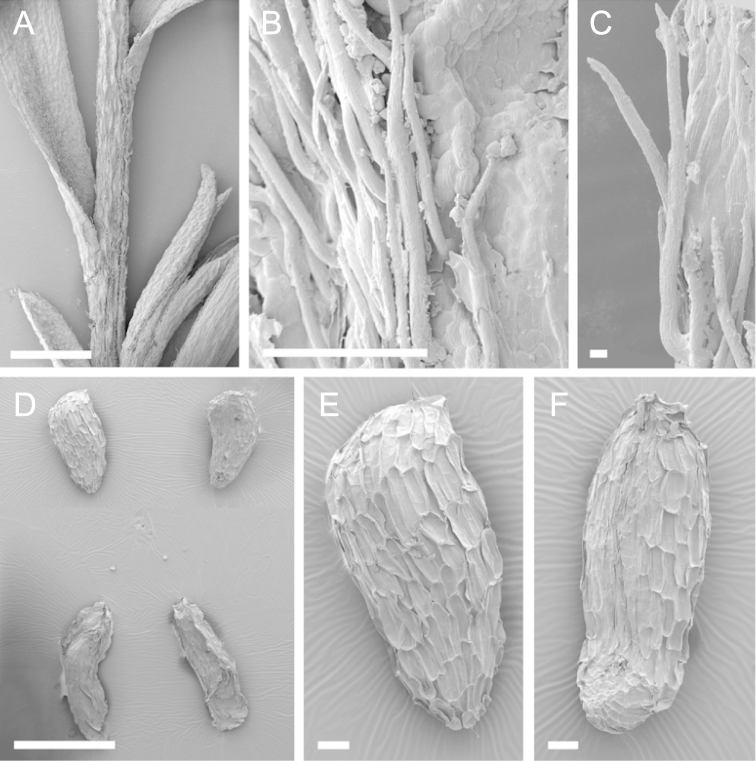
Scanning Electron Micrographs of *Kunzea robusta* (common variant). (**A–C** all AK 285565) Branchlet indumentum **D–F** Seeds (AK 285565). Scale bars: (**A**) 1 mm; (**B**) 100 μm; (**C**) 10 μm; (**D**) 1mm; (**E, F**) 100 μm.

**Figure 56.**
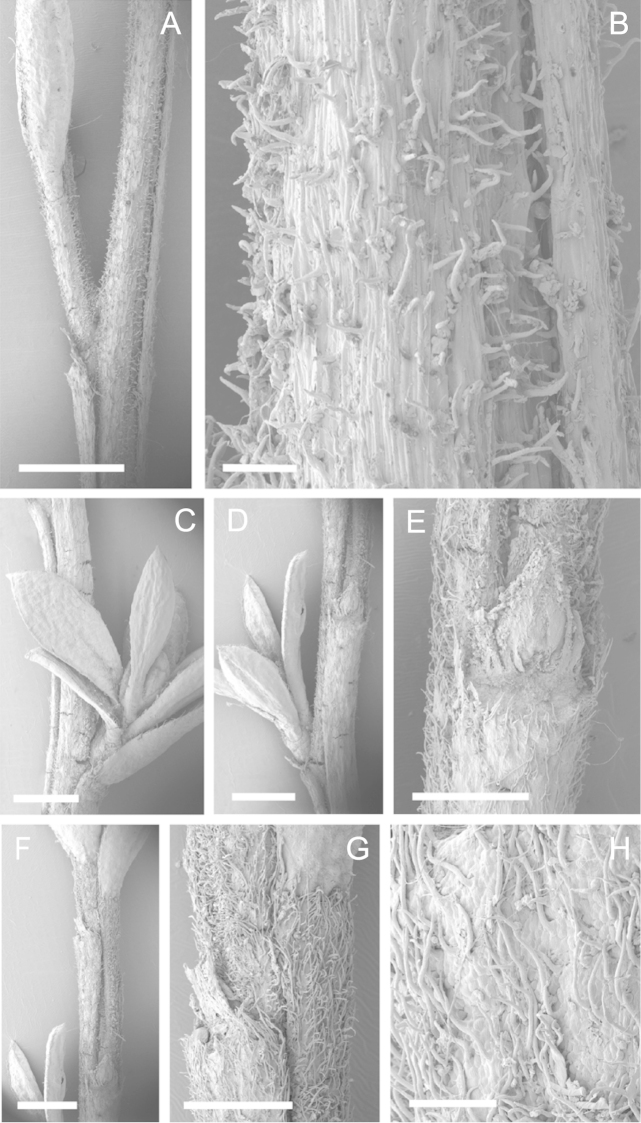
Scanning Electron Micrographs of *Kunzea robusta* (Rangitikei variant). **A–H** all AK 288076 **A–B** Branchlet indumentum of juvenile **C–H** Branchlet indumentum of adult.

**Figure 57.**
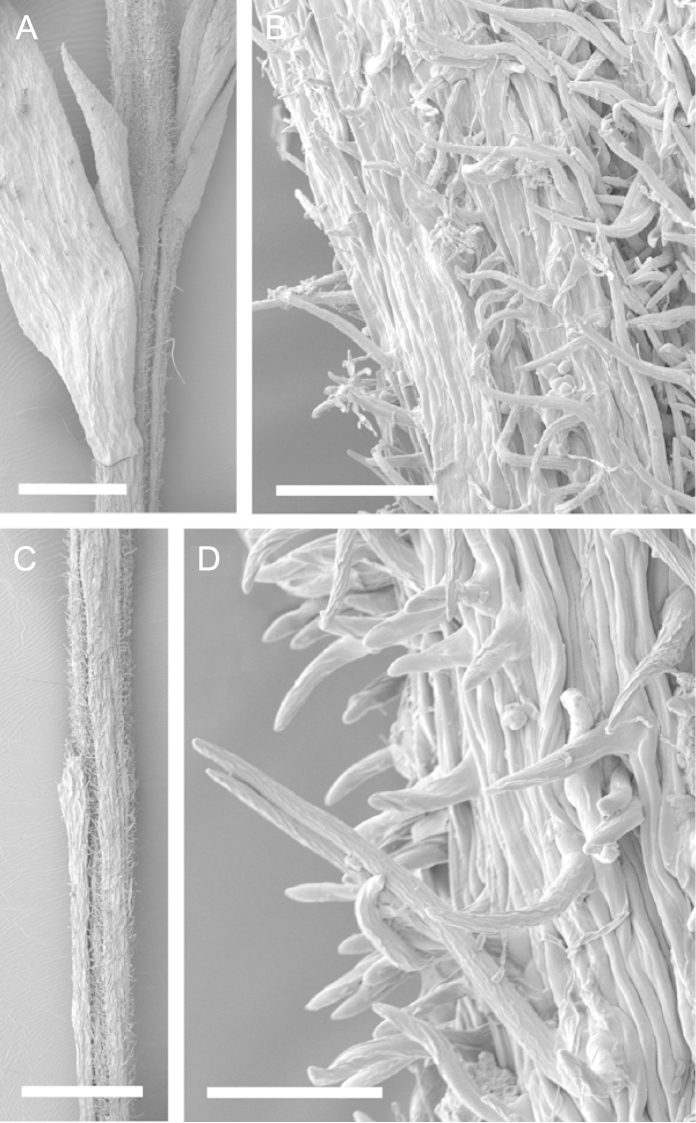
Scanning Electron Micrographs of *Kunzea robusta* (eastern North Island variant). **A–D** all AK 286067, Branchlet indumentum. Scale bars: (**A, C**) 1 mm; (**B**) 100 μm; (**D**) 50 μm.

**Figure 58.**
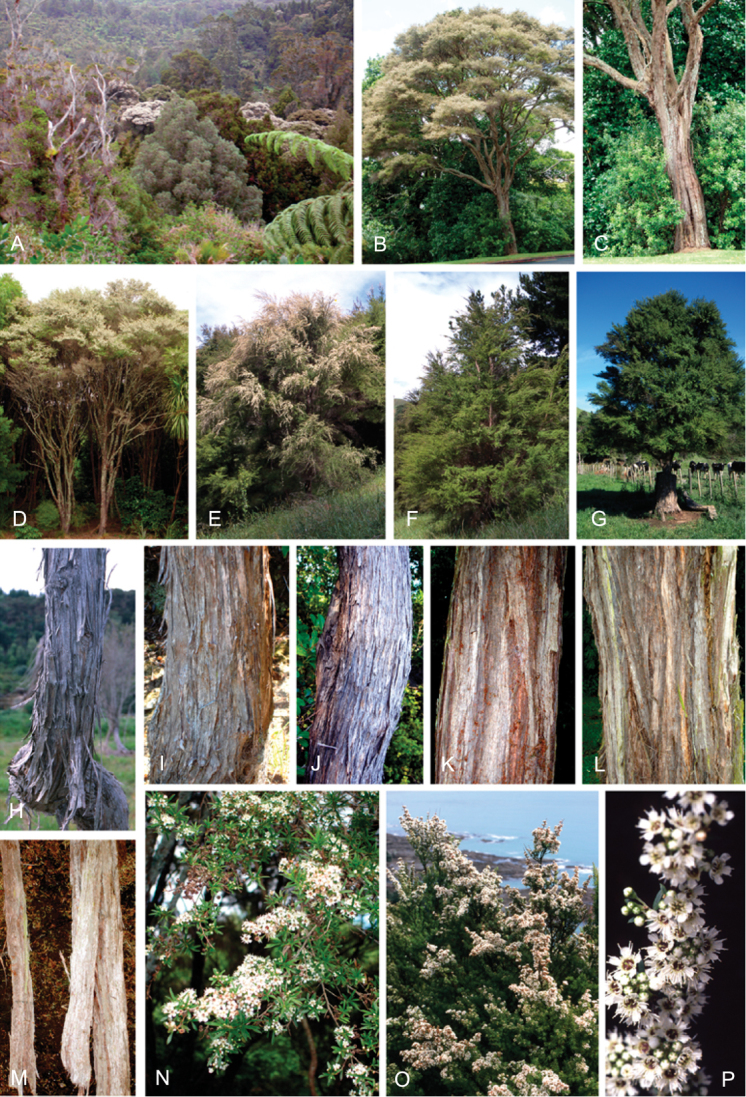
*Kunzea robusta*. **A** *Kunzea robusta* in full flower as an emergent in Kauri (*Agathis australis*) dominated forest, North Island, Waitakere Ranges (photo: *P. J. de Lange*) **B** Adult tree of *Kunzea robusta* showing distinctive growth habit and broad, spreading canopy, North Island, Auckland City (photo: *P. J. de Lange*) **C** Trunk and lower branches of *Kunzea robusta* showing branching pattern and bark (photo: *P. J. de Lange*) **D** *Kunzea robusta* young trees of the common variant in full flower, North Island, Auckland, Western Springs (photo: *P. J. de Lange*) **E** *Kunzea robusta* example of the fine-leaved eastern North Island variant, Hawke’s Bay, Tangoio (photo: *P. J. de Lange*) **F** Young tree of *Kunzea robusta* in open pasture showing branching from base, North Island, near Wairoa (photo: *P. J. de Lange*) **G** *Kunzea robusta* tree protruding from *Cupressus macrocarpa* stump in pasture, North Island, Wairarapa (photo: *J. E. Braggins*); (**H–M**) *Kunzea robusta* bark types: (**H**) North Island, Wairakei **I** North Island, Kendal’s Bay **J** North Island, Hunua Range, (**K, L**) North Island, Hamilton, Waikato River **M** Bark flakes showing narrowly, tabular shape and regular margins, North Island, Hamilton, Waikato River (photos: *P. J. de Lange*) **N** Flowering branchlets, North Island, Green Bay (photo: *P. J. de Lange*) **O** *Kunzea robusta* holotype tree in full flower, North Island Papatea Bay (photo: *P. J. de Lange*) **P** Close up of flowers, Aotea (Great Barrier Island) (photo: *G. M. Crowcroft*).

**(Figs 53, 54, 55, 56, 57, 58).** *Growth habit* trees (8–)20–25(–30) m tall, rarely decumbent shrubs up to 1 × 3 m; trees, depending on local conditions, mostly forming broad spreading canopies; in exposed situations branching at or close to the trunk base, while those growing in dense stands or sheltered sites usually with the lower 50–75% of the trunk devoid of branches. *Trunk* 1(–6) erect, 0.10–0.65(–1.0) m d.b.h.; mature trees usually devoid of branches for at least the first 1–3 m, basal portion of trunks covered with firm to semi-detached, stringy to coarsely tessellated, corky-coriaceous bark. *Bark* early bark subcoriaceous, grey-brown, cinnamon brown or dark brown, elongate, usually bearing deep transverse cracks (especially on branch flanges and decurrent leaf bases) otherwise firmly attached, margins elongate, sinuous, ± entire with scarcely any flaking; old bark either stringy, or coarsely tessellated, mostly corky-coriaceous, though in dense forest stands tending toward subcoriaceous or chartaceous, firmly attached above, detaching basally, often hanging semidetached; peeling upwards along trunk in narrow to broad, tabular strips up to 4 m long, margins ± entire to weakly irregular, usually straight; upper surface either ± smooth with coarsely tessellated but firm upper surface, or deeply and longitudinally corrugated and cracked (rarely peeling); early bark flakes usually crumbling in hand, old bark strips firm and not crumbling, snapping with a ± entire margin. *Branches* initially arising from or close to trunk base; these initial branches progressively dying, such that branches are increasingly confined to the upper 50–75% of trunk. Branches weakly flexuose or not, initially erect, soon arching outwards and spreading with distal ends mostly erect, rarely with whole branch or distal portion completely pendulous; branchlets numerous, slender, clustered toward branch ends, ± quadrangular to subterete, with leaves ± evenly spaced along length or in exposed situations, crowded toward apices; branchlets sericeous, indumentum copious, hairs hyaline to translucent (appearing white when young, maturing grey); mostly either long or short antrorse-appressed; if long, then usually weakly flexuose hairs 0.15–0.20(–0.38) mm long; if short, not flexuose, 0.09–0.15 mm long. In eastern Coromandel Peninsula and coastal East Cape to near Mahia Peninsula, branchlet indumentum in mixtures of mainly short (0.03–)0.05(–0.08) mm long divergent hairs, and sparse, 0.1–0.2 mm long, antrorse-appressed hairs (see Fig. 57A–D). In the Rangitikei region, seedling and juvenile plants up to 2 m tall have branchlet hairs mostly divergent, short (0.04–)0.08(–0.10) μm long. *Vegetative buds* conspicuous; at resting stage 0.3–2.8 mm diam., ovoid to broadly ellipsoid; scales scarious, deciduous or persistent, 0.6–0.8(–1.4) mm long, amber, red-brown to wine-red, basally broadly ovate, grading through ovate-deltoid to broadly lanceolate, cuspidate; midrib prominent, strongly keeled, prolonged to short cuspidate tip, lateral veins absent, colliculate, with oil glands, scattered, colourless, drying the same colour as the scale body, apical scale margins, keel, and keel apex copiously covered in long, white, sericeous hairs. *Leaves* with distinct juvenile and adult forms, sessile to shortly petiolate, well spaced to crowded along branchlets, spreading, suberect to patent, flat to weakly recurved in apical 30–50%, light green or dark green above, paler beneath; oblanceolate, broadly oblanceolate, broadly lanceolate, lanceolate to linear-lanceolate, rarely elliptic to obovate; apex subacute to acute, rarely obtuse, rostrate or shortly apiculate, base attenuate to narrowly attenuate; adaxial surface flat, weakly convex to slightly v-shaped; oil glands up to 600, evident when fresh, becoming more conspicuous when dry, midrib very slightly raised near base, otherwise not evident for rest of length, leaf base finely covered in antrorse-appressed, silky hairs, glabrate; abaxial surface slightly concave to flat or v-shaped in apical recurved portion otherwise weakly concave, finely glandular punctate, oil glands abundant up to 500, more evident when dry; midrib slightly raised for entire length, prolonged slightly at apex, hairs as for adaxial surface; lamina margin initially finely covered with a thin often interrupted band of 0.2–0.8 mm long, flexuose, spreading to antrorse-appressed hairs not or rarely meeting at apex; hairs mostly shedding with age, usually with only the basal portion ± retained. Lamina of juvenile plants from mainly coastal areas and northern North Island (14.6–)19.0(–28.4) × (1.6–)2.2(–2.5) mm; from inland areas, especially the Rangitikei, central and northern Wairarapa and Mt Egmont, (3.2–)4.6(–6.3) × (0.7–)1.2(–1.5) mm; adult lamina of plants from mainly coastal areas and northern North Island (4.9–)14.2(–20.1) × (0.9–)1.7(–3.0) mm; from inland areas, especially from the central North Island, Rangitikei, Wairarapa, and Central Otago (5.8–)9.3(–12.3) × (1.2–)1.8(–2.2). *Perules* usually very conspicuous, rarely obscured by surrounding leaves; at resting stage 1.9–3.0 mm diam., broad ovoid, ovoid, narrow-ovoid to broadly ellipsoid, squamiform; scales scarious, persistent, 0.6–1.0(–1.6) mm long, red-brown to red, basally broadly ovate, grading through ovate-deltoid to broadly lanceolate, cuspidate; midrib prominent, becoming even more on old or dried specimens, strongly keeled, prolonged to a short cuspidate tip (this becoming more obvious on drying), lateral veins absent, colliculate, with oil glands, scattered, colourless, drying the same colour as the scale body, upper scale margins, keel, and keel apex copiously covered in long, white, silky hairs. *Inflorescence* mostly a compact corymbiform to shortly elongate (1–)12(–30)-flowered botryum up to 60 mm long; usually on brachyblasts with the terminal shoot corymbiform or extending toward the end of the flowering season as a slightly longer (up to 80 mm long) 4–12-flowered, elongate botryum; flowers usually crowded, terminal portion usually bearing undeveloped flowers and dormant vegetative bud or active vegetative growth. Inflorescence axis densely invested with short, spreading to antrorse-appressed silky hairs. *Pherophylls* deciduous or more or less persistent; squamiform grading into foliose; squamiform pherophylls tightly clasping pedicels, 0.4–1.2 × 0.3–0.6 mm, red-brown to brown, broadly to narrowly deltoid or lanceolate, apex acute, subacute to obtuse, weakly keeled, upper keel and margins finely ciliate; foliose pherophylls spreading, flat or weakly recurved, (6.0–)9.0(–17.9) × (1.1–)1.2(–1.8) mm, green, elliptic, oblanceolate, broadly lanceolate to lanceolate, apex obtuse, cuspidate, base attenuate; adaxial surface usually convex to weakly v-shaped, oil glands 10–30(–50), midrib slightly raised near base, evident for rest of length, basally covered in sparse to dense, antrorse-appressed, silky hairs; abaxial surface flat or weakly convex, glandular punctate, oil glands 20–40; midrib raised for most of length, densely covered in antrorse-appressed, silky hairs to apex, lamina margin obscured by dense covering of antrorse-appressed hairs. *Pedicels* (1.2–)3.8(–5.2) mm long at anthesis, usually elongating slightly after anthesis, terete, sparsely to densely invested in antrorse-appressed, weakly flexuose, silky hairs. *Flower buds* pyriform to obconic, apex flat or weakly domed prior to bud burst; calyx valves not meeting. Fresh flowers when fully expanded (4.3–)7.7(–12.0) mm diam., usually reducing in size toward end of flowering season. *Hypanthium* (2.1–)3.1(–4.1) × (3.0–)3.9(–5.2) mm, with free portion 0.4–0.9 mm long, dark green or red-green, drying green-brown or red-brown; mostly broadly obconic to turbinate, sometimes cupular, terminating in dark-green to red-green coriaceous rim bearing five persistent calyx lobes. Hypanthium surface when fresh faintly ribbed and sparingly dotted with pink or colourless oil glands, these drying dull yellow-brown or brown; either finely pubescent with the ribs and veins conspicuously covered in longer silky, antrorse-appressed hairs, or glabrous; hypanthium similar when dry though with the ribs more strongly defined and clearly leading up to calyx lobes. Calyx lobes 5, persistent, mostly spreading, coriaceous, (0.52–)0.83(–1.1) × (0.60–)0.90(–1.4) mm, pale green to red-green, broadly ovate, ovate-truncate to broadly obtuse, weakly keeled, external face of keel usually obscured by a broad band of antrorse-appressed, silky, white hairs, otherwise glabrous; margins white or pale green often flushed pink, surface somewhat sparsely glandular punctate, oil glands ± colourless when fresh drying dark yellow to yellow-brown, otherwise glabrate. Receptacle green or pink at anthesis, consistently darkening to crimson after fertilisation. *Petals* 5(–6), (1.5–)2.6(–3.8) × (1.3–)2.6(–3.6) mm, white, rarely pink (sometimes drying pale yellow or cream), spreading, orbicular, suborbicular to ovate, apex rounded to obtuse, margins ± finely and irregularly denticulate or crimped 1–6 or more times, rarely entire, oil glands colourless, drying opaque or grey. *Stamens* (15–)33(–58) in 2 weakly defined whorls, arising from receptacular rim, filaments white. Antipetalous stamens 3–5(–6) sometimes petaloid, antisepalous stamens (3–)5(–8). Outermost antipetalous stamens usually outcurved, sometimes weakly incurved or in mixtures of both on filaments 1.5–4.6(–5) mm long, inner stamens usually at the base of the outermost antipetalous pair (0.8–)2.3–3.1 mm, weakly incurved. Antisepalous stamens mostly shorter than outermost antipetalous stamens, sometimes of comparable length, generally 0.6–1.2 mm, weakly to strongly incurved, very rarely a few outcurved. Anthers dorsifixed, 0.38–0.63 × 0.18–0.32 mm, ellipsoid to ovoid-ellipsoid or deltoid, latrorse. Pollen white (9.1–)14.7(–15.1) μm. Anther connective gland prominent, light pink, salmon pink, yellow to orange when fresh, drying dark orange, orange-brown or dark brown, spheroidal, finely rugulose or papillate. *Ovary* 5(–6) locular, each with 15–26(–36) ovules in two rows on each placental lobe. Style 2.0–2.5(–3.5) mm long at anthesis, elongating slightly after anthesis, white or pinkish-white; stigma broadly capitate, at least 1.5× style diam., flat, greenish-white or pale pink, flushing red after anthesis, surface finely granular-papillate. *Fruits* mostly all falling within 1–2 months of seed dehiscence, but a few long persistent, (2.2–)3.8(–4.6) × (3.2–)4.0(–5.3) mm, initially dark green to chesnut-brown fading with age to greyish white, obconic, broadly obconic to ± turbinate, rarely cupular; veins and ribs ± conspicuous on drying; external surface distinctly hairy, very rarely glabrescent or glabrous; hairs short to long antrorse-appressed; calyx valves incurved, splits concealed by dried, erect, free portion of hypanthium. *Seeds* 0.9–1.0(–1.1) × 0.35–0.40(–0.48) mm, oblong, oblong-obovate, oblong-elliptic, curved near apex, laterally compressed, 2–3-angled with convex to flattened faces, apex rounded to subacute, base oblique, ± flattened; testa semi-glossy, orange-brown to dark brown, surface coarsely reticulate. FL: (Aug–)Nov–Jan–Feb(–Jun). FT: (Jul–)Feb–Apr(–May). Chromosome Number *n* = 11_II_, 2*n* = 22, 23 (see de Lange and Murray 2004).

#### Representative specimens

**(620 sheets seen).** **New Zealand (North Island).** Mangamuka Gorge Scenic Reserve, P. J. de Lange 4138, 17 Jan 2000, (AK 287965); Maropiu, Omamari Road, Te Kawa Stream, P. J. de Lange 4202 & L. J. Forester, 21 Jan 2000, (AK 288034, Duplicate: AD); Pakiri, Rahuikiri Beach Road, P. J. de Lange 5532 & G. M. Crowcroft, 5 Oct 2002, (AK 283235); Ponui Island, unnamed stream draining south to Motunau Bay, P. J. de Lange 6688 & E. K. Cameron, 16 Oct 2005, (AK 297493), Auckland City, Western Springs, P. J. de Lange 4619 & M. A. Crowcroft, 30 Oct 2000, (AK 288078, Duplicate: AD); Mauku, Bald Hills, Manukau, H. Carse s.n., 15 Nov 1901, (CHR 296314); Coromandel Peninsula, Moehau Range, Little Moehau, P. J. de Lange 4742, 2 Dec 2000, (AK 287038, Duplicate: AD); Whangamarino, Falls Road, P. J. de Lange 4015, 23 Nov 1999, (AK 242673); Hamilton City, Waikato River, Delamere Street, P. J. de Lange 1195, 6 Jan 1992, (AK 207191, Duplicates: AD, BISH, CHR, HO); Te Kauri Scenic Reserve, Waikuku Valley, Devlin Track, P. J. de Lange 4265 & P. de Lange, 29 Jan 2000, (AK 286134, Duplicates: AD, NSW); Kohioawa Beach, Ohinekoao Cliffs, P. J. de Lange 5325, 25 Oct 2001, AK 287041 (Duplicates: AD, HO); Whirinaki Forest, Arohaki Lagoon, southern end, P. J. de Lange 6690 & P. B. Cashmore, 15 Mar 2006, (AK 297496); Taupo, Rotongaio, R. H. Steele s.n., 7 Feb 1964, (WELTU 3048); 3 km east of Matiere, P. J. de Lange 4337, 31 Jul 2000, (AK 286126, Duplicates: AD, MEL); Mt Egmont National Park, Mt Taranaki/Egmont, Dawson Falls (Manaia Road End), J. Clarkson s.n. & S. Caldwell, 26 Jan 2007, (AK 298312, Duplicate: AD, CHR, MEL, NSW, WELT); Lottin Point Road, Otanga, P. J. de Lange 4649, 8 Nov 2000, (AK 288520, Duplicates: AD, MSC, P, WAIK); Gisborne Plains, Mangaoporo River, P. J. de Lange 4653, 8 Nov 2000, (AK 286067, Duplicate: AD, WELT); Mahia Peninsula, Whangawehi Stream, P. J. de Lange 4660, 9 Nov 2000, (AK 286161, Duplicate: AD); Hawke’s Bay, Tangoio, Te Ngaru Stream, P. J. de Lange 4670, 9 Nov 2000, (AK 286251, Duplicate: AD, CHR); 4 km southwest of Horopito, above Makotuku River, P. J. de Lange 4245 & N. J. D. Singers, 27 Jan 2000, (AK 288038, Duplicate: AD); Kawhatau River, Kawhatau Valley Road, P. J. de Lange 4379, 10 Aug 2000, (AK 288076, Duplicate: AD); Whangaehu River Mouth, Whitiau Scientific Reserve, C. C. Ogle 4887 & A. Dijkgraaf, 15 Dec 2004, (AK 297361); east of Puketoi Range, Waihoke, A. P. Druce s.n., Dec 1973, (CHR 273332); eastern Wairarapa, 3 km west of Whakataki, P. J. de Lange 649 & G. M. Crowcroft, 9 Jan 1991, (AK 202104, Duplicate; AD); Upper Hutt, Trentham, W. M. Fleming s.n., 6 Jan 1965, (WELTU 17697). **Poor Knights Islands.** Tawhiti Rahi, near Puketuaho, G. R. Parrish s.n., 18 Sep 2006, (AK 297487, Duplicate: AD). **Mokohinau Islands.** Motukino (Fanal Island), E. K. Cameron 7717 & P. J. de Lange, 15 Sep 1994, (AK 226069); **Hauturu (Little Barrier Island).** Te Maraeroa, J. P. Burrell s.n., Dec 1962, (OTA 7350). **Aotea (Great Barrier Island).** Whangaparapara Road, near Stamping Battery Remains, P. J. de Lange 4546 & G. M. Crowcroft, 8 Oct 2000, (AK 251367, Duplicate: AD). **New Zealand (South Island).** Wangapeka Valley, R. Mason s.n., 22 Dec 1946, (CHR 58115); Fairdown, P. J. de Lange 4783 & P. I. Knightbridge, 4 Dec 2000, (AK 288488, Duplicate: CHR); D'Urville Island, Catherine Cove, Kiangawari, Pylon Track, P. J. de Lange 5057 & G. M. Crowcroft, 19 Jan 2001, (AK 288511, Duplicate: AD); Queen Charlotte Sound, Green Bay, P. B. Heenan s.n., 8 Jan 2004, (CHR 569892); Rarangi - Golf Links Road, P. J. de Lange 5115, 23 Jan 2001, (AK 288571, Duplicate: AD); Waima (Ure) River, Ure Road, P. J. de Lange 5448 & G. M. Crowcroft, 4 Nov 2001, (AK 286221); Motunau Settlement Road, Water Supply Creek, QE II Covenant, P. J. de Lange 5104, 21 Jan 2001, (AK 288517) (Duplicate: AD); Port Levy, Banks Peninsula, B. H. Macmillan 66/4, 1 Jan 1966, (CHR 166497); Grey River, Atarau Road, P. J. de Lange 4809 & P. I. Knightbridge, 8 Dec 2000, (AK 288289, Duplicates: AD); Old Christchurch Road, nr Okuku Reservoir, Kawhaka Road, P. J. de Lange 4811 & P. I. Knightbridge, 8 Dec 2000, (AK 288288, Duplicates: AD, CHR, HO); Lake Wanaka, T. Kirk 957, 6 Jan 1877, (WELT SP029439); Trotters Gorge, P. B. Heenan s.n., 9 Jan 2003, (CHR 567749); Otago Peninsula, Pikikaretu Beach, J. P. Burrell K25, 21 Mar 1962, (OTA 7376); Taieri River Mouth, J. P. Burrell K23, 6 Feb 1962, (OTA 7370); Dunstan Mountains, Bendigo, J. P. Burrell s.n., 26 Dec 1961, (OTA 7362); Roxburgh Dam, near Chasm, L. B. Moore s.n., 17 Dec 1969, (CHR 132250); Queensberry, A. P. Druce APD1388, Jan 1992, (CHR 471980); Clutha River, South of Balclutha, J. P. Burrell K15, 14 Feb 1962, (OTA 7367).

#### Distribution

**Figure 59.**
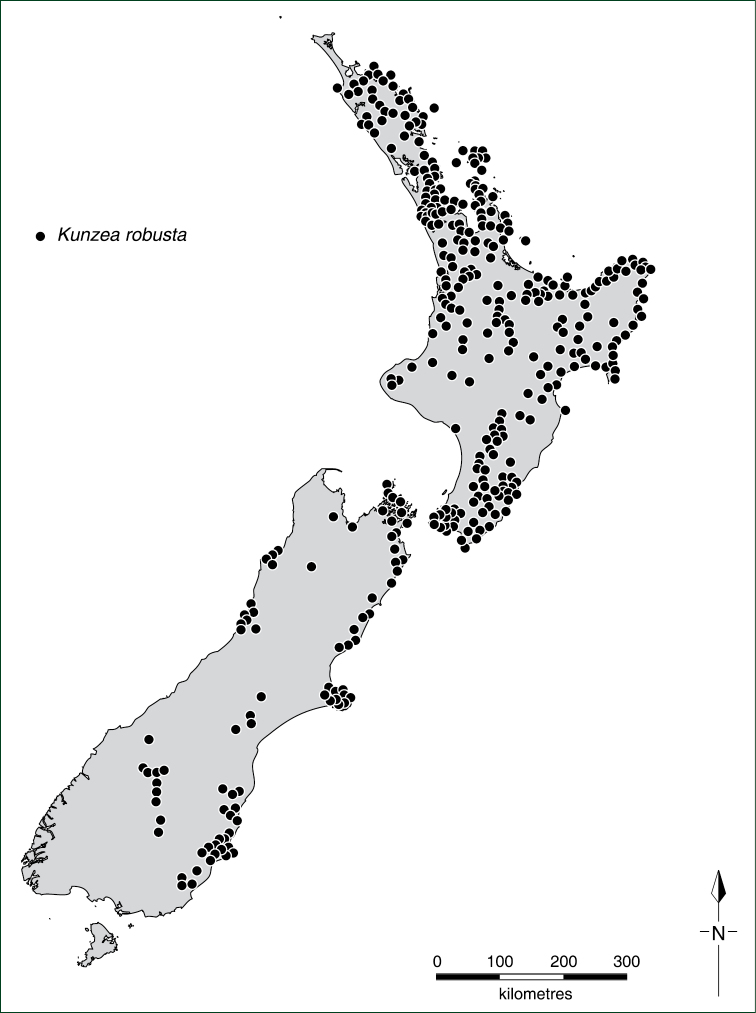
Distribution of *Kunzea robusta*.

**(Fig. 59).** Endemic. New Zealand, North and South Islands (sea level – 1000 m a.s.l.). In the North Island widespread with the exception of Te Paki and the sand tombolo of Te Aupouri. Scarce in Taranaki, from where de Lange (2006) (treated there as Kunzea aff. ericoides (B)) had erroneously stated that the species was absent from Mt Taranaki/Egmont because at that time the populations of *Kunzea* on that mountain were believed to represent another allied, but potentially distinct, species, Kunzea aff. ericoides (f) (see de Lange and Murray 2004; de Lange et al. 2005). In the southern one-third of the North Island, it appears to be absent from Kapiti Island from where only *Kunzea amathicola* has thus far been collected. In the South Island, *Kunzea robusta*, although wide ranging, is often absent over large parts of seemingly suitable habit. It is also naturally absent from most of north and south-west Nelson where it is replaced by *Kunzea ericoides* and, in the extreme north-west, *Kunzea amathicola*. However, occasional trees and stands grow near Wangapeka and in places along the Buller River. It was also planted around Totaranui, Abel Tasman National Park from where it began to naturalise.Those plantings have now been eradicated. On the West Coast, in an area centred on Fairdown, Westport, and Cape Foulwind and also within the lower Grey River catchment, *Kunzea robusta* is locally abundant. It also grows to the west of the main divide along the upper Ahaura River, below Mt Ranunculus, and on the foothills of the Alexandra Range. South of here, *Kunzea robusta* is scarce with only isolated, mainly roadside stands present near Kumara and Hokitika. The close association of these stands to roadsides suggests that the stands may not be natural, or that the species has benefited from the frequent disturbance caused by road construction and ongoing maintenance. Occasional trees of this species have also been collected from Okarito (e.g., B. H. Macmillian 97/22 & E. H. Woods (CHR 512939)) where they occur as planted specimens and from which source it is now naturalising. In the eastern South Island this species is more widespread, though initially strictly as a coastal and lowland tree of the Marlborough Sounds south to about North Canterbury. In North Canterbury, *Kunzea robusta* occasionally extends well inland up the river valleys where it is sympatric with and eventually replaced by *Kunzea serotina*. South of there, on Banks Peninsula, *Kunzea robusta* seems to be the only species present, while on the adjacent Canterbury Plains it is completely replaced by *Kunzea serotina* until, on the slightly more elevated foothills of south Canterbury, *Kunzea robusta* reappears as a local dominant. In north-eastern Otago, *Kunzea robusta* is common around Trotters Gorge and the Horse Range but south of here it has an otherwise mainly coastal distribution, reaching its greatest abundance around Dunedin and on the adjacent Otago Peninsula. A few inland locations are known, especially around Lakes Hawea and Wanaka, where the species is sympatric with *Kunzea serotina*. *Kunzea robusta* is also common along the northern and eastern foothills of the Dunstan Range south of which it occurs only very locally, in isolated patches along the Clutha River as far south as Kaitangata and Balclutha. These southerly outliers are not only the southern limit for the species but also for the genus worldwide.

#### Recognition.

The potential distinctiveness of *Kunzea robusta* was first recognised in New Zealand by William Colenso (1811–1899) who collected specimens of it from the Pahawa [Pahaoa] River, eastern Wairarapa (W. Colenso 2011, (WELT SP022862, Duplicate: K). Obviously impressed by its sturdy habit, Colenso regarded it as a new species for which he proposed the name “Leptospermum pahawaense” to J. D. Hooker. However, Hooker never took up the name (Hooker 1867). In hindsight it is intriguing that New Zealand botanists have failed to recognise that *Kunzea robusta* is distinct from *Kunzea ericoides*. It would seem that this has come about for two main reasons. Firstly, because the type of *Leptospermum ericoides* (≡ *Kunzea ericoides*) was lodged in an overseas herbarium (P), where it was not easily accessible to New Zealand-based botanists, and so until now had not been critically examined. Secondly, *Kunzea robusta* is the most common of the *Kunzea ericoides* complex, and, in the absence of a critical evaluation of the type, it is understandable that New Zealand botanists have come to assume that this common tree was the one described by Richard (1832). Even so, recourse to Richard’s protologue does provide a very clear description of *Kunzea ericoides sens. str.*, which should have enabled the recognition of *Kunzea robusta* long before now. For example, Richard clearly stated that *Leptospermum ericoides* (≡ *Kunzea ericoides*) had glabrous stems, and leaves (except for the leaf margins), glabrous pedicels, glabrescent calyces and capsules. Even allowing for the fact that *Kunzea ericoides* does have minute hairs on its branches, they are so small and sparingly distributed that they are easily missed, so Richard’s use of ‘glabrous’ in that context makes sense. Irrespective, any *Kunzea* with conspicuously hairy stems, leaves, pedicels and fruits could not fit Richard’s species. Despite this, and probably because it was so widespread and common, *Kunzea robusta* remained unrecognised, ironically ended up serving as the ‘type species’ from which Thomas Kirk and George Simpson segregated their Leptospermum ericoides var. lineare (≡ *Kunzea linearis*), *Leptospermum sinclairii* (≡ *Kunzea sinclairii*) and Leptospermum ericoides var. microflora (≡ *Kunzea tenuicaulis*).

As circumscribed here, *Kunzea robusta* remains a very variable species. It is primarily a coastal to lowland, rarely montane, arborescent species (Fig. 58A–G), which normally reaches heights of between 20–25 m tall, and 0.65 m d.b.h. (Fig. 58B, C). Occasional trees attaining heights of up to 30 m, and trunks up to 1 m d.b.h. are known from the North Island (Northland, Aotea (Great Barrier Island) and the eastern Wairarapa), making this species the largest and tallest in the genus (Fig. 58B–D). The bark of *Kunzea robusta* is typically extremely coriaceous, and is characteristically shed in long tabular strips up to 4 m long (Fig. 58H–M) that often hang in a loose skirt around the trunk. The upper surface of these strips is usually intact, with little secondary peeling (Fig. 58M), though trees growing in heavy shade, or wetter than usual conditions may have thinner, subcoriaceous bark, and some secondary peeling. The adult branchlet indumentum of *Kunzea robusta*, for most of its range, consists of antrorse-appressed, silky hairs (Fig. 55A–C), though hair length can be extremely variable. As a rule, *Kunzea robusta* gathered from montane situations, and from the drier eastern side of both islands, tend to have shorter hairs, which may require a hand lens of at least 10× magnification to see, while those from the northern half of the North Island, Marlborough Sounds, Banks Peninsula and Dunedin, particularly specimens growing in coastal to lowland sites, tend to have visibly longer antrorse-appressed branchlet hairs (Fig. 55A–C). Nevertheless, despite these geographic generalisations, it is not uncommon for two trees growing side by side to have either short or long hairs. The mature leaves of this species, though variable in size and shape (Fig. 54L, M, P–Q), are unified in being consistently glabrate with the lamina margin and abaxial midrib sparsely to conspicuously silky hairy (Fig. 54N–O), with the hairs only very rarely meeting at the leaf apex (Fig. 54O). The adaxial and abaxial leaf surfaces of *Kunzea robusta* are conspicuously glandular punctate (Fig. 54L–M), with the adaxial surface characteristically dull rather than glossy green. *Kunzea robusta* has extremely variable pherophylls both within and between populations. These range from squamiform to foliose, with either one or both types found on any particular individual. Irrespective of type, they are almost always shed during flowering. However, in some populations the foliose pherophylls are retained below some (but not all) flowers, and in these cases the pherophylls of any particular individual can range from elliptic, through oblanceolate, to broadly lanceolate or lanceolate. The inflorescence of *Kunzea robusta* is initially corymbiform, and in most places (see below for exceptions) this changes toward the end of the initial flowering through activation of the terminal vegetative bud (Fig. 53A). The subsequent vegetative growth partially elongates the initial inflorescence, and so in this species (more than any other of the species possessing an initially corymbiform botryum) the vegetative growth usually progresses into a further late season flowering as a distinctively elongated botryum. However, in the eastern Coromandel Peninsula and in coastal sites from East Cape south to the sand tombolo connecting Mahia Peninsula to the mainland, the late season elongated botrya of *Kunzea robusta* do not appear to be developed. Inflorescences of plants in these areas (called here the “eastern North Island variant”) remain corymbiform (Fig. 53C), the apical vegetative bud of each inflorescence appearing to abort, and so each flowering episode is marked by a further series of brachyblasts bearing inflorescences, with vegetative growth restricted to the primary axis of the main terminal branchlet of each branch system. The fruits of *Kunzea robusta* are mostly obconic to broadly obconic (Fig. 53I) and are on average the longest (up to 4.6 mm long), and, next to *Kunzea amathicola*, the widest of the New Zealand species (up to 5.3 mm wide). Further, they are usually uniformly hairy, a character shared among usually obconic-fruited species only by the decumbent shrub, *Kunzea sinclairii*.

*Kunzea robusta* has a consistent chromosome karyotype comprising four large (2.0–2.5 μm), six intermediate (1.5–1.8 μm) and one small (0.6 μm) set of chromosomes (de Lange and Murray 2004). With the exception of Mt Egmont samples, the combined sequence data obtained from the ITS and ETS marker regions showed that *Kunzea robusta* had the same sequence as *Kunzea sinclairii* (de Lange 2007). The only variable character in the ETS sequence of *Kunzea robusta*, a guanine, was shared with *Kunzea amathicola* and *Kunzea sinclairii* (Table 2). Three samples of *Kunzea robusta* from Mt Egmont National Park provided the only departure from this pattern, possessing a guanine/cytosine mix (Table 2). The same mix is shared with *Kunzea ericoides*, *Kunzea linearis*, *Kunzea salterae*, *Kunzea serotina* and *Kunzea toelkenii* (Table 2; see also de Lange 2007). Previously, de Lange and Murray (2004) and de Lange et al. (2004) had regarded the Mt Egmont *Kunzea* populations as comprising another, potentially unnamed species, Kunzea aff. ericoides (f). This decision was based in part on the presence of this guanine/cytosine mix but also because of the behaviour of cultivated Mt Egmont plants, which retained their glaucescent, juvenile foliage despite flowering. However, this juvenile form is not retained in the wild, and adult trees are otherwise indistinguishable from the more usual form of *Kunzea robusta*. It was concluded that the neotonic form and flowering of the cultivated plants was probably induced through these plants (in effect 2 m tall individuals) becoming root-bound and stressed rather than having a genetic basis. To confirm this two of the Egmont *Kunzea* were planted and these ceased flowering and developed ‘adult’ foliage (i.e., foliage typical of *Kunzea robusta* elsewhere) within one year of planting. Finally, the chromosome complements of the Egmont plants, which were not reported on by de Lange and Murray (2004), are consistent with those of all other *Kunzea robusta* populations. Therefore, Mt Egmont plants, despite the apparently anomalous, persistent juvenile form exhibited by cultivated specimens grown at the University of Auckland, is treated here as *Kunzea robusta*.

Nevertheless, some regional variants within *Kunzea robusta* can be distinguished. However, as these tend to intergrade across wide parts of their range with other forms of *Kunzea robusta* and I can find no clear zones of sympatry or ecological partitioning, I feel that little purpose is served by further formal taxonomic division of this species. That said, one notable variant may repay further study. This variant, called here the “Rangitikei variant” is mostly found within the Rangitikei River catchment in the calcareous siltstone (‘papa’) country from about Hunterville and Umutoi north to near Moawhango. This variant differs markedly from other *Kunzea robusta* with respect to the branchlet indumentum (Fig. 56) and leaf size of the seedlings and juveniles. While *Kunzea robusta* is markedly heteroblastic, the usual condition (with the exception of some eastern North Island populations that may have smaller linear to linear-lanceolate leaves) is for the juvenile to have much larger, laxer, oblanceolate leaves than the adult. Even so, the juveniles consistently still have the long, antrorse-appressed branchlet indumentum typical of the species throughout most of its range. In the Rangitikei this is not the case. There, juveniles have short, divergent hairs (Fig. 56A, B) of the form seen also in *Kunzea serotina* (see Fig. 10A–E). Further, as with *Kunzea serotina*, they typically have an erect, more or less pyramidal growth habit, though with more broadly spreading branches than is the case in *Kunzea serotina*. The juvenile leaves of these *Kunzea robusta* populations are generally smaller than the adult (up to 6.3 × 1.5 mm in the juvenile cf. up to 28.4 × 2.5 mm in the adult). Although particularly distinctive I have retained this variant within *Kunzea robusta* because this juvenile condition is lost in the adult which develops the more usually antrorse-appressed branchlet hairs typical of the species elsewhere (Fig. 56C–H), and because the rDNA ITS and ETS sequences, and chromosome complements are typical of *Kunzea robusta* from elsewhere (de Lange 2007; de Lange and Murray 2004). Another variation on branchlet indumentum is seen in the “eastern North Island variant” which occurs mainly in the East Cape area of the North Island. Here, in a narrow band of mainly coastal or near coastal lowland locations from about Oweka south to the northern portion of the sand tombolo of Mahia Peninsula, *Kunzea robusta* populations have mixtures of long antrorse-appressed and short divergent hairs (Fig. 57). These populations also tend to have linear to linear-lanceolate, spreading leaves and corymbiform inflorescences (Fig. 53C). Similar plants also occur along the eastern side of the Coromandel Peninsula and on Tuhua (Mayor Island) but these have only the long, antrorse-appressed indumentum typical of most *Kunzea robusta* populations and corymbiform to shortly elongate inflorescences. Again the DNA sequences and chromosome karyotypes of “eastern North Island variant” conform to the rest of the range exhibited by *Kunzea robusta* (Egmont plants excluded) (de Lange 2007, de Lange and Murray 2004) which precludes, at this stage at least, any further taxonomic subdivision.

Throughout its range *Kunzea robusta* is sympatric with all but the allopatric Three Kings endemic, *Kunzea triregensis*. Branchlet indumentum alone readily distinguishes *Kunzea robusta* from species with divergent and or divergent curled hairs, e.g., *Kunzea ericoides*, *Kunzea salterae*, *Kunzea serotina*, *Kunzea tenuicaulis*. Further detailed distinctions between *Kunzea robusta* and these species are provided under their treatments and in Table 1. Although *Kunzea toelkenii* mostly has divergent hairs, these are characteristically short, curled and/or spiralled and often occur in mixtures with long, appressed-antrorse hairs, so indumentum is not helpful in distinguishing it from *Kunzea robusta*. In *Kunzea toelkenii* it is the multi-trunked growth habit, sinuous, twisted, pendulous branches, and trailing epicormic growth that distinguish it from *Kunzea robusta* in the field. The spiciform inflorescences and sessile to subsessile flowers of *Kunzea linearis* immediately distinguish it from *Kunzea robusta* (see *Kunzea linearis* and Table 1 for further differences). *Kunzea amathicola* differs from *Kunzea robusta* by having consistently elongate inflorescences, and flowers which are subtended by a persistent oblong, oblong-obovate, broadly obovate to elliptic, dark glossy green pherophyll. Other distinctions are offered under *Kunzea robusta* and in Table 1.

#### Ecology.

*Kunzea robusta* is the species that has most usually been described in ecological treatments of “*Kunzea ericoides*” (Burrows 1973; Wardle 1991) because it is the most widespread member of the genus in New Zealand. It is mostly found in coastal and low lying areas and adjacent hill country. It does not usually grow in upper montane situations though it has occasionally been collected in places up to 1000 m a.s.l. Favouring disturbance, this is the species that is most frequently seen colonising marginal hill country, particularly in areas with slip-prone, poorly draining clay soils, or in the clay soils of the drier, drought-prone eastern parts of both islands. It is sometimes regarded as a serious weed in these habitats because of its ability to rapidly reclaim rough pasture land.

*Kunzea robusta* is, as a rule, not common within relatively intact indigenous forest systems, being mostly seen colonising slip scars, and other areas of damage resulting from flooding and/or storm damage. Nevertheless, in some forest types such as that dominated by kauri (Fig. 58A), occasional stands or scattered mature canopy trees can be found with ages of between 200 and 280 years (de Lange 2007). On occasion, such as within the calcareous siltstone-dominated landscapes of the northern Hawke's Bay and Rangitikei, it can form a distinct, forest type. This is probably because the siltstones are naturally prone to frequent slipping, thus maintaining sufficient disturbance to ensure the persistence of this species. *Kunzea robusta* is usually found in association with *Sophora tetraptera* J.S.Mill. (Hawke's Bay) and *Sophora godleyi* Heenan et de Lange (Rangitikei) in these forest types. Sometimes it may be found growing with black beech (*Fuscospora solandri*) in these areas.

As a species, *Kunzea robusta* provides a key habitat for a host of fungi (McKenzie et al. 2006), and the deep leaf litter it produces is also favoured by terrestrial orchids, especially of the genera *Acianthus*, *Caladenia*, *Corybas*, *Gastrodia*, and *Pterostylis*. The branchlets are often heavily parasitised by the dwarf mistletoe *Korthalsella salicornioides*, and in some locations by the green mistletoe (*Ileostylus micranthus*). In many areas, *Kunzea robusta* is the favoured habitat of geckos of the genera *Dactylocnemis*, *Hoplodactylus*, *Mokopirirakau*, *Naultinus*, *Toropuku* and *Woodworthia* (R. Hitchmough pers. comm.).

As a rule the bark of *Kunzea robusta* supports little other vegetation. However, in some sites it can be heavily colonised by lichens, usually of the genera *Coccocarpia* Pers., *Crocodia* Link, *Heterodermia*, *Pannaria*, *Parmeliella* Müll.Arg., *Parmotrema*, *Physcia* (Schreb.) Michx., *Pseudocyphellaria*, *Punctelia* Krog, *Usnea* J.Hill, *Ramalina*, and *Chrysothrix* Mont. *Kunzea robusta* is also host to a range of hornworts, liverworts and mosses. Of these, mosses are typically scarce, though occasionally, such as near the branch bases and forks, growths of *Leptostomum macrocarpa* (Hedw.) Pyl. may be common. Sparse patches of *Hypnum cupressiforme* Hedw., and *Macromitrium* spp., especially *Macromitrium brevicaule* (Besch.) Broth., *Macromitrium gracile* (Hook.) Schwägr., *Macromitrium longipes* (Hook.) Schwägr, and less frequently *Macromitrium submucronifolium* C.Muell. et Hampe may also be found on the mid trunk and branches. The liverwort flora of *Kunzea robusta* is more diverse with 40 species having been recorded from its bark (J. E. Braggins pers. comm.). The most commonly encountered of the liverworts seem to be species of *Acrolejeunea* (Spruce) Schiffn., *Austrolejeunea* (Schuster) Schuster, *Frullania*, *Lejeunea* and *Metzgeria*, followed by *Harpalejeunea latitans* (Hook.f. et Taylor) Grolle, Drepanolejeunea aff. aucklandica, *Metalejeunea cucullata* (Reinw., Blume et Nees) Grolle and *Siphonolejeunea nudipes* (Hook.f. et Taylor) Herzog. In contrast only one species of hornwort, *Dendroceros granulatus* Mitt., is commonly associated with *Kunzea robusta* bark.

#### Hybridism.

*Kunzea robusta* is sympatric with all the other New Zealand *Kunzea* species except the allopatric Three Kings endemic *Kunzea triregensis*. With the exception of that species, and also *Kunzea salterae* and *Kunzea toelkenii*, putative field evidence for hybrids involving the other six species is common, and these hybrid combinations (*Kunzea amathicola* × *Kunzea robusta*, *Kunzea ericoides* × *Kunzea robusta*, *Kunzea linearis* × *Kunzea robusta*, *Kunzea serotina* × *Kunzea robusta*, *Kunzea sinclairii* × *Kunzea robusta*, *Kunzea robusta* × *Kunzea tenuicaulis*) are discussed in detail under the preceeding species and in de Lange (2007). Significantly, these putative hybrid observations are all supported by experimental evidence which showed that hybrids using *Kunzea robusta* (see de Lange et al. 2005, as Kunzea aff. ericoides (b)) as either pistillate or staminate parent were readily produced between all species, except *Kunzea salterae* which was unavailable during that study. All hybrid combinations were successfully raised and flowered, and none showed any reduction in pollen fertility or seed set when selfed.

#### Vernacular name.

It is *Kunzea robusta* which is the species now most frequently meant when people use the name ‘kanuka’. The meaning and origin of the name ‘kanuka’ is however, uncertain (de Lange 2013). The name seems to have first appeared in the East Cape area about 1871 (Orsman 1997). Despite this, the majority of herbarium specimens collected between the 1860 and the 1930s refer to this species (and indeed the *Kunzea* species recognised here collectively) as ‘manuka’ and this is the name by which it is still most commonly referred to by elders of, especially, northern North Island Maori. Interestingly, according to Kirk (1889), Colenso asserted that *Kunzea ericoides* (and in this case he almost certainly meant *Kunzea robusta*) was universally known as ‘manuka-rauriki’ (see also Laing 1907), a name that I have not heard spoken, seen written down on any herbarium specimen, or mentioned in any other literature. Despite the supposed origin of ‘kanuka’ in the East Cape area (Orsman 1997), Kirk (1889) records that, aside from the universal name manuka, in the East Cape area *Kunzea* (and in this case definitely the *Kunzea robusta* described in this paper) was also known as ‘maru’ (Kirk 1889) a name I heard used by Tuhoe elders in Te Urewera National Park in the 1980s. In the South Island, based on early herbarium records (1830s, 1840s) the French also recorded the name ‘titira’ and ‘atitire’ for both *Kunzea robusta* (Akaroa specimens) and *Kunzea ericoides* (Abel Tasman coastline), although these names may simply be a transliteration of ‘ti tree’ as in ‘tea tree’ because even by that time, the fresh and dried leaves of *Leptospermum* and *Kunzea* were routinely being brewed as a tonic by many Maori who had adopted the ‘tea drinking’ practice through observation of and/or intermarriage with European whalers/settlers whose women used these genera as a tea (*Camellia sinensis* (L.) Kuntze) substitute. In the far north of the North Island, aside from ‘manuka’ *Kunzea robusta* is still also occasionally referred to by Ngati Kuri, Te Rarawa, and Nga Puhi iwi as ‘rawirinui’, to distinguish it from the smaller and more slender ‘rawiri’ (*Kunzea linearis*) and ‘rawiritoa’ (*Kunzea amathicola*) (L. Foley pers. comm.).

#### Conservation status.

*Kunzea robusta*, as the most widespread and abundant of the New Zealand *Kunzea*, is here regarded as ‘Not Threatened’ using the criteria of Townsend et al. (2008).

### Incertae sedis

**Figure 60.**
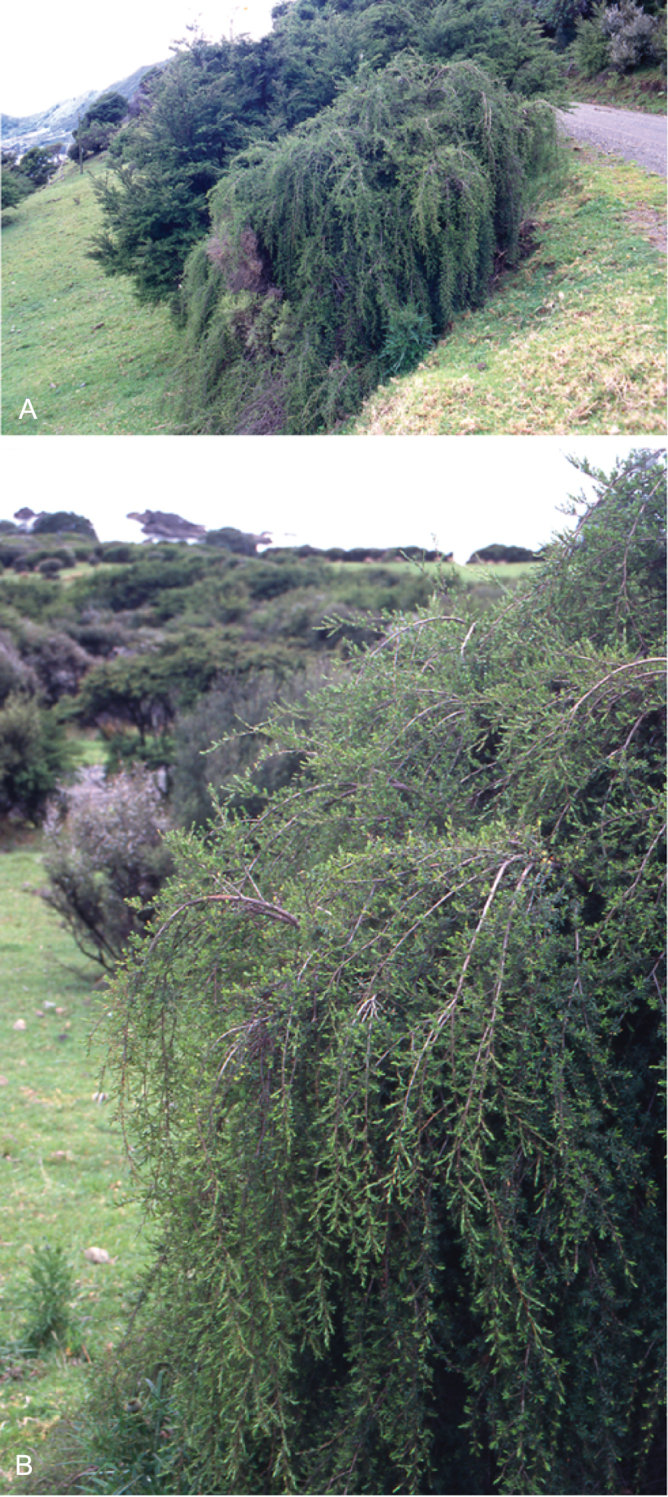
Kunzea aff. ericoides shrub, North Island, Lottin Point road. **A** Showing growth habit of a shrub growing next to *Kunzea robusta* **B** Close up of branches showing distinctive pendulous growth habit.

In November 2000 during a visit to the East Cape Region of the North Island, I discovered growing on the road to Lottin Point amongst otherwise ‘normal’ arborescent *Kunzea robusta*, 20 *Kunzea* with a distinctive multi-trunked, pendulous branched habit forming shrubs up to 2–3 × 3(–6) m (Fig. 7, 60). No flowering specimens were seen. Later in May 2004, a further sterile sample was obtained. At that time three specimens were found, the site having apparently been partially cleared by the landowner (M. Thorsen pers. comm.). As forms of *Kunzea robusta* with pendulous branches are occasionally found throughout that species range, the Lottin Point plant was initially assumed to be similar. However, the presence, at least in November 2000, of 20 specimens of varying sizes and so assumed ages, with the same unusual growth habit, suggested it may be a more widespread genotype worthy of further investigation. Samples from the two known herbarium specimens were sequenced and the results obtained from ITS and ETS markers (Table 2) suggest that the Lottin Point *Kunzea* is potentially distinct and worthy of taxonomic recognition (de Lange 2007; de Lange et al. 2010). Morphologically, aside from growth habit, the plants have leaves most similar to *Kunzea robusta*, though the copiously hairy leaf margin, and in particular the hairs sometimes extending to the leaf tip in mature foliage suggest a relationship with *Kunzea amathicola*.

By 2010 all known plants of the Lottin Point *Kunzea* had been destroyed by land development and a brief survey in 2013 failed to locate further plants in likely sites in the general vicinity. Therefore, at this stage, without flowering material or the opportunity to germinate seed and examine the growth behaviour of the Lottin Point *Kunzea* in cultivation, and without supporting cytogenetic information, formal taxonomic recognition is considered inappropriate. For the purposes of this treatment the Lottin Point *Kunzea* is here regarded as a potentially distinct variant sharing a morphological relationship to both *Kunzea amathicola* and *Kunzea robusta* that, should further plants be located, deserves taxonomic attention.

**Specimens seen.** **New Zealand, North Island (East Cape) Lottin Point.** P. J. de Lange 4650, 8 Nov 2000, (AK 286098, Duplicates: AD, CHR, HO, WELT); M. J. Thorsen s.n., 18 May 2004, (AK 288605, Duplicate: AD).

## Supplementary Material

XML Treatment for Kunzea

XML Treatment for Kunzea ericoides

XML Treatment for Kunzea serotina

XML Treatment for Kunzea tenuicaulis

XML Treatment for Kunzea salterae

XML Treatment for Kunzea toelkenii

XML Treatment for Kunzea linearis

XML Treatment for Kunzea amathicola

XML Treatment for Kunzea triregensis

XML Treatment for Kunzea sinclairii

XML Treatment for Kunzea robusta
